# Supplementary material for: An artificial intelligence accelerated virtual screening platform for drug discovery
Source: Nat Commun. 2024 Sep 5;15:7761. doi: 10.1038/s41467-024-52061-7 (PMC11377542; doi:10.1038/s41467-024-52061-7)
Supplement: Supplementary file 1 — Supplementary Information [file 41467_2024_52061_MOESM1_ESM.pdf]

Supplementary Information:
An artificial intelligence accelerated virtual screening platform for drug discovery

Guangfeng Zhou[1,2,†], Domnita-Valeria Rusnac[3,†], Hahnbeom Park[4,5], Daniele Canzani[6], Hai Minh Nguyen[7], Lance Stewart[2], Matthew F. Bush[6], Phuong Tran Nguyen[8], Heike Wulff[7], Vladimir Yarov-Yarovoy[8,9], Ning Zheng[3,*], Frank DiMaio[1,2,*]

1. Department of Biochemistry, University of Washington, Seattle, WA 98195, USA.
2. Institute for Protein Design, University of Washington, Seattle, WA 98195, USA.
3. Howard Hughes Medical Institute, Department of Pharmacology, University of Washington, Seattle, WA 98195, USA
4. Brain Science Institute, Korea Institute of Science and Technology, Seoul 02792, Republic of Korea
5. KIST-SKKU Brain Research Center, SKKU Institute for Convergence, Sungkyunkwan University, Suwon 16419, Republic of Korea
6. Department of Chemistry, University of Washington, Seattle, WA 98195, USA.
7. Department of Pharmacology, University of California Davis, Davis, CA 95616, USA.
8. Department of Physiology and Membrane Biology, University of California Davis, Davis, CA 95616, USA.
9. Department of Anesthesiology and Pain Medicine, University of California Davis, Sacramento CA 95817, USA

[†] contributed equally

[*]correspondence: nzheng@uw.edu, dimaio@u.washington.edu

Content

Supplementary Methods - The subset of DUD-E
Supplementary Methods - RosettaVS XML of different run modes
Supplementary Fig. 1 | A flowchart of the artificial intelligence accelerated virtual screening protocol.
Supplementary Fig. 2 | CASF2016 scoring power results of all the methods.
Supplementary Fig. 3 | CASF2016 docking power results of all the methods.
Supplementary Fig. 4 | CASF2016 screening power results of all the methods.
Supplementary Fig. 5 | CASF2016 screening power results of all the methods.
Supplementary Fig. 6 | CASF2016 reverse screening power results of all the methods.
Supplementary Fig. 7 | CASF2016 binding funnel analysis for all methods.
Supplementary Fig. 8 | CASF2016 screening power subset success rate.
Supplementary Fig. 9 | The receiver operating characteristic (ROC) curves of DUD targets.
Supplementary Fig. 10 | Examples of the docked poses of DUD from VSH vs. VSX.
Supplementary Fig. 11 | Predicted binding affinities of KLHDC2 virtual screening.
Supplementary Fig. 12 | Predicted binding affinities of Na_V1.7 VSD4 virtual screening.
Supplementary Fig. 13 | Twenty-nine compounds from initial screening of KLHDC2.
Supplementary Fig. 14 | BioLayer Interferometry competition assay of compound 29.
Supplementary Fig. 15 | Twenty-one compounds from the focused screening of KLHDC2.
Supplementary Fig. 16 | Nine compounds from the virtual screening of Na_V1.7 VSD4.
Supplementary Fig. 17 | Whole-cell patch clamp recordings for Na_V1.7 VSD4 compounds.
Supplementary Fig. 18 | Selectivity of inhibitory effect of Z8739902234 against inactivated state, resting state of Nav1.7, Nav1.5 and HERG.
Supplementary Table 1 | Data collection and refinement statistics (molecular replacement).
Supplementary Table 2 | Chemical properties of ordered compounds.

Supplementary Methods

The subset of DUD-E. The subset of DUD-E used for determining the optimal weights for the default entropy model contains ten targets that are randomly selected from the targets with around 200 actives. And these targets are abl1, aofb, cp2c9, def, fpps, hivint, kit, mcr, thb, xiap.

RosettaVS XML of different run modes

Evaluation run mode:

```
<ROSETTASCRIPTS>
  <SCOREFXNS>
    <ScoreFunction name="genpot_soft" weights="beta_cart">
      <Reweight scoretype="fa_rep" weight="0.2"/>
    </ScoreFunction>
    <ScoreFunction name="genpot" weights="beta_cart">
      <Reweight scoretype="coordinate_constraint" weight="1.0"/>
    </ScoreFunction>
  </SCOREFXNS>

  <MOVERS>
    <GALigandDock name="dock" scorefxn="genpot_soft" scorefxn_relax="genpot"
              runmode="eval" turnon_flexscs_at_relax="1" contact_distance="0.0"
entropy_method="Simple" >
    </GALigandDock>

  </MOVERS>

  <PROTOCOLS>
    <Add mover="dock"/>
  </PROTOCOLS>
  <OUTPUT scorefxn="genpot"/>
</ROSETTASCRIPTS>
```

VSX run mode:

```
<ROSETTASCRIPTS>
  <SCOREFXNS>
    <ScoreFunction name="genpot_soft" weights="beta_cart">
      <Reweight scoretype="fa_rep" weight="0.2"/>
    </ScoreFunction>
    <ScoreFunction name="genpot" weights="beta_cart"/>
  </SCOREFXNS>

  <MOVERS>
    <GALigandDock name="dock" scorefxn="genpot_soft" scorefxn_relax="genpot"
          multiple_ligands_file="%%liglist%%" runmode="VSX" premin_ligand="1"
          estimate_dG="1" use_mean_maxRad="1" stdev_multiplier="1.5" nrelax="20"
          padding="4.0" cartmin_lig="0" >
    </GALigandDock>

  </MOVERS>
```

```
  <PROTOCOLS>
    <Add mover="dock"/>
  </PROTOCOLS>
  <OUTPUT scorefxn="genpot"/>
</ROSETTASCRIPTS>
```

VSH run mode:

```
<ROSETTASCRIPTS>
  <SCOREFXNS>
    <ScoreFunction name="genpot_soft" weights="beta_cart">
      <Reweight scoretype="fa_rep" weight="0.2"/>
    </ScoreFunction>
    <ScoreFunction name="genpot" weights="beta_cart"/>
  </SCOREFXNS>

  <MOVERS>
    <GALigandDock name="dock" scorefxn="genpot_soft" scorefxn_relax="genpot"
          runmode="VSH" premin_ligand="1" estimate_dG="1" >
    </GALigandDock>

  </MOVERS>

  <PROTOCOLS>
    <Add mover="dock"/>
  </PROTOCOLS>
  <OUTPUT scorefxn="genpot"/>
</ROSETTASCRIPTS>
```

Example command lines for running RosettaVS VSX:

```
~/Rosetta/main/source/bin/rosetta_scripts.linuxgccrelease \
@ flags_params.txt \
-s complex.pdb \
-extra_res_fa ./complex_lig.params \
-gen_potential \
-overwrite \
-beta_cart \
-parser:protocol dock_vsx.xml \
-parser:script_vars liglist=ligand_list.txt \
-no_autogen_cart_improper \
-multi_cool_annealer 10 \
-missing_density_to_jump \
-score::hb_don_strength hbdon_GENERIC_SC:1.45 \
-score::hb_acc_strength hbacc_GENERIC_SP2SC:1.19 \
-score::hb_acc_strength hbacc_GENERIC_SP3SC:1.19 \
-score::hb_acc_strength hbacc_GENERIC_RINGSC:1.19 \
-out:levels all:300 protocols.ligand_docking.GALigandDock:300 \
-out:prefix prefix. \
-out:file:silent output.out \
```

```
-out:file:scorefile score.sc \
-mute all
```

where flags_params.txt contains:

```
-extra_res_fa molecule1.params
-extra_res_fa molecule2.params
-extra_res_fa molecule3.params
…
-extra_res_fa molecule50.params
```

and ligand_list.txt contains:

```
molecule1
molecule2
molecule3
…
molecule50
```

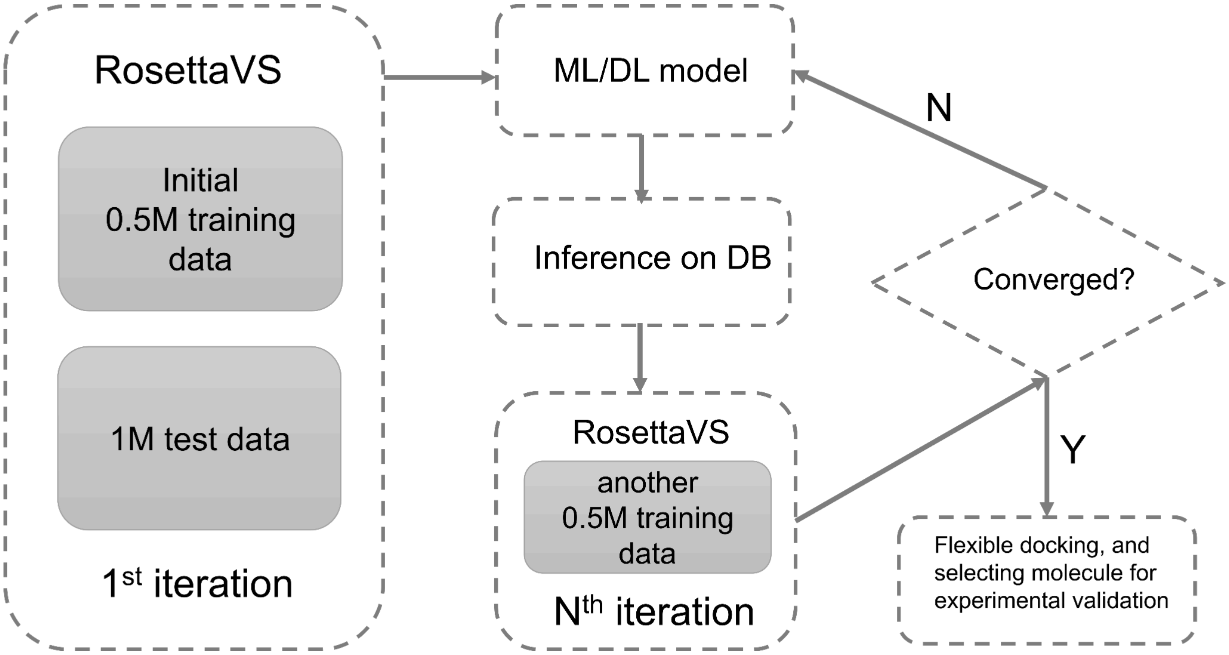

Supplementary Fig. 1 | A flowchart of the artificial intelligence accelerated virtual screening protocol. The number of iteration N is determined by checking if the predicted binding affinities of top ranked compounds have converged. The fully detailed workflow is described in the Methods.

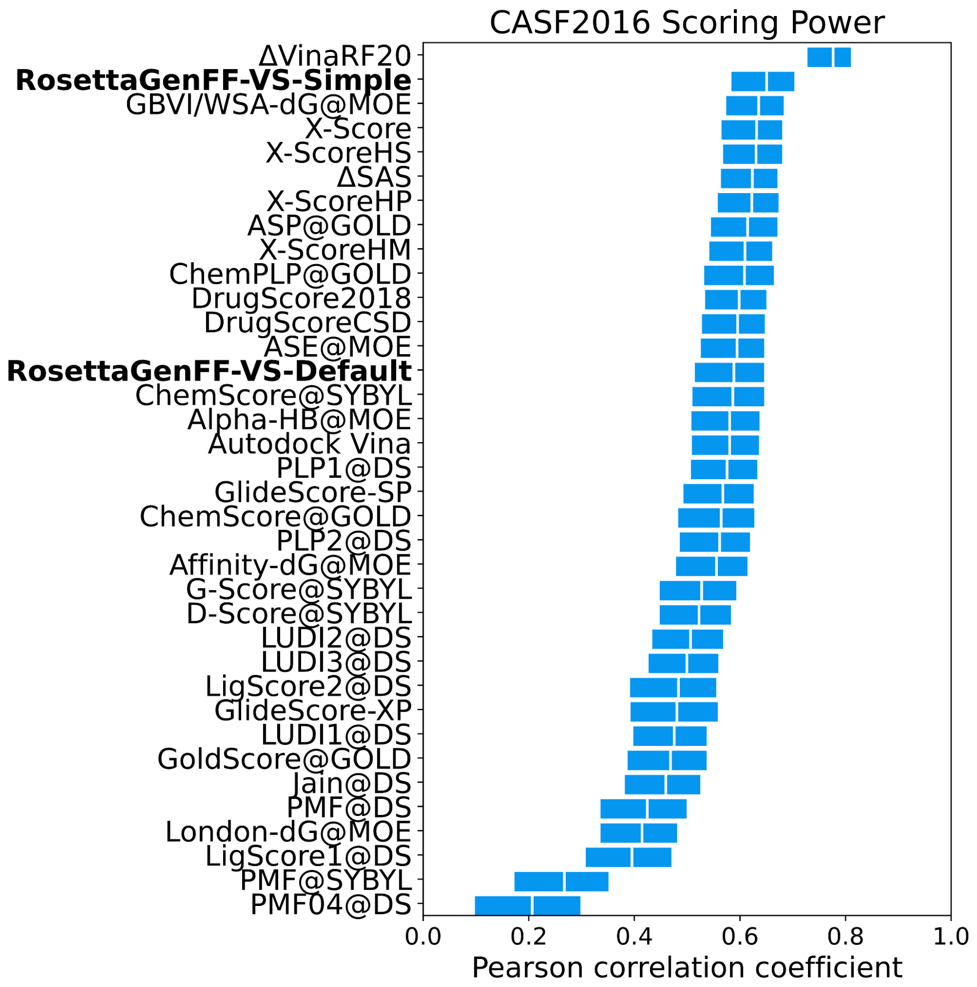

Supplementary Fig. 2 | CASF2016 scoring power results of all the methods. The Pearson correlation coefficients and 90% confidence intervals are shown. These results are obtained on the locally optimized complex structures. Performance of other methods are from Ref[28]. Source data are provided as a Source Data file.

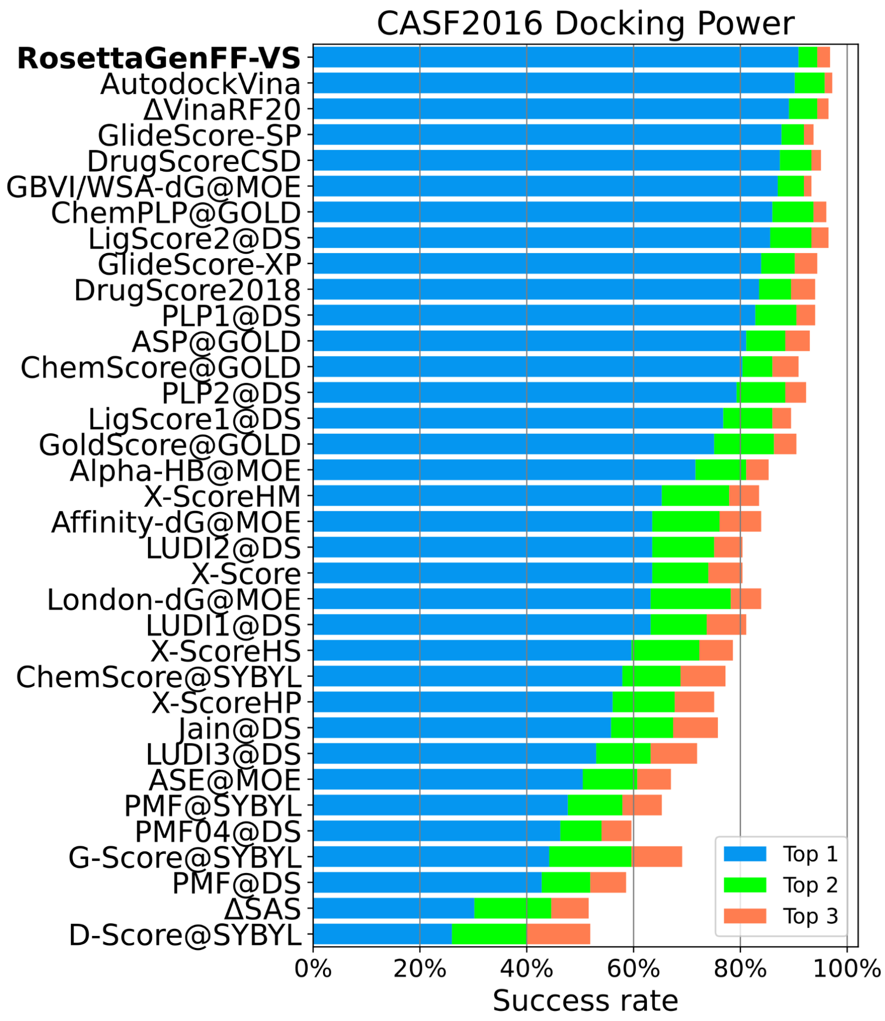

Supplementary Fig. 3 | CASF2016 docking power results of all the methods. Success rate of the top 1/2/3 decoys with less than 2Å rmsd to the native structure. Results are obtained with the native ligand pose included in the decoy set. Docking power of RosettaGenFF-VS does not depend on the entropy model so it is reported without the specification of the entropy model. Performance of other methods are from Ref[28]. Source data are provided as a Source Data file.

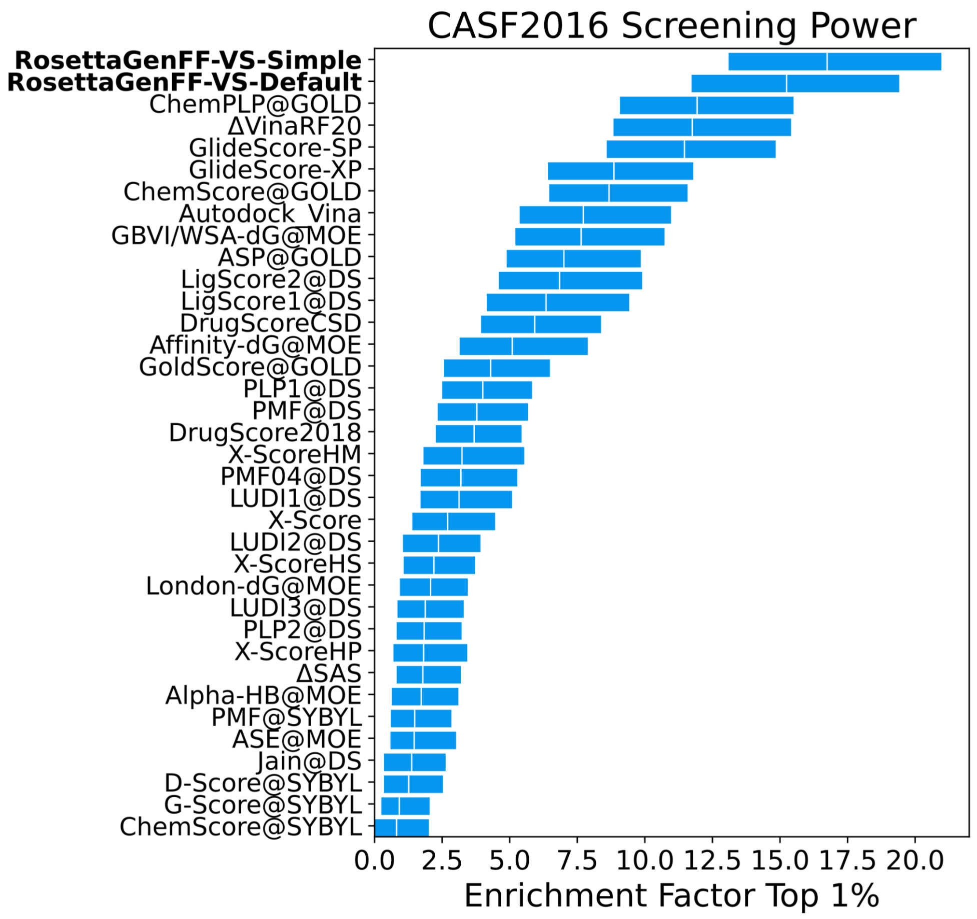

Supplementary Fig. 4 | CASF2016 screening power results of all the methods. The top 1% enrichment factors with 90% confidence intervals of all the methods are shown. Performance of other methods are from Ref[28]. Source data are provided as a Source Data file.

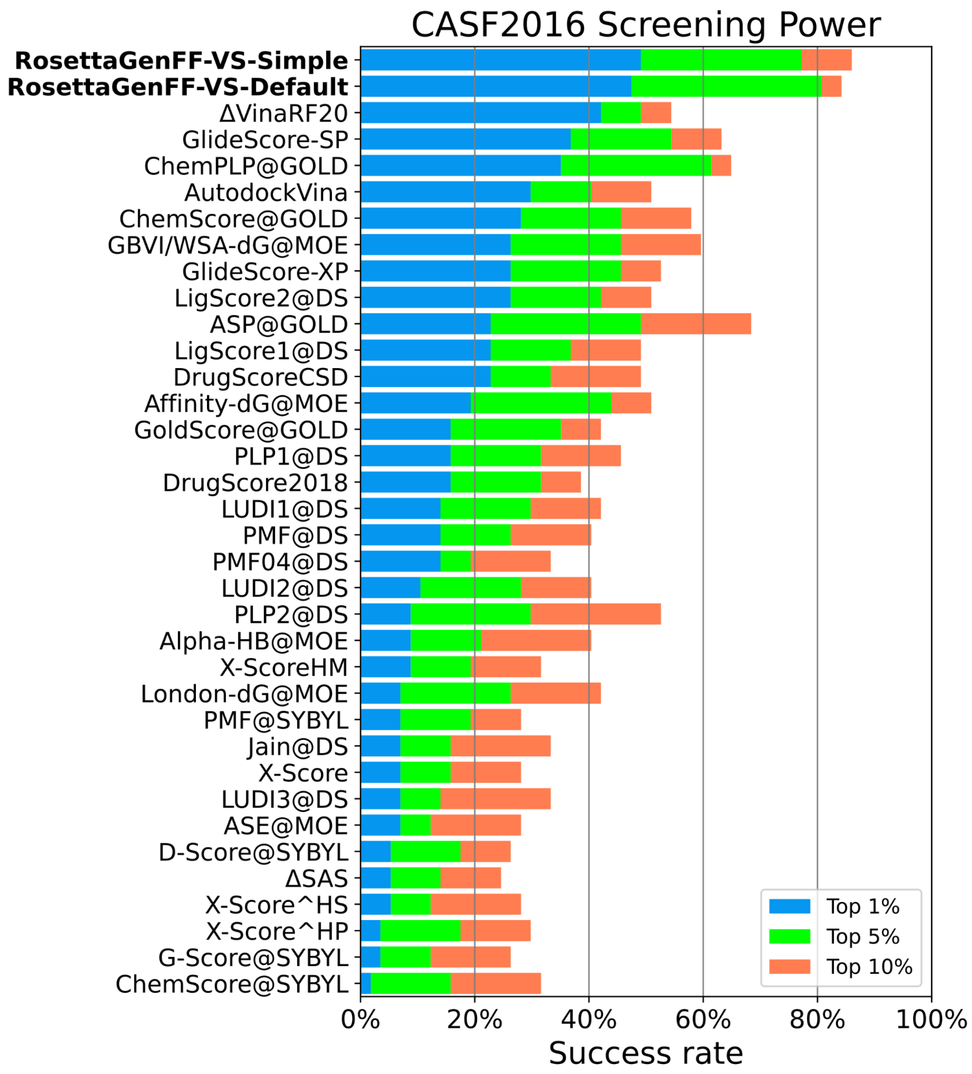

Supplementary Fig. 5 | CASF2016 screening power results of all the methods. The success rate of including the best binder in the top 1/5/10% ranked molecules. Performance of other methods are from Ref[28]. Source data are provided as a Source Data file.

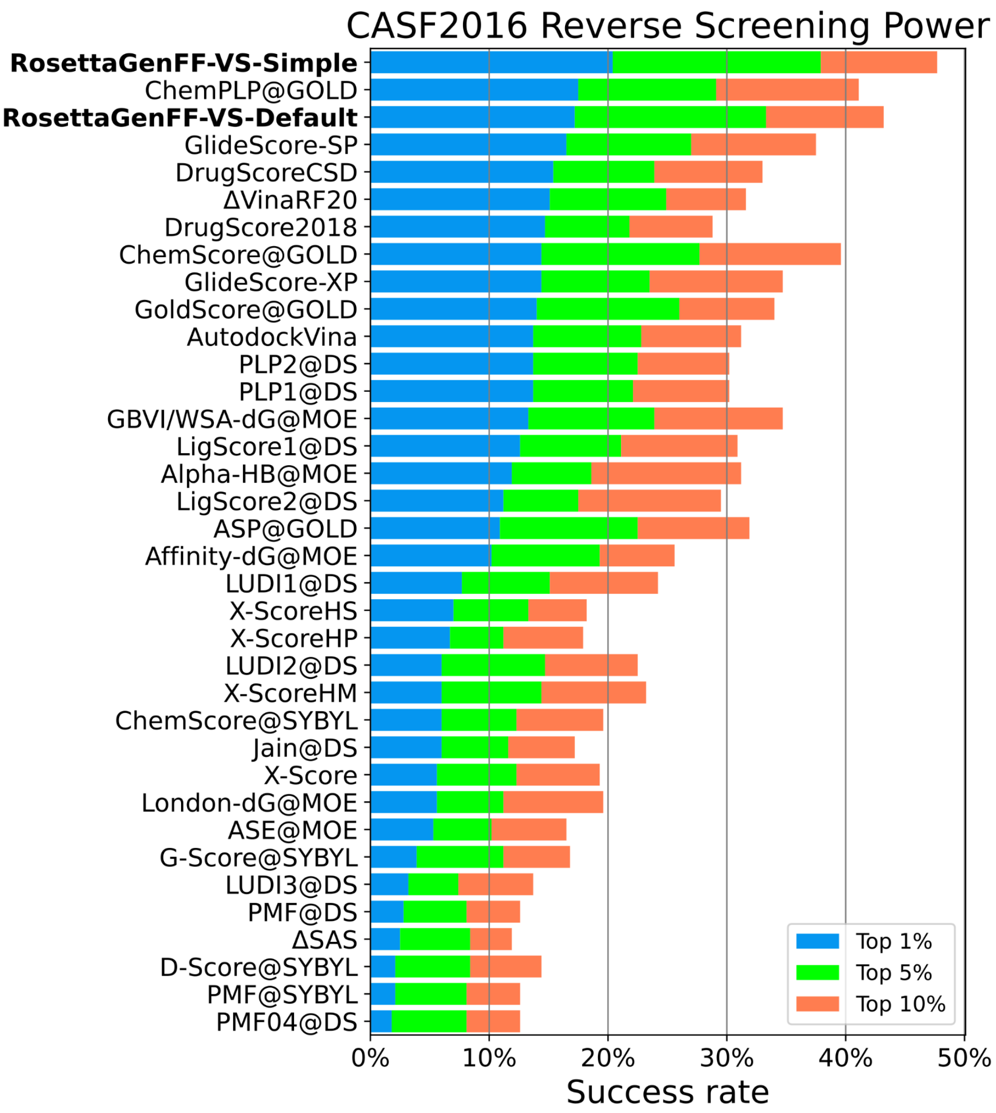

Supplementary Fig. 6 | CASF2016 reverse screening power results of all the methods. The success rate of ranking the best protein target among the top 1/5/10% of all the targets given the ligand. Performance of other methods are from Ref[28]. Source data are provided as a Source Data file.

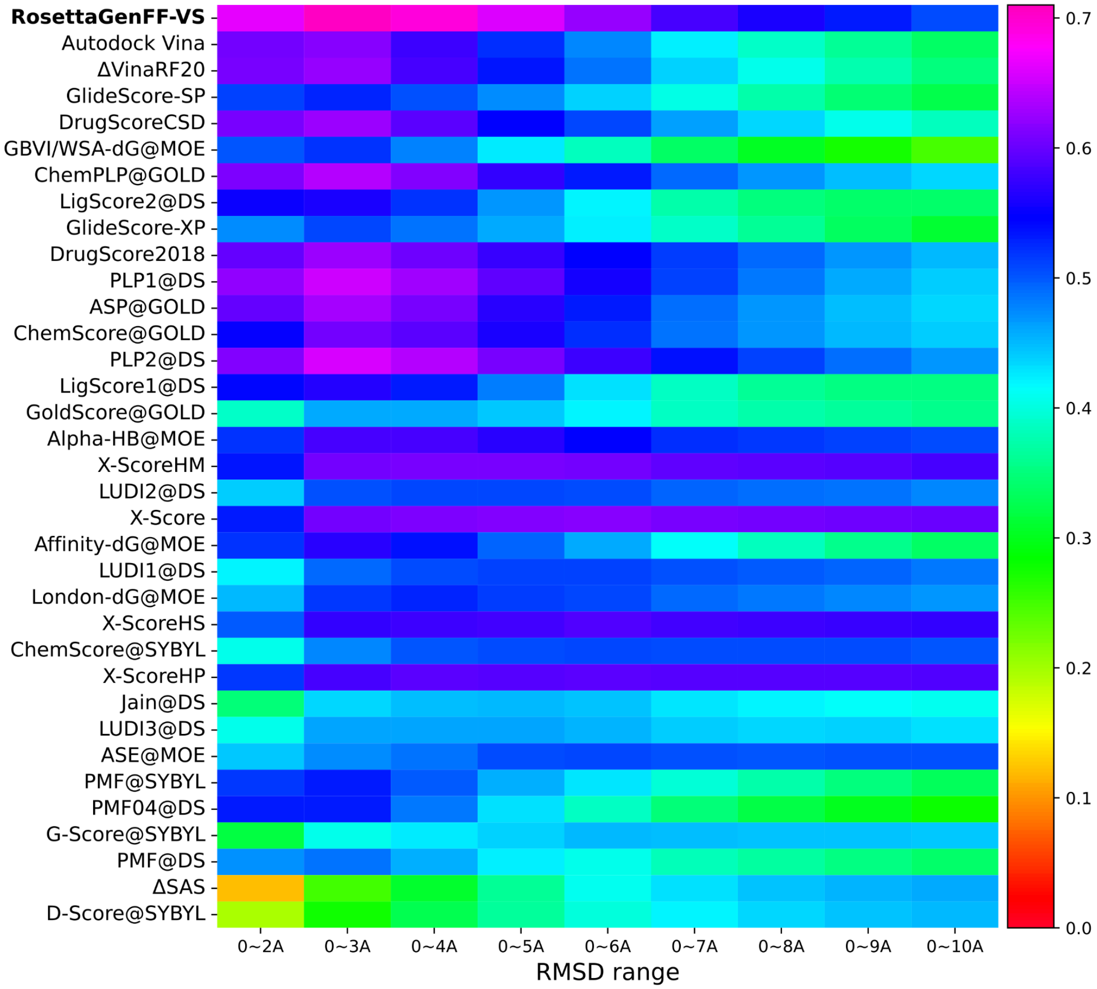

Supplementary Fig. 7 | CASF2016 binding funnel analysis for all methods. The Spearman correlation coefficients between the RMSD ranges and the computed scores are reported. Binding funnels of RosettaGenFF-VS doesn't depend on the entropy model, so it is reported without the specification of the entropy model. Performance of other methods are from Ref[28]. Source data are provided as a Source Data file.

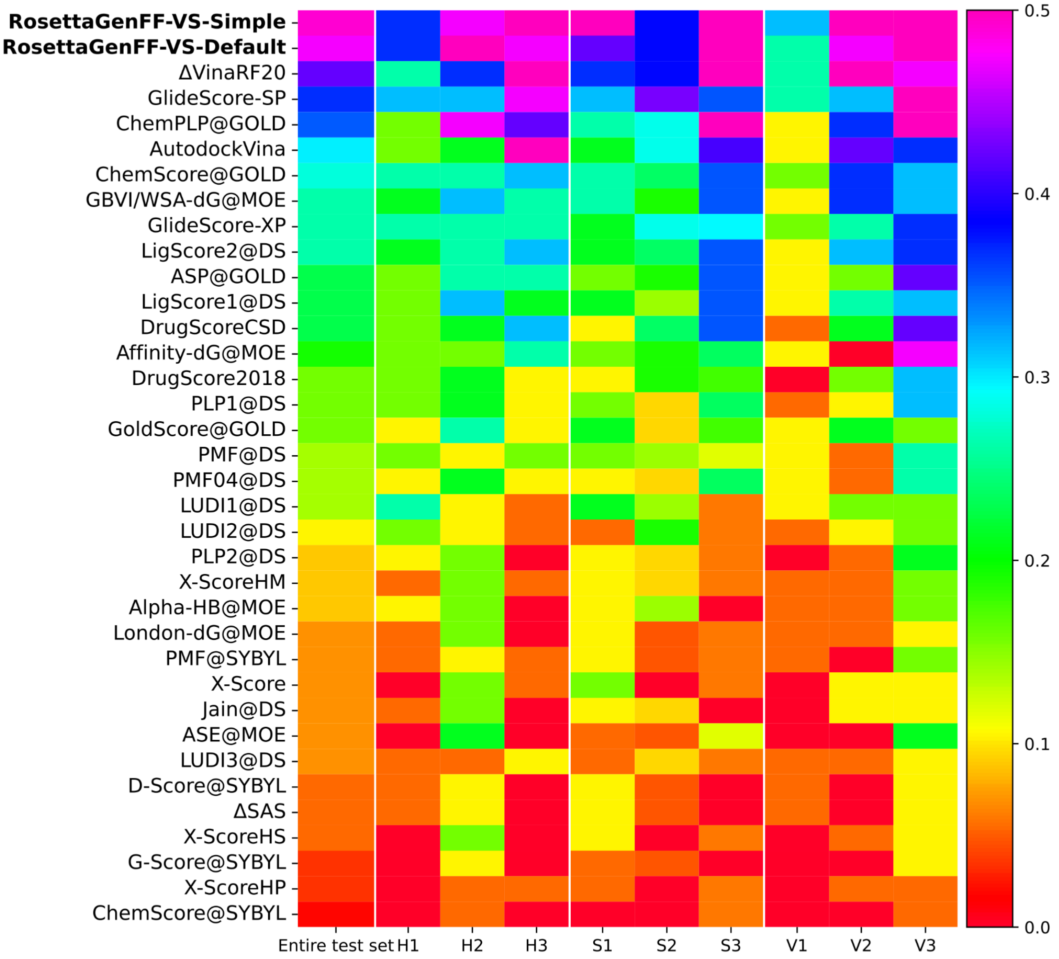

Supplementary Fig. 8 | CASF2016 screening power subset success rate. The success rate of identifying the best-affinity ligand among top 1% ranked ligands for each protein target in the CASF2016 forward screening power test on three subsets. Performance of other methods are from Ref[28]. Source data are provided as a Source Data file.

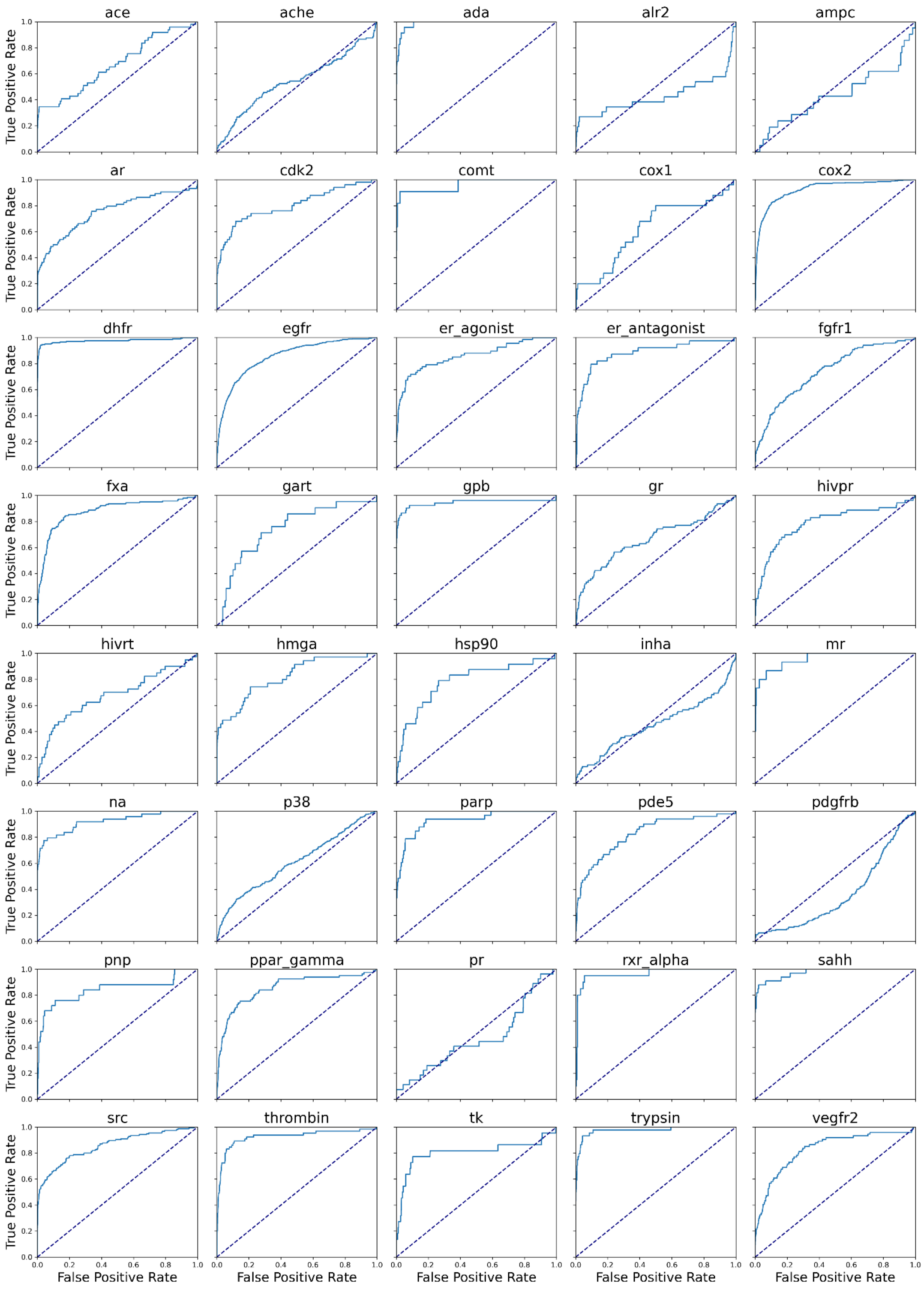

Supplementary Fig. 9 | The receiver operating characteristic (ROC) curves of DUD targets. The virtual screening results are obtained using VSH mode in RosettaVS. Source data are provided as a Source Data file.

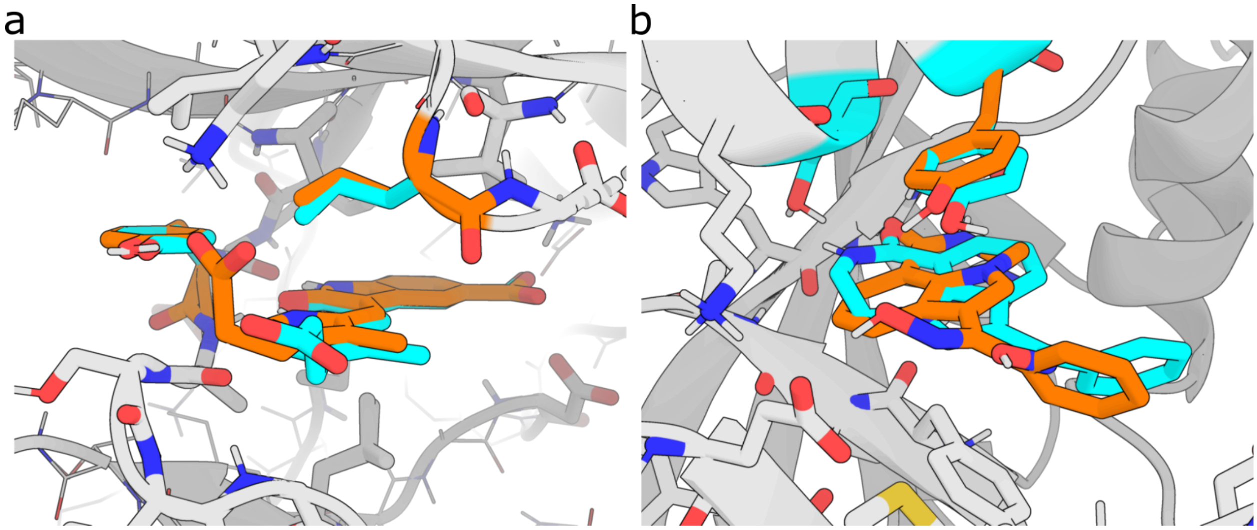

Supplementary Fig. 10 | Examples of the docked poses of DUD from VSH vs. VSX. In both cases, VSH (orange) predicted a better docking pose than VSX (cyan) and ranked the ligand among top 1% due to predicted movement in receptor sidechains. The target and ligand ID is (a) fgfr1 and ZINC03815565, and (b) parp and ZINC03832197.

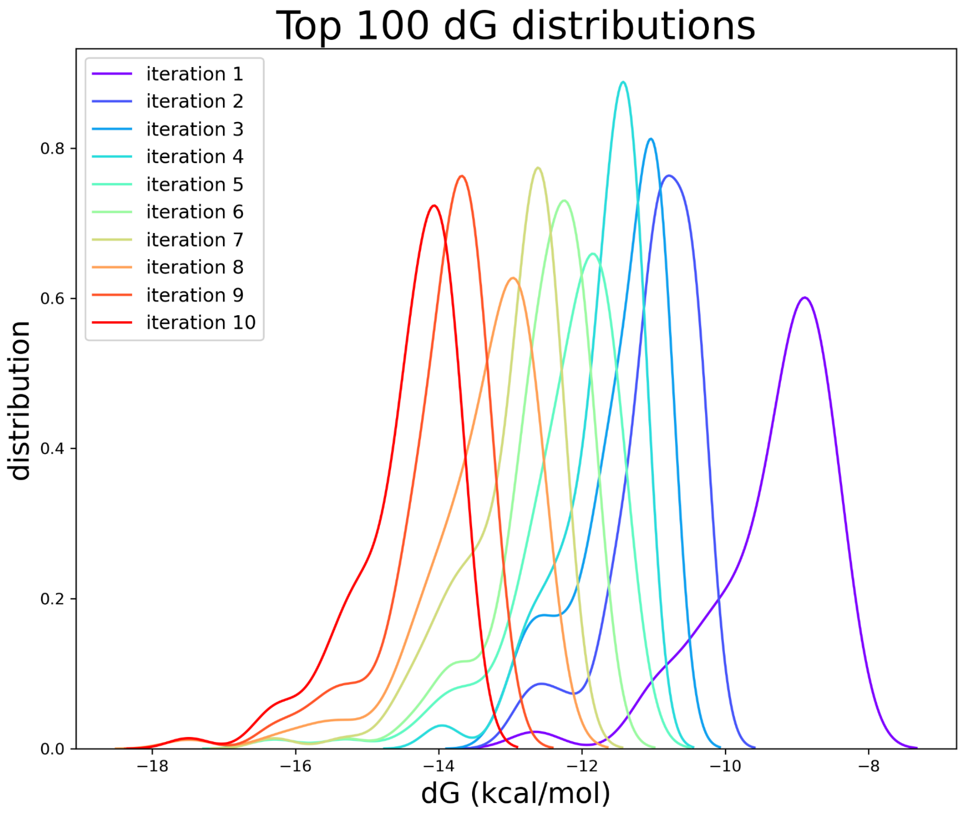

Supplementary Fig. 11 | Predicted binding affinities of KLHDC2 virtual screening. The distributions of the predicted binding affinities of the top 100 ranked molecules after each iteration of KLHDC2 virtual screening are shown. Source data are provided as a Source Data file.

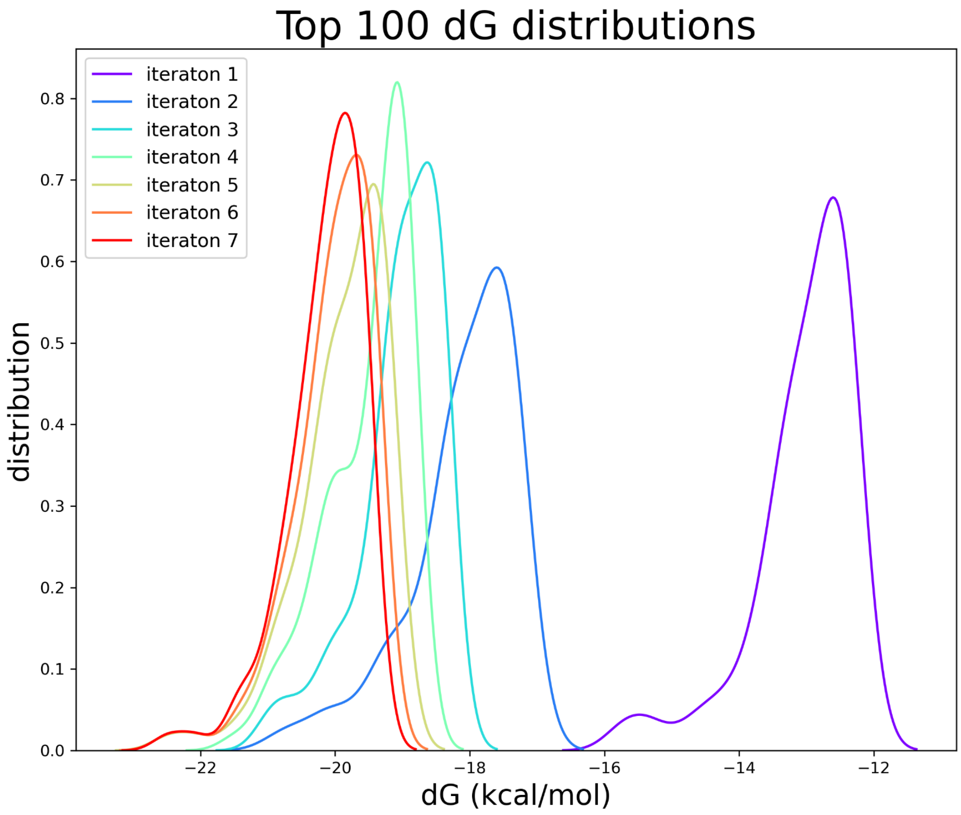

Supplementary Fig. 12 | Predicted binding affinities of Na_V1.7 VSD4 virtual screening. The distribution of the predicted binding affinities of the top 100 ranked molecules after each iteration of Na_V1.7 virtual screening. Source data are provided as a Source Data file.

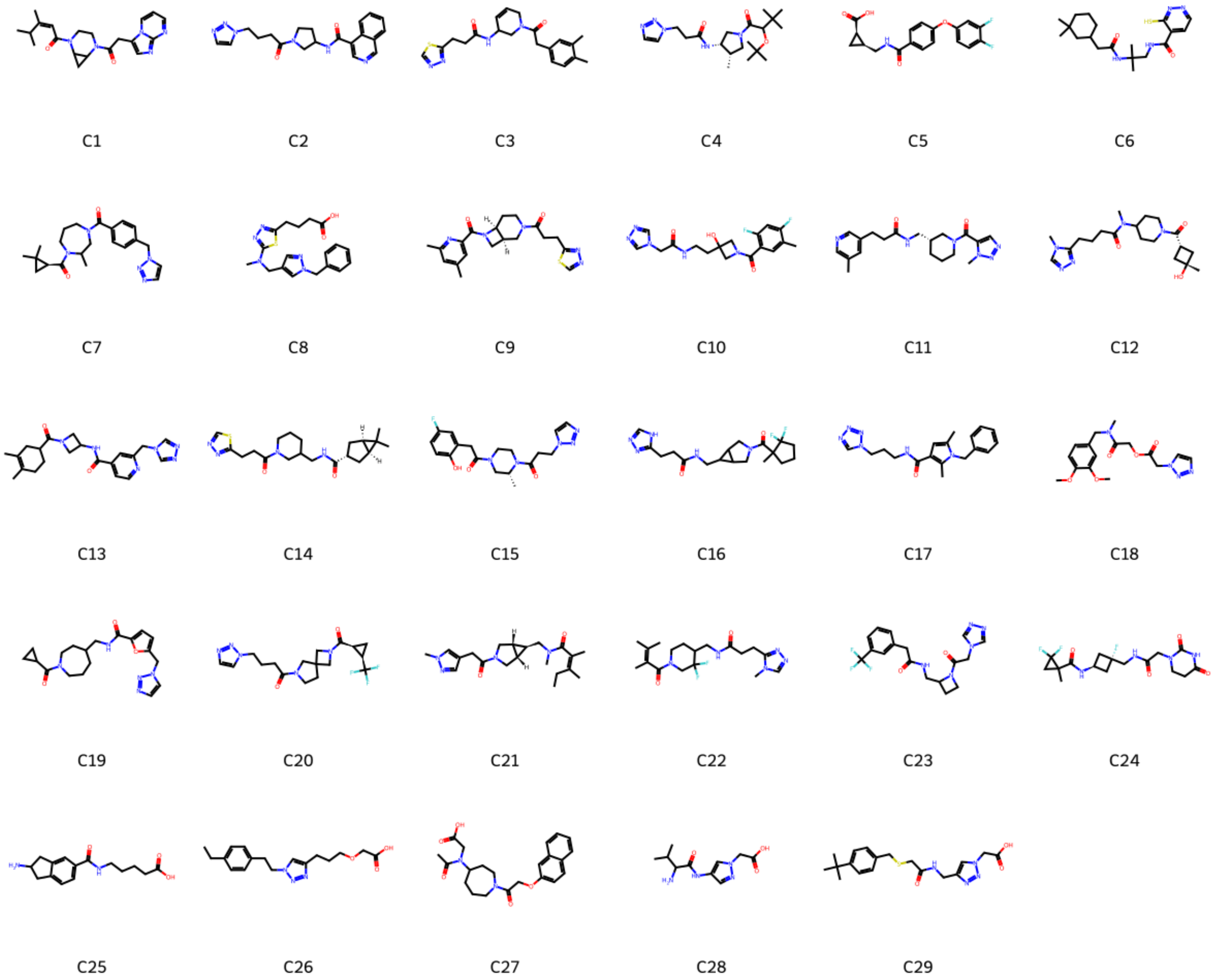

Supplementary Fig. 13 | Twenty-nine compounds from initial screening of KLHDC2. The 2D chemical structures of the experimentally tested twenty-nine compounds from the initial virtual screening of KLHDC2.

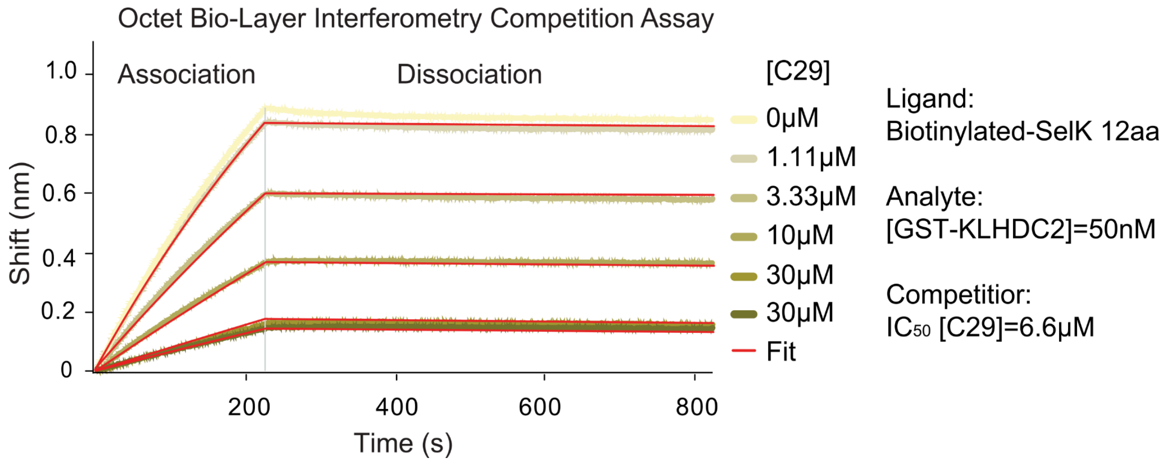

Supplementary Fig. 14 | BioLayer Interferometry competition assay of compound 29. Streptavidin coated optical probes were loaded with Biotin-SelK (12 aa). The binding of the analyte GST-KLHDC2 in the presence or absence of the C29, to the loaded optical probes was measured in the association buffer. The dissociation was measured while the probes were incubated in the Octet buffer. The association and dissociation curves were fitted with a Local Full fit, 1:1 ligand model.

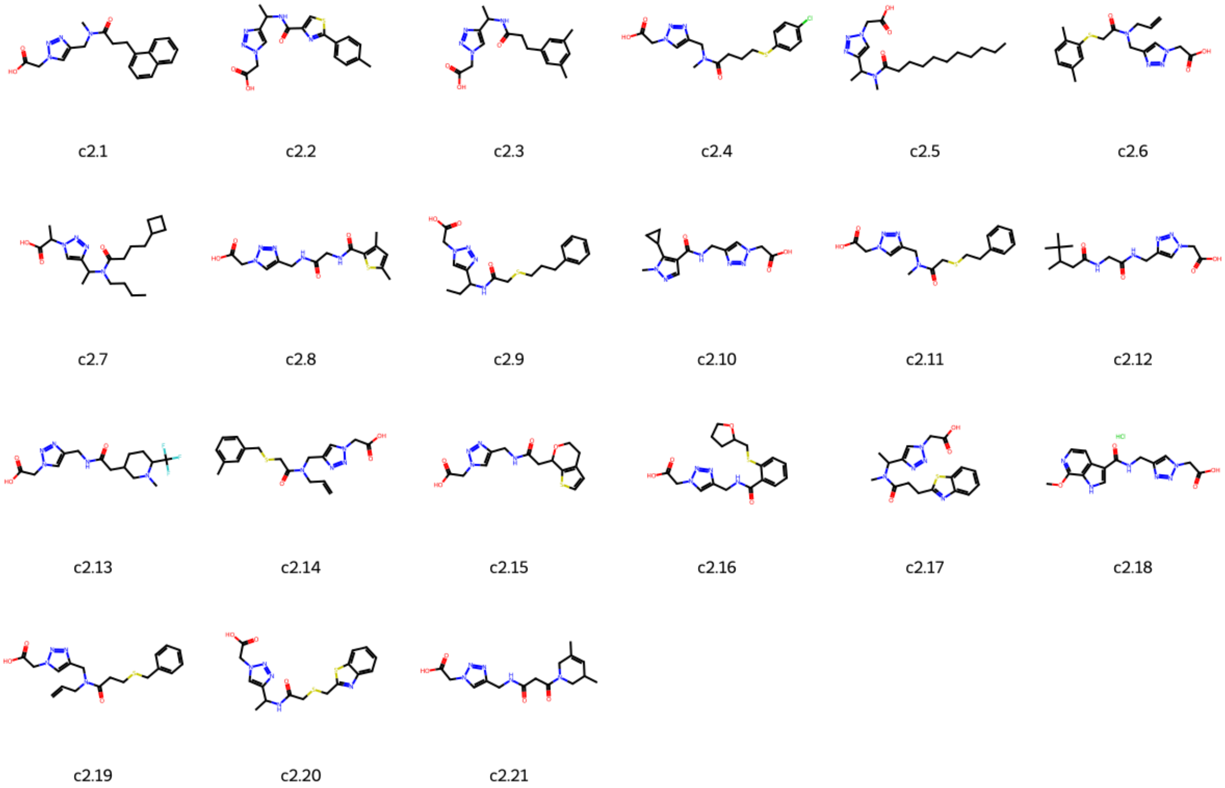

Supplementary Fig. 15 | Twenty-one compounds from the focused screening of KLHDC2. The 2D chemical structures of the experimentally tested twenty-one compounds from the focused library virtual screening of KLHDC2.

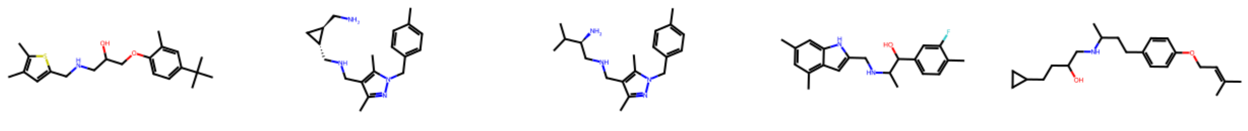

Z8739902234 Z3831472882 Z8739902231 Z8718594710 Z8739905023

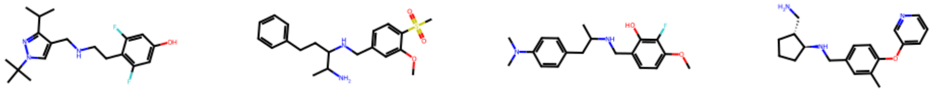

Z8718594704 Z4315497494 Z4415557415 Z8718594701

Supplementary Fig. 16 | Nine compounds from the virtual screening of Na$_V$1.7 VSD4. The 2D chemical structures of the experimentally tested nine compounds from the virtual screening of Na$_V$1.7 VSD4.

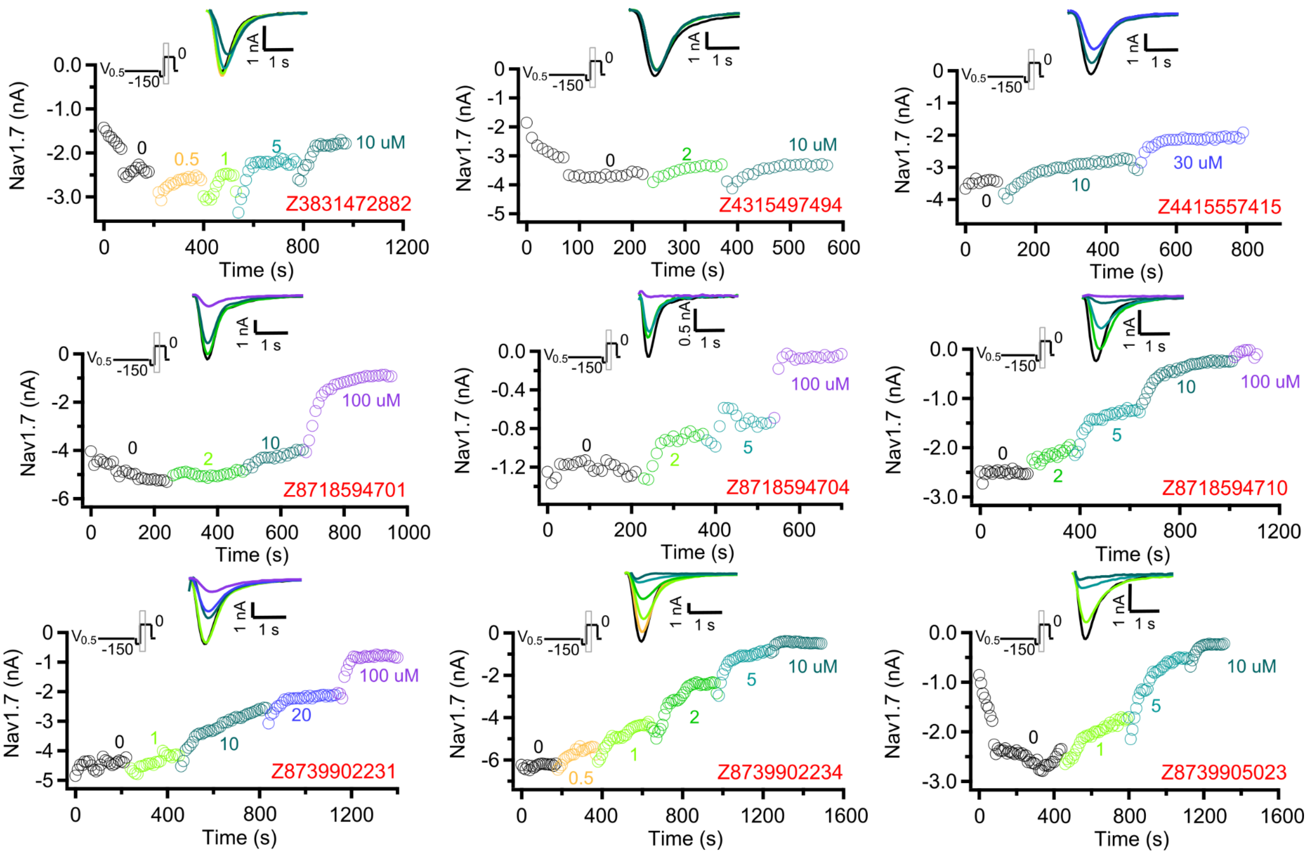

Supplementary Fig. 17 | Whole-cell patch clamp recordings for Na$_V$1.7 VSD4 compounds. The whole-cell patch clamp recording of the nine compounds from the virtual screening of Na$_V$1.7 VSD4. The compounds bind to the inactivated state of the channel.

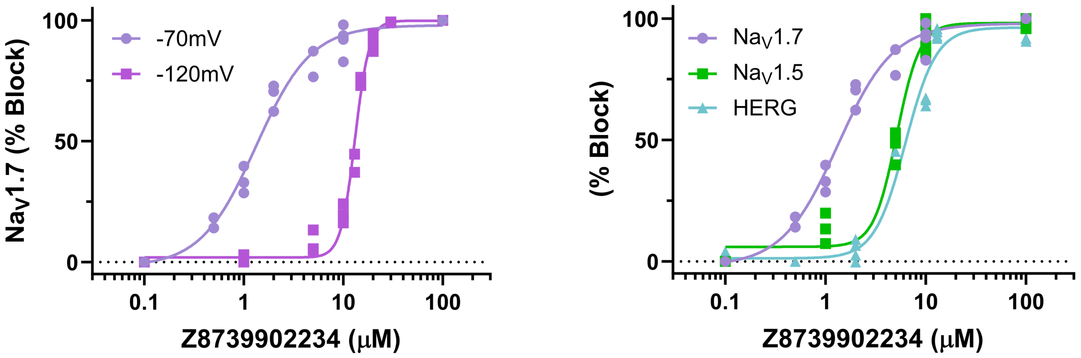

Supplementary Fig. 18 | Selectivity of inhibitory effect of Z8739902234 against inactivated state, resting state of Nav1.7, Nav1.5 and HERG. Left, Comparison of concentration–response relationship of Z8739902234 inhibition against Na_V1.7 in inactivated state (-70 mV holding potential) and resting state (-120 mV holding potential). IC_{50} (in μM, mean, 95% CI) for inactivated state: 1.33, 1.14 - 1.55; resting state: 13.11, 12.78 - 13.45. Right, Selectivity of inhibitory effect of Z8739902234 on Na_V1.7, Na_V1.5 and HERG. IC_{50} (in μM, mean, 95% CI). Na_V1.7 (-70 mV holding potential): 1.33, 1.14 - 1.55; Na_V1.5 (-80 mV holding potential): 5.14, 4.74 - 5.56; HERG: 6.27, 5.28–7.37. Source data are provided as a Source Data file.

Supplementary Table 1 | Data collection and refinement statistics (molecular replacement)

	KLHDC2-C29 (PDB ID: 8UXS [https://doi.org/10.2210/pdb8UXS/pdb])
**Data collection**	
Space group	P 1 21 1
Cell dimensions	
a, *b*, *c* (Å)	44.381 88.165 88.512
a, b, g (°)	90 104.395 90
Resolution (Å)	42.99 – 2.0 (2.071 – 2.0)
R_{merge}	0.05737 (0.1776)
I / s*I*	16.44 (3.20)
Completeness (%)	99.86 (99.48)
Redundancy	3.5 (3.6)
**Refinement**	
Resolution (Å)	44.08 – 2.00 (2.04 – 2.00)
No. reflections	85458 (5178)
R_{work} / R_{free}	0.17 / 0.20 (0.23 / 0.27)
No. atoms	
Protein	5184
Ligand/ion	98
Water	261
B-factors	
Protein	28.80
Ligand/ion	41.22
Water	29.78
R.m.s. deviations	
Bond lengths (Å)	0.002
Bond angles (°)	0.575

One crystal was used.
*Values in parentheses are for highest-resolution shell.

Supplementary Table 2 | Chemical properties of ordered compounds.

**Target**	**KLHDC2**			**Nav1.7**		
Property	QED[a]↑	cLogP[b]	SA[c]↓	QED[a]↑	cLogP[b]	SA[c]↓
min	0.43	-2.16	2.24	0.57	0.80	3.16
mean	0.69	0.83	3.35	0.76	2.40	3.81
max	0.87	2.61	4.88	0.85	3.47	4.35

The statistics of important chemical properties of ordered molecules are shown in this table. [a]QED, Quantitative Estimate of Drug-likeness, ranges from 0 (drug-unlike) to 1 (drug-like)[74]. [b]cLogP, the calculated octanol-water partition coefficient, assesses the hydrophobicity of organic compounds, higher value means more hydrophobic[75]. [c]SA, synthetic accessibility, quantifies the difficulty of chemical synthesis of organic compounds with values between 0 (easy to synthesize) to 10 (difficult to synthesize)[76]. Source data are provided as a Source Data file.
