# Supplementary material for: An artificial intelligence accelerated virtual screening platform for drug discovery
Source: Nat Commun. 2024 Sep 5;15:7761. doi: 10.1038/s41467-024-52061-7 (PMC11377542; doi:10.1038/s41467-024-52061-7)
Supplement: Supplementary file 3 — Description of Additional Supplementary Files [file 41467_2024_52061_MOESM3_ESM.docx]

**Description of Additional Supplementary Files**

File Name: Supplementary Data 1

Description: Comprehensive CASF2016 benchmarks.

File Name: Supplementary Data 2

Description: Purity of synthesized compounds

File Name: Supplementary Data 3

Description: Liquid chromatography– mass spectrometry of the synthesized compounds.
