# Supplementary material for: An artificial intelligence accelerated virtual screening platform for drug discovery
Source: Nat Commun. 2024 Sep 5;15:7761. doi: 10.1038/s41467-024-52061-7 (PMC11377542; doi:10.1038/s41467-024-52061-7)
Supplement: Supplementary file 6 — Supplementary Data 3 [file 41467_2024_52061_MOESM6_ESM.zip › LC-MS-spectra/KLHDC2/Z8381047289.PDF]

BA888172$1

MaxPeak: 100.00%
Ret_Time: 0.910 min

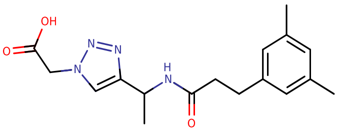

Mol Wt 330.38

Exact Mass 330.19

#	Time	Area%
1	0.910	100.00

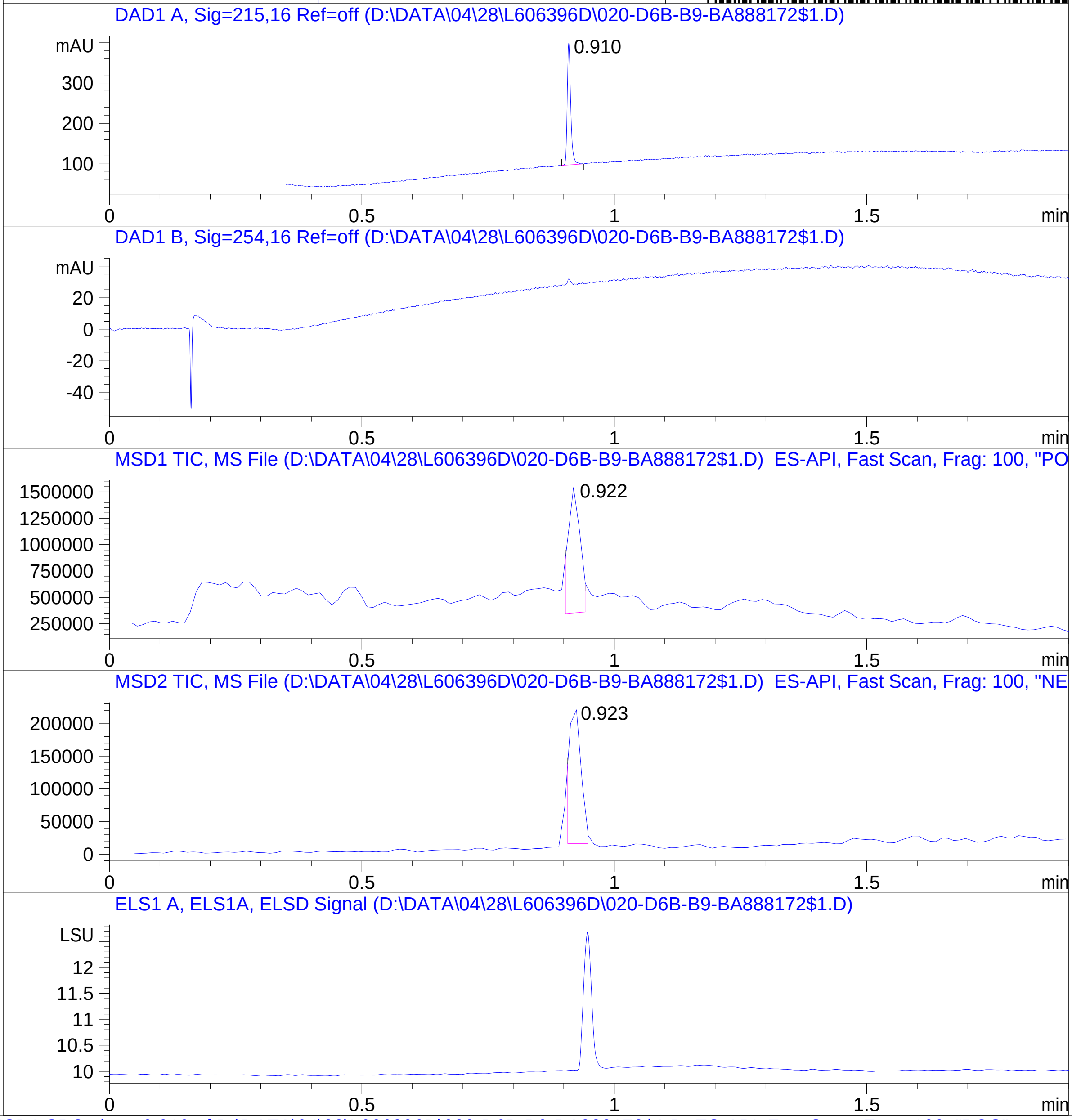

RT 0.922

RT 0.923

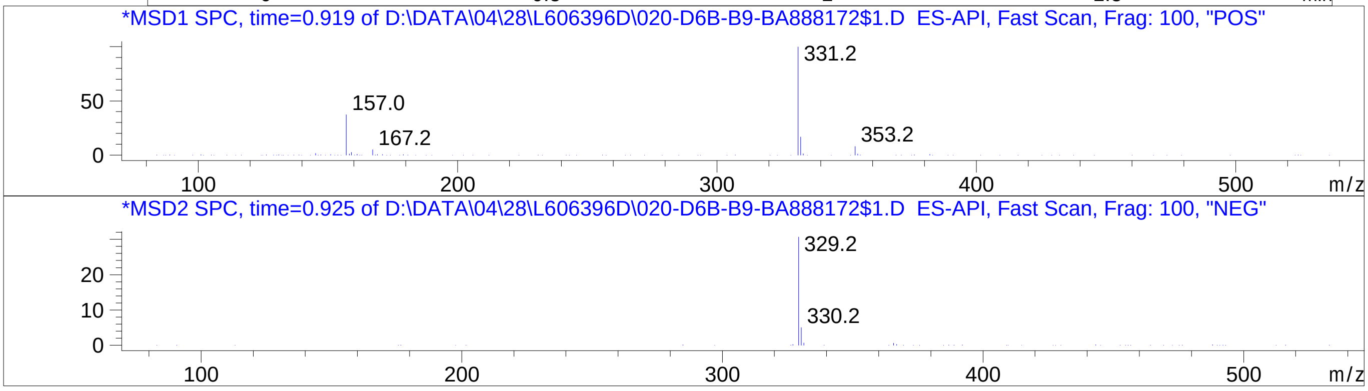

Inj.Date 4/27/2023 CH <invalid> 22 Acq. Method C:\Users\ -> ->
