# Supplementary material for: An artificial intelligence accelerated virtual screening platform for drug discovery
Source: Nat Commun. 2024 Sep 5;15:7761. doi: 10.1038/s41467-024-52061-7 (PMC11377542; doi:10.1038/s41467-024-52061-7)
Supplement: Supplementary file 6 — Supplementary Data 3 [file 41467_2024_52061_MOESM6_ESM.zip › LC-MS-spectra/KLHDC2/Z8381047276.PDF]

BA888182$1

MaxPeak: 95.37%
Ret_Time: 1.215 min

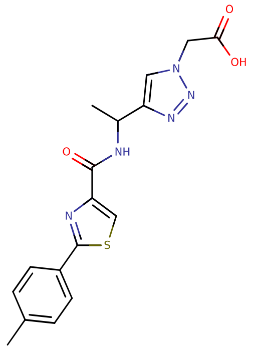

Mol Wt 371.41

Exact Mass 371.11

#	Time	Area%
1	1.215	95.37
2	1.566	4.63

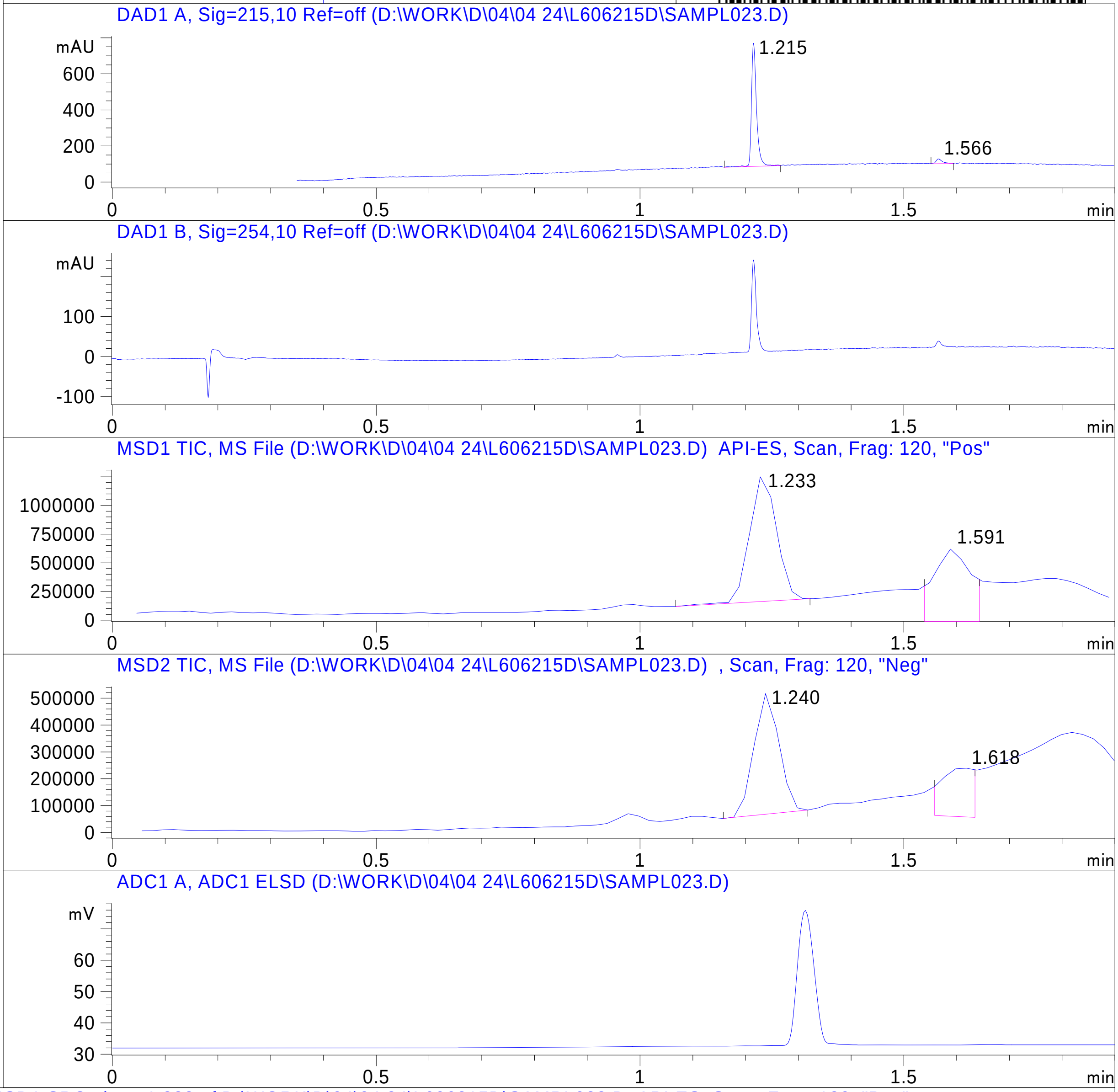

RT 1.233

RT 1.591

RT 1.240

RT 1.618

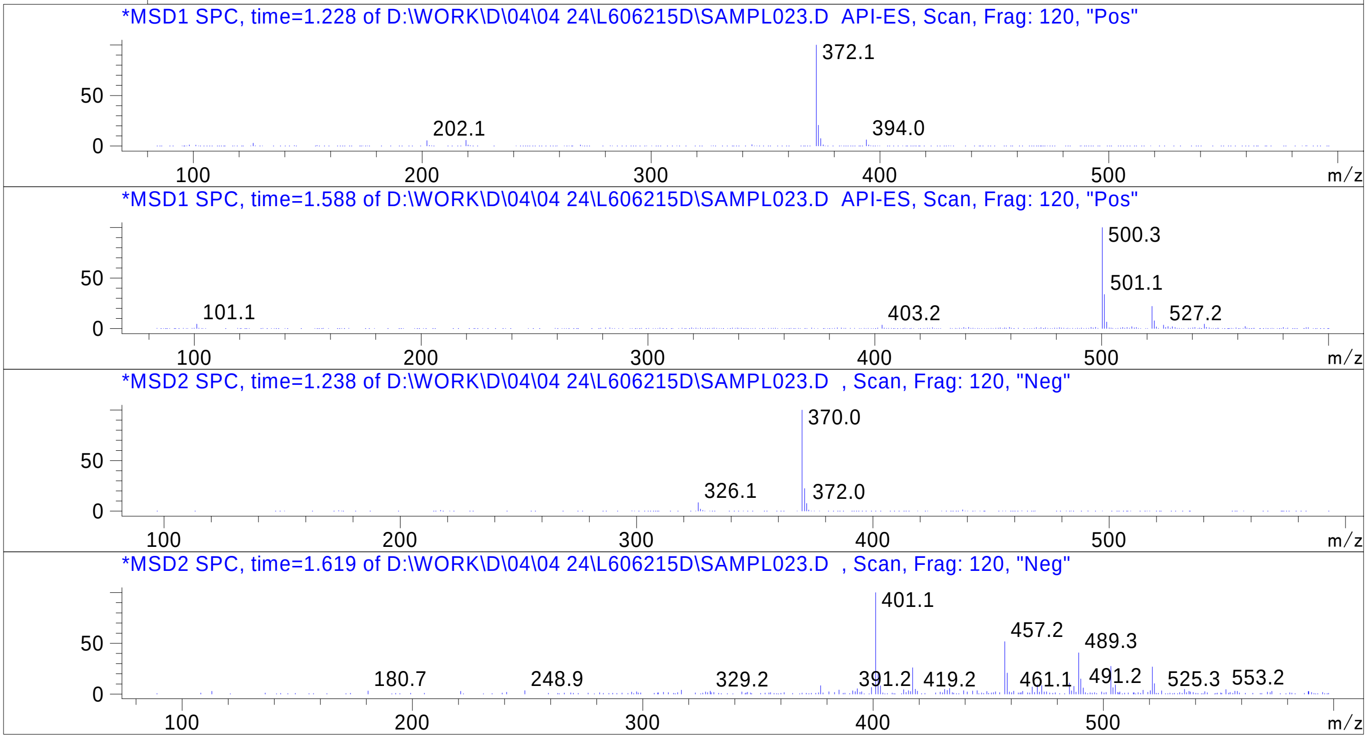

Inj.Date 4/28/2023 K P2-C-05 -SL- Acq. Method C:\HPCHEM\-> ->
