# Supplementary material for: An artificial intelligence accelerated virtual screening platform for drug discovery
Source: Nat Commun. 2024 Sep 5;15:7761. doi: 10.1038/s41467-024-52061-7 (PMC11377542; doi:10.1038/s41467-024-52061-7)
Supplement: Supplementary file 6 — Supplementary Data 3 [file 41467_2024_52061_MOESM6_ESM.zip › LC-MS-spectra/KLHDC2/Z8381047060.PDF]

MaxPeak: 93.07%
Ret_Time: 0.645 min

BB323362$112

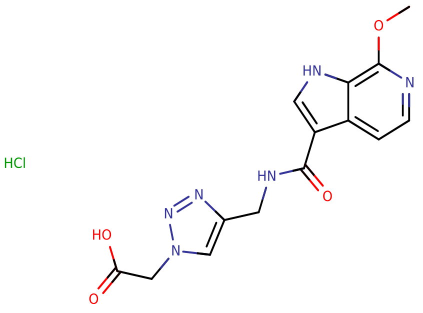

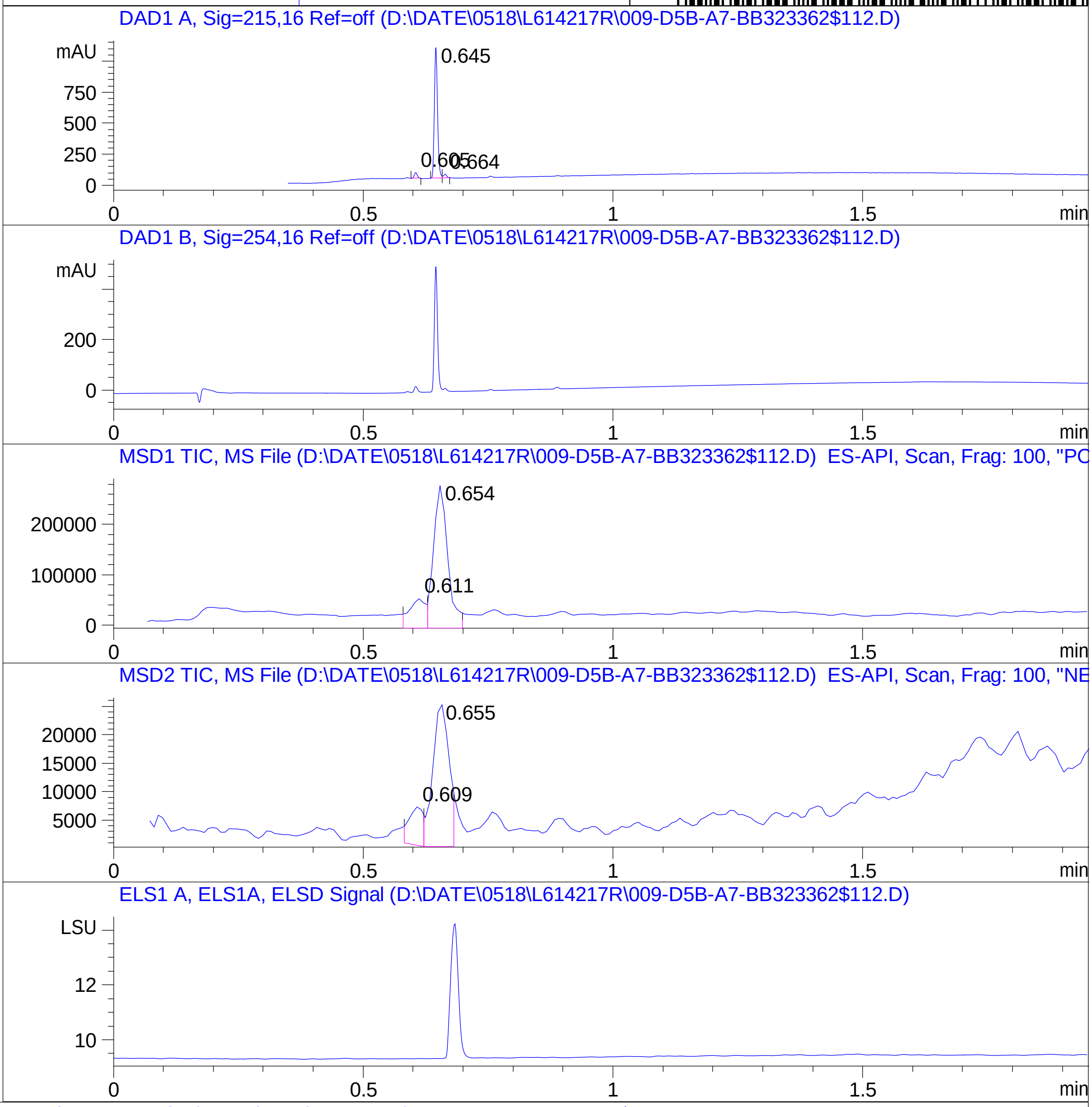

Mol Wt 366.76

Exact Mass 330.1

#	Time	Area%
1	0.605	4.14
2	0.645	93.07
3	0.664	2.79

RT 0.611

RT 0.654

RT 0.609

RT 0.655

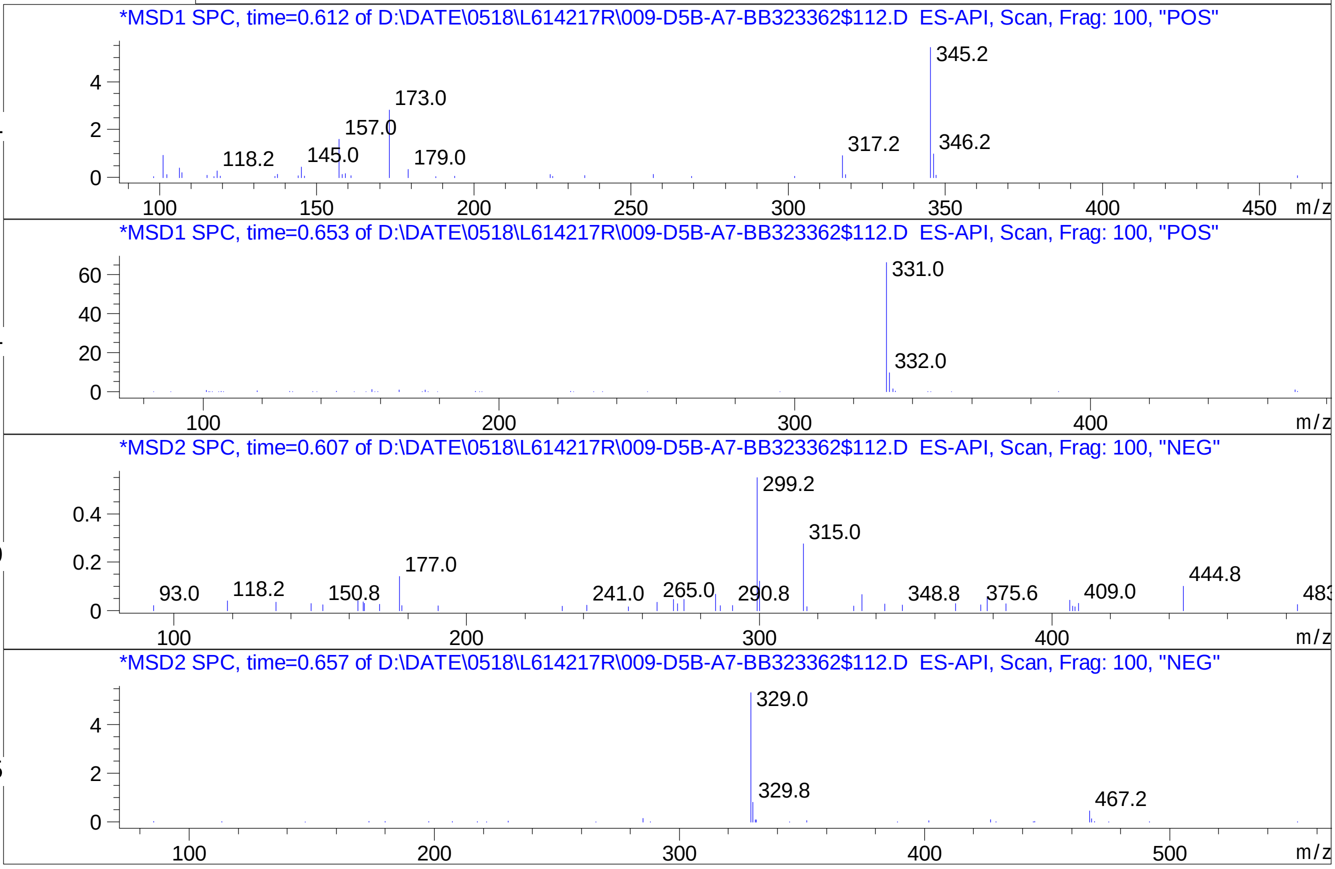

Inj.Date 5/18/2023 CH -17- Acq. Method C:\Chem32\-> ->
