# Supplementary material for: An artificial intelligence accelerated virtual screening platform for drug discovery
Source: Nat Commun. 2024 Sep 5;15:7761. doi: 10.1038/s41467-024-52061-7 (PMC11377542; doi:10.1038/s41467-024-52061-7)
Supplement: Supplementary file 6 — Supplementary Data 3 [file 41467_2024_52061_MOESM6_ESM.zip › LC-MS-spectra/KLHDC2/Z7881785655.PDF]

MaxPeak: 97.10%
Ret_Time: 1.389 min

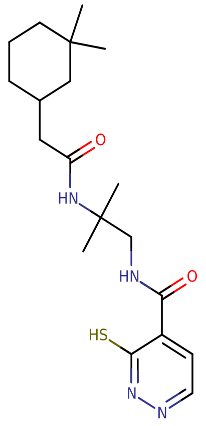

BA005648$3

Mol Wt 378.53

Exact Mass 378.25

#	Time	Area%
1	1.344	2.90
2	1.389	97.10

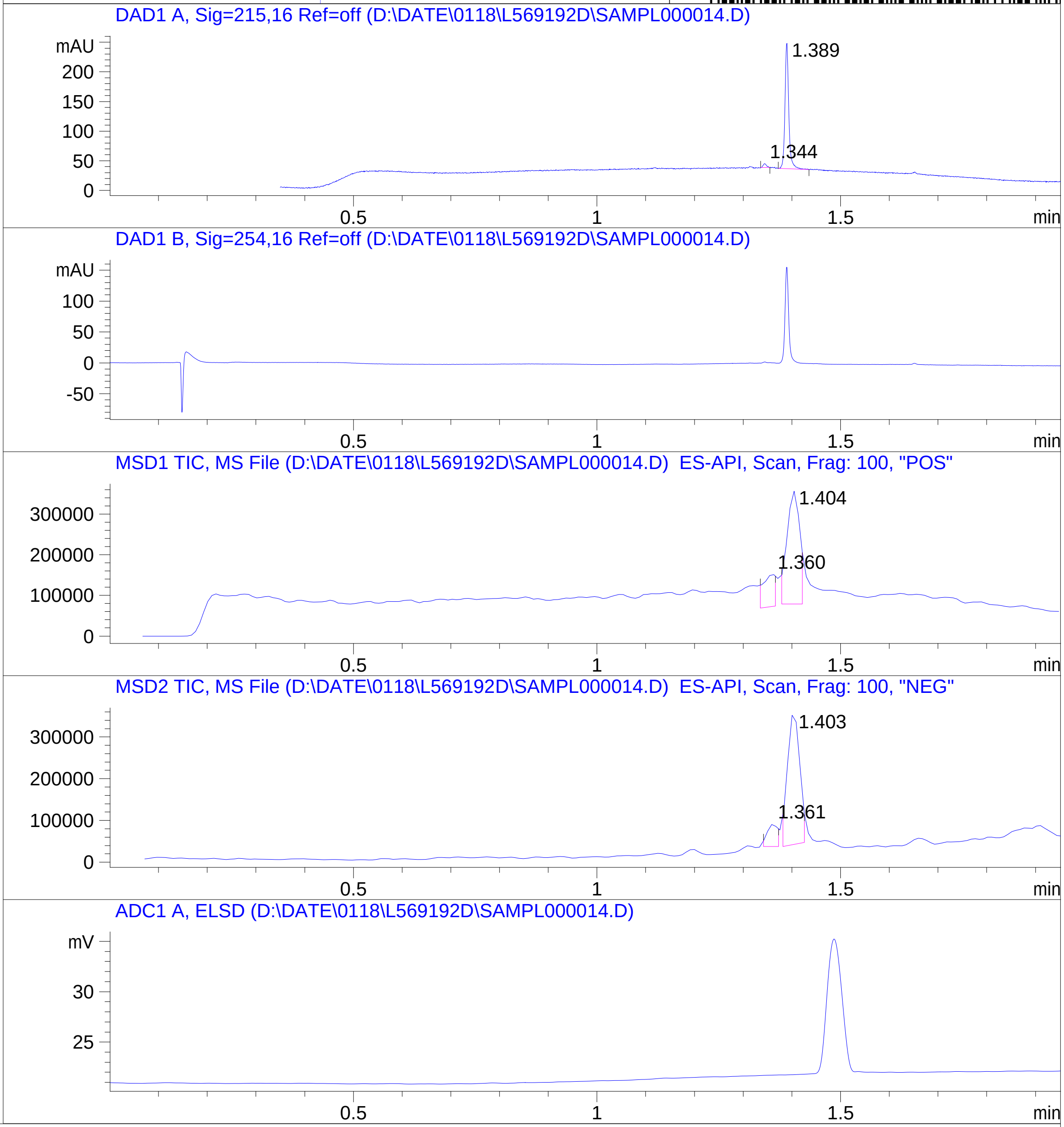

RT 1.360

RT 1.404

RT 1.361

RT 1.403

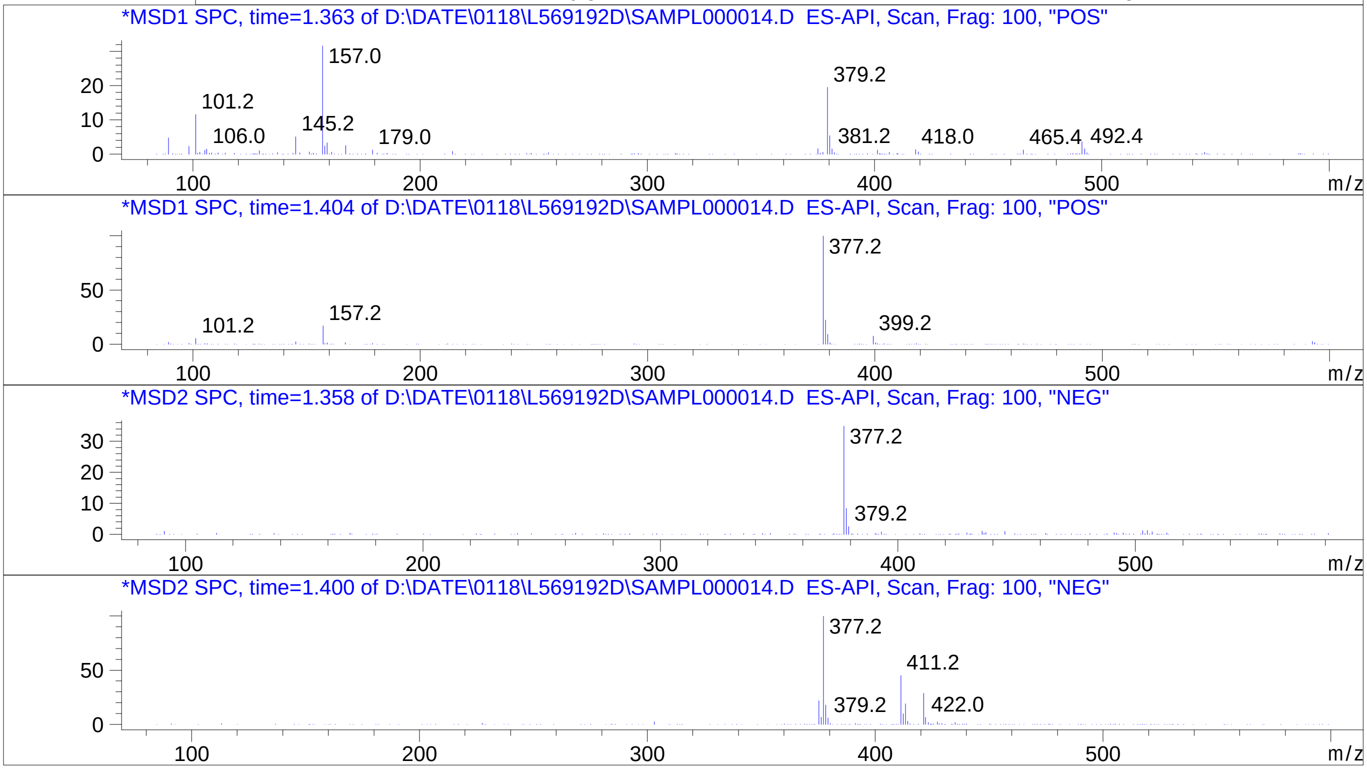

Inj.Date 1/18/2023   E   Acq. Method C:\CHEM32\->   ->
