# Supplementary material for: An artificial intelligence accelerated virtual screening platform for drug discovery
Source: Nat Commun. 2024 Sep 5;15:7761. doi: 10.1038/s41467-024-52061-7 (PMC11377542; doi:10.1038/s41467-024-52061-7)

BA005638$3

MaxPeak: 100.00%
Ret_Time: 1.218 min

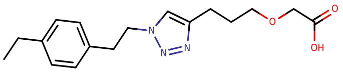

Mol Wt 317.38

Exact Mass 317.2

#	Time	Area%
1	1.218	100.00

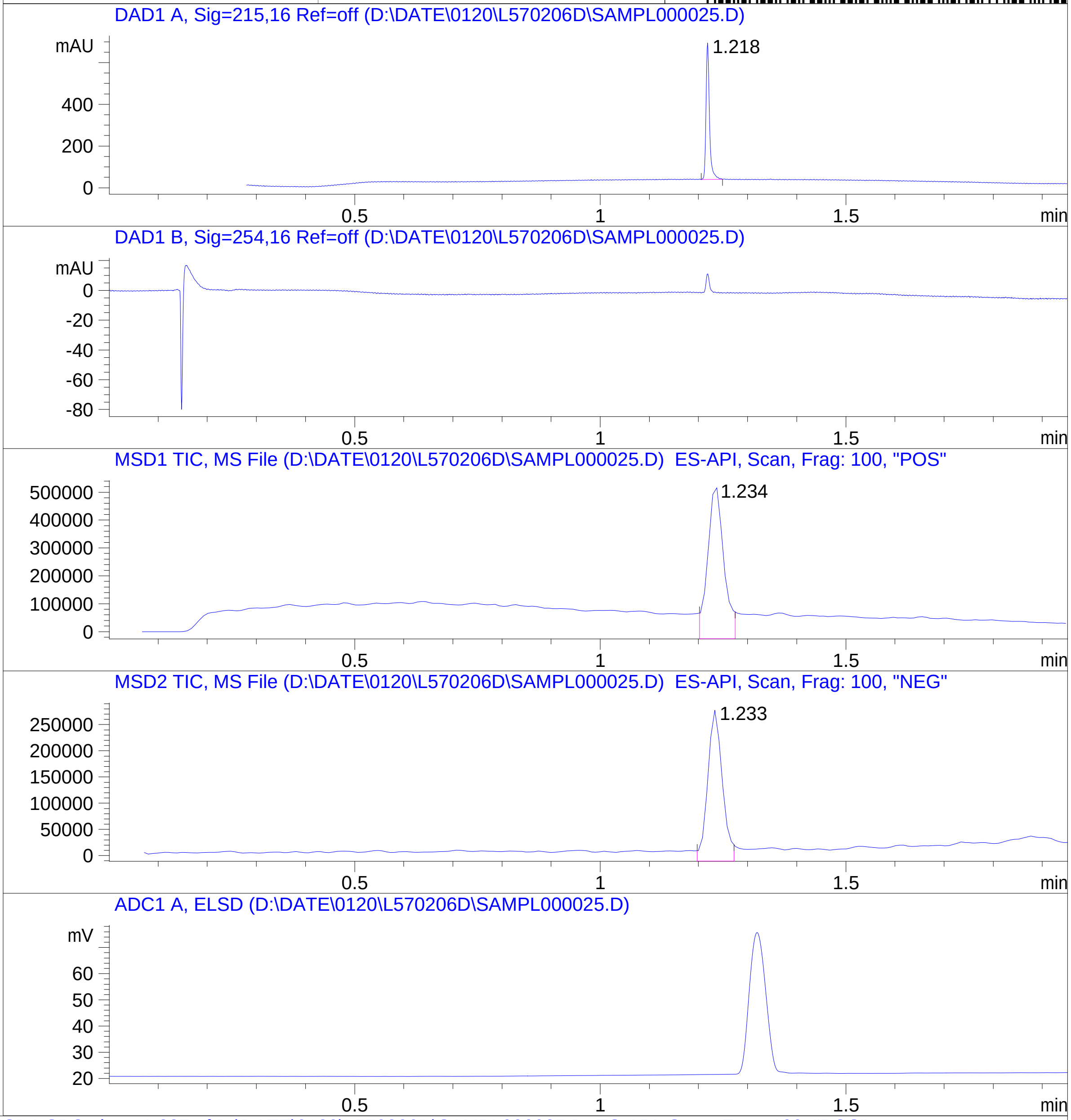

RT 1.234

RT 1.233

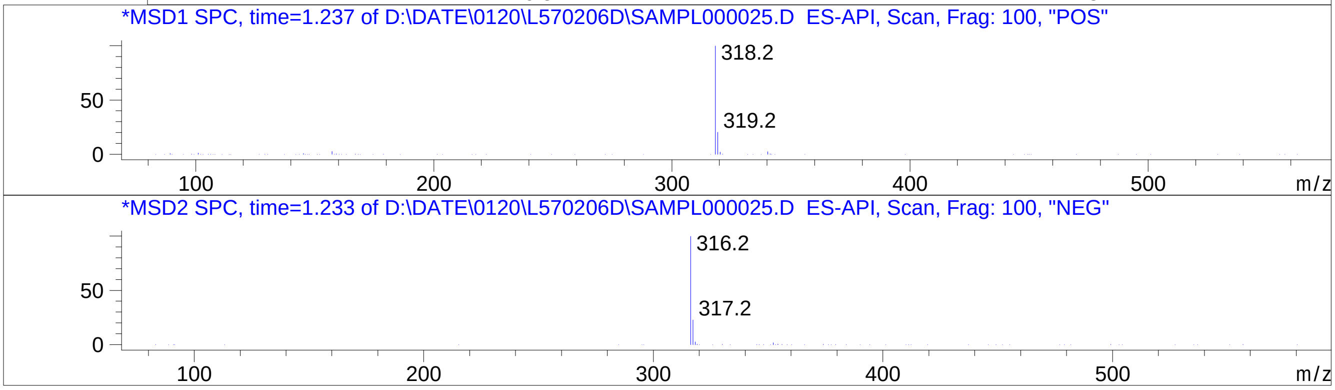

Supplement: Supplementary file 6 — Supplementary Data 3 [file 41467_2024_52061_MOESM6_ESM.zip › LC-MS-spectra/KLHDC2/Z4079348547.PDF]
