# Supplementary material for: An artificial intelligence accelerated virtual screening platform for drug discovery
Source: Nat Commun. 2024 Sep 5;15:7761. doi: 10.1038/s41467-024-52061-7 (PMC11377542; doi:10.1038/s41467-024-52061-7)
Supplement: Supplementary file 6 — Supplementary Data 3 [file 41467_2024_52061_MOESM6_ESM.zip › LC-MS-spectra/KLHDC2/Z7881785708.PDF]

MaxPeak: 98.37%
Ret_Time: 1.088 min

BA005616$3

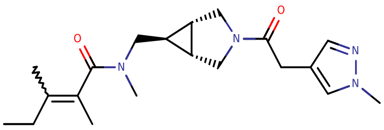

Mol Wt 358.48

Exact Mass 358.28

#	Time	Area%
1	1.015	1.63
2	1.088	98.37

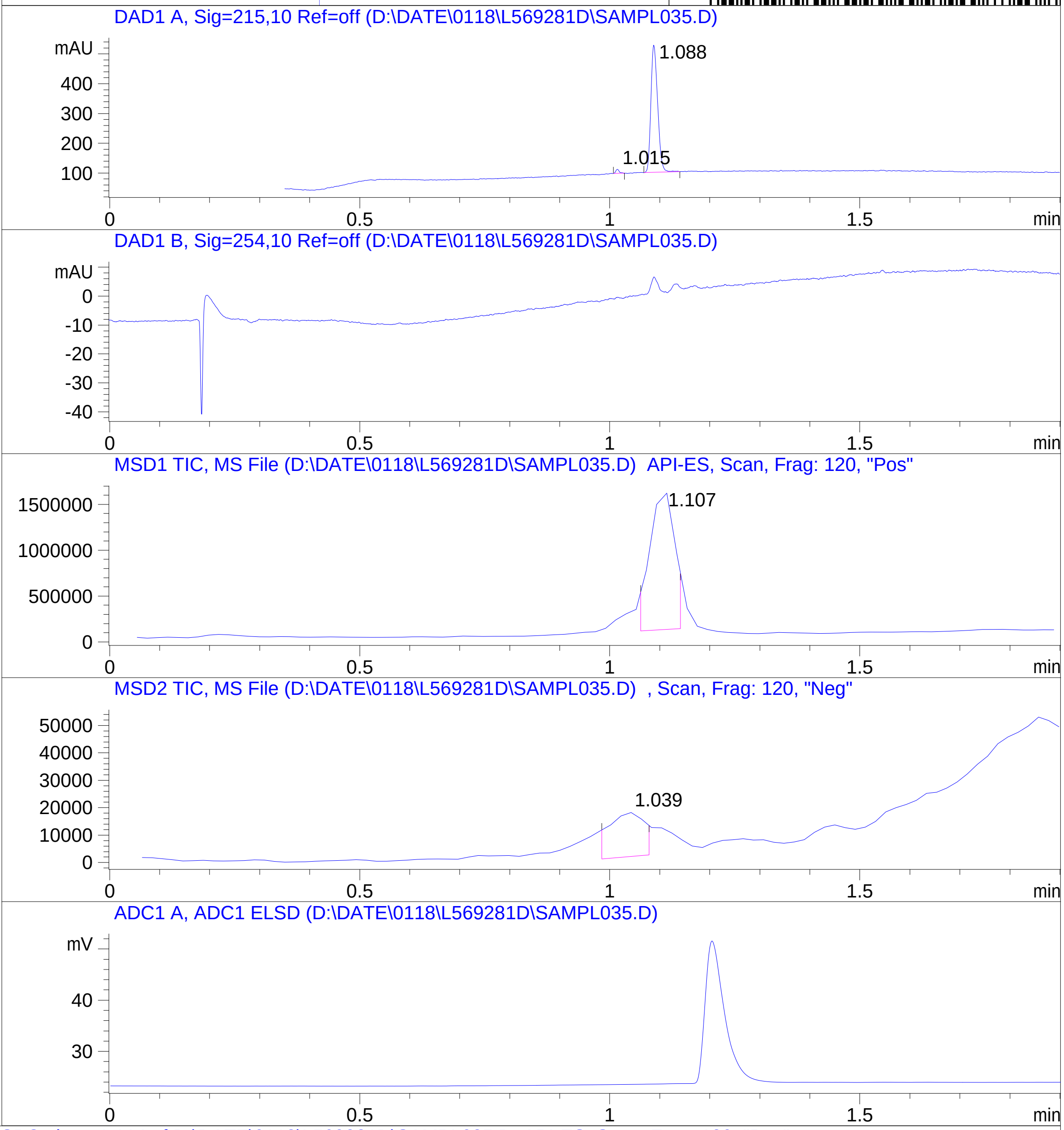

RT 1.107

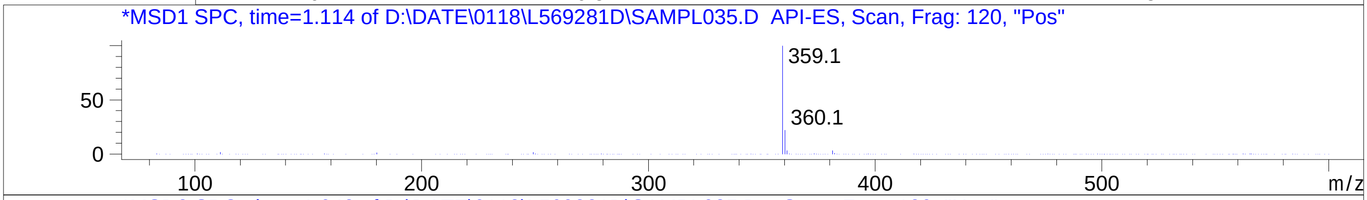

RT 1.039

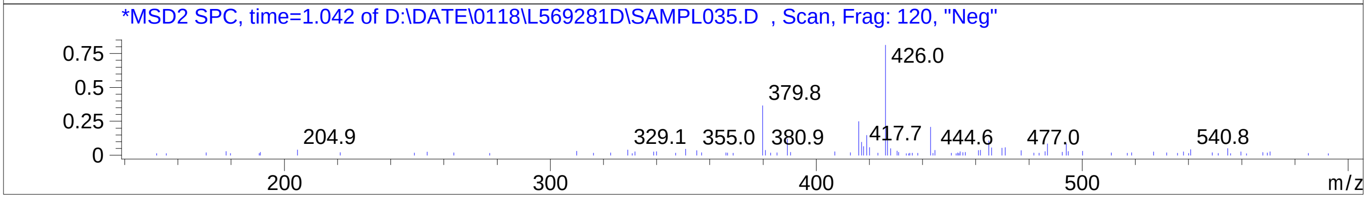

Inj.Date 1/19/2023 E -VL- Acq. Method C:\HPCHEM\-> ->
