# Supplementary material for: An artificial intelligence accelerated virtual screening platform for drug discovery
Source: Nat Commun. 2024 Sep 5;15:7761. doi: 10.1038/s41467-024-52061-7 (PMC11377542; doi:10.1038/s41467-024-52061-7)

MaxPeak: 100.00%
Ret_Time: 1.113 min

BA005623$1

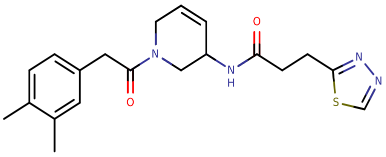

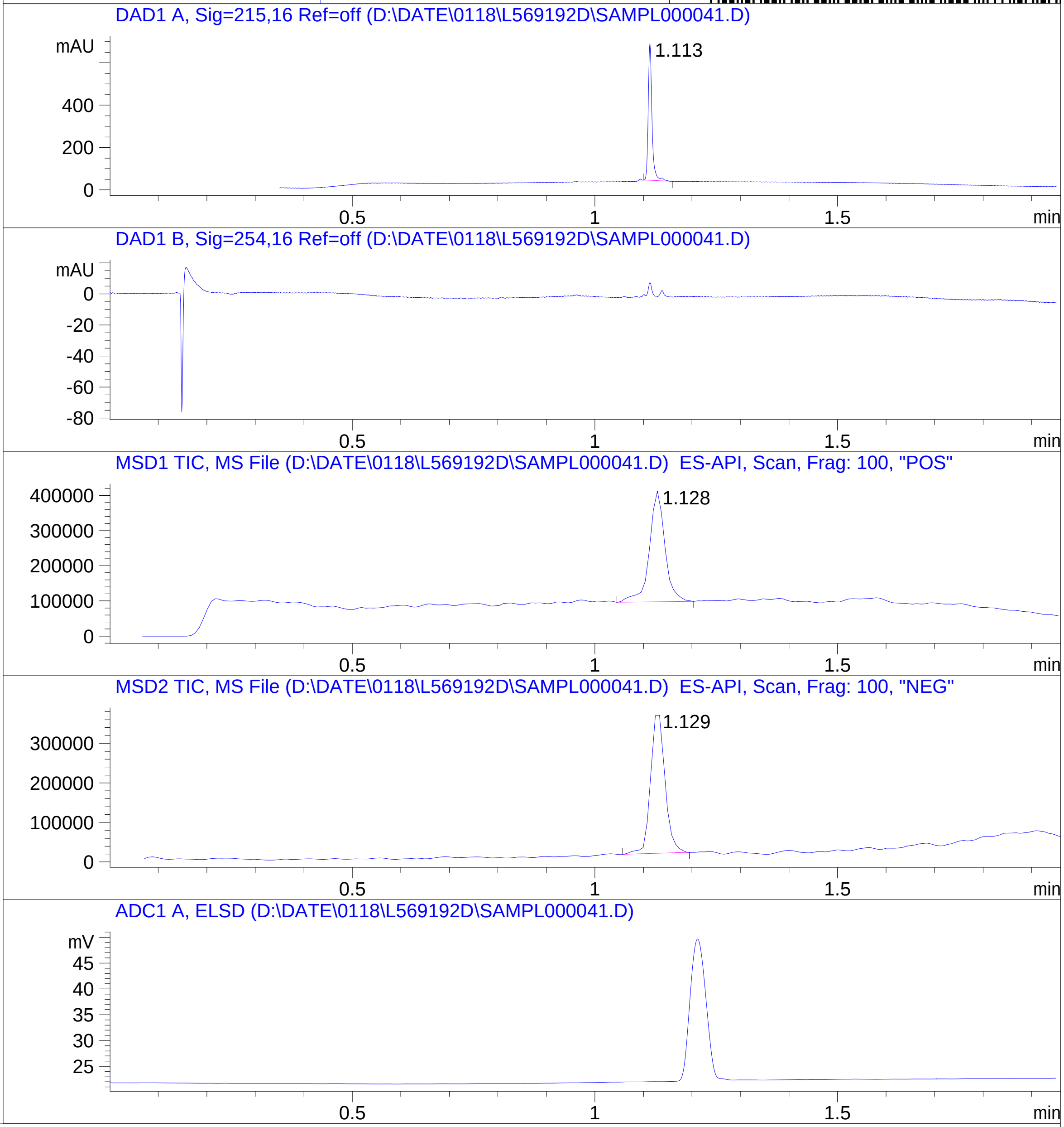

Mol Wt 384.5

Exact Mass 384.19

```
#  Time    Area%
-----------------
1  1.113   100.00
```

RT 1.128

RT 1.129

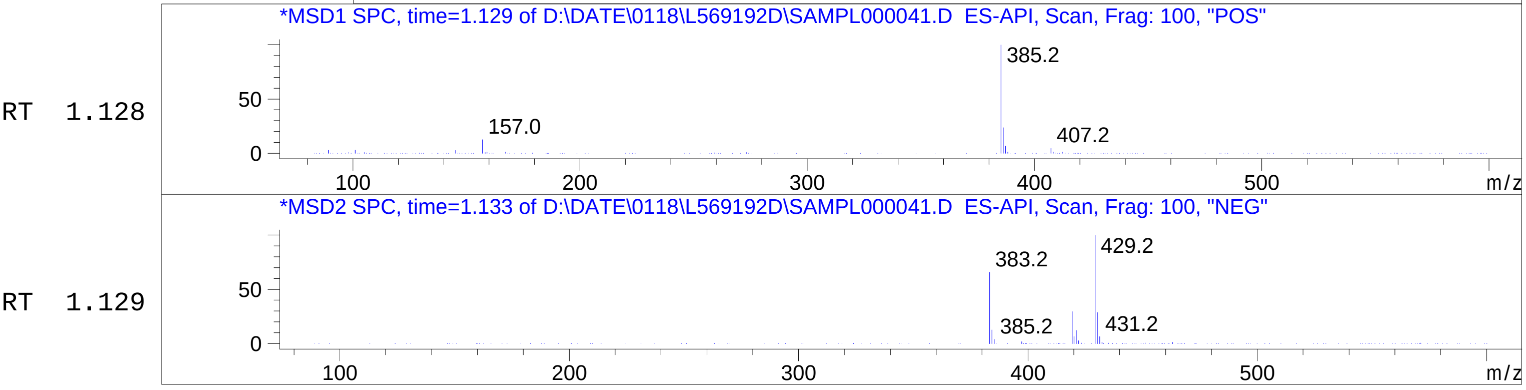

Supplement: Supplementary file 6 — Supplementary Data 3 [file 41467_2024_52061_MOESM6_ESM.zip › LC-MS-spectra/KLHDC2/Z7881785697.PDF]
