# Supplementary material for: An artificial intelligence accelerated virtual screening platform for drug discovery
Source: Nat Commun. 2024 Sep 5;15:7761. doi: 10.1038/s41467-024-52061-7 (PMC11377542; doi:10.1038/s41467-024-52061-7)
Supplement: Supplementary file 6 — Supplementary Data 3 [file 41467_2024_52061_MOESM6_ESM.zip › LC-MS-spectra/KLHDC2/Z7881785863.PDF]

MaxPeak: 95.73%
Ret_Time: 0.974 min

BA005624$3

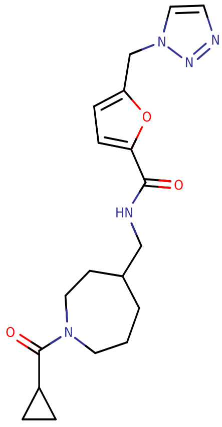

Mol Wt 371.43

Exact Mass 371.22

#	Time	Area%
1	0.974	95.73
2	1.451	4.27

DAD1 A, Sig=215,10 Ref=off (D:\DATE\0117\L568997D\SAMPL005.D)

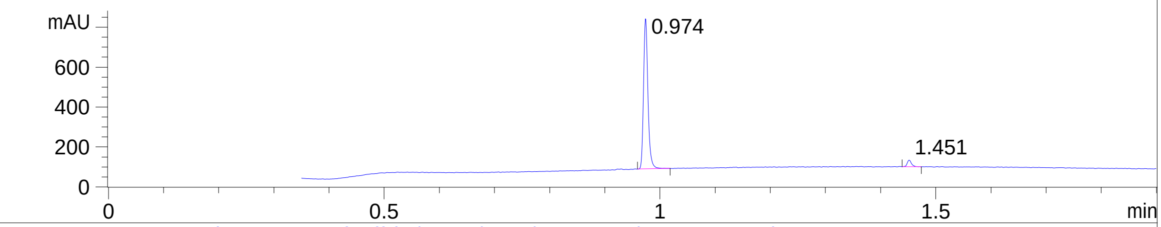

DAD1 B, Sig=254,10 Ref=off (D:\DATE\0117\L568997D\SAMPL005.D)

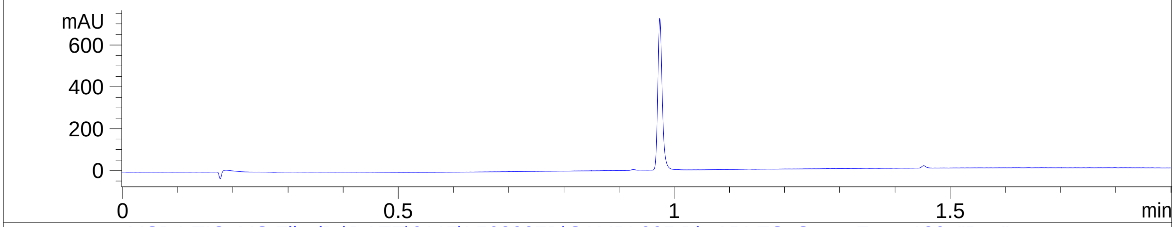

MSD1 TIC, MS File (D:\DATE\0117\L568997D\SAMPL005.D) API-ES, Scan, Frag: 120, "Pos"

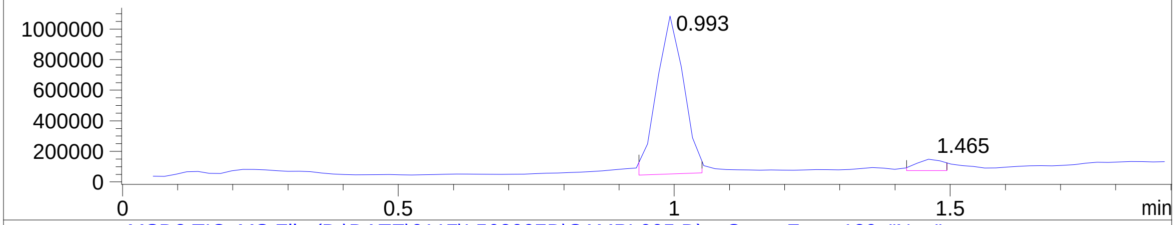

MSD2 TIC, MS File (D:\DATE\0117\L568997D\SAMPL005.D) , Scan, Frag: 120, "Neg"

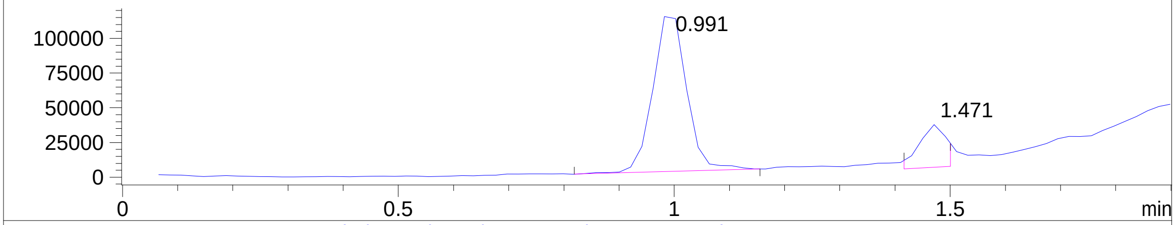

ADC1 A, ADC1 ELSD (D:\DATE\0117\L568997D\SAMPL005.D)

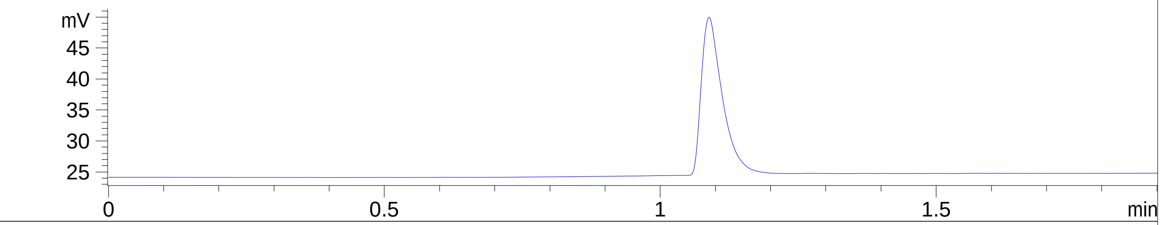

*MSD1 SPC, time=0.992 of D:\DATE\0117\L568997D\SAMPL005.D API-ES, Scan, Frag: 120, "Pos"

RT 0.993

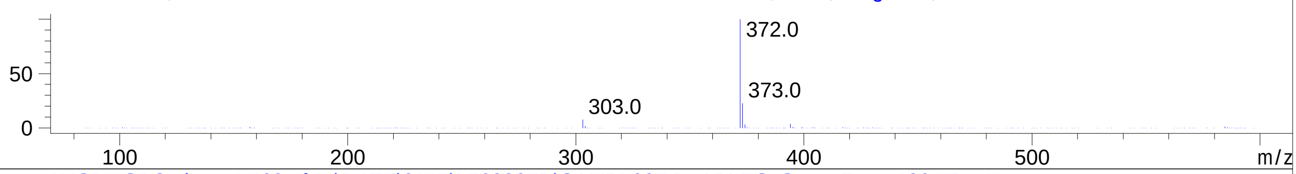

*MSD1 SPC, time=1.460 of D:\DATE\0117\L568997D\SAMPL005.D API-ES, Scan, Frag: 120, "Pos"

RT 1.465

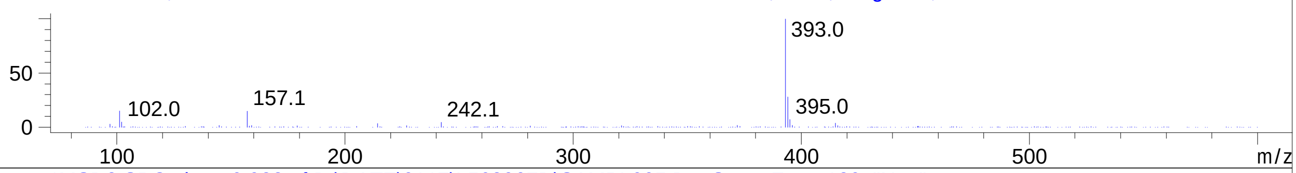

*MSD2 SPC, time=0.982 of D:\DATE\0117\L568997D\SAMPL005.D , Scan, Frag: 120, "Neg"

RT 0.991

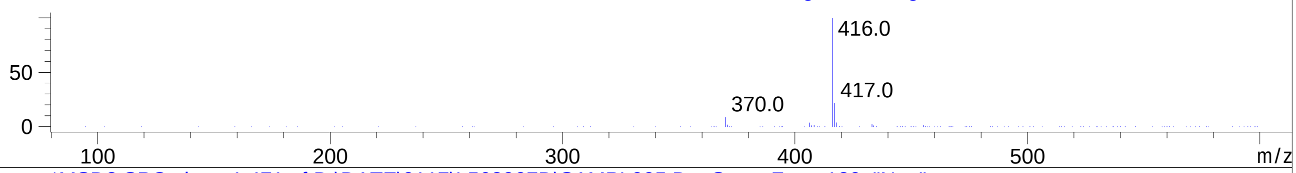

*MSD2 SPC, time=1.471 of D:\DATE\0117\L568997D\SAMPL005.D , Scan, Frag: 120, "Neg"

RT 1.471

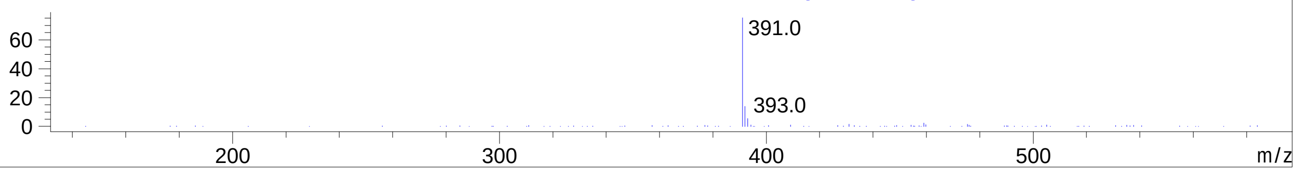

Inj.Date 1/18/2023   E   -VL-   Acq. Method C:\HPCHEM\->   ->
