# Supplementary material for: An artificial intelligence accelerated virtual screening platform for drug discovery
Source: Nat Commun. 2024 Sep 5;15:7761. doi: 10.1038/s41467-024-52061-7 (PMC11377542; doi:10.1038/s41467-024-52061-7)
Supplement: Supplementary file 6 — Supplementary Data 3 [file 41467_2024_52061_MOESM6_ESM.zip › LC-MS-spectra/KLHDC2/Z7881785877.PDF]

MaxPeak: 100.00%
Ret_Time: 0.855 min

BA005625$2

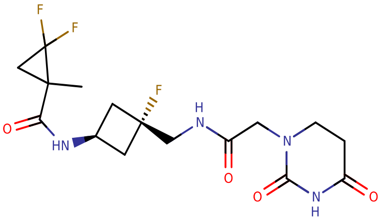

Mol Wt 390.36

Exact Mass 390.17

#	Time	Area%
1	0.855	100.00

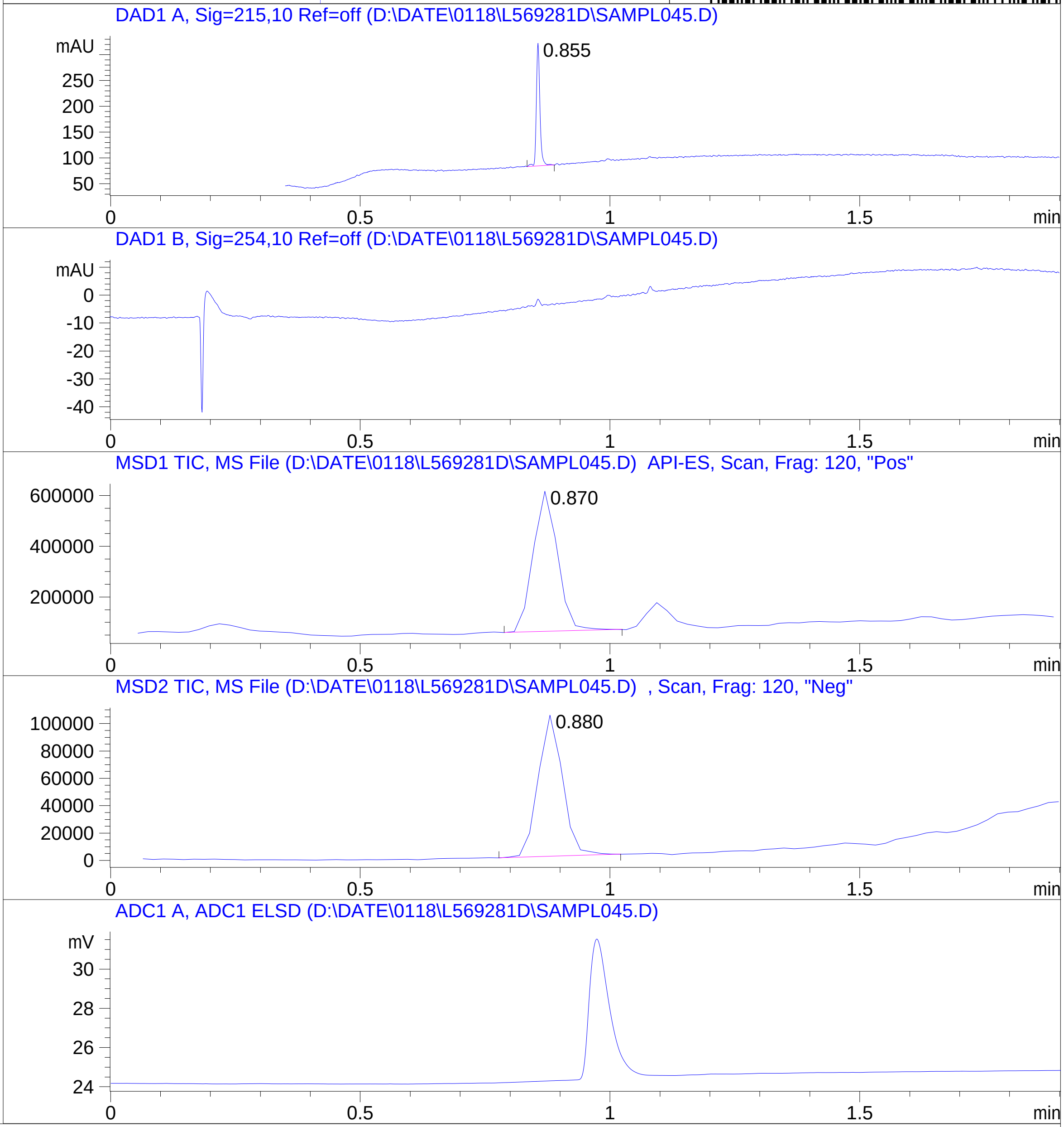

RT 0.870

RT 0.880

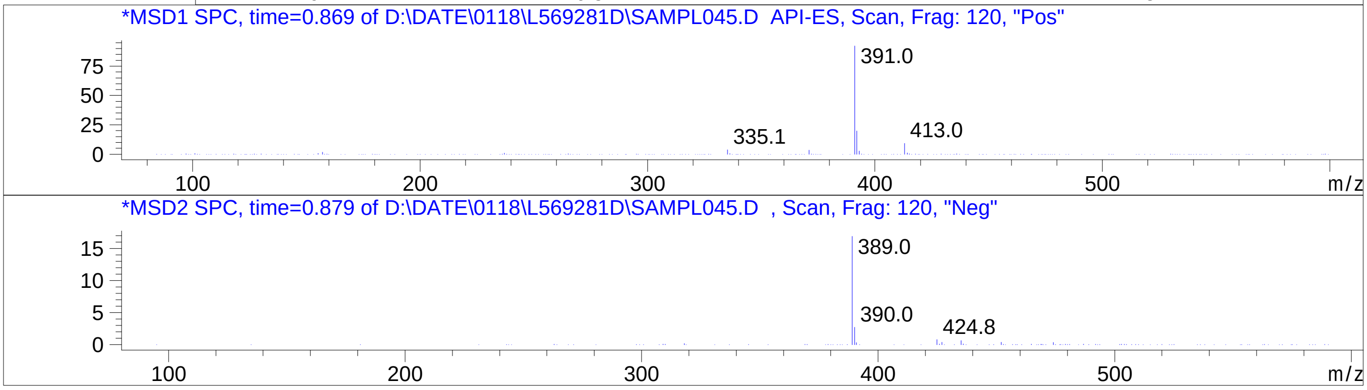

Inj.Date 1/19/2023 E -VL- Acq. Method C:\HPCHEM\-> ->
