# Supplementary material for: An artificial intelligence accelerated virtual screening platform for drug discovery
Source: Nat Commun. 2024 Sep 5;15:7761. doi: 10.1038/s41467-024-52061-7 (PMC11377542; doi:10.1038/s41467-024-52061-7)
Supplement: Supplementary file 6 — Supplementary Data 3 [file 41467_2024_52061_MOESM6_ESM.zip › LC-MS-spectra/KLHDC2/Z8381047264.PDF]

MaxPeak: 96.50%
Ret_Time: 0.886 min

BA888193$3

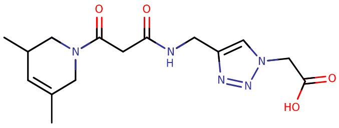

Mol Wt 335.36

Exact Mass 335.17

#	Time	Area%
1	0.886	96.50
2	0.922	1.64
3	0.991	1.86

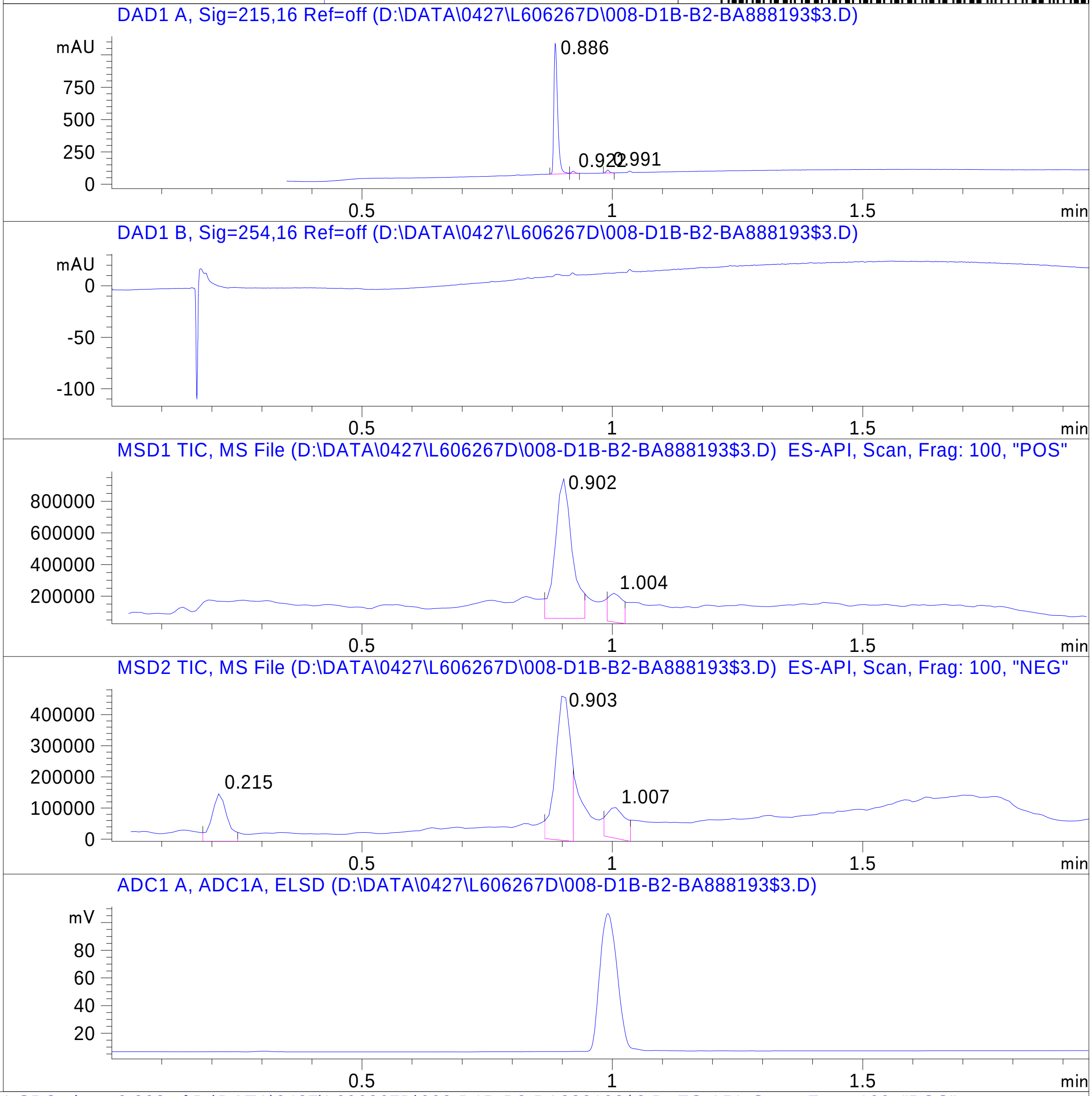

RT 0.902

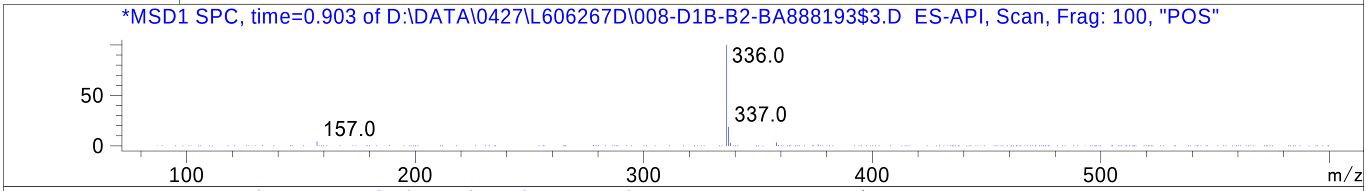

RT 1.004

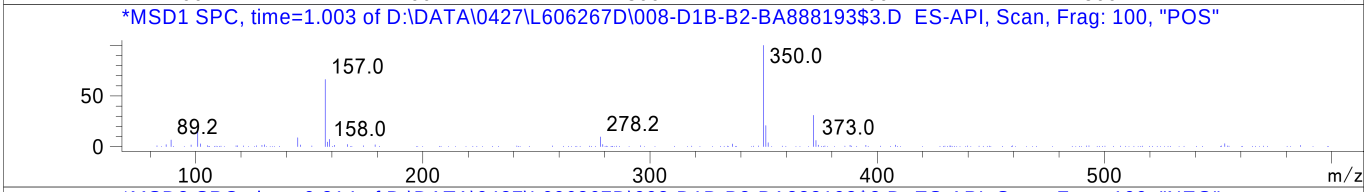

RT 0.215

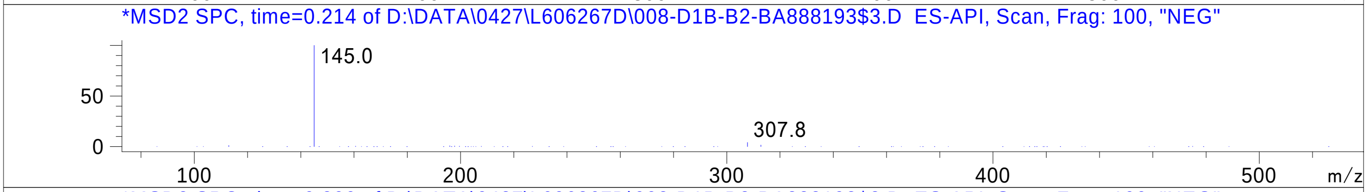

RT 0.903

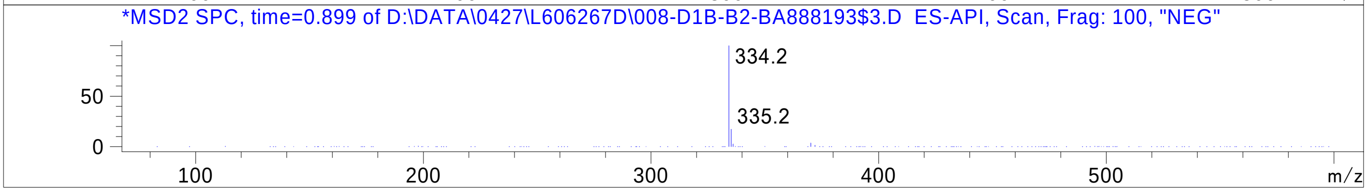

RT 1.007

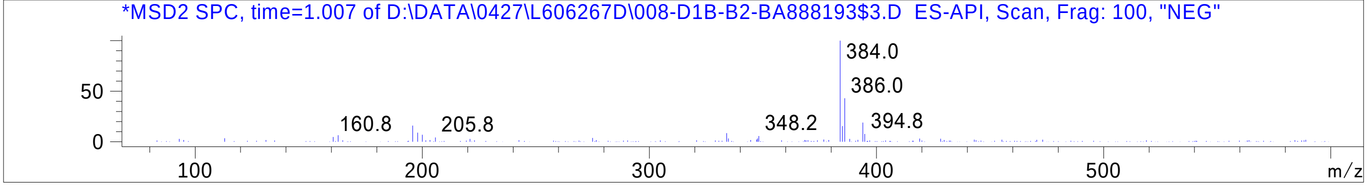
*MSD2 SPC, time=1.007 of D:\DATA\0427\L606267D\008-D1B-B2-BA888193$3.D ES-API, Scan, Frag: 100, "NEG"
384.0
386.0
394.8
348.2
160.8
205.8
50
0
100
200
300
400
500
m/z
