# Supplementary material for: An artificial intelligence accelerated virtual screening platform for drug discovery
Source: Nat Commun. 2024 Sep 5;15:7761. doi: 10.1038/s41467-024-52061-7 (PMC11377542; doi:10.1038/s41467-024-52061-7)
Supplement: Supplementary file 6 — Supplementary Data 3 [file 41467_2024_52061_MOESM6_ESM.zip › LC-MS-spectra/KLHDC2/Z8381047073.PDF]

# BA888156$2

MaxPeak: 100.00%
Ret_Time: 0.940 min

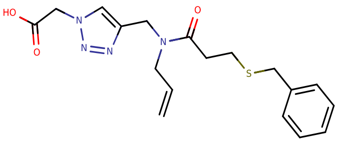


**Mol Wt** 374.46

**Exact Mass** 374.16

| # | Time | Area% |
|---|---|---|
| 1 | 0.940 | 100.00 |

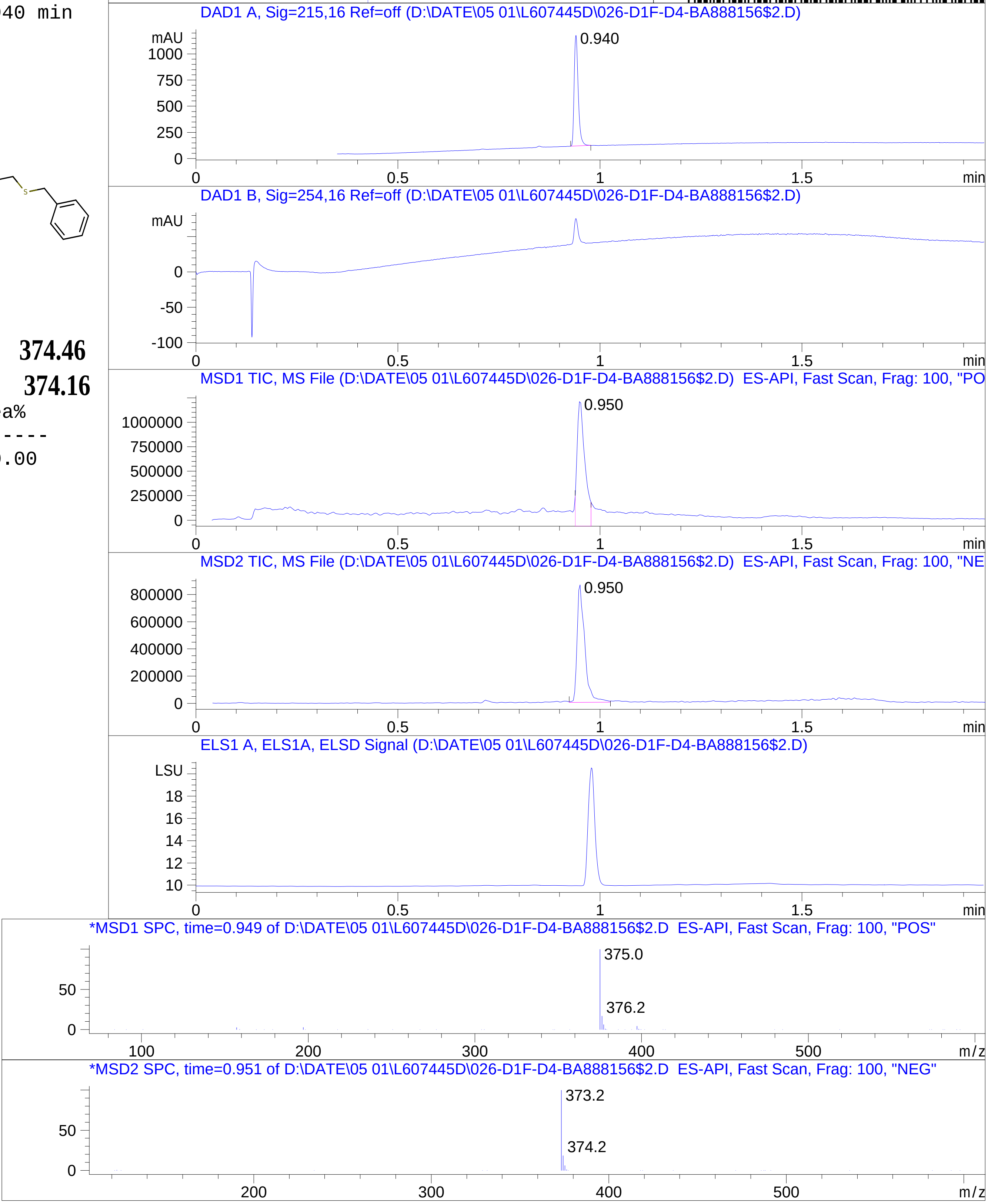


RT 0.950

RT 0.950

Inj.Date 5/1/2023 E Acq. Method C:\Users\ -> ->
