# Supplementary material for: An artificial intelligence accelerated virtual screening platform for drug discovery
Source: Nat Commun. 2024 Sep 5;15:7761. doi: 10.1038/s41467-024-52061-7 (PMC11377542; doi:10.1038/s41467-024-52061-7)
Supplement: Supplementary file 6 — Supplementary Data 3 [file 41467_2024_52061_MOESM6_ESM.zip › LC-MS-spectra/KLHDC2/Z3587812408.PDF]

BA005612$2

MaxPeak: 100.00%
Ret_Time: 1.026 min

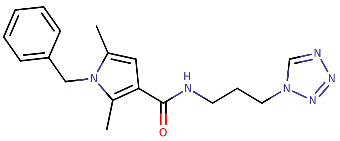

Mol Wt 338.41

Exact Mass 338.21

#	Time	Area%
1	1.026	100.00

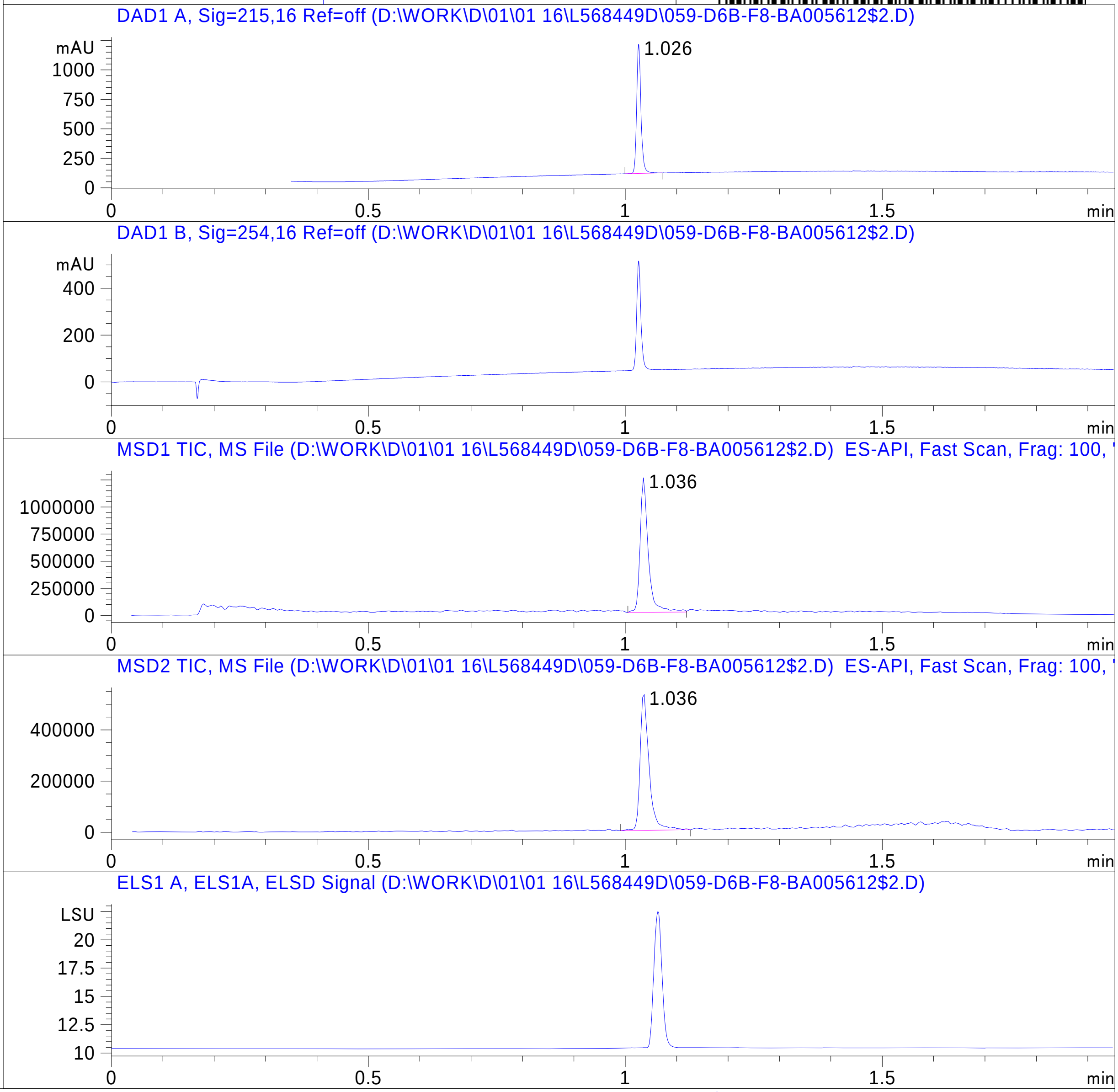

RT 1.036

RT 1.036

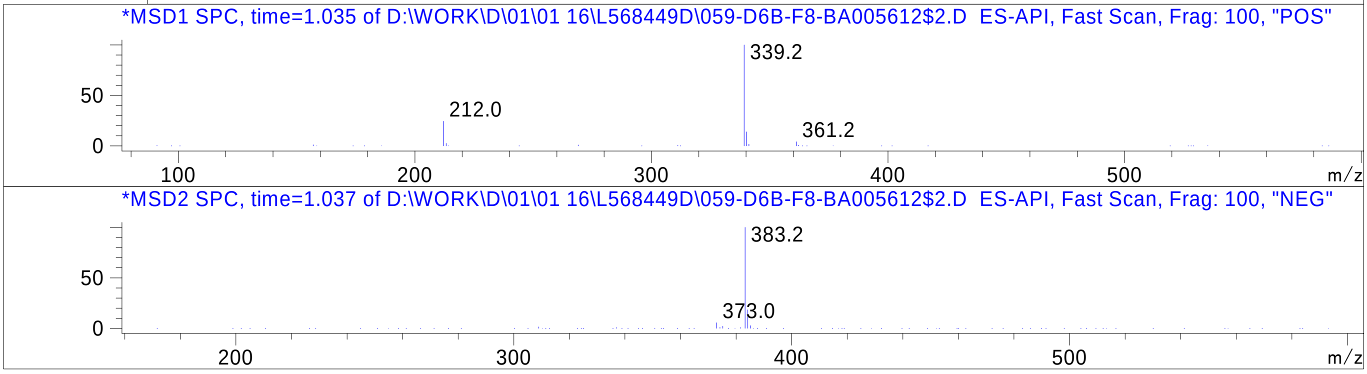

Inj.Date 1/16/2023 K <invalid> Acq. Method C:\Users\ -> ->
