# Supplementary material for: An artificial intelligence accelerated virtual screening platform for drug discovery
Source: Nat Commun. 2024 Sep 5;15:7761. doi: 10.1038/s41467-024-52061-7 (PMC11377542; doi:10.1038/s41467-024-52061-7)
Supplement: Supplementary file 6 — Supplementary Data 3 [file 41467_2024_52061_MOESM6_ESM.zip › LC-MS-spectra/KLHDC2/Z7881785646.PDF]

MaxPeak: 97.00%
Ret_Time: 0.749 min

# BA005615$4

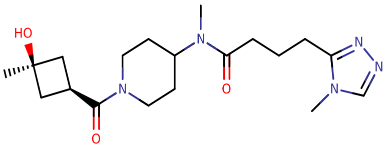


**Mol Wt** 377.48

**Exact Mass** 377.28

| # | Time | Area% |
|---|---|---|
| 1 | 0.749 | 97.00 |
| 2 | 0.850 | 3.00 |

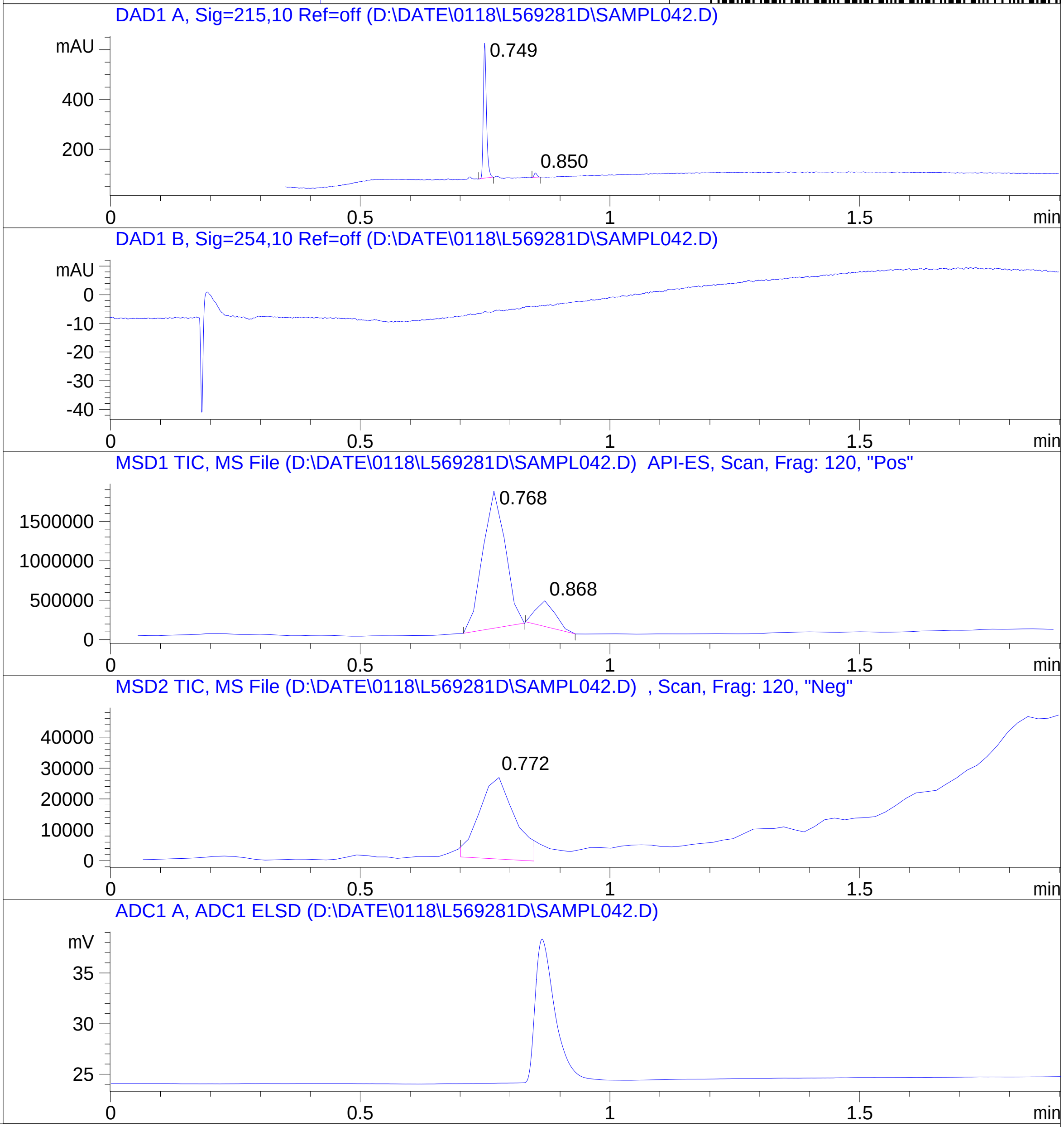


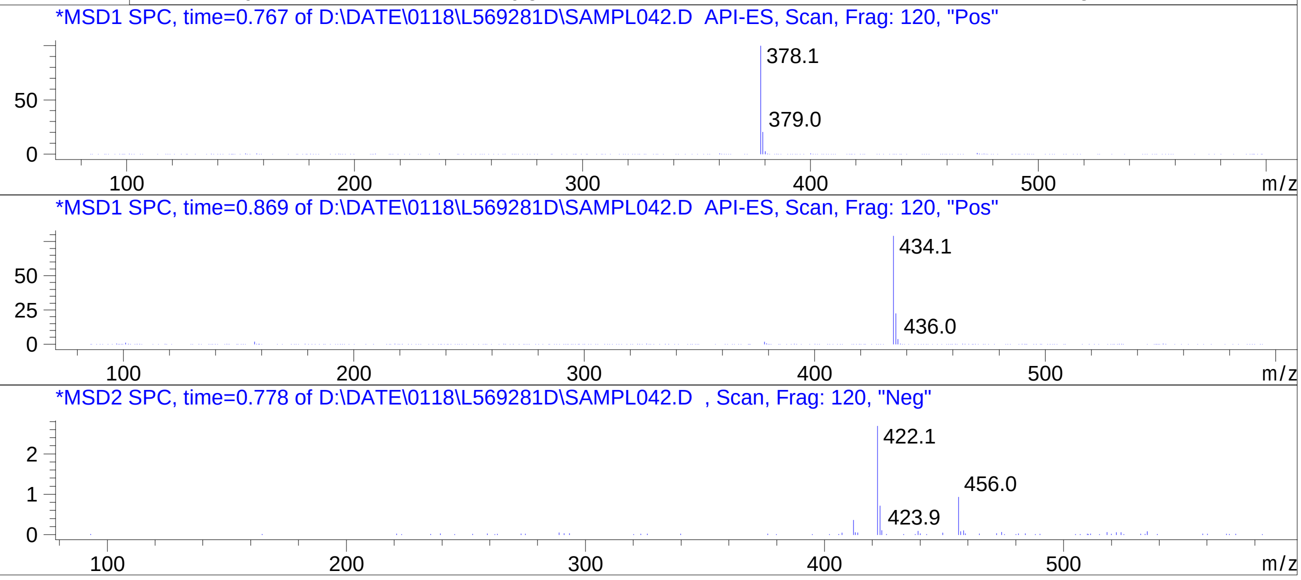


RT 0.768

RT 0.868

RT 0.772

Inj.Date 1/19/2023 E -VL- Acq. Method C:\HPCHEM\-> ->
