# Supplementary material for: An artificial intelligence accelerated virtual screening platform for drug discovery
Source: Nat Commun. 2024 Sep 5;15:7761. doi: 10.1038/s41467-024-52061-7 (PMC11377542; doi:10.1038/s41467-024-52061-7)
Supplement: Supplementary file 6 — Supplementary Data 3 [file 41467_2024_52061_MOESM6_ESM.zip › LC-MS-spectra/KLHDC2/Z4983979702.PDF]

MaxPeak: 100.00%
Ret_Time: 1.091 min

BA005647$8

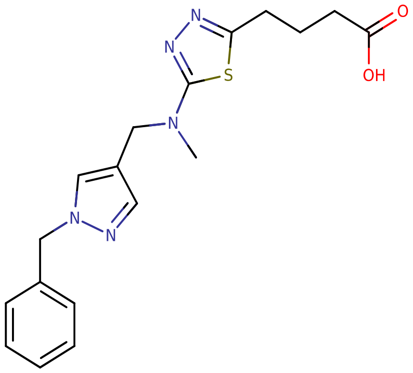

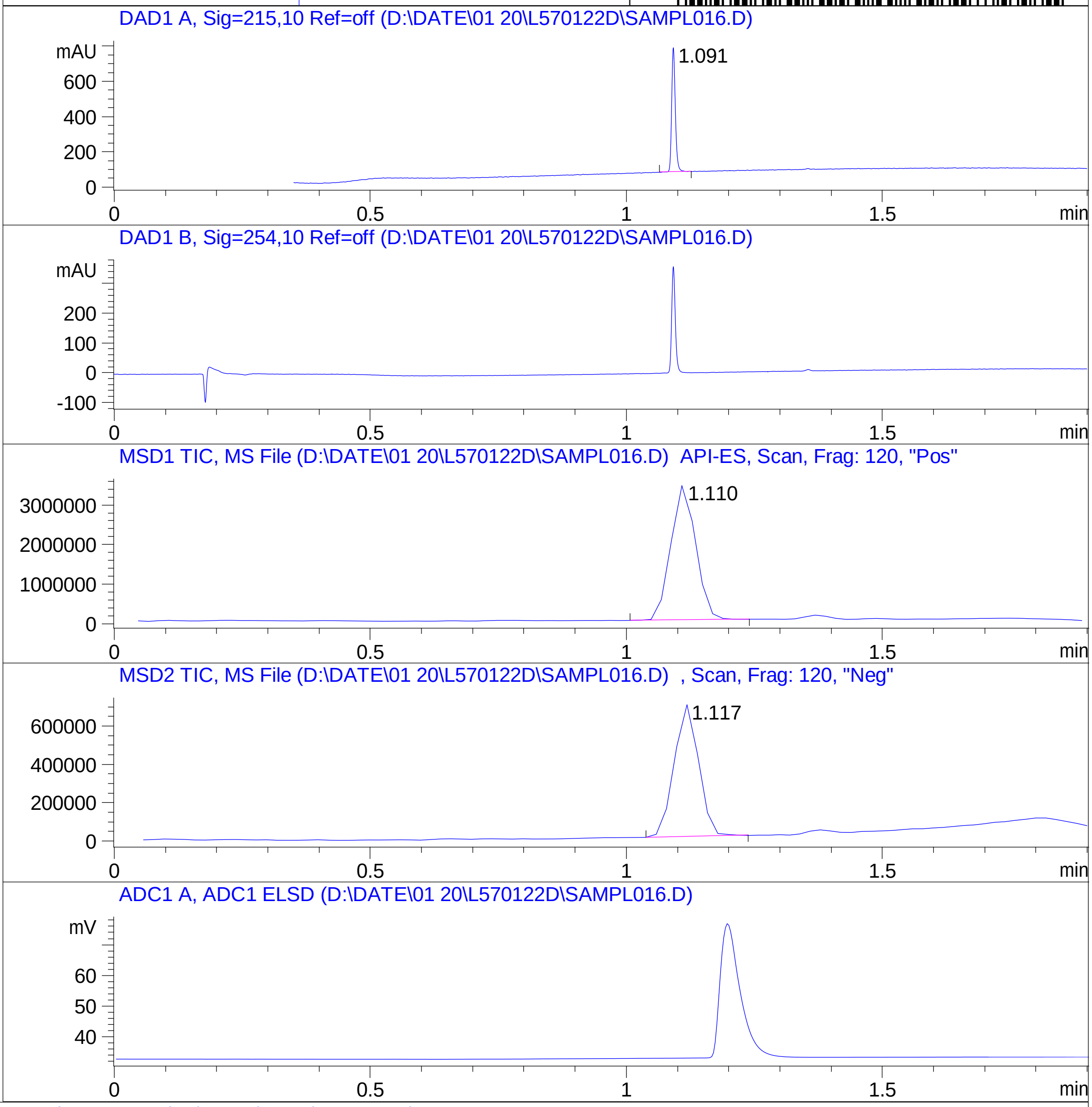

Mol Wt 371.46

Exact Mass 371.16

#	Time	Area%
1	1.091	100.00

RT 1.110

RT 1.117

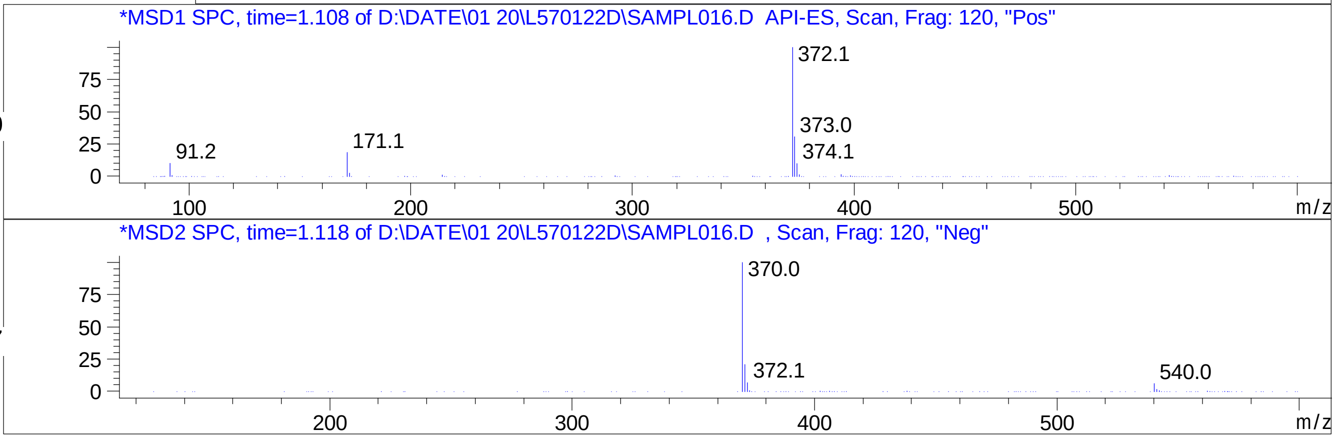

Inj.Date 1/20/2023 CH -SL- Acq. Method C:\HPCHEM\-> ->
