# Supplementary material for: An artificial intelligence accelerated virtual screening platform for drug discovery
Source: Nat Commun. 2024 Sep 5;15:7761. doi: 10.1038/s41467-024-52061-7 (PMC11377542; doi:10.1038/s41467-024-52061-7)
Supplement: Supplementary file 6 — Supplementary Data 3 [file 41467_2024_52061_MOESM6_ESM.zip › LC-MS-spectra/KLHDC2/Z7881785813.PDF]

MaxPeak: 98.31%
Ret_Time: 1.265 min

# BA005650$1

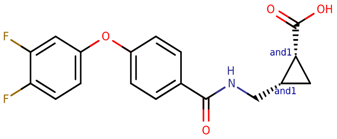


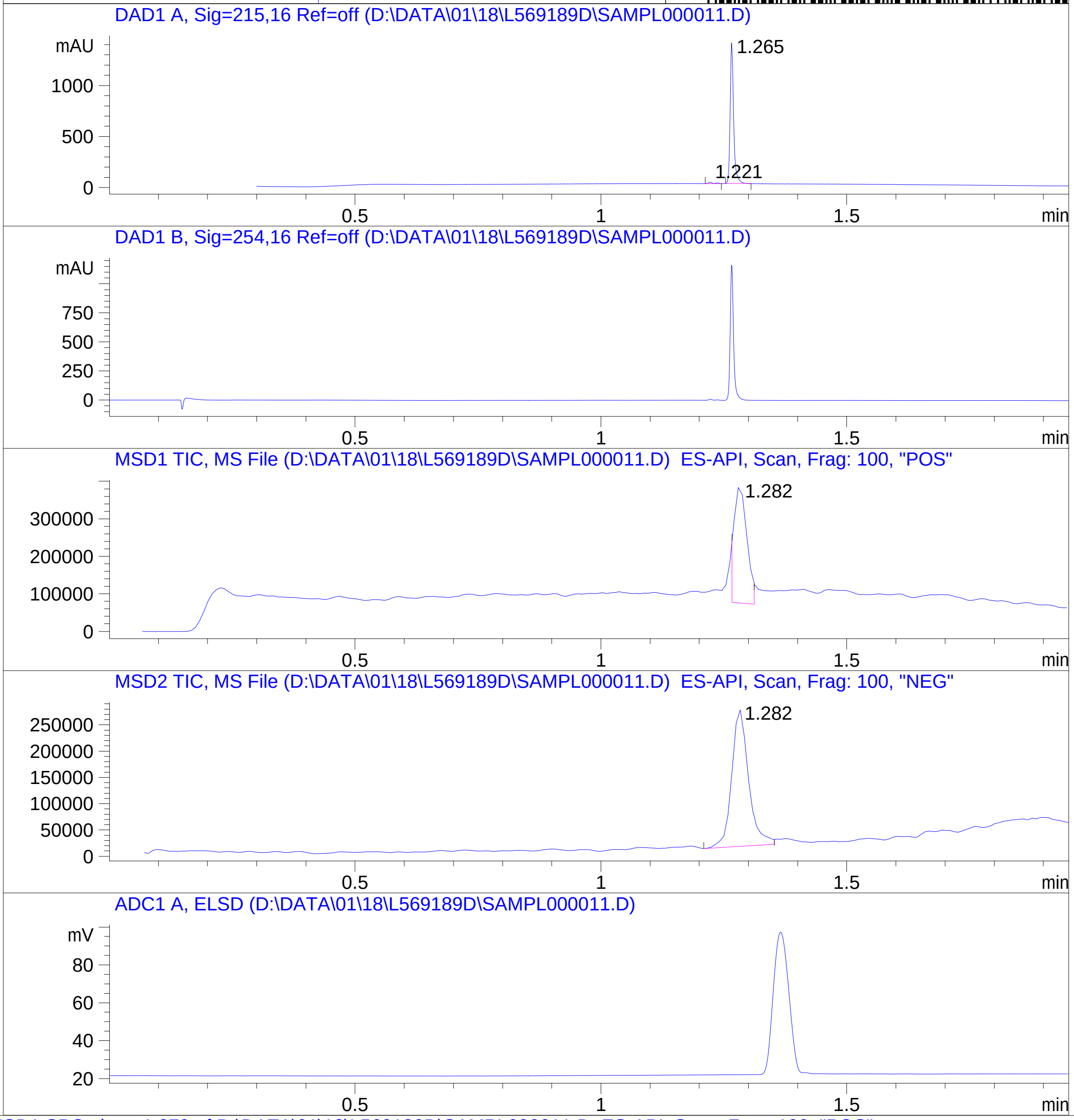


**Mol Wt** 347.31

**Exact Mass** 347.11

| # | Time | Area% |
|---|---|---|
| 1 | 1.221 | 1.69 |
| 2 | 1.265 | 98.31 |

RT 1.282

RT 1.282

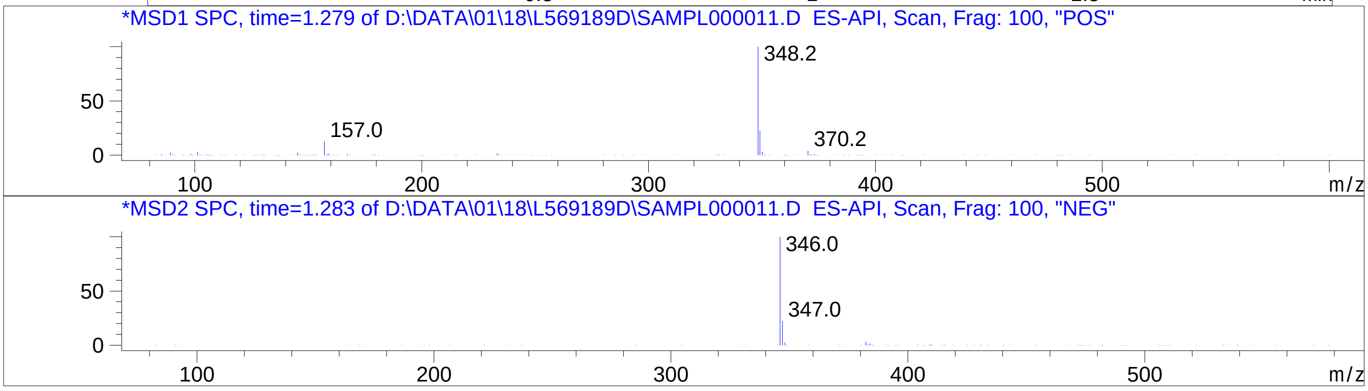


Inj.Date 1/18/2023 CH P2-B-01 -6- Acq. Method C:\CHEM32\-> ->
