# Supplementary material for: An artificial intelligence accelerated virtual screening platform for drug discovery
Source: Nat Commun. 2024 Sep 5;15:7761. doi: 10.1038/s41467-024-52061-7 (PMC11377542; doi:10.1038/s41467-024-52061-7)
Supplement: Supplementary file 6 — Supplementary Data 3 [file 41467_2024_52061_MOESM6_ESM.zip › LC-MS-spectra/KLHDC2/Z7881785753.PDF]

BA005649$1

MaxPeak: 100.00%
Ret_Time: 1.192 min

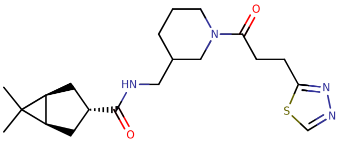

Mol Wt 390.54

Exact Mass 390.25

#	Time	Area%
1	1.192	100.00

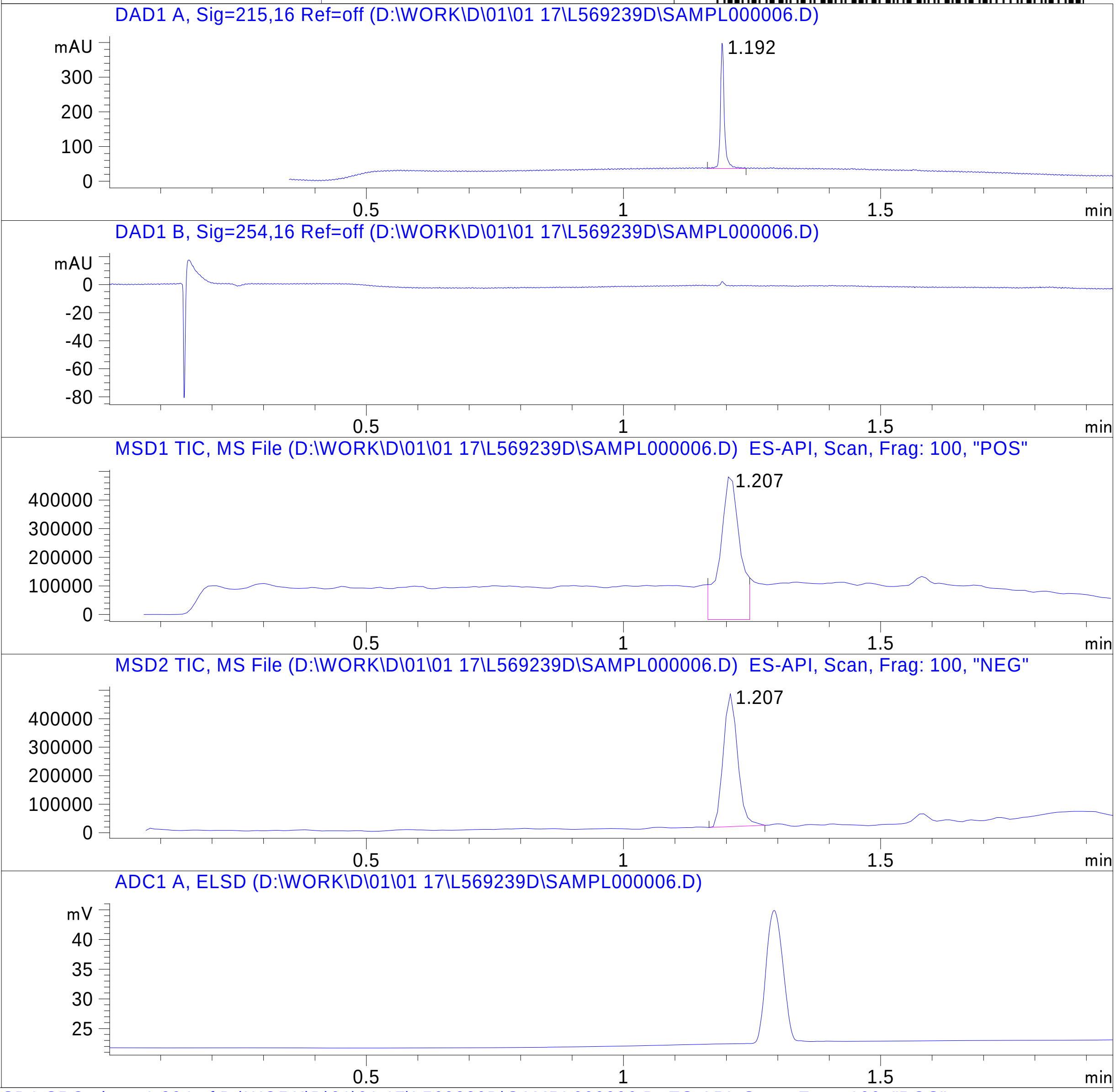

RT 1.207

RT 1.207

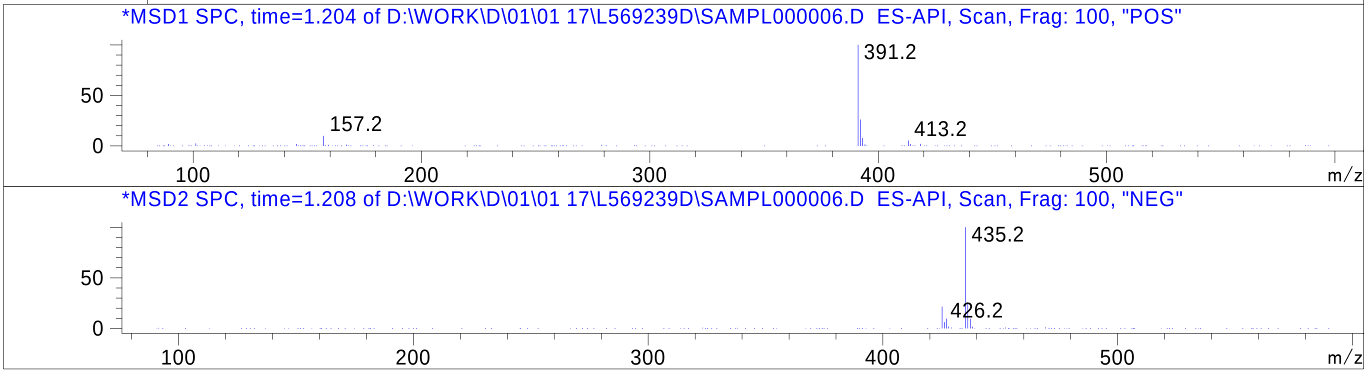

Inj.Date 1/18/2023 K P2-A-07 -6- Acq. Method C:\CHEM32\-> ->
