# Supplementary material for: An artificial intelligence accelerated virtual screening platform for drug discovery
Source: Nat Commun. 2024 Sep 5;15:7761. doi: 10.1038/s41467-024-52061-7 (PMC11377542; doi:10.1038/s41467-024-52061-7)
Supplement: Supplementary file 6 — Supplementary Data 3 [file 41467_2024_52061_MOESM6_ESM.zip › LC-MS-spectra/KLHDC2/Z3009405982.PDF]

MaxPeak: 94.01%
Ret_Time: 1.246 min

BA005645$1

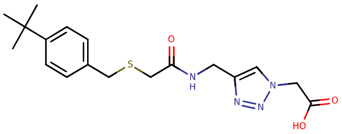

Mol Wt 376.47

Exact Mass 376.18

#	Time	Area%
1	1.183	2.64
2	1.219	3.36
3	1.246	94.01

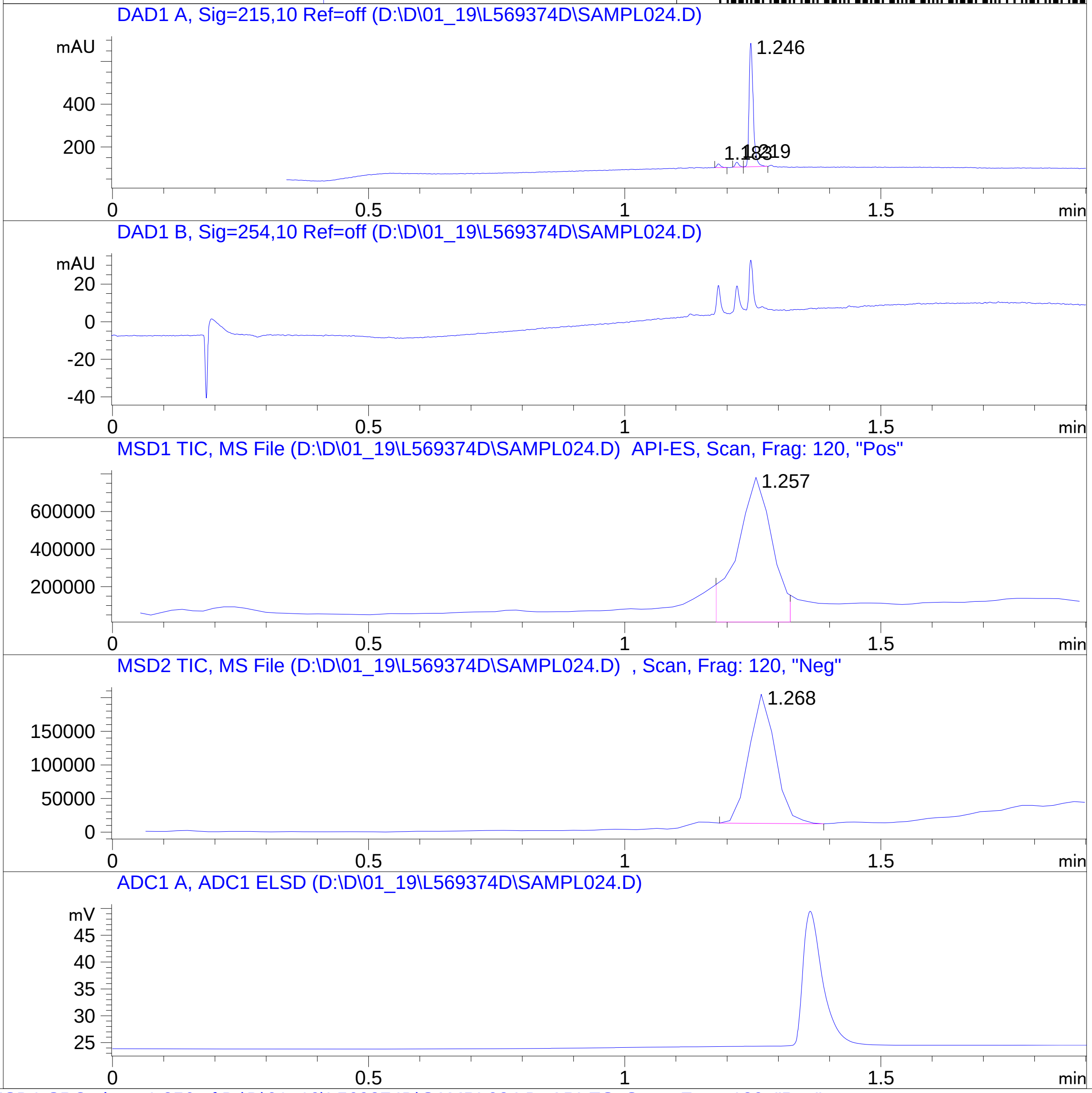

RT 1.257

RT 1.268

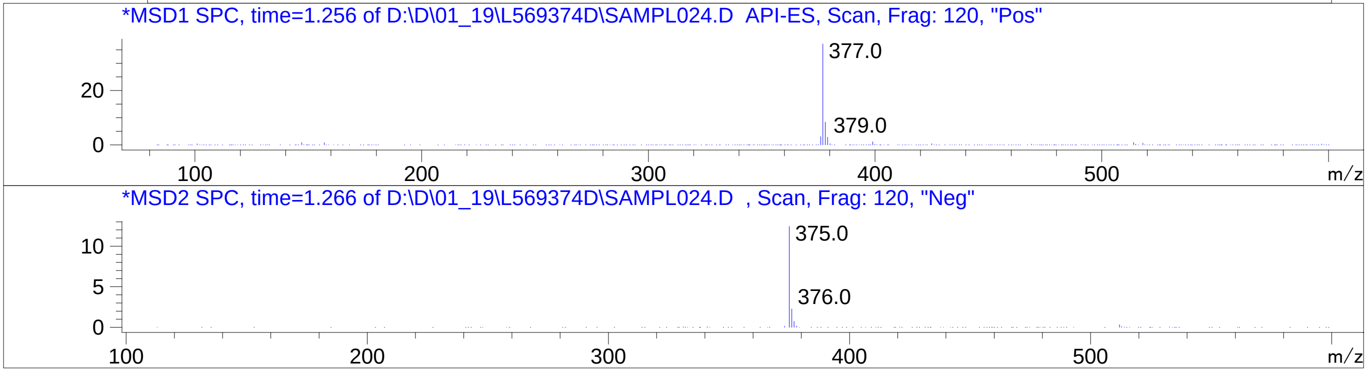

Inj.Date 1/19/2023 N -VL- Acq. Method C:\HPCHEM\-> ->
