# Supplementary material for: An artificial intelligence accelerated virtual screening platform for drug discovery
Source: Nat Commun. 2024 Sep 5;15:7761. doi: 10.1038/s41467-024-52061-7 (PMC11377542; doi:10.1038/s41467-024-52061-7)
Supplement: Supplementary file 6 — Supplementary Data 3 [file 41467_2024_52061_MOESM6_ESM.zip › LC-MS-spectra/KLHDC2/Z1903295352.PDF]

MaxPeak: 98.24%
Ret_Time: 0.755 min

BA005629$2

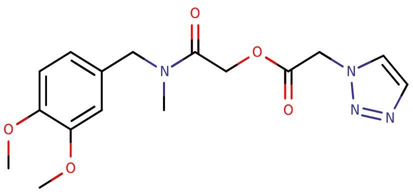

Mol Wt 348.35
Exact Mass 348.15

#	Time	Area%
1	0.755	98.24
2	1.089	1.76

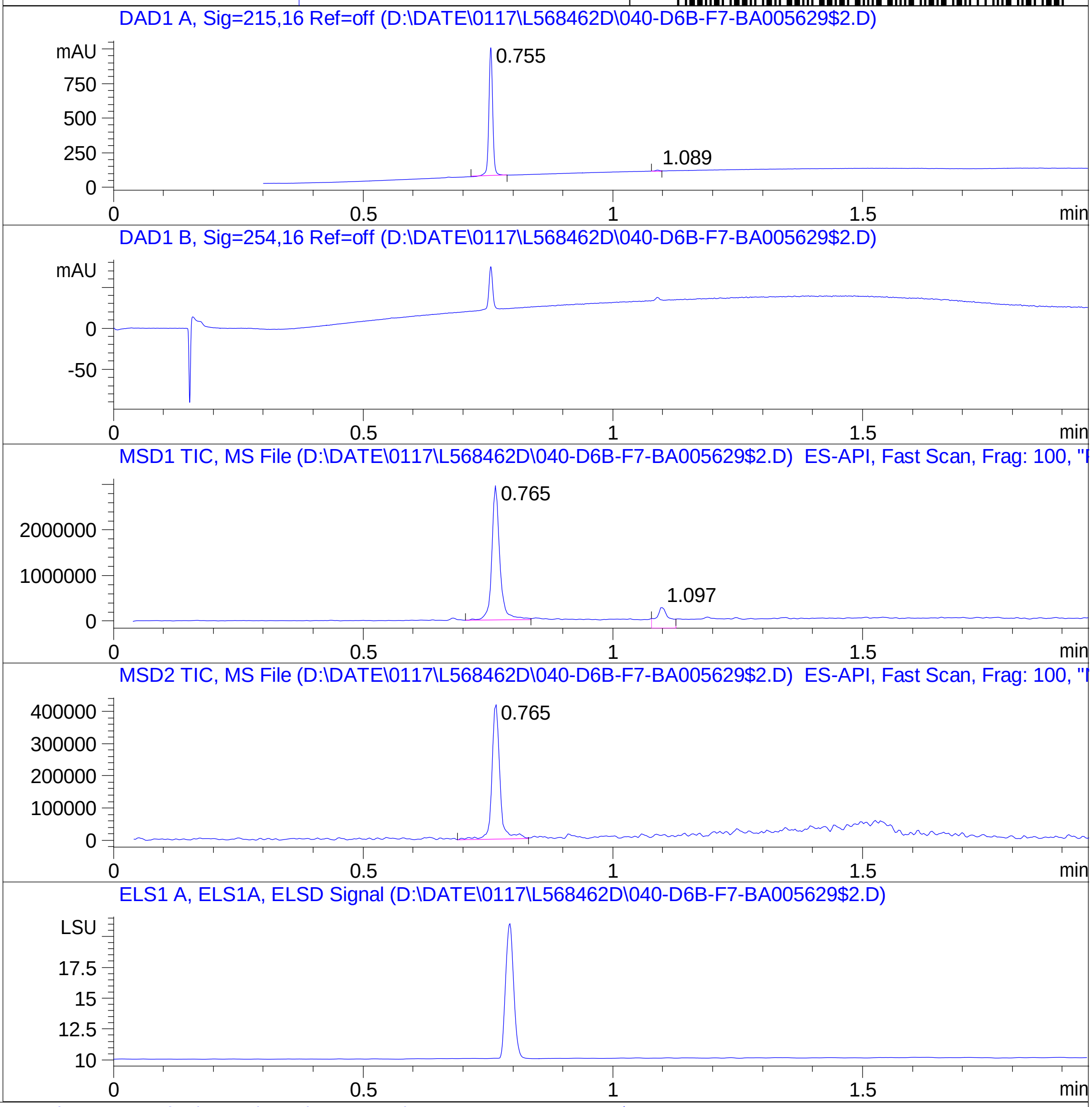

RT 0.765

RT 1.097

RT 0.765

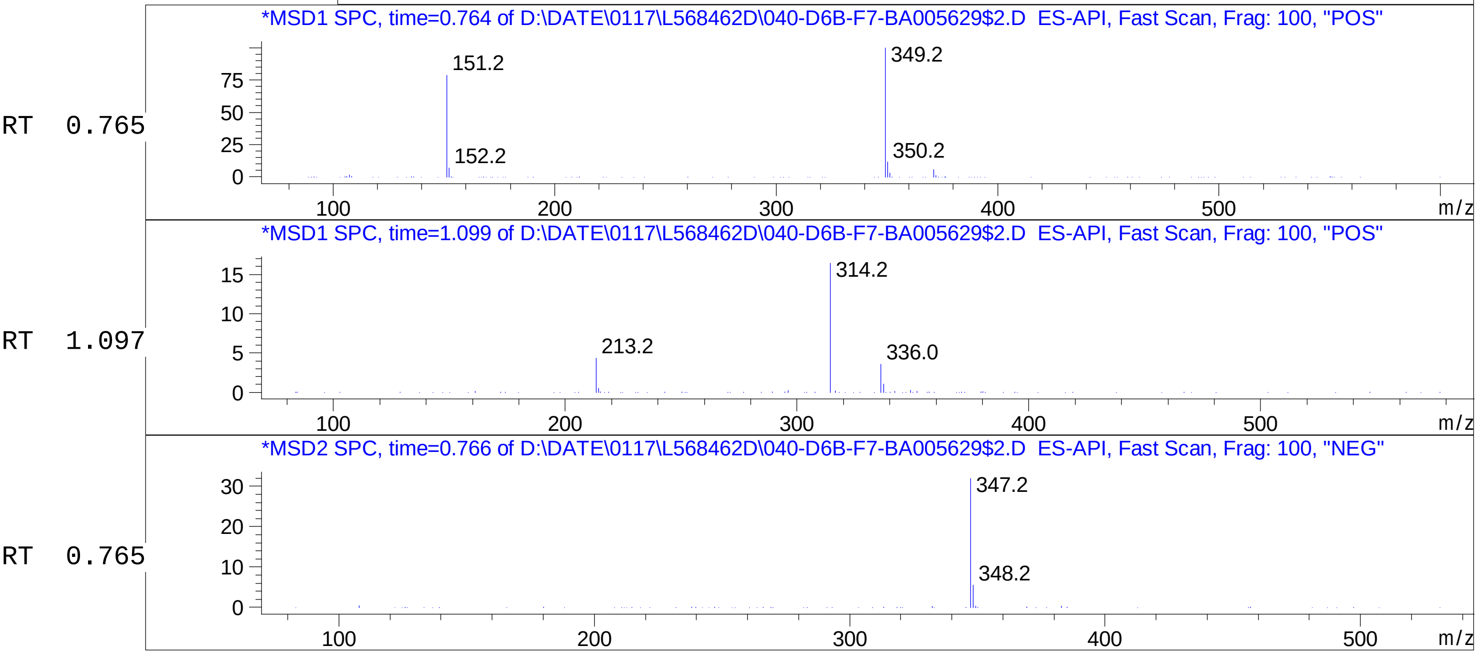

Inj.Date 1/17/2023 CH Acq. Method C:\Users\ -> ->
