# Supplementary material for: An artificial intelligence accelerated virtual screening platform for drug discovery
Source: Nat Commun. 2024 Sep 5;15:7761. doi: 10.1038/s41467-024-52061-7 (PMC11377542; doi:10.1038/s41467-024-52061-7)
Supplement: Supplementary file 6 — Supplementary Data 3 [file 41467_2024_52061_MOESM6_ESM.zip › LC-MS-spectra/KLHDC2/Z7881785950.PDF]

MaxPeak: 100.00%
Ret_Time: 0.714 min

BA005607$6

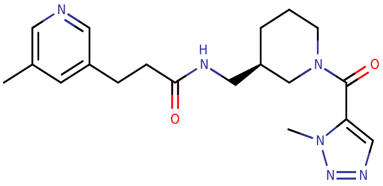

Mol Wt 370.45

Exact Mass 370.24

#	Time	Area%
1	0.714	100.00

DAD1 A, Sig=215,10 Ref=off (D:\DATE\0118\L569281D\SAMPL041.D)

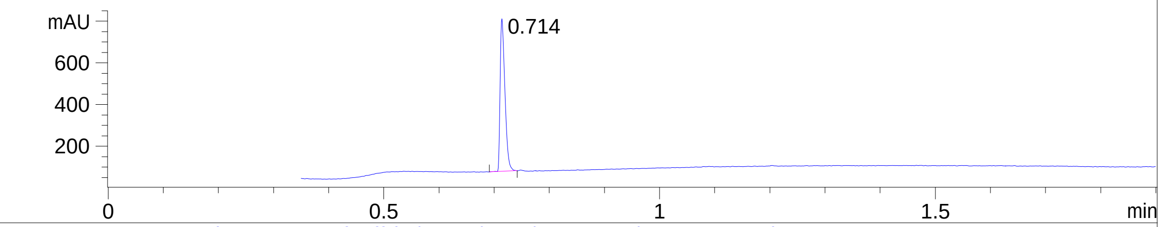

DAD1 B, Sig=254,10 Ref=off (D:\DATE\0118\L569281D\SAMPL041.D)

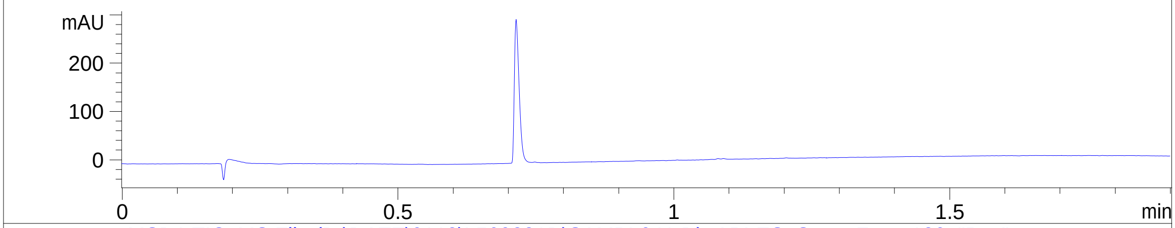

MSD1 TIC, MS File (D:\DATE\0118\L569281D\SAMPL041.D) API-ES, Scan, Frag: 120, "Pos"

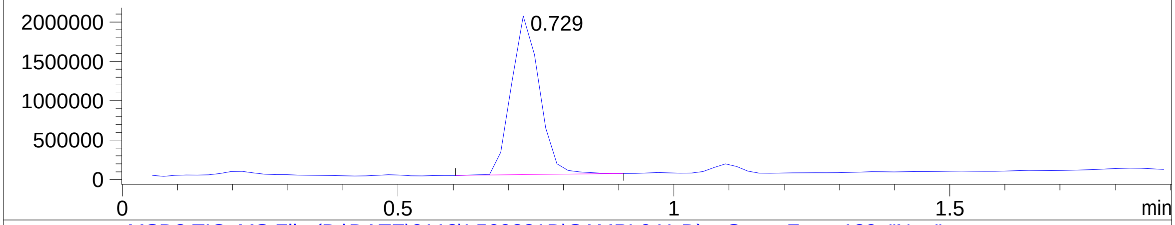

MSD2 TIC, MS File (D:\DATE\0118\L569281D\SAMPL041.D) , Scan, Frag: 120, "Neg"

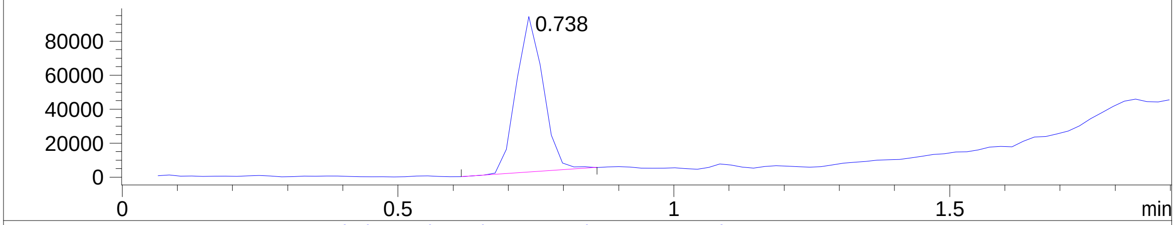

ADC1 A, ADC1 ELSD (D:\DATE\0118\L569281D\SAMPL041.D)

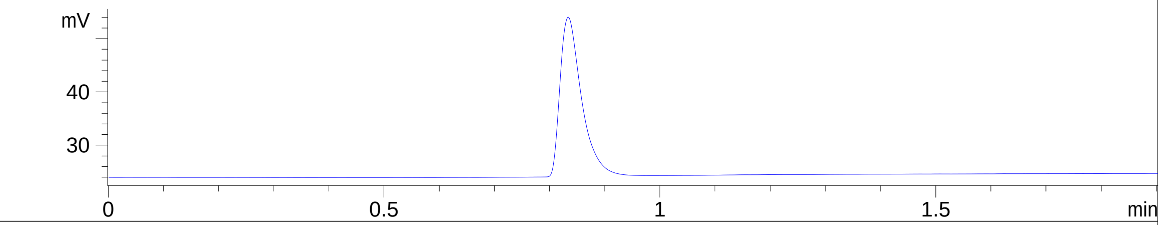

RT 0.729

*MSD1 SPC, time=0.727 of D:\DATE\0118\L569281D\SAMPL041.D API-ES, Scan, Frag: 120, "Pos"

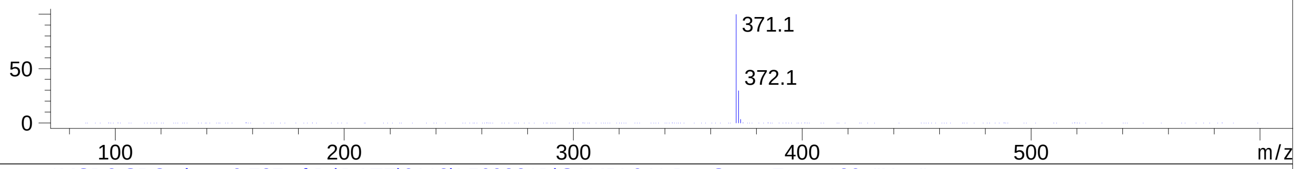

RT 0.738

*MSD2 SPC, time=0.737 of D:\DATE\0118\L569281D\SAMPL041.D , Scan, Frag: 120, "Neg"

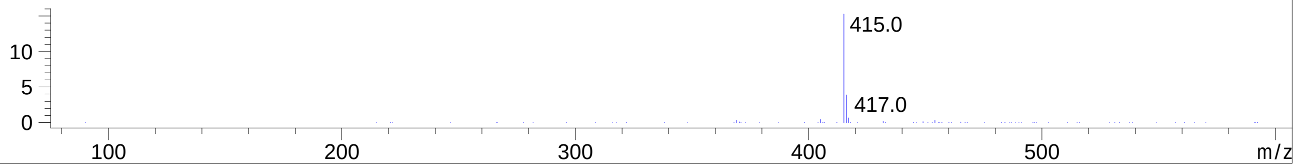

Inj.Date 1/19/2023 E -VL- Acq. Method C:\HPCHEM\-> ->
