# Supplementary material for: An artificial intelligence accelerated virtual screening platform for drug discovery
Source: Nat Commun. 2024 Sep 5;15:7761. doi: 10.1038/s41467-024-52061-7 (PMC11377542; doi:10.1038/s41467-024-52061-7)
Supplement: Supplementary file 6 — Supplementary Data 3 [file 41467_2024_52061_MOESM6_ESM.zip › LC-MS-spectra/KLHDC2/Z1991868966.PDF]

BA888179$2

```
MaxPeak:  100.00%
Ret_Time: 0.949 min
```

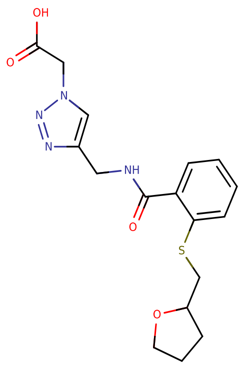

Mol Wt 376.43

Exact Mass 376.13

```
#  Time    Area%
------------------
1  0.949   100.00
```

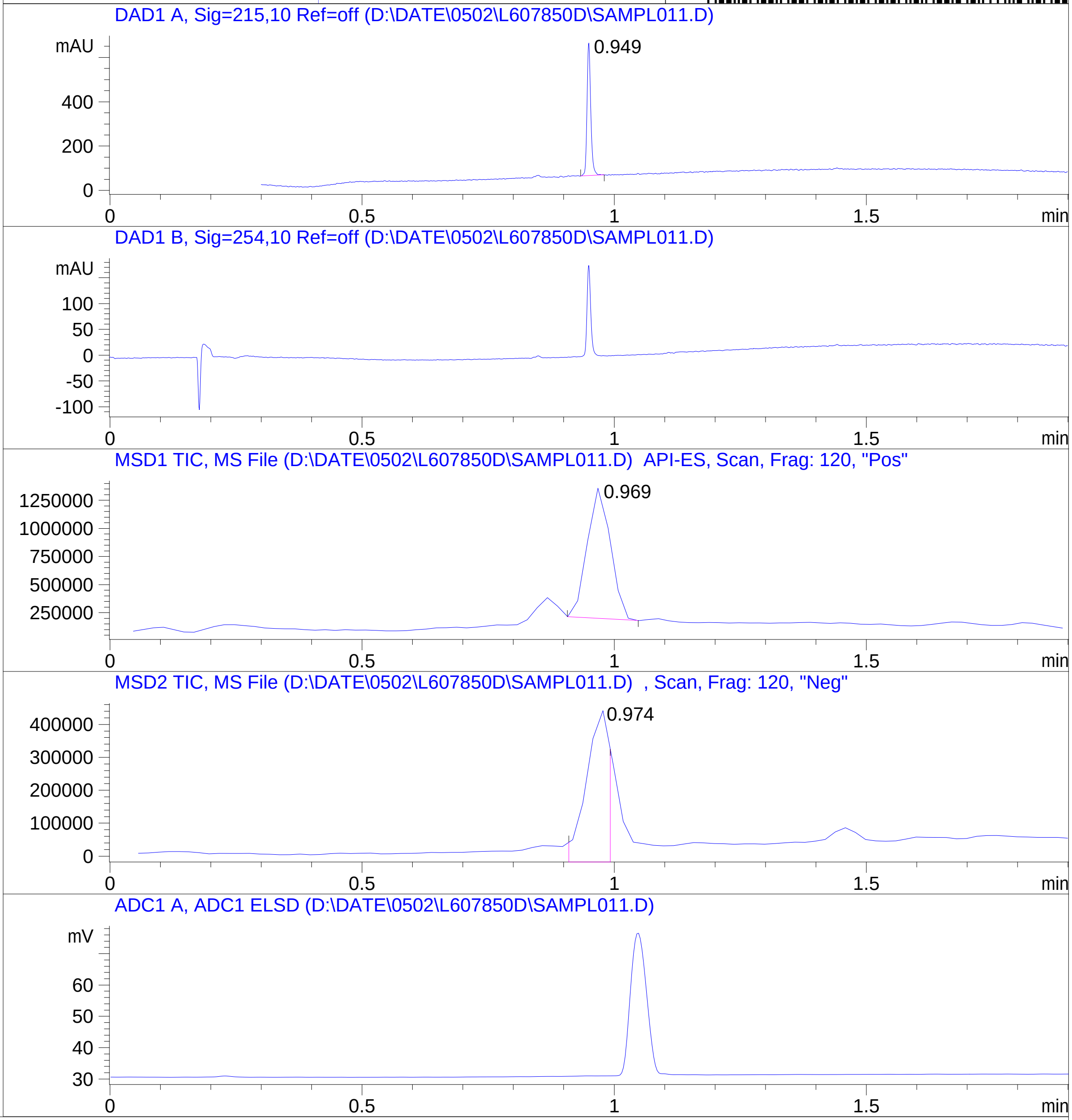

RT 0.969

RT 0.974

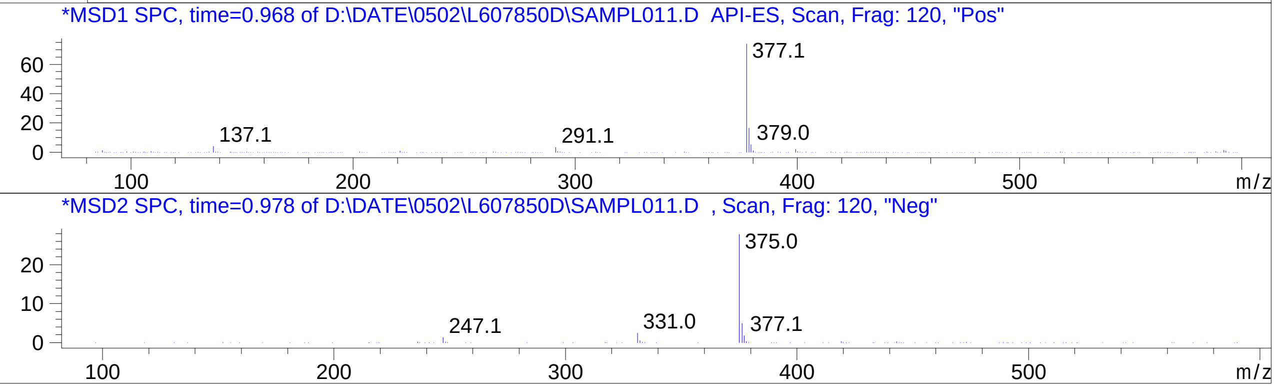


```
Inj.Date 5/3/2023          E          -SL-     Acq. Method C:\HPCHEM\->    ->
```
