# Supplementary material for: An artificial intelligence accelerated virtual screening platform for drug discovery
Source: Nat Commun. 2024 Sep 5;15:7761. doi: 10.1038/s41467-024-52061-7 (PMC11377542; doi:10.1038/s41467-024-52061-7)
Supplement: Supplementary file 6 — Supplementary Data 3 [file 41467_2024_52061_MOESM6_ESM.zip › LC-MS-spectra/KLHDC2/Z7881785983.PDF]

MaxPeak: 100.00%
Ret_Time: 1.014 min

BA005628$2

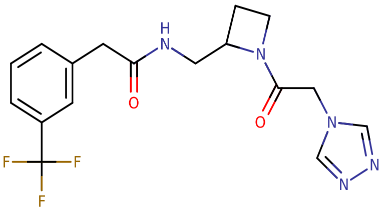

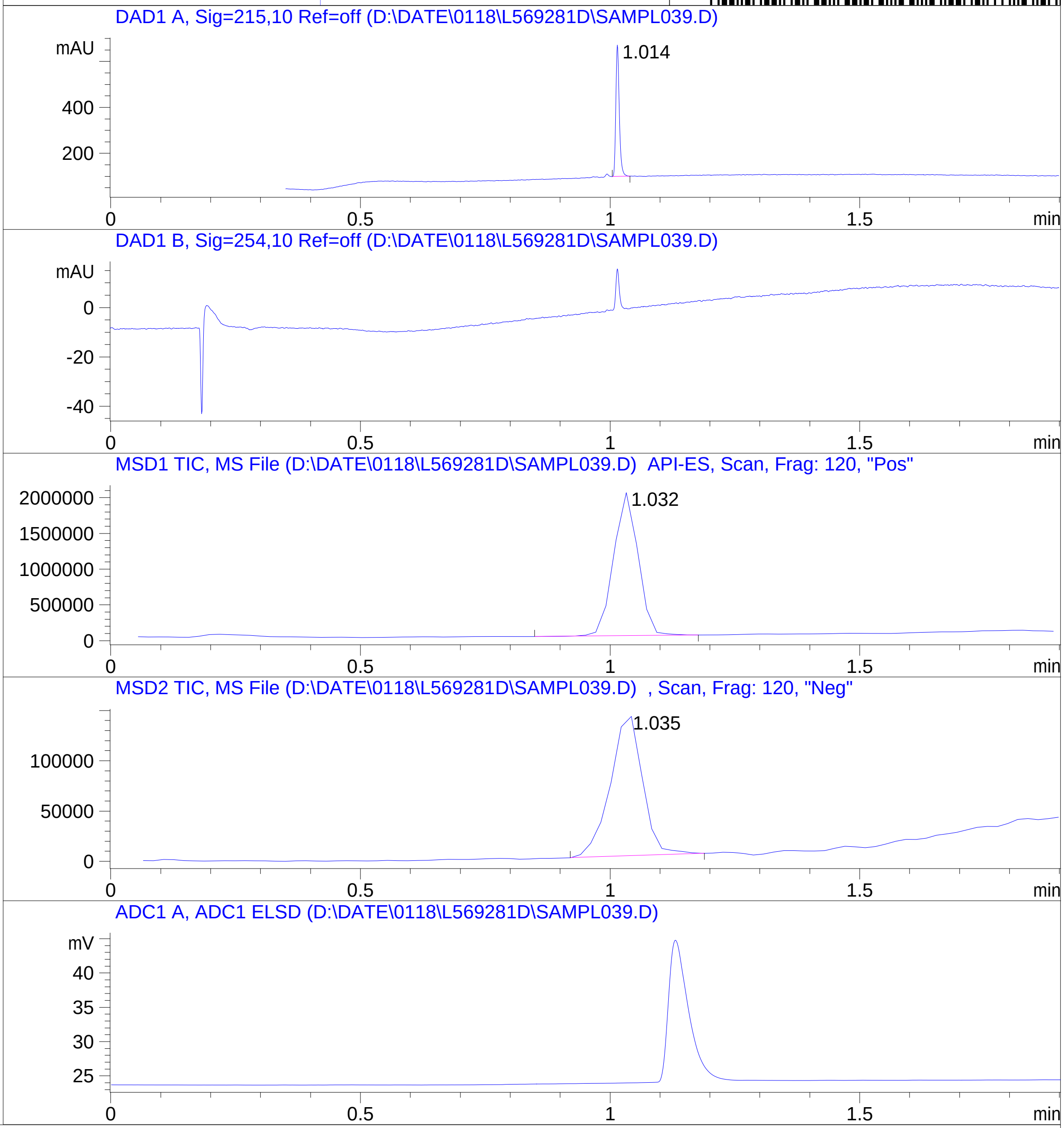

Mol Wt 381.35

Exact Mass 381.16

#	Time	Area%
1	1.014	100.00

RT 1.032

RT 1.035

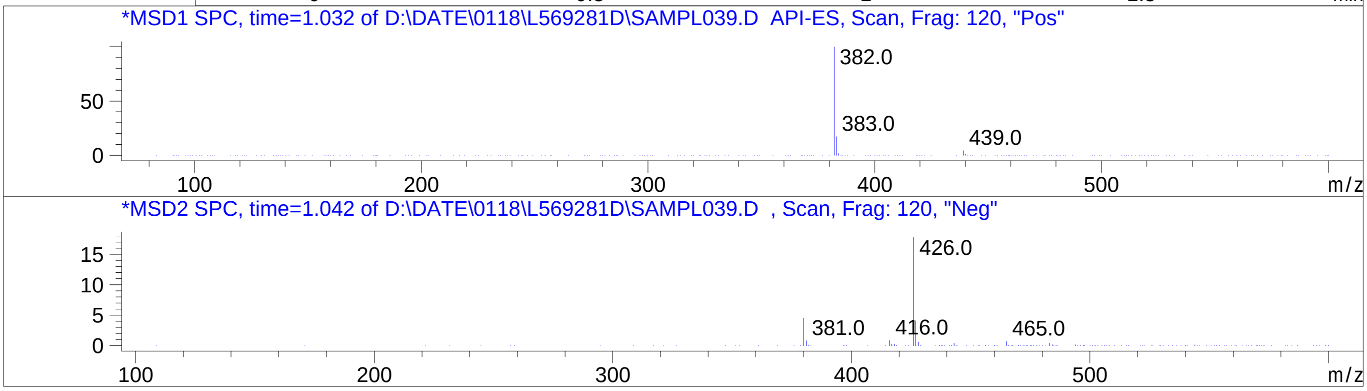

Inj.Date 1/19/2023   E   -VL-   Acq. Method C:\HPCHEM\->   ->
