# Supplementary material for: An artificial intelligence accelerated virtual screening platform for drug discovery
Source: Nat Commun. 2024 Sep 5;15:7761. doi: 10.1038/s41467-024-52061-7 (PMC11377542; doi:10.1038/s41467-024-52061-7)
Supplement: Supplementary file 6 — Supplementary Data 3 [file 41467_2024_52061_MOESM6_ESM.zip › LC-MS-spectra/KLHDC2/Z7881785940.PDF]

MaxPeak: 97.24%
Ret_Time: 0.913 min

# BA005657$2

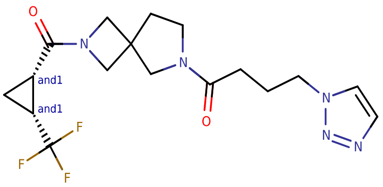


**Mol Wt** 385.38

**Exact Mass** 385.2

| # | Time | Area% |
|---|---|---|
| 1 | 0.913 | 97.24 |
| 2 | 1.401 | 2.76 |

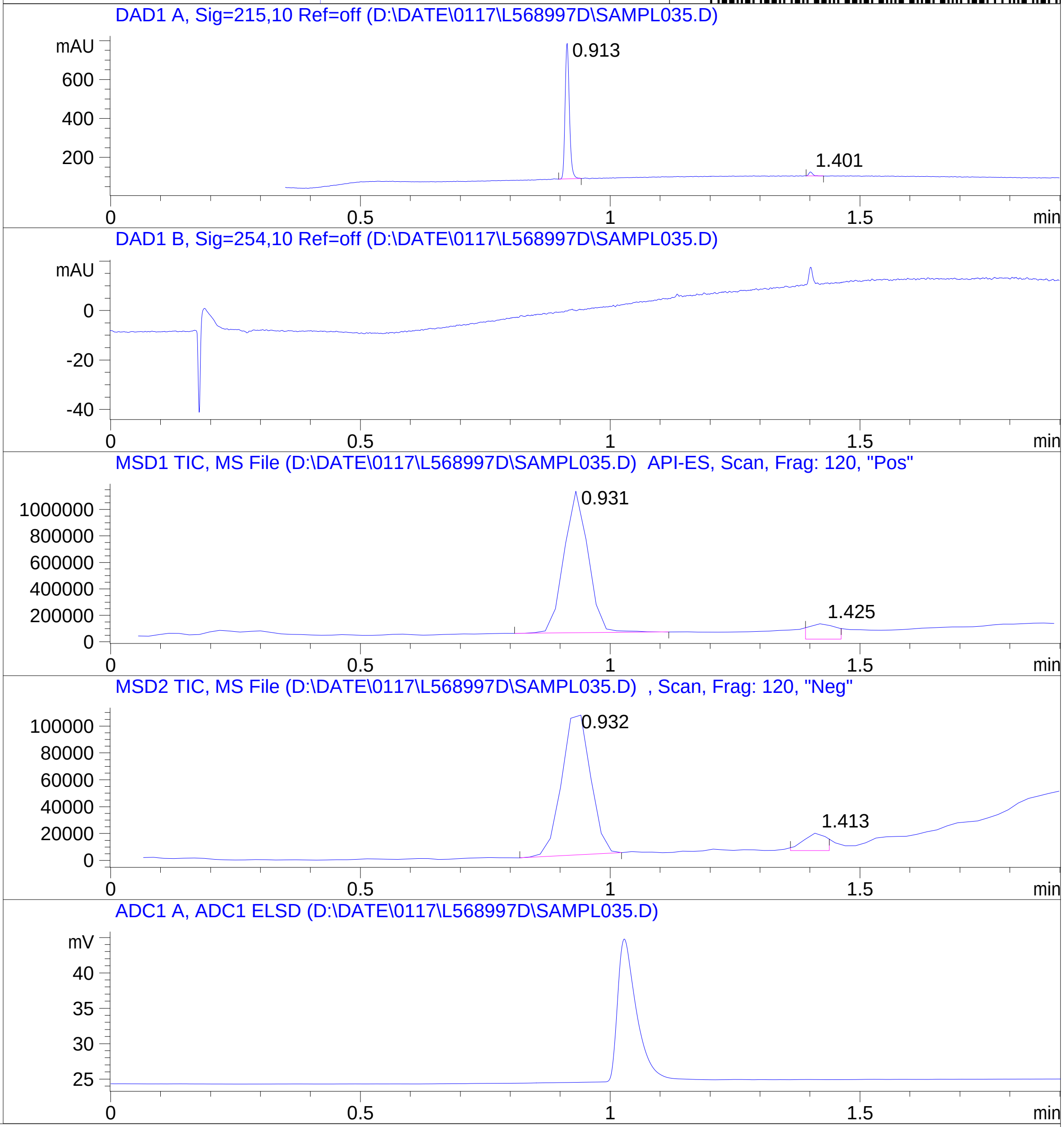


RT 0.931

RT 1.425

RT 0.932

RT 1.413

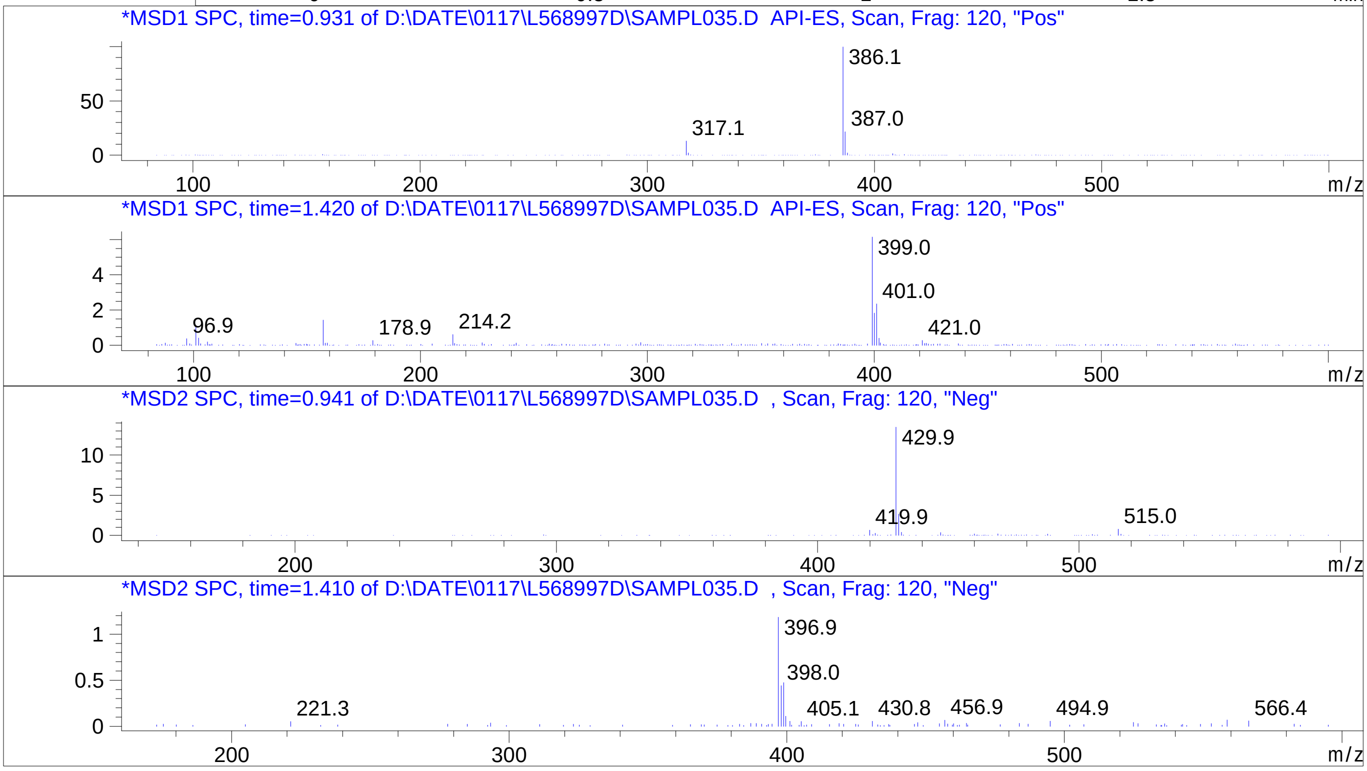


Inj.Date 1/18/2023 E -VL- Acq. Method C:\HPCHEM\-> ->
