# Supplementary material for: An artificial intelligence accelerated virtual screening platform for drug discovery
Source: Nat Commun. 2024 Sep 5;15:7761. doi: 10.1038/s41467-024-52061-7 (PMC11377542; doi:10.1038/s41467-024-52061-7)

BA005635$2

MaxPeak: 97.08%
Ret_Time: 0.704 min

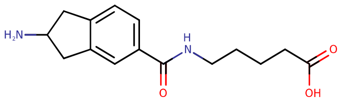

Mol Wt 276.33

Exact Mass 276.17

#	Time	Area%
1	0.704	97.08
2	0.753	2.92

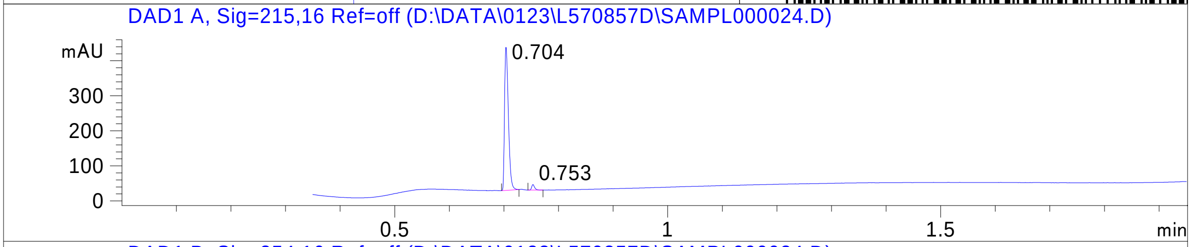

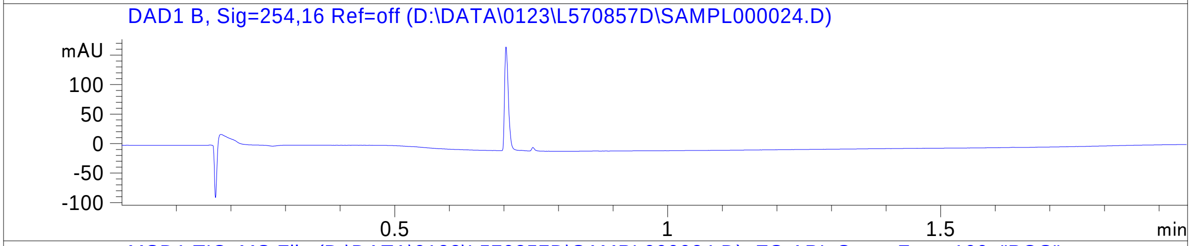

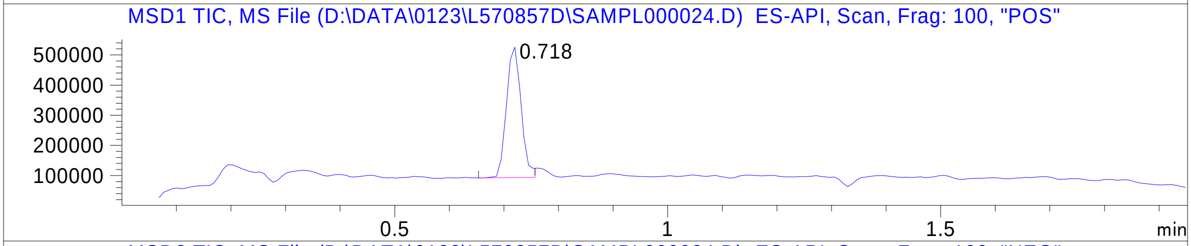

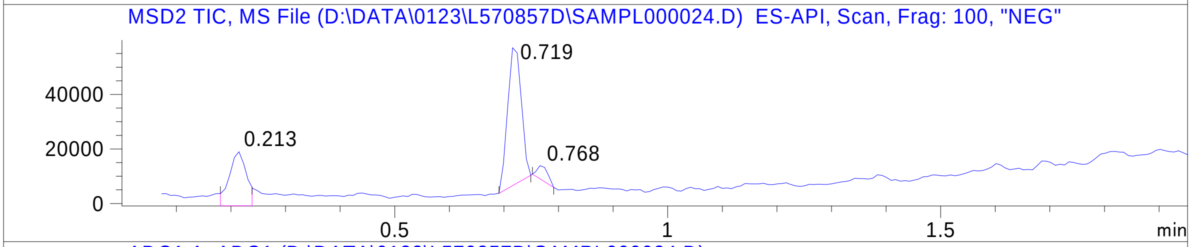

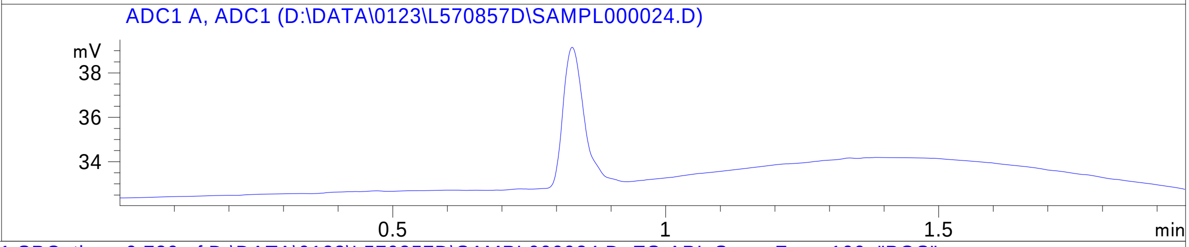

RT 0.718

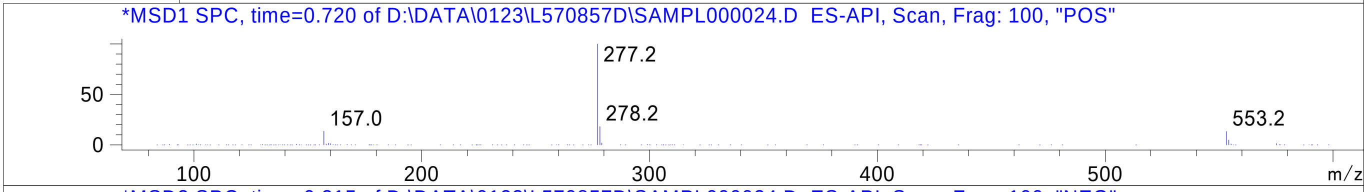

RT 0.213

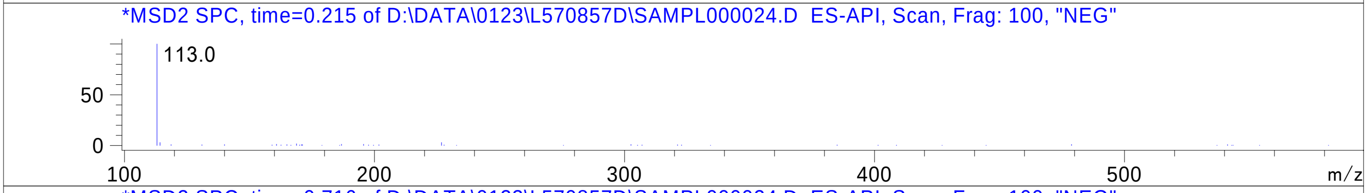

RT 0.719

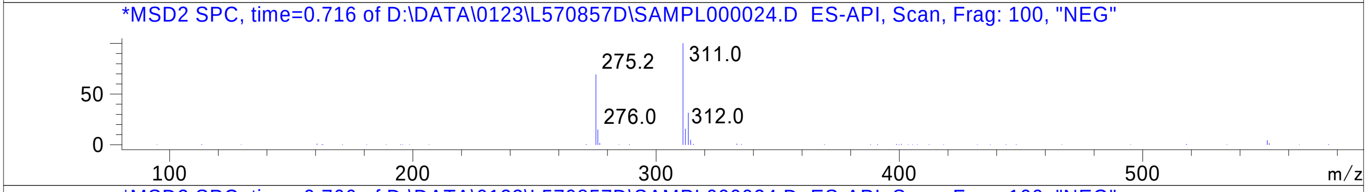

RT 0.768

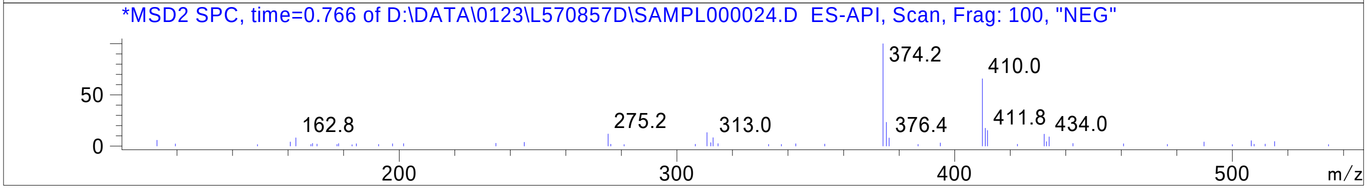

Supplement: Supplementary file 6 — Supplementary Data 3 [file 41467_2024_52061_MOESM6_ESM.zip › LC-MS-spectra/KLHDC2/Z7881785919.PDF]
