# Supplementary material for: An artificial intelligence accelerated virtual screening platform for drug discovery
Source: Nat Commun. 2024 Sep 5;15:7761. doi: 10.1038/s41467-024-52061-7 (PMC11377542; doi:10.1038/s41467-024-52061-7)
Supplement: Supplementary file 6 — Supplementary Data 3 [file 41467_2024_52061_MOESM6_ESM.zip › LC-MS-spectra/KLHDC2/Z7881785649.PDF]

MaxPeak: 94.59%
Ret_Time: 1.068 min

BA005640$2

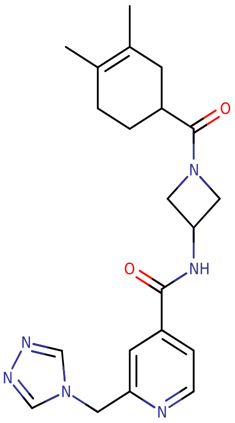

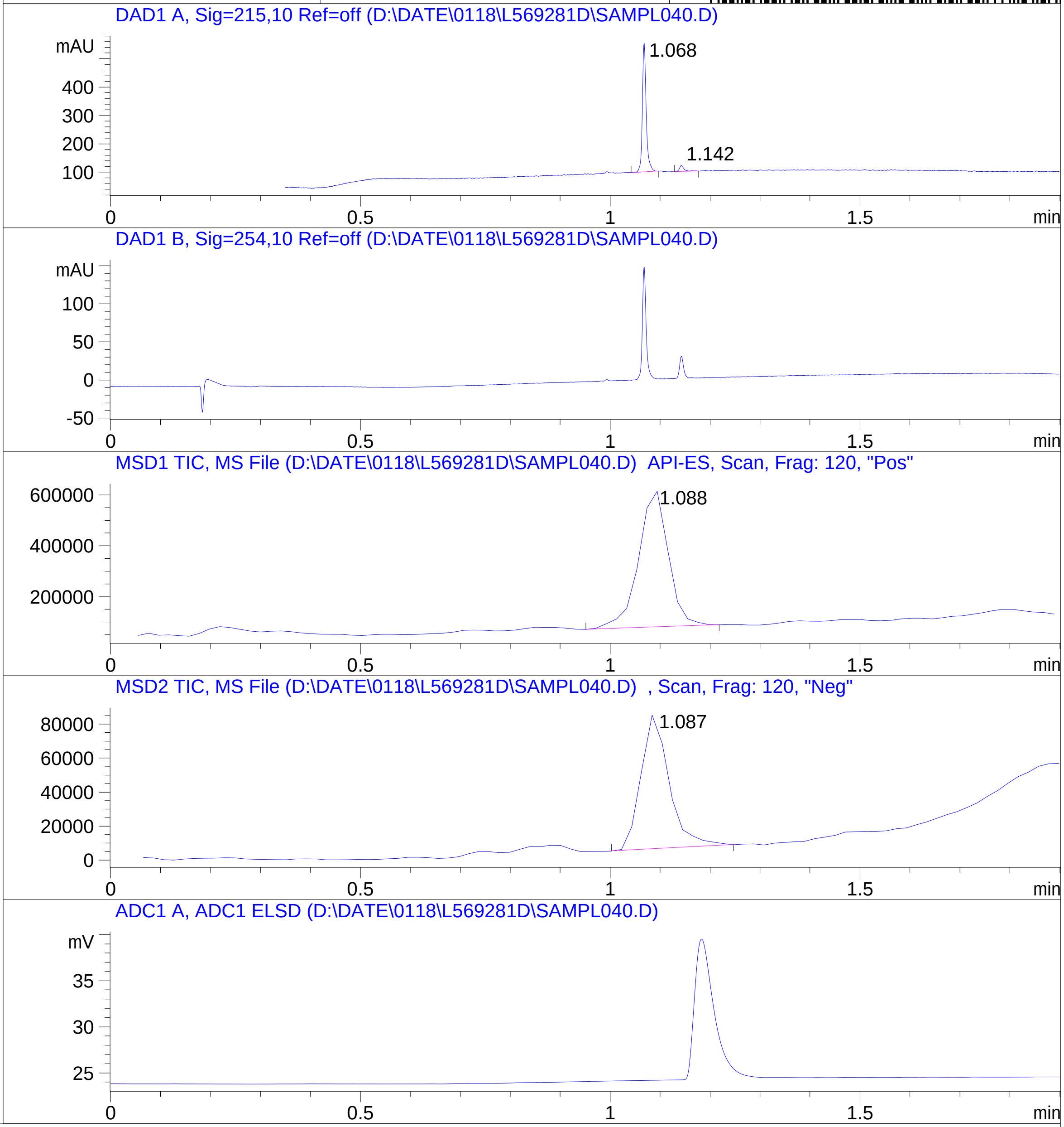

Mol Wt 394.47

Exact Mass 394.24

#	Time	Area%
1	1.068	94.59
2	1.142	5.41

RT 1.088

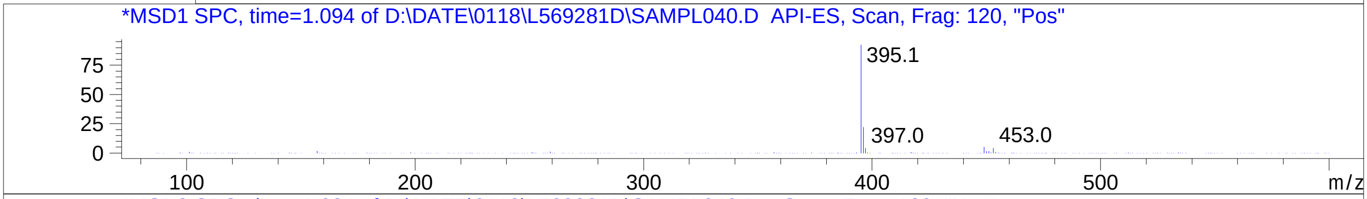

RT 1.087

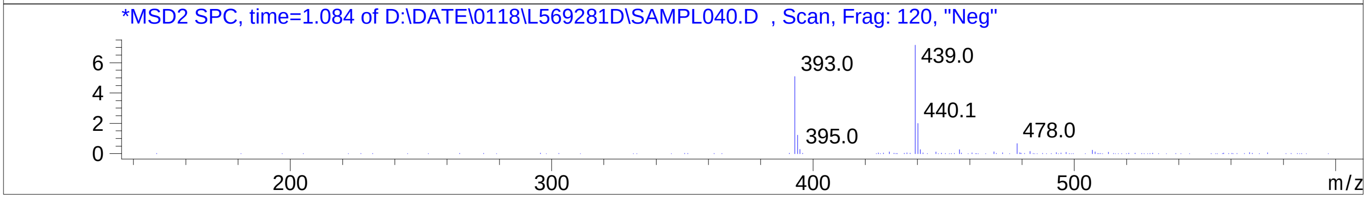

Inj.Date 1/19/2023 E -VL- Acq. Method C:\HPCHEM\-> ->
