# Supplementary material for: An artificial intelligence accelerated virtual screening platform for drug discovery
Source: Nat Commun. 2024 Sep 5;15:7761. doi: 10.1038/s41467-024-52061-7 (PMC11377542; doi:10.1038/s41467-024-52061-7)

MaxPeak: 93.00%
Ret_Time: 1.020 min

BA888184$3

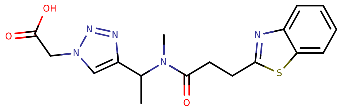

Mol Wt 373.43

Exact Mass 373.13

#	Time	Area%
1	0.927	1.80
2	1.020	93.00
3	1.041	1.02
4	1.428	4.17

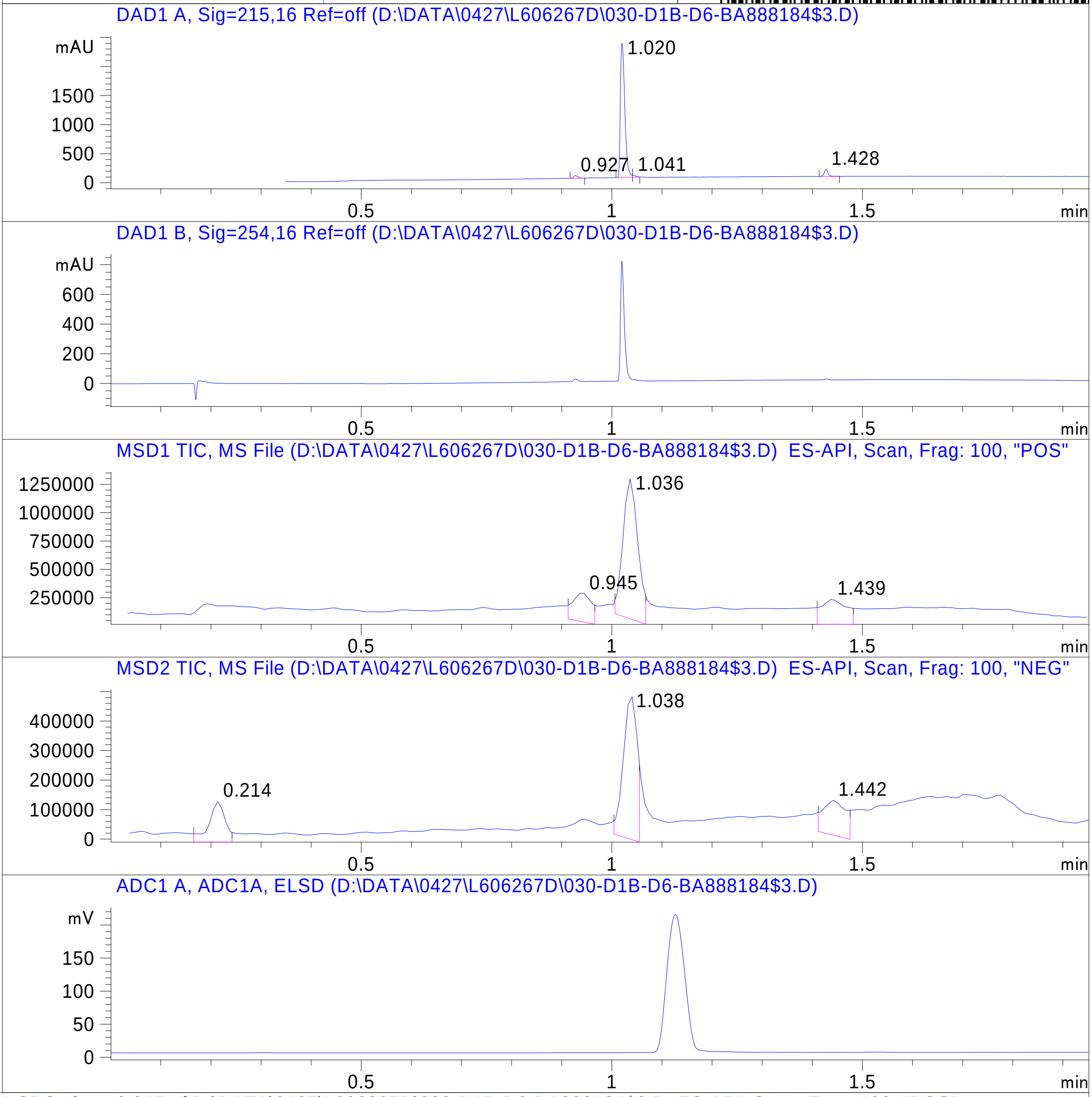

RT 0.945

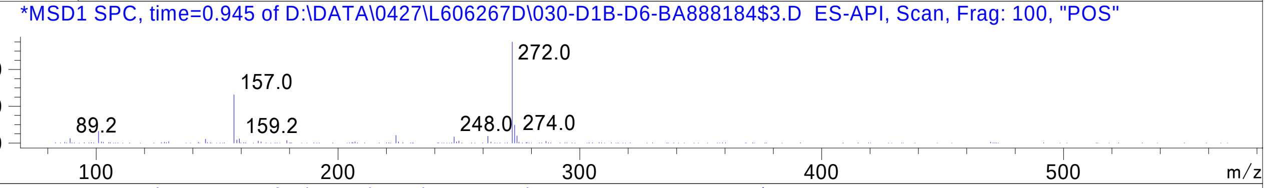

RT 1.036

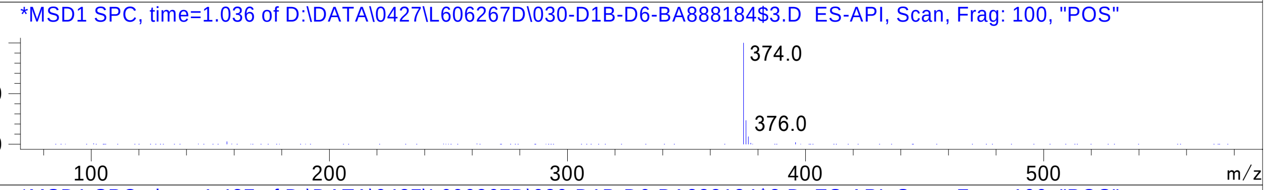

RT 1.439

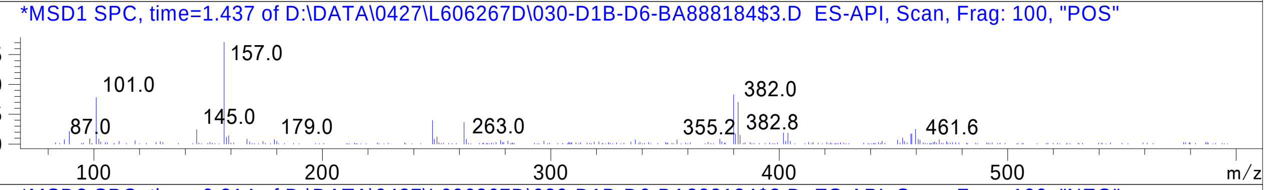

RT 0.214

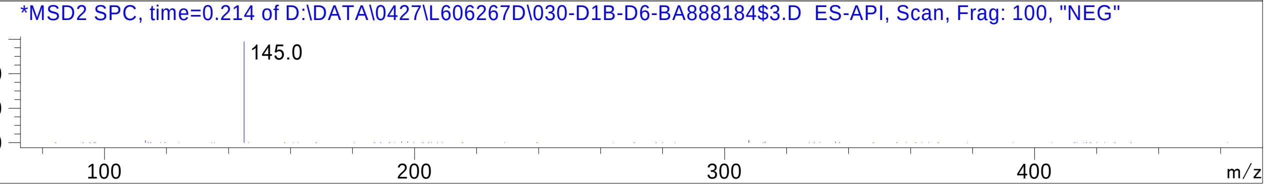

RT 1.038

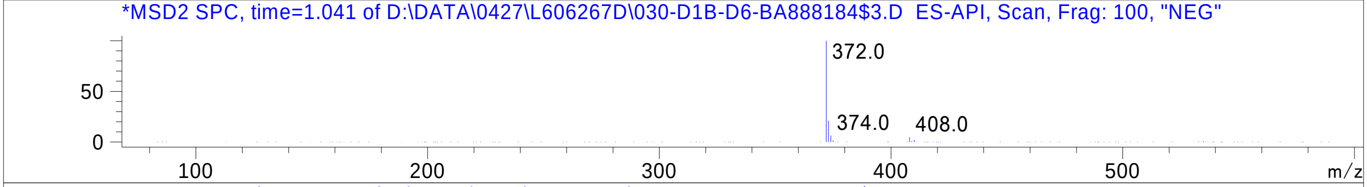

RT 1.442

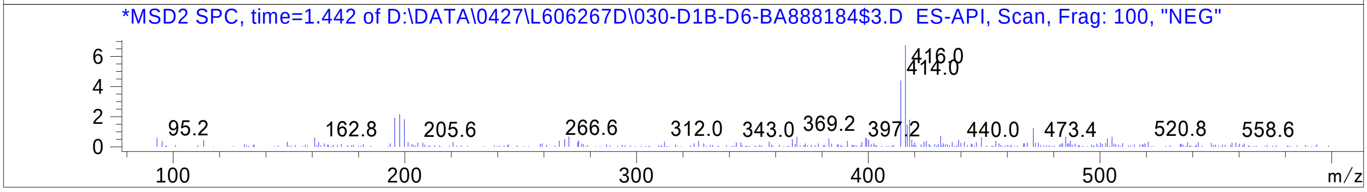

Supplement: Supplementary file 6 — Supplementary Data 3 [file 41467_2024_52061_MOESM6_ESM.zip › LC-MS-spectra/KLHDC2/Z8381047286.PDF]
