# Supplementary material for: An artificial intelligence accelerated virtual screening platform for drug discovery
Source: Nat Commun. 2024 Sep 5;15:7761. doi: 10.1038/s41467-024-52061-7 (PMC11377542; doi:10.1038/s41467-024-52061-7)

MaxPeak: 68.51%
Ret_Time: 0.913 min

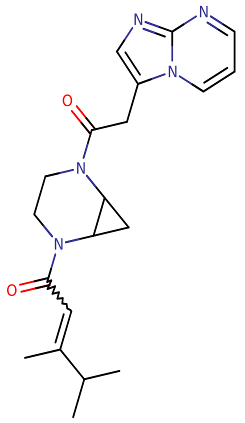

Mol Wt 367.44

Exact Mass 367.23

#	Time	Area%
1	0.913	68.51
2	0.927	31.49

BA005614$1

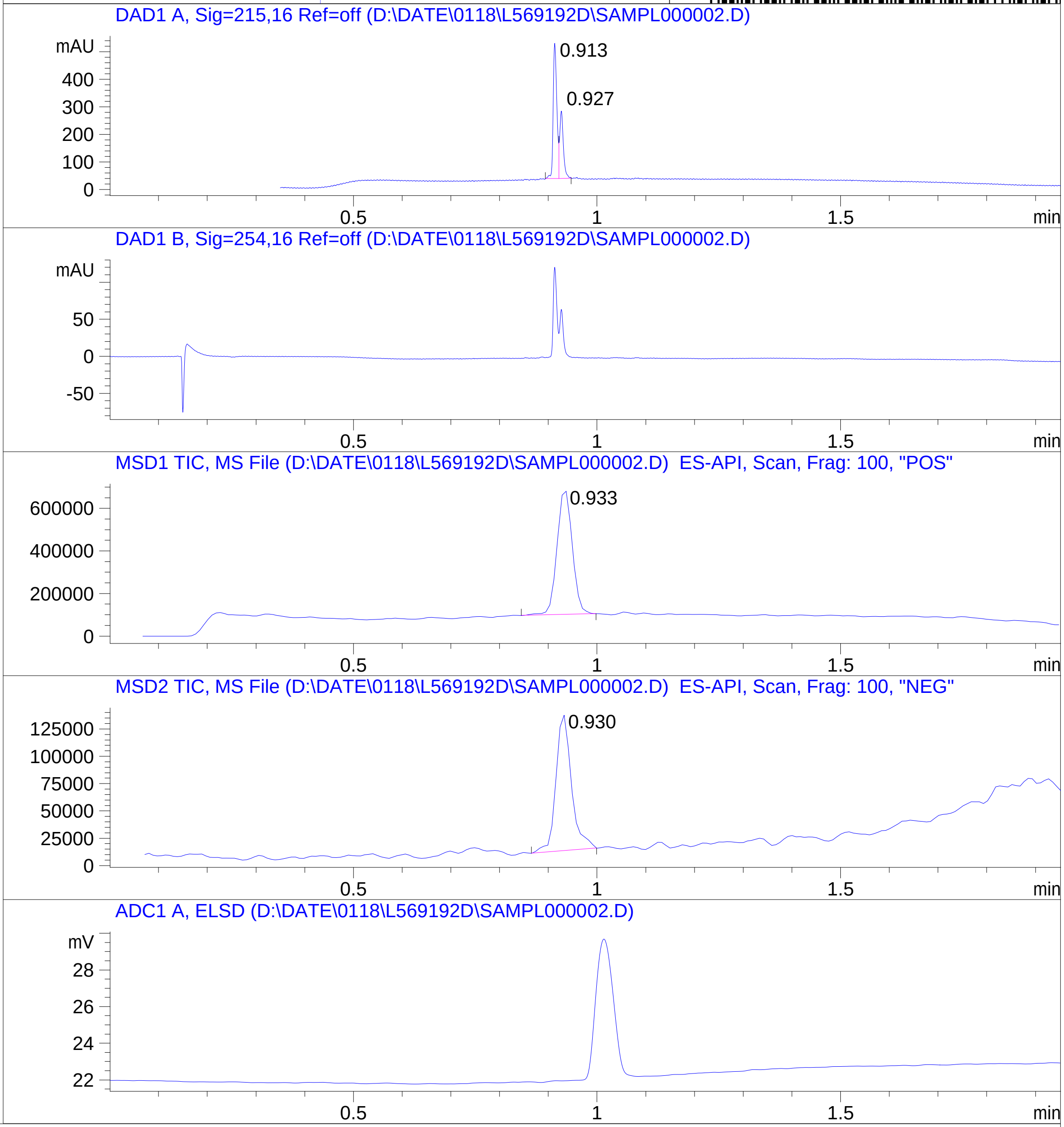

RT 0.933

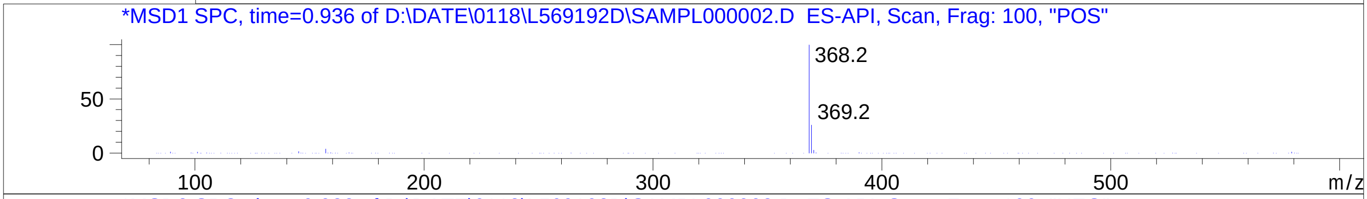

RT 0.930

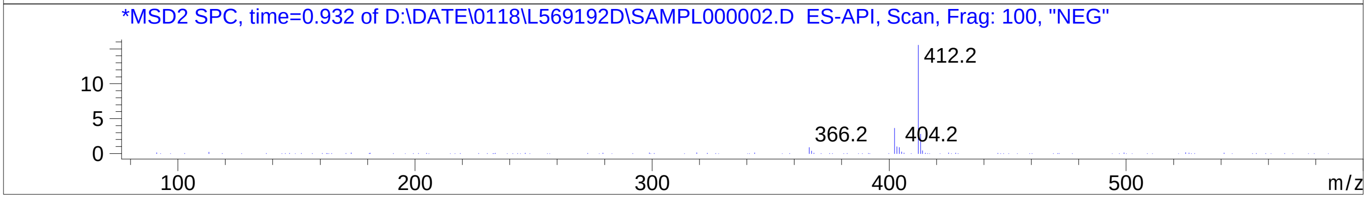

Supplement: Supplementary file 6 — Supplementary Data 3 [file 41467_2024_52061_MOESM6_ESM.zip › LC-MS-spectra/KLHDC2/Z7881785896.PDF]
