# Supplementary material for: An artificial intelligence accelerated virtual screening platform for drug discovery
Source: Nat Commun. 2024 Sep 5;15:7761. doi: 10.1038/s41467-024-52061-7 (PMC11377542; doi:10.1038/s41467-024-52061-7)
Supplement: Supplementary file 6 — Supplementary Data 3 [file 41467_2024_52061_MOESM6_ESM.zip › LC-MS-spectra/KLHDC2/Z8381047291.PDF]

BA888186$3

MaxPeak: 93.87%
Ret_Time: 0.686 min

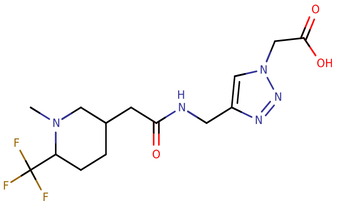

Mol Wt 363.34

Exact Mass 363.17

#	Time	Area%
1	0.686	93.87
2	1.147	6.13

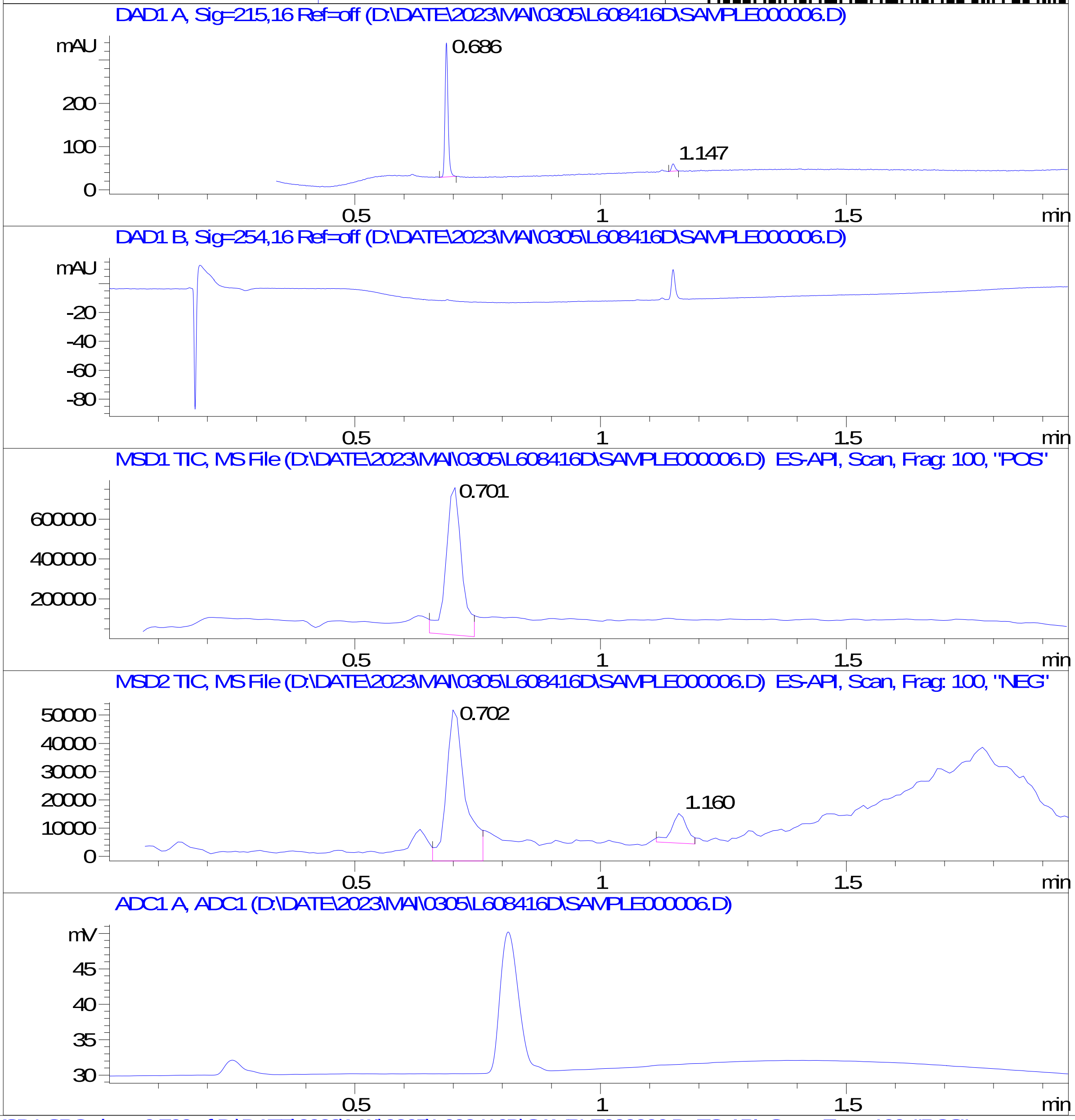

RT 0.701

RT 0.702

RT 1.160

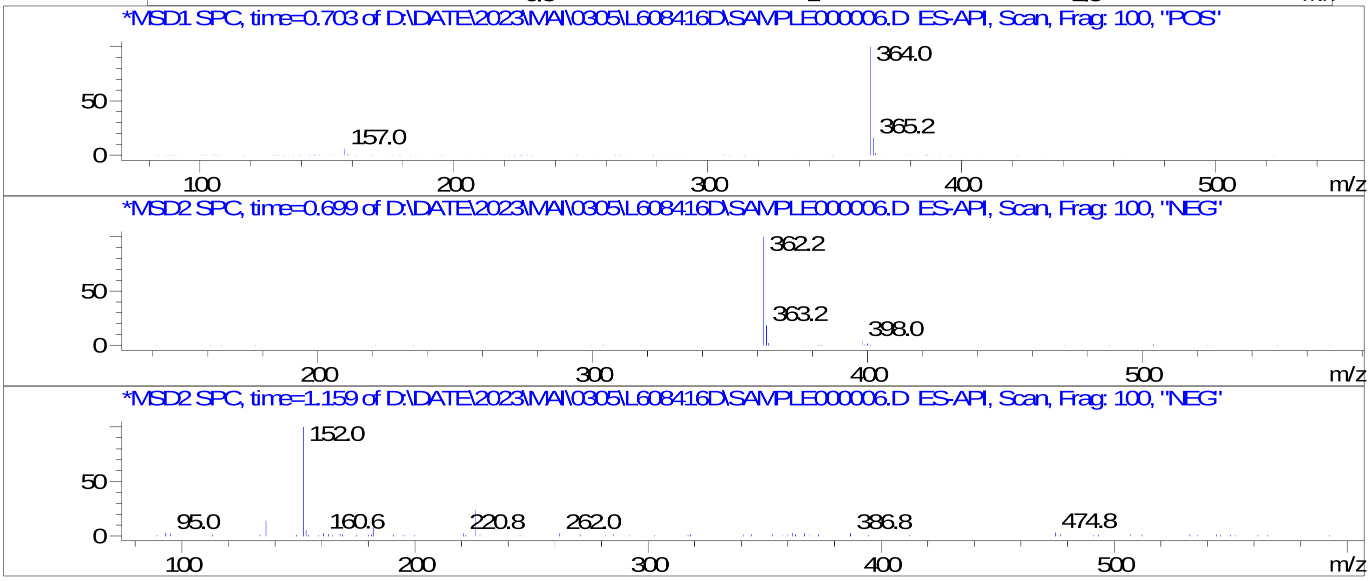

Inj.Date 5/3/2023 AN P2-A-05 - 4 - Acq. Method C:\CHEM32\-> ->
