# Supplementary material for: An artificial intelligence accelerated virtual screening platform for drug discovery
Source: Nat Commun. 2024 Sep 5;15:7761. doi: 10.1038/s41467-024-52061-7 (PMC11377542; doi:10.1038/s41467-024-52061-7)

MaxPeak: 100.00%
Ret_Time: 1.190 min

BA005633$3

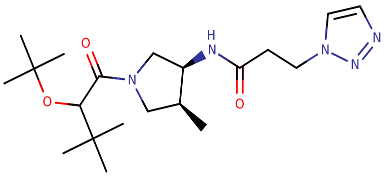

Mol Wt 393.52

Exact Mass 393.32

#	Time	Area%
1	1.190	100.00

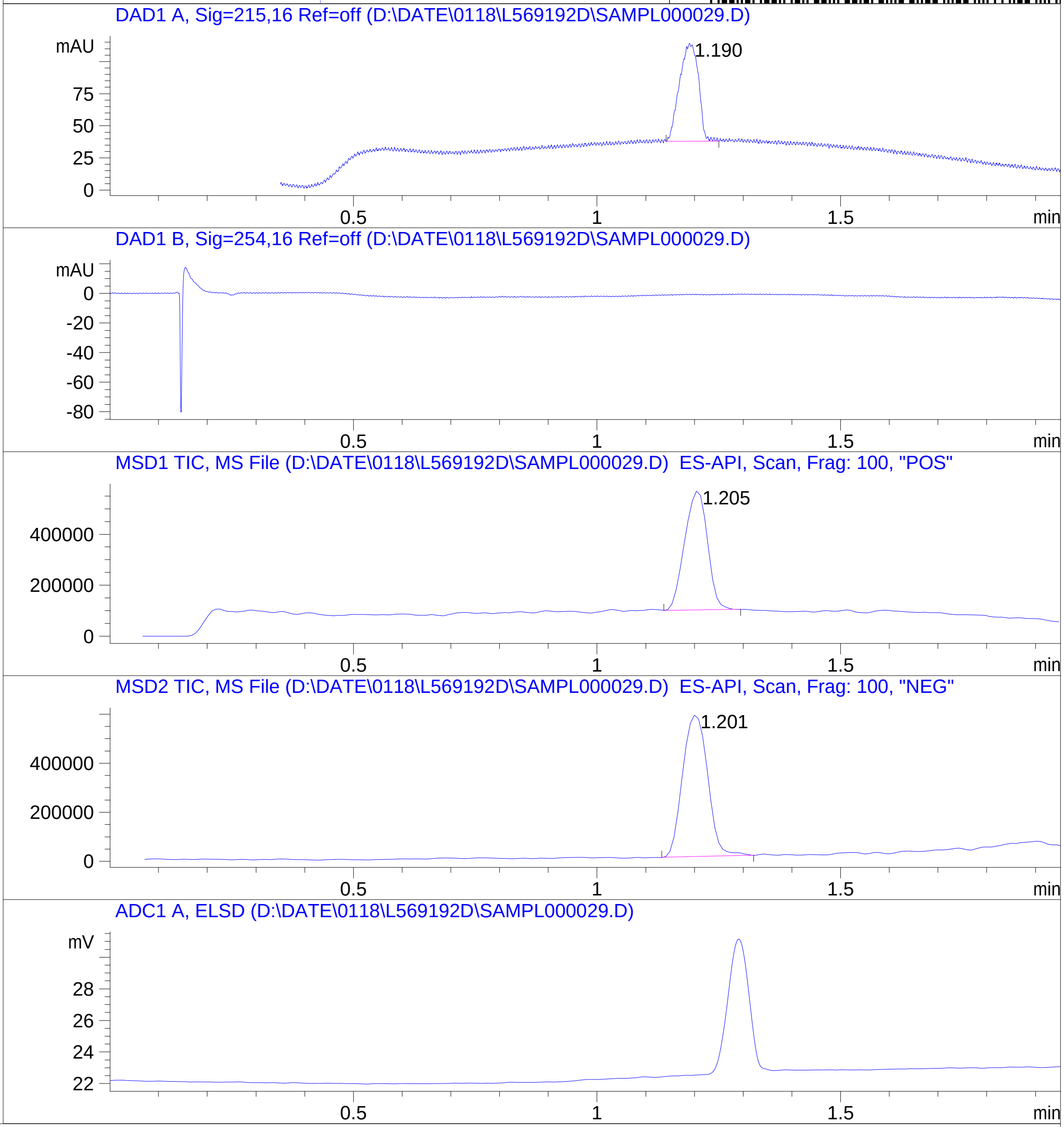

RT 1.205

RT 1.201

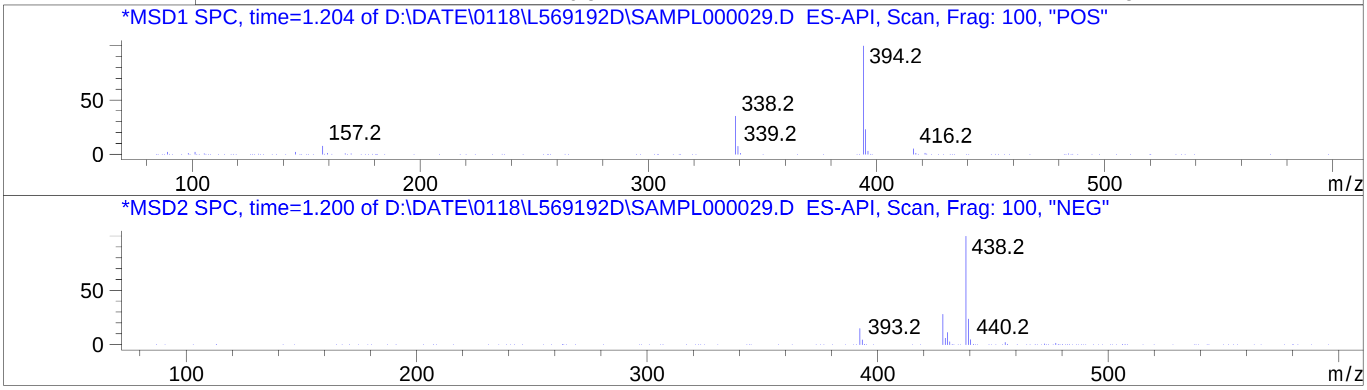

Supplement: Supplementary file 6 — Supplementary Data 3 [file 41467_2024_52061_MOESM6_ESM.zip › LC-MS-spectra/KLHDC2/Z7881785713.PDF]
