# Supplementary material for: An artificial intelligence accelerated virtual screening platform for drug discovery
Source: Nat Commun. 2024 Sep 5;15:7761. doi: 10.1038/s41467-024-52061-7 (PMC11377542; doi:10.1038/s41467-024-52061-7)
Supplement: Supplementary file 6 — Supplementary Data 3 [file 41467_2024_52061_MOESM6_ESM.zip › LC-MS-spectra/KLHDC2/Z802625304.PDF]

BA005622$2

MaxPeak: 100.00%
Ret_Time: 0.525 min

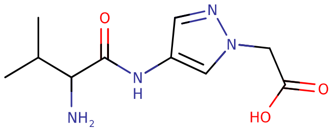

Mol Wt 240.26

Exact Mass 240.13

#	Time	Area%
1	0.525	100.00

DAD1 A, Sig=215,10 Ref=off (D:\WORK\D\01\01 19\L570268D\SAMPL047.D)

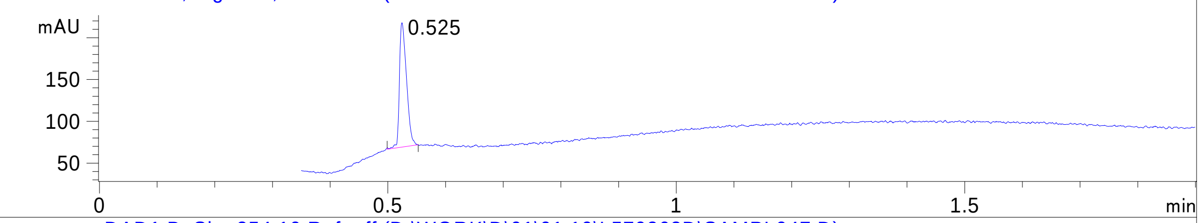

DAD1 B, Sig=254,10 Ref=off (D:\WORK\D\01\01 19\L570268D\SAMPL047.D)

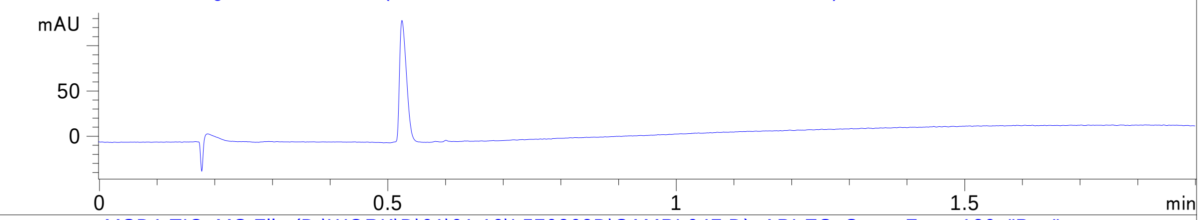

MSD1 TIC, MS File (D:\WORK\D\01\01 19\L570268D\SAMPL047.D) API-ES, Scan, Frag: 120, "Pos"

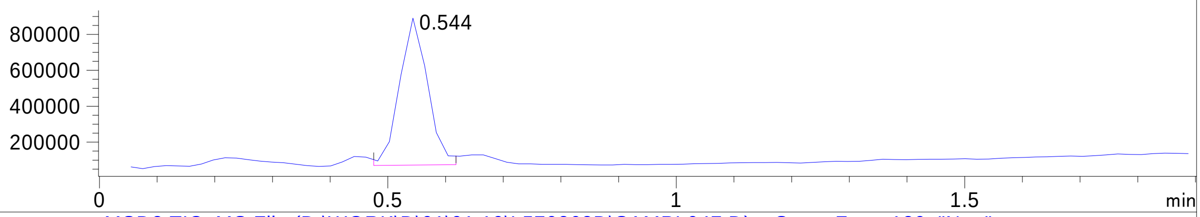

MSD2 TIC, MS File (D:\WORK\D\01\01 19\L570268D\SAMPL047.D) , Scan, Frag: 120, "Neg"

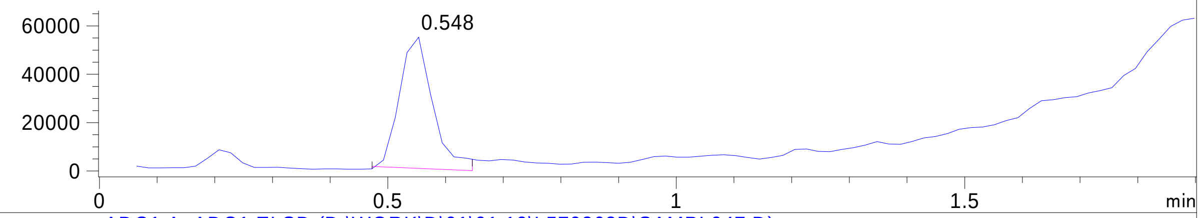

ADC1 A, ADC1 ELSD (D:\WORK\D\01\01 19\L570268D\SAMPL047.D)

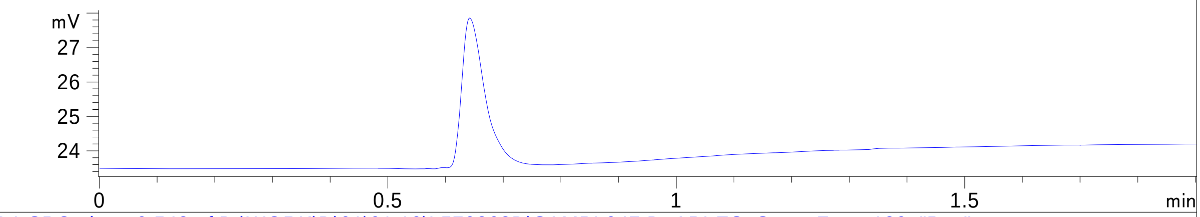

RT 0.544

*MSD1 SPC, time=0.543 of D:\WORK\D\01\01 19\L570268D\SAMPL047.D API-ES, Scan, Frag: 120, "Pos"

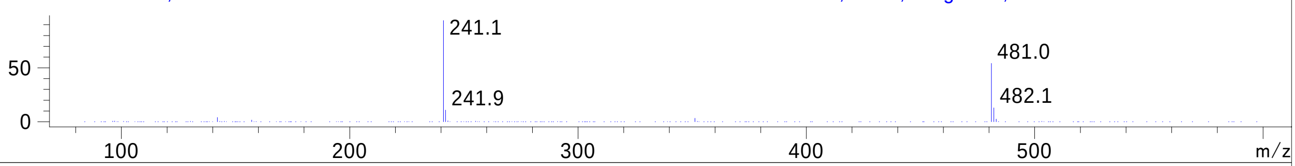

RT 0.548

*MSD2 SPC, time=0.554 of D:\WORK\D\01\01 19\L570268D\SAMPL047.D , Scan, Frag: 120, "Neg"

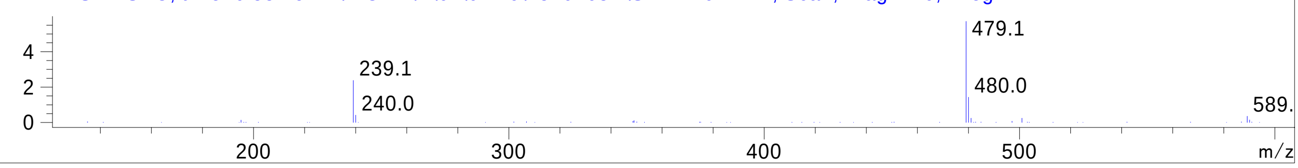

Inj.Date 1/21/2023 K P2-F-02 -VL- Acq. Method C:\HPCHEM\-> ->
