# Supplementary material for: An artificial intelligence accelerated virtual screening platform for drug discovery
Source: Nat Commun. 2024 Sep 5;15:7761. doi: 10.1038/s41467-024-52061-7 (PMC11377542; doi:10.1038/s41467-024-52061-7)
Supplement: Supplementary file 6 — Supplementary Data 3 [file 41467_2024_52061_MOESM6_ESM.zip › LC-MS-spectra/Nav1.7/Z4415557415.PDF]

BC054656$2

MaxPeak: 90.39%
Ret_Time: 0.606 min

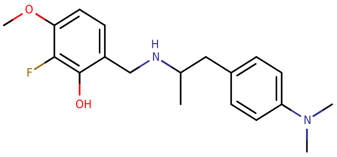

Mol Wt 332.41

Exact Mass 332.23

#	Time	Area%
1	0.606	90.39
2	0.762	4.08
3	0.821	3.89
4	0.892	1.64

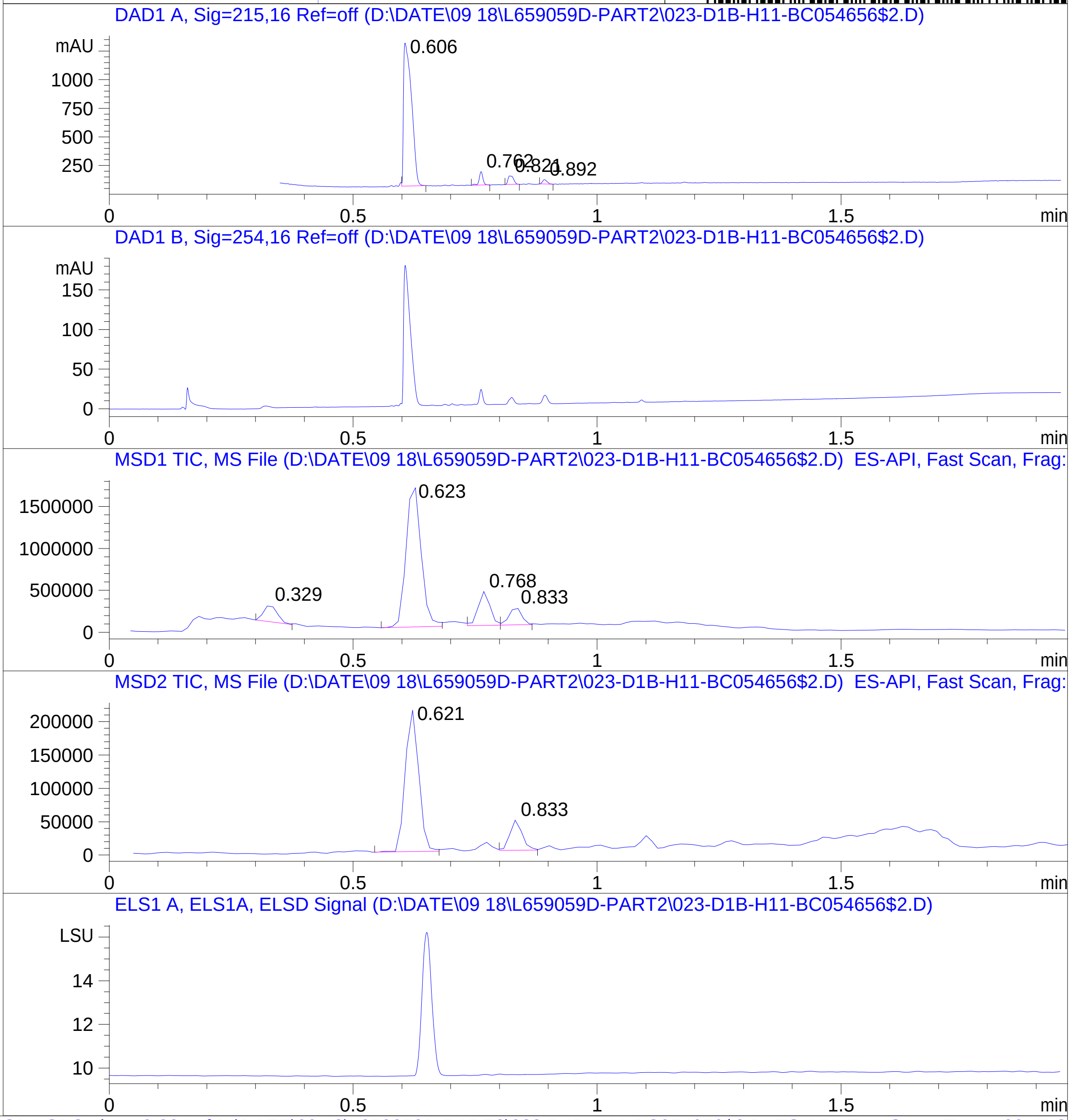

RT 0.329

RT 0.623

RT 0.768

RT 0.833

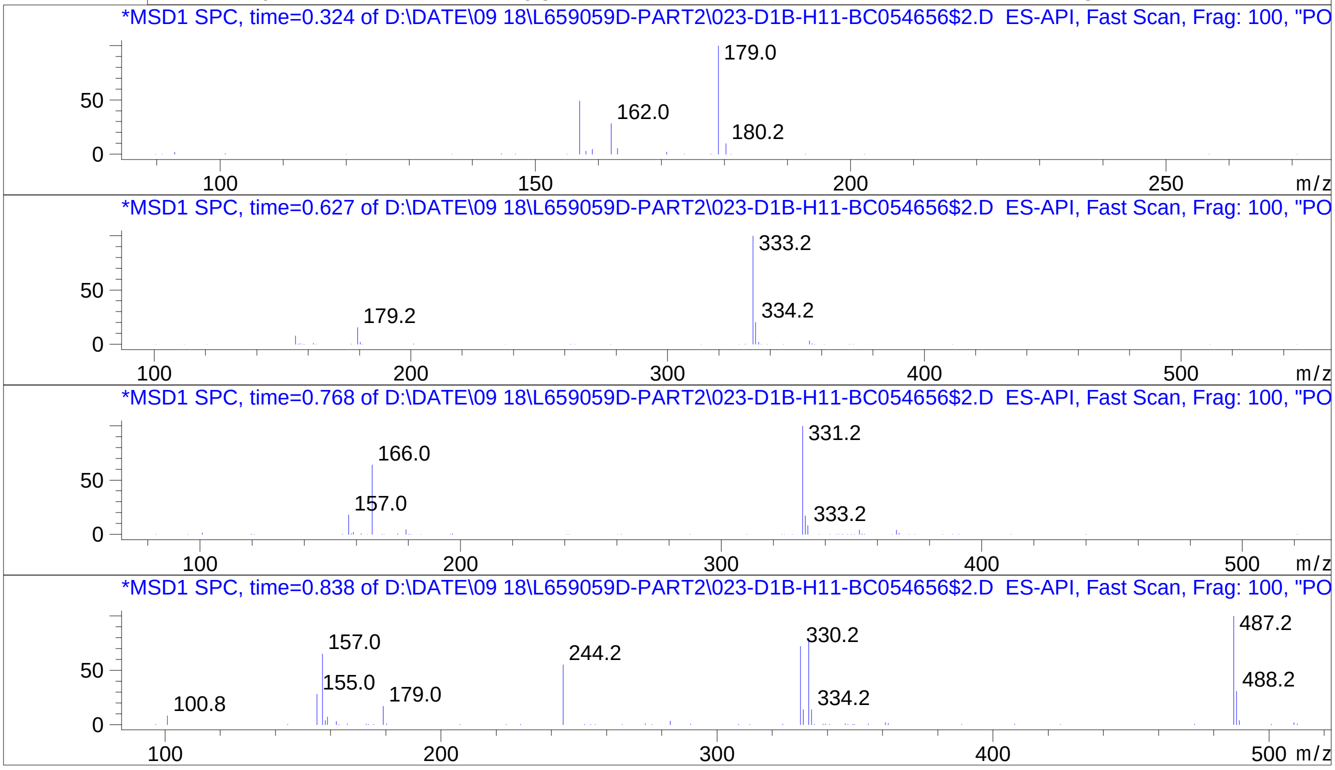

Inj.Date 9/18/2023 E Acq. Method C:\Users\ -> ->

RT 0.621

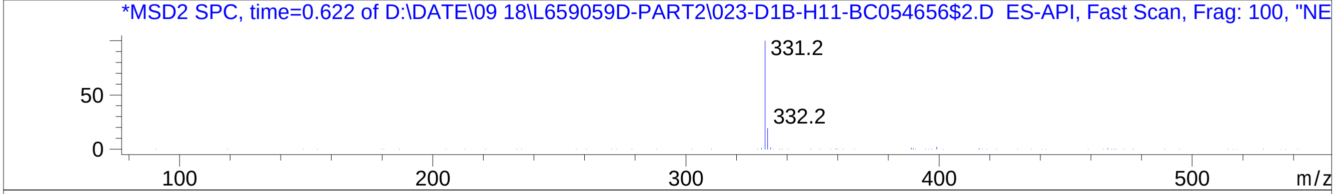

RT 0.833

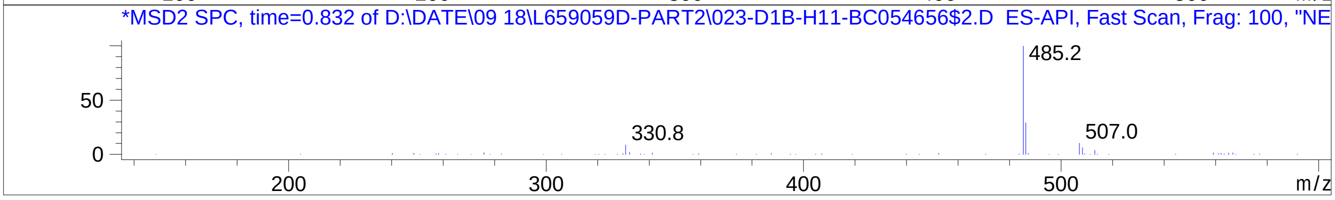

Inj.Date 9/18/2023 E Acq. Method C:\Users\ -> ->
