# Supplementary material for: An artificial intelligence accelerated virtual screening platform for drug discovery
Source: Nat Commun. 2024 Sep 5;15:7761. doi: 10.1038/s41467-024-52061-7 (PMC11377542; doi:10.1038/s41467-024-52061-7)

BC030623$22

MaxPeak: 96.99%
Ret_Time: 0.517 min

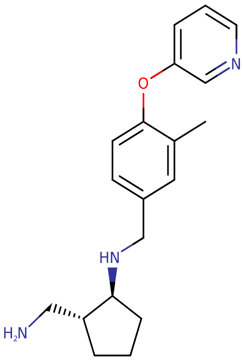

Mol Wt 311.42

Exact Mass 311.24

#	Time	Area%
1	0.517	96.99
2	0.719	1.02
3	0.883	0.72
4	1.054	1.26

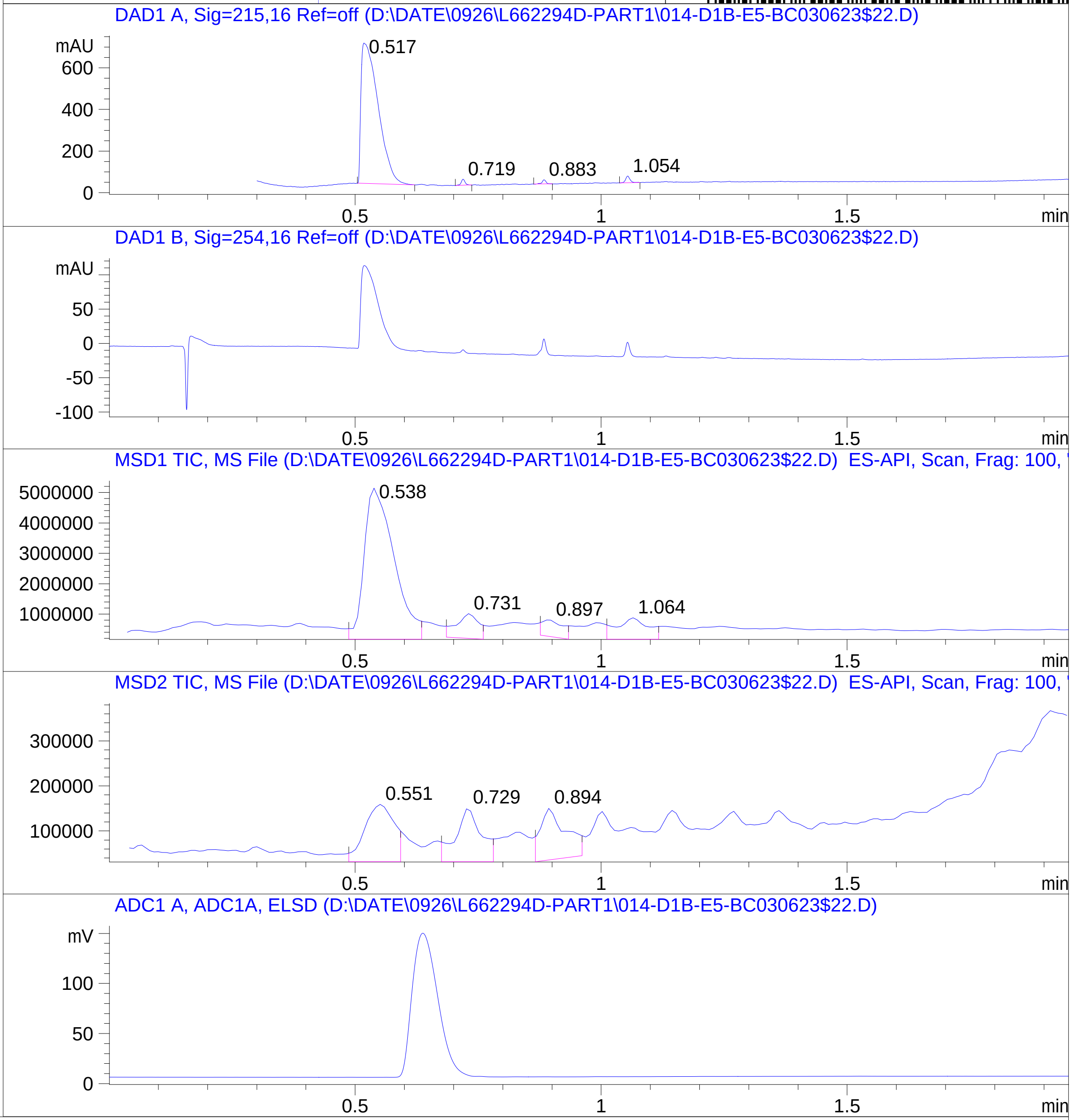

RT 0.538

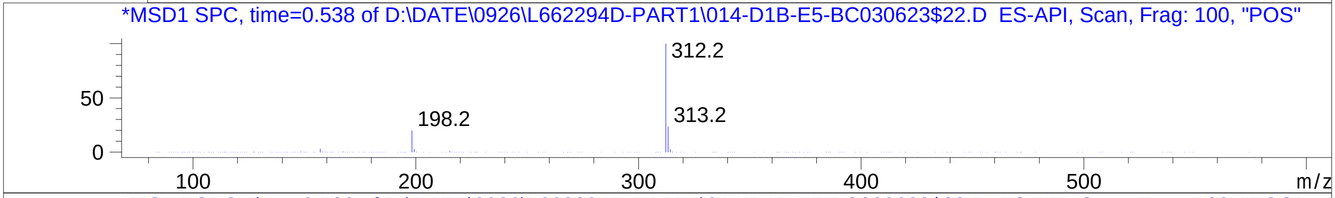

RT 0.731

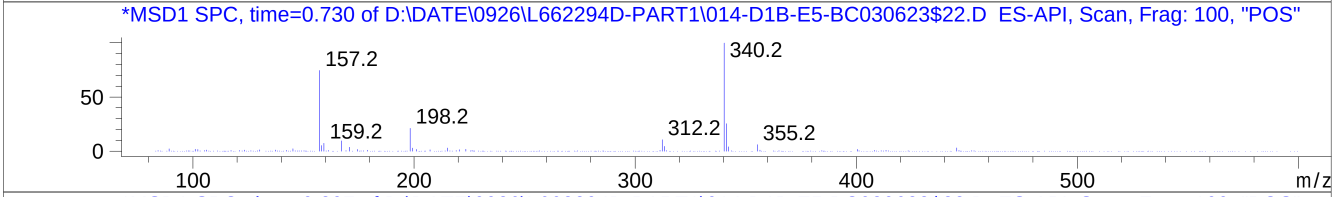

RT 0.897

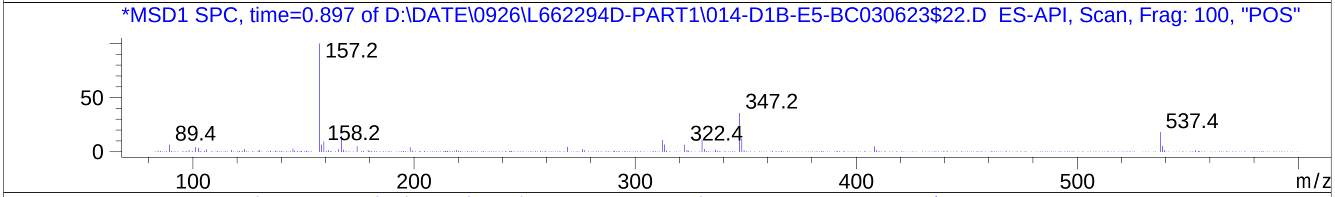

RT 1.064

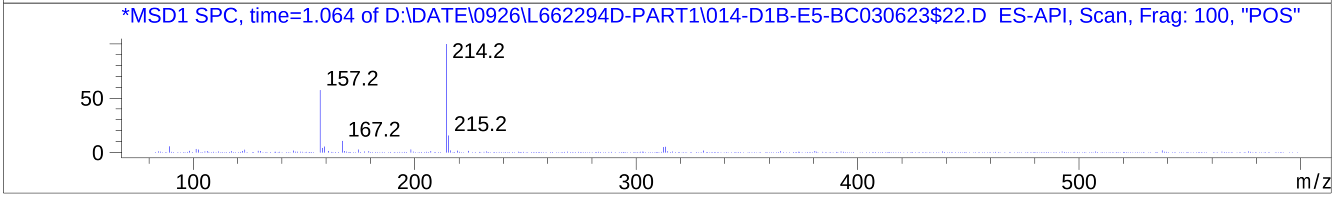

RT 0.551

RT 0.729

RT 0.894

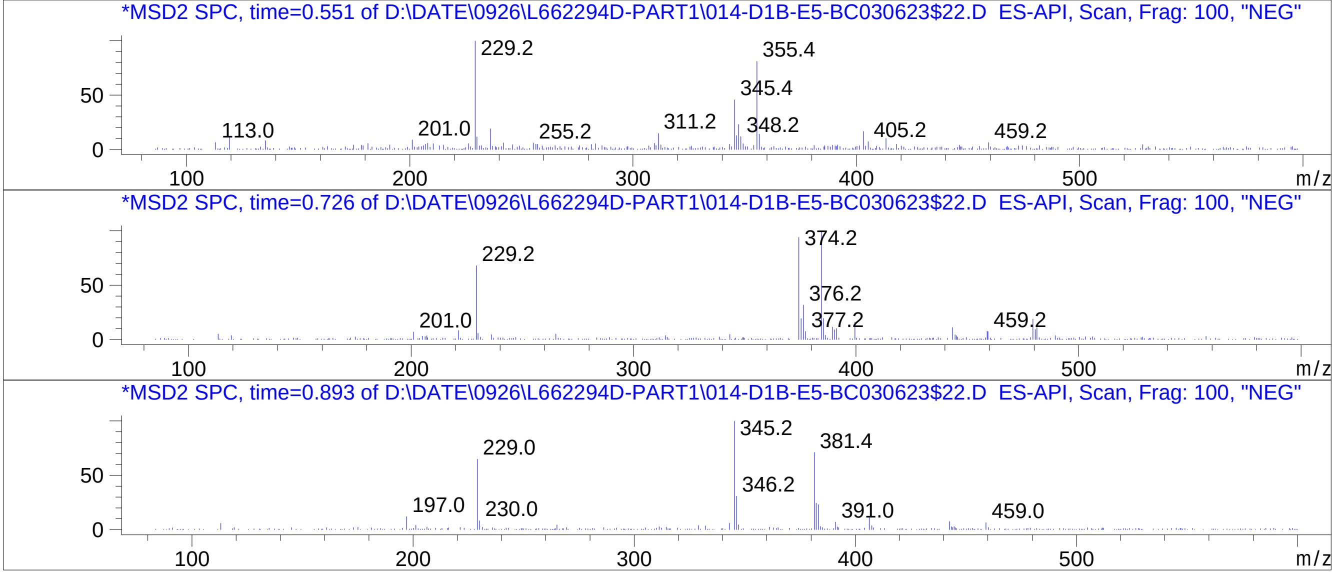

Supplement: Supplementary file 6 — Supplementary Data 3 [file 41467_2024_52061_MOESM6_ESM.zip › LC-MS-spectra/Nav1.7/Z8718594701.PDF]
