# Supplementary material for: An artificial intelligence accelerated virtual screening platform for drug discovery
Source: Nat Commun. 2024 Sep 5;15:7761. doi: 10.1038/s41467-024-52061-7 (PMC11377542; doi:10.1038/s41467-024-52061-7)

MaxPeak: 95.22%
Ret_Time: 0.807 min

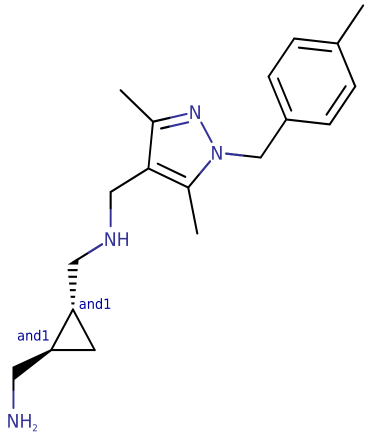

BC030627$4

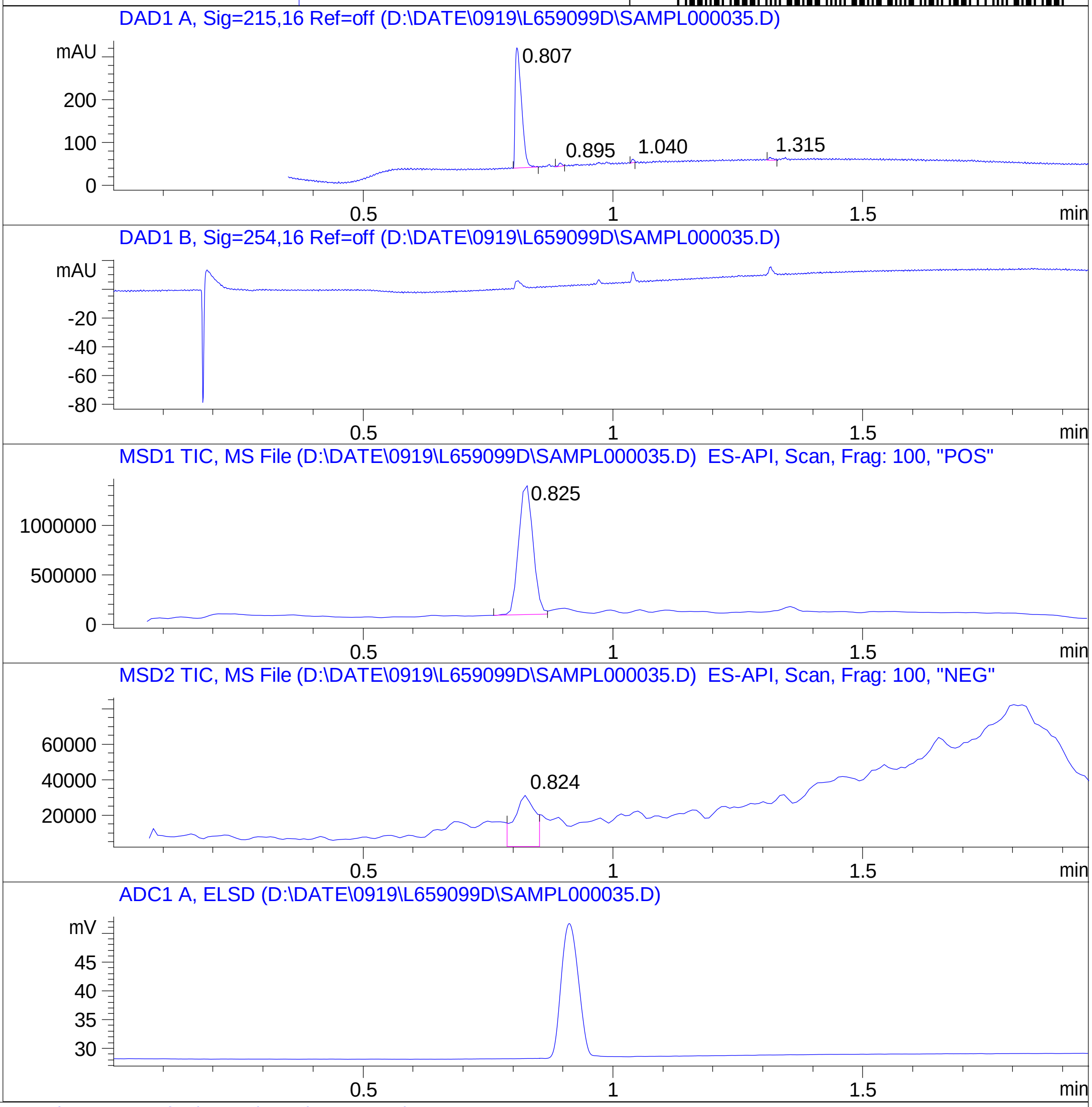

Mol Wt 312.45

Exact Mass 312.28

#	Time	Area%
1	0.807	95.22
2	0.895	1.57
3	1.040	1.30
4	1.315	1.91

RT 0.825

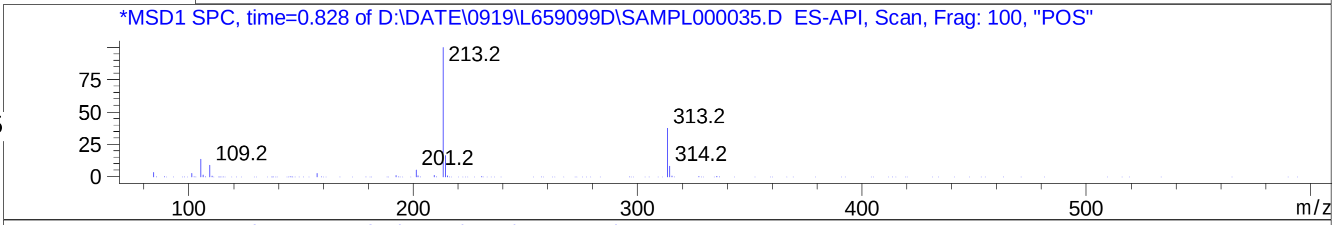

RT 0.824

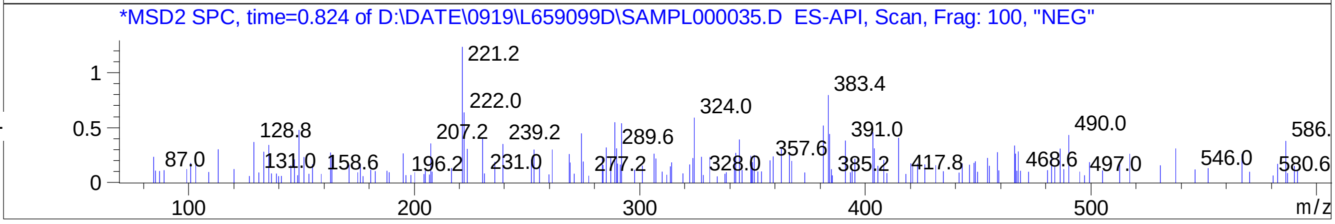

Supplement: Supplementary file 6 — Supplementary Data 3 [file 41467_2024_52061_MOESM6_ESM.zip › LC-MS-spectra/Nav1.7/Z3831472882.PDF]
