# Supplementary material for: An artificial intelligence accelerated virtual screening platform for drug discovery
Source: Nat Commun. 2024 Sep 5;15:7761. doi: 10.1038/s41467-024-52061-7 (PMC11377542; doi:10.1038/s41467-024-52061-7)
Supplement: Supplementary file 6 — Supplementary Data 3 [file 41467_2024_52061_MOESM6_ESM.zip › LC-MS-spectra/Nav1.7/Z8739905023.PDF]

BC054655$7

MaxPeak: 92.33%
Ret_Time: 1.036 min

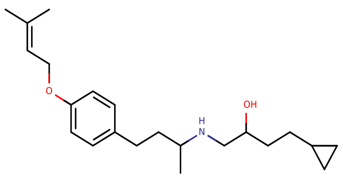

Mol Wt 345.52

Exact Mass 345.33

#	Time	Area%
1	0.991	1.44
2	1.015	2.42
3	1.036	92.33
4	1.170	3.81

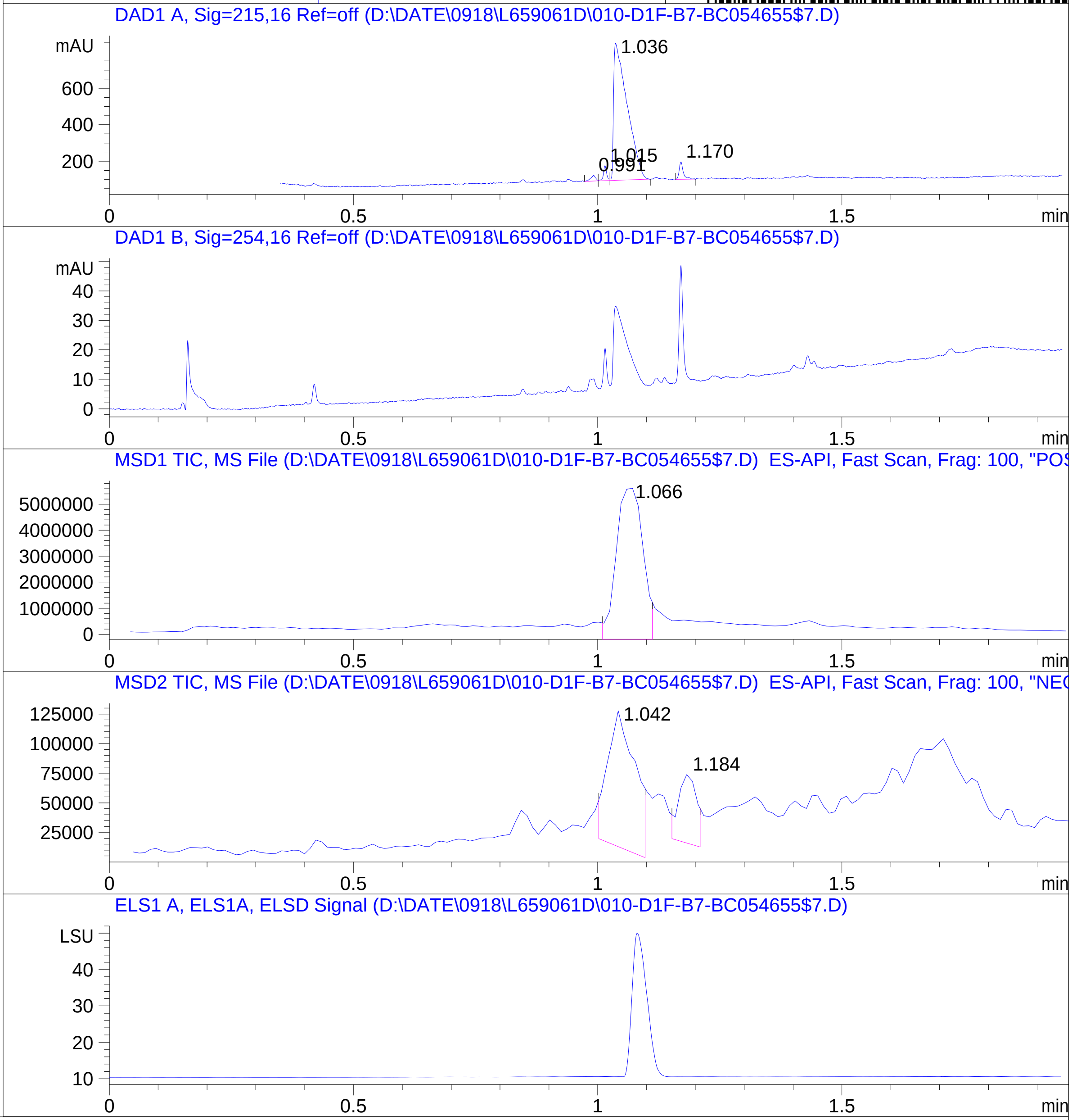

RT 1.066

RT 1.042

RT 1.184

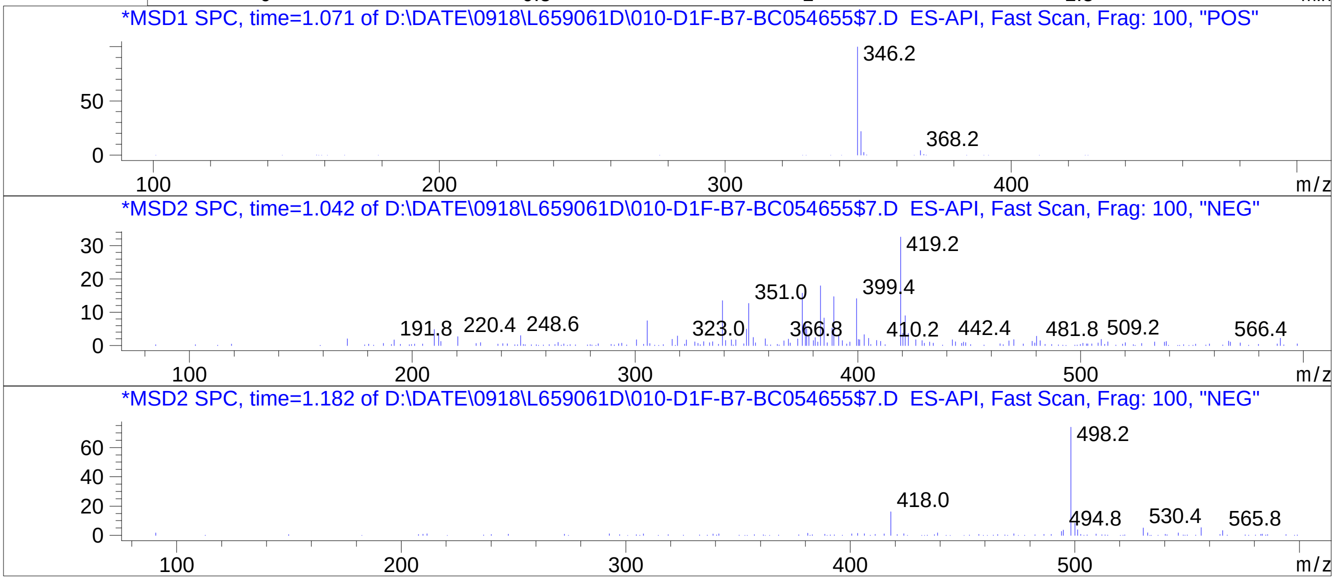

Inj.Date 9/18/2023 E Acq. Method C:\Users\ -> ->
