# Supplementary material for: An artificial intelligence accelerated virtual screening platform for drug discovery
Source: Nat Commun. 2024 Sep 5;15:7761. doi: 10.1038/s41467-024-52061-7 (PMC11377542; doi:10.1038/s41467-024-52061-7)
Supplement: Supplementary file 6 — Supplementary Data 3 [file 41467_2024_52061_MOESM6_ESM.zip › LC-MS-spectra/Nav1.7/Z4315497494.PDF]

BC030624$2

MaxPeak: 62.62%
Ret_Time: 2.118 min

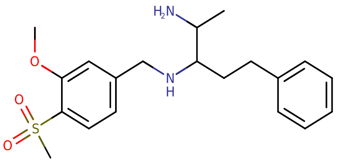

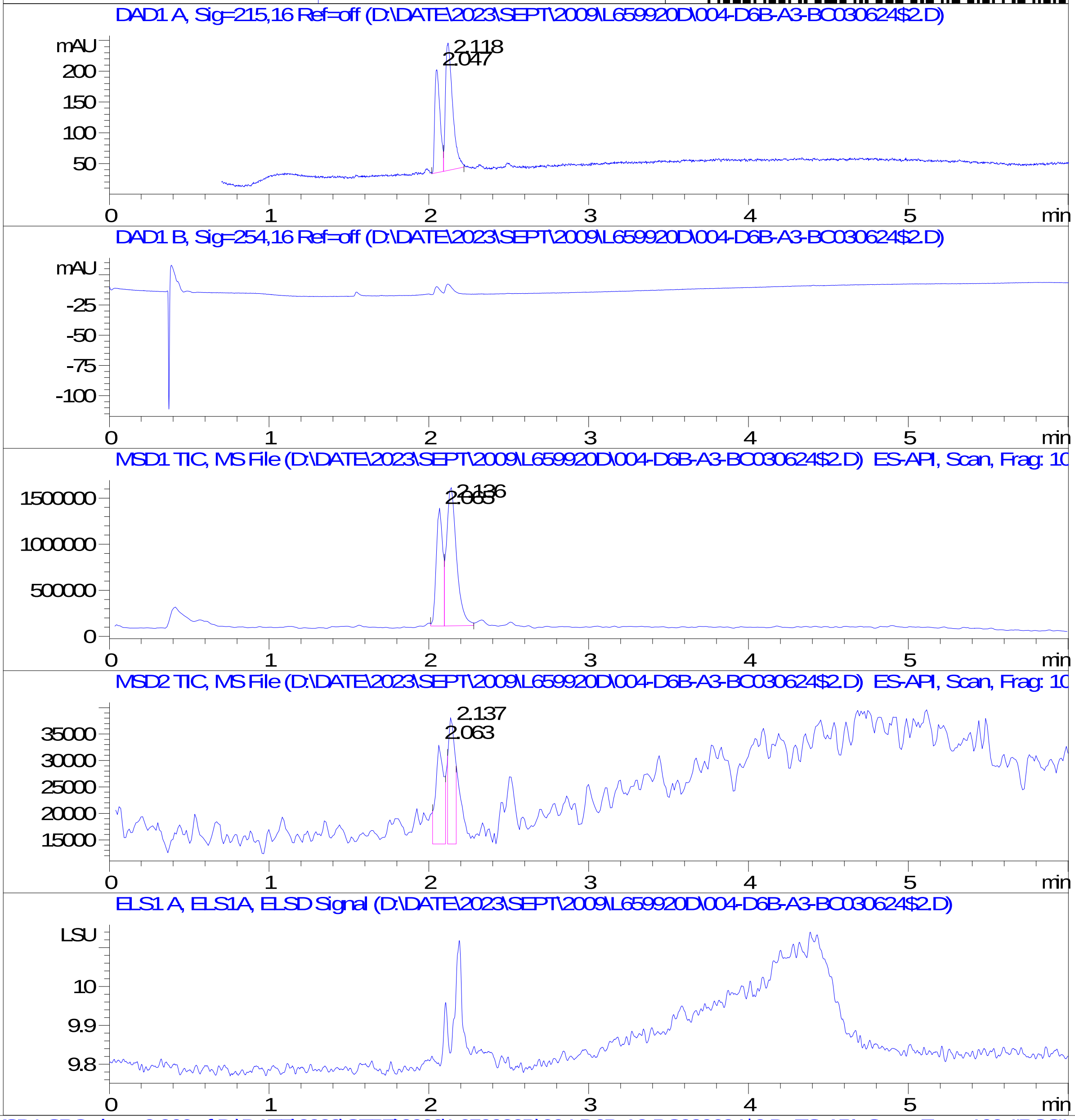

Mol Wt 376.51

Exact Mass 376.22

#	Time	Area%
1	2.047	37.38
2	2.118	62.62

RT 2.065

RT 2.136

RT 2.063

RT 2.137

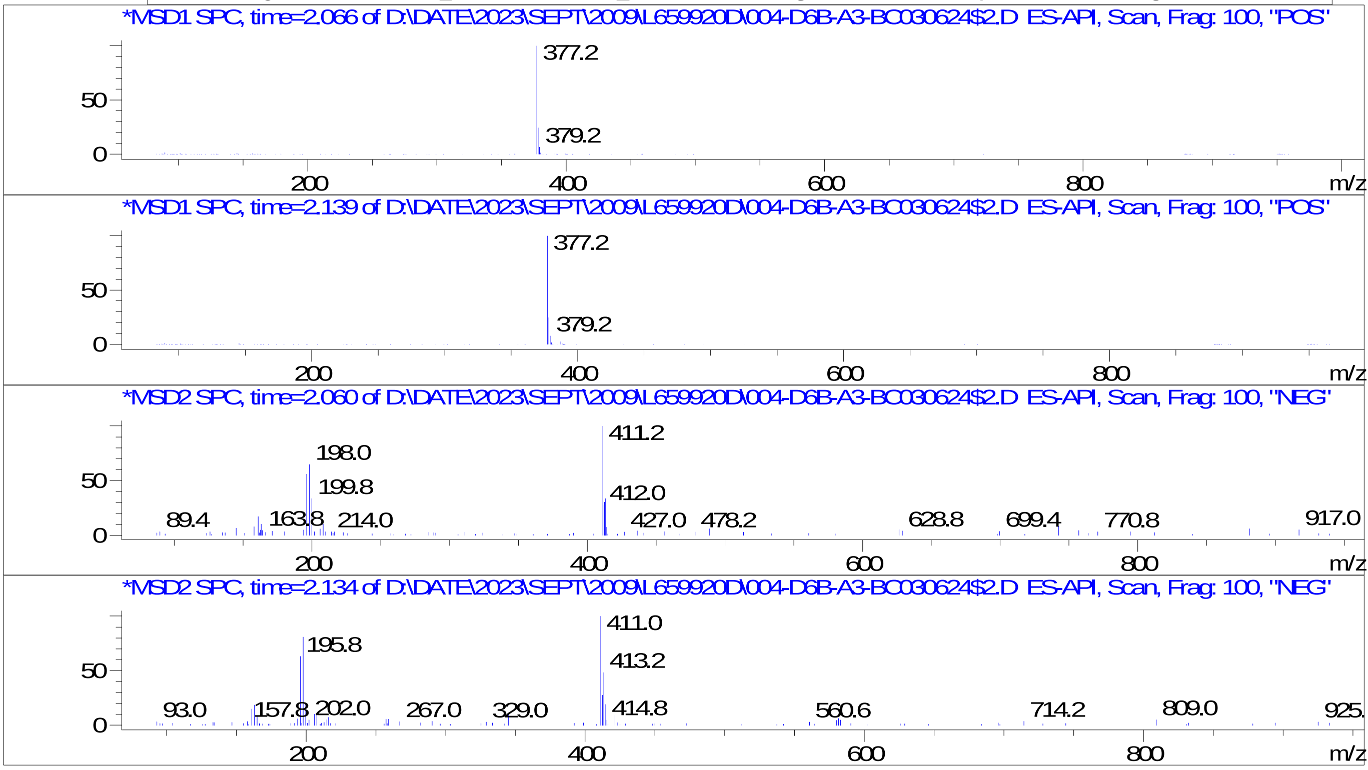

Inj.Date 9/19/2023 AN <invalid> -25- Acq. Method C:\Users\ -> ->
