# Supplementary material for: An artificial intelligence accelerated virtual screening platform for drug discovery
Source: Nat Commun. 2024 Sep 5;15:7761. doi: 10.1038/s41467-024-52061-7 (PMC11377542; doi:10.1038/s41467-024-52061-7)

BC054657$2

```
MaxPeak:  93.12%
Ret_Time: 0.846 min
```

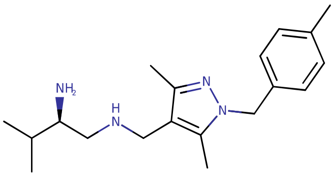

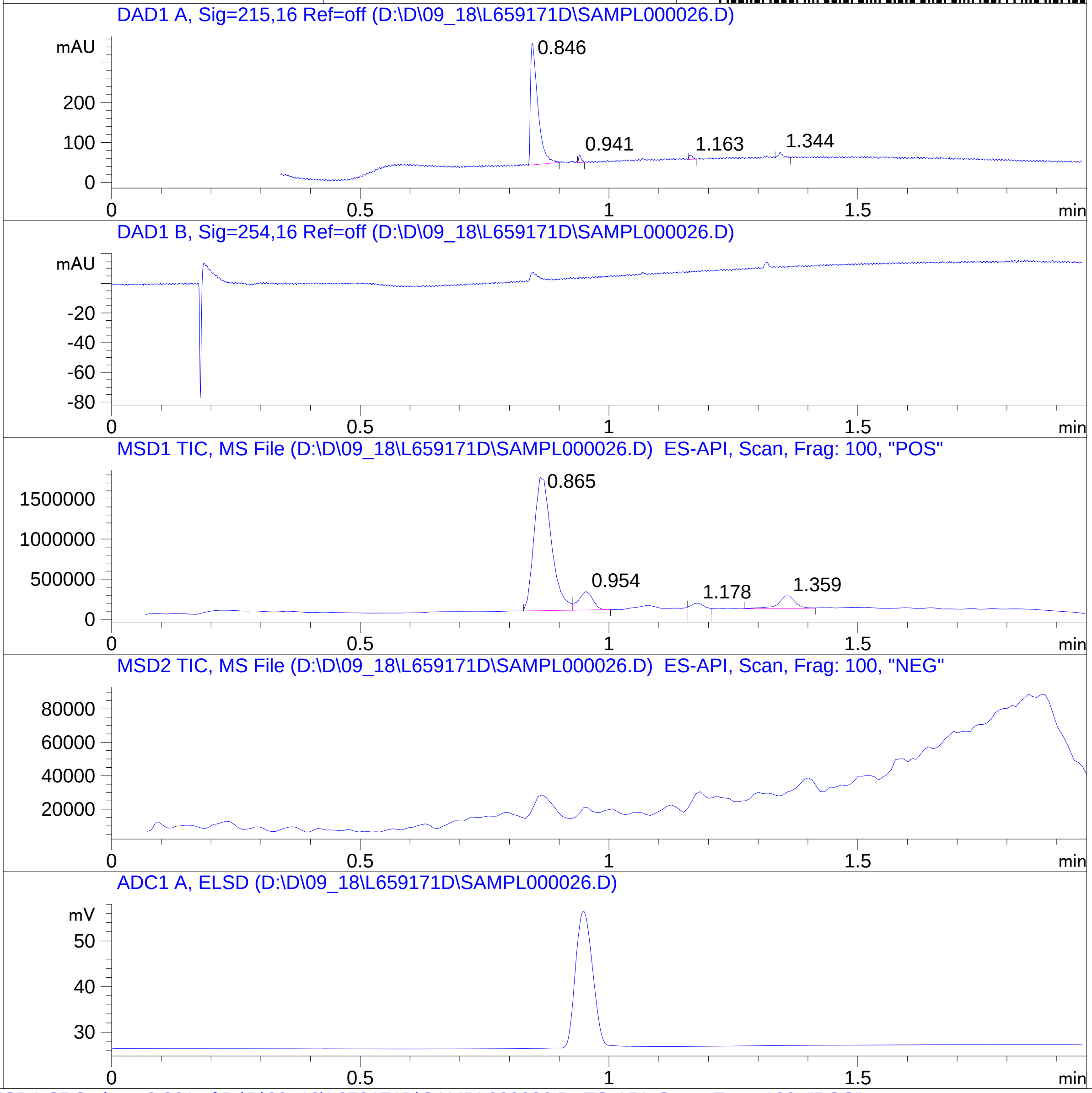

Mol Wt 314.47

Exact Mass 314.3

#	Time	Area%
1	0.846	93.12
2	0.941	2.28
3	1.163	1.64
4	1.344	2.97

RT 0.865

RT 0.954

RT 1.178

RT 1.359

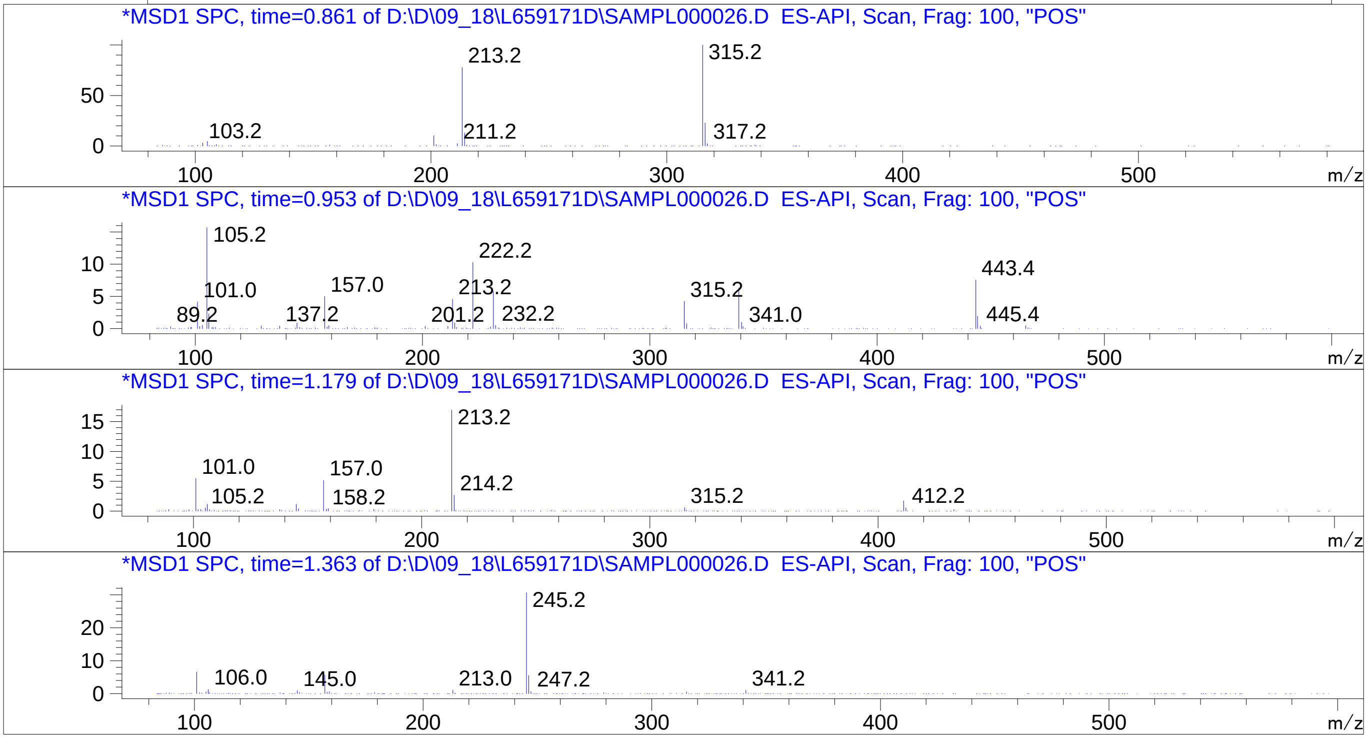

Supplement: Supplementary file 6 — Supplementary Data 3 [file 41467_2024_52061_MOESM6_ESM.zip › LC-MS-spectra/Nav1.7/Z8739902231.PDF]
