# Supplementary material for: An artificial intelligence accelerated virtual screening platform for drug discovery
Source: Nat Commun. 2024 Sep 5;15:7761. doi: 10.1038/s41467-024-52061-7 (PMC11377542; doi:10.1038/s41467-024-52061-7)
Supplement: Supplementary file 6 — Supplementary Data 3 [file 41467_2024_52061_MOESM6_ESM.zip › LC-MS-spectra/Nav1.7/Z8739902234.PDF]

BC054660$1

MaxPeak: 100.00%
Ret_Time: 1.262 min

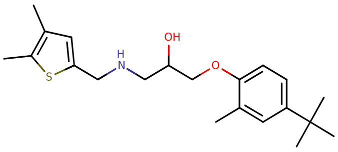

Mol Wt 361.54
Exact Mass 361.26

#	Time	Area%
1	1.262	100.00

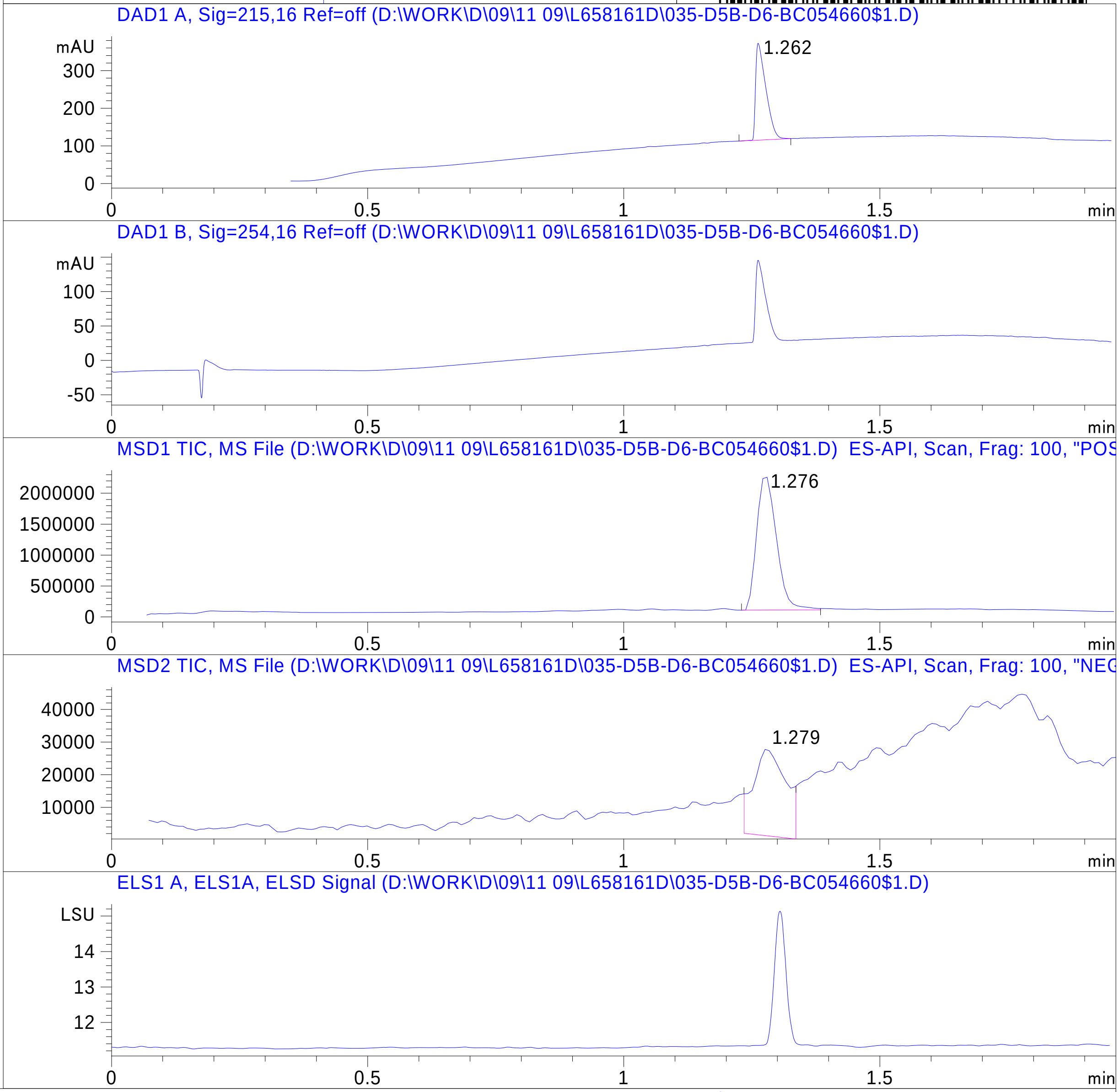

RT 1.276

RT 1.279

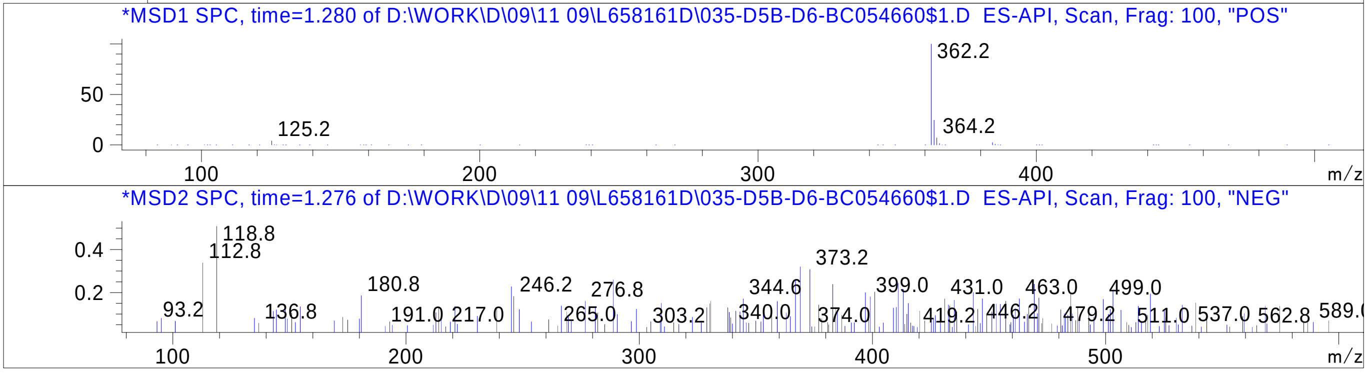

Inj.Date 9/14/2023 K <invalid> -15- Acq. Method C:\Chem32\-> ->
