# Supplementary figures and images for: An artificial intelligence accelerated virtual screening platform for drug discovery
Source: Nat Commun. 2024 Sep 5;15:7761. doi: 10.1038/s41467-024-52061-7 (PMC11377542; doi:10.1038/s41467-024-52061-7)

BA888174\$1

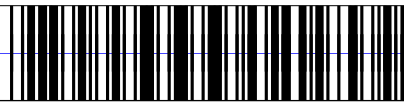

MaxPeak: 100.00%  
Ret\_Time: 1.015 min

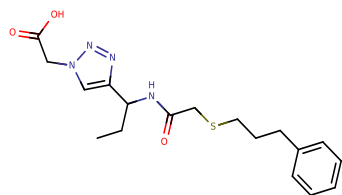

Mol Wt 376.47

Exact Mass 376.18

| # | Time  | Area%  |
|---|-------|--------|
| 1 | 1.015 | 100.00 |

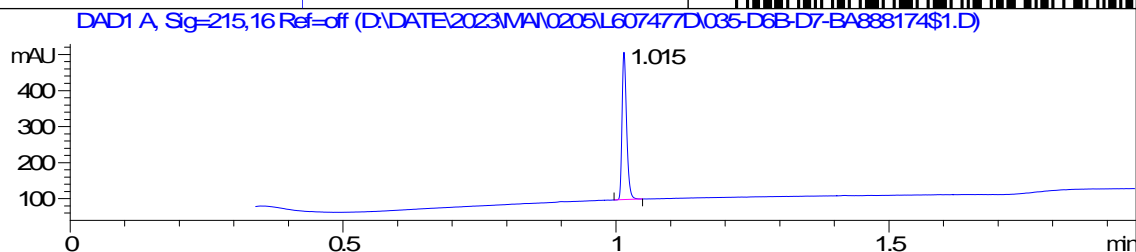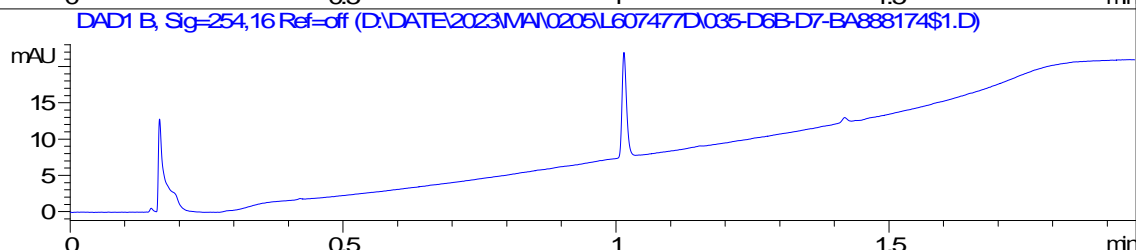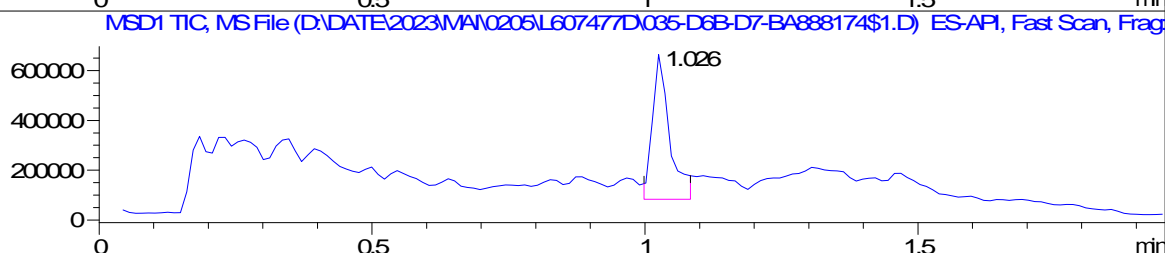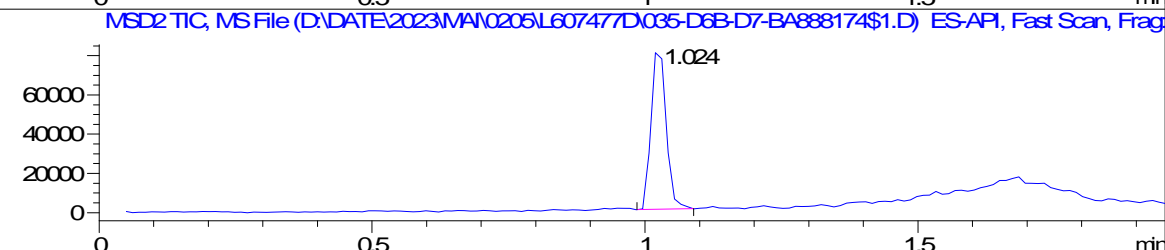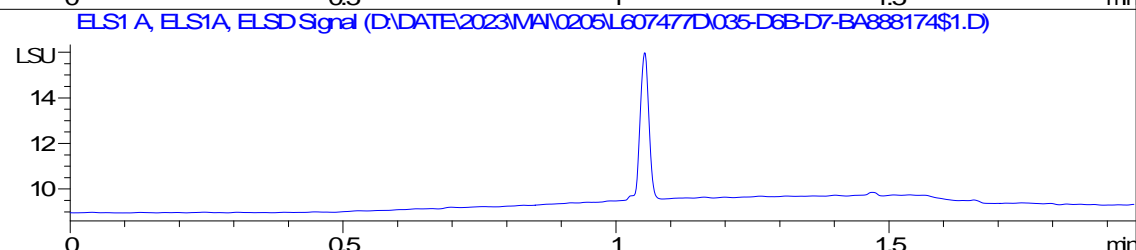

RT 1.026

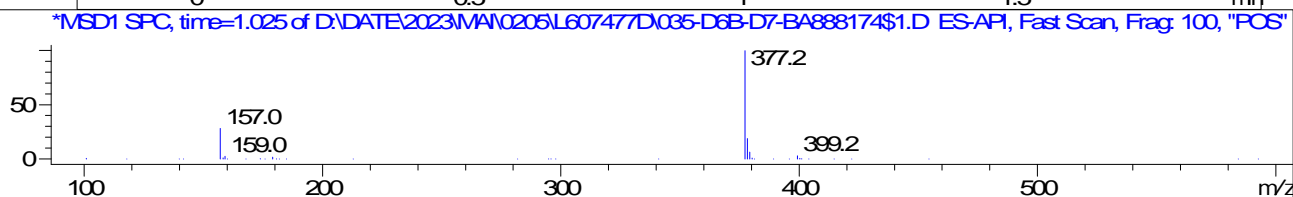

RT 1.024

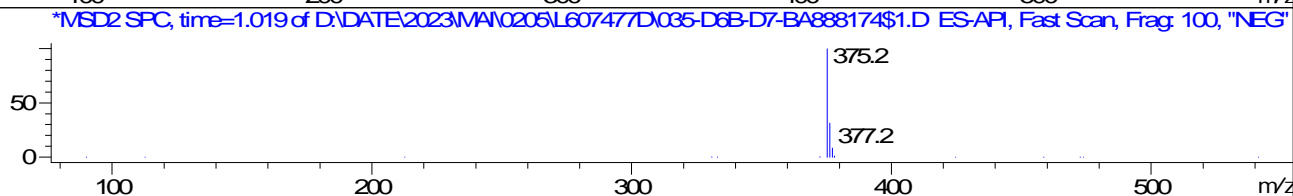

Supplement: Supplementary file 6 — Supplementary Data 3 [file 41467_2024_52061_MOESM6_ESM.zip › LC-MS-spectra/KLHDC2/Z8381047277.PDF]

BA888161\$3

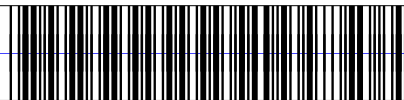

MaxPeak: 97.97%  
Ret\_Time: 1.012 min

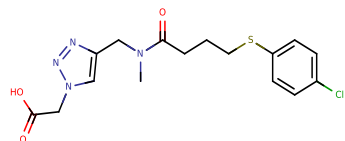

Mol Wt 382.86  
Exact Mass 382.1

| # | Time  | Area% |
|---|-------|-------|
| 1 | 1.012 | 97.97 |
| 2 | 1.125 | 2.03  |

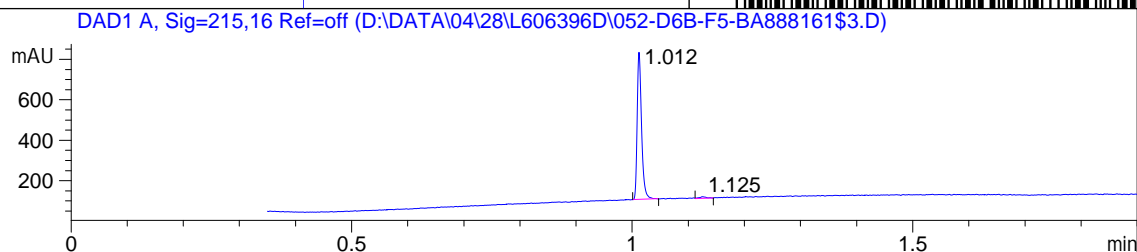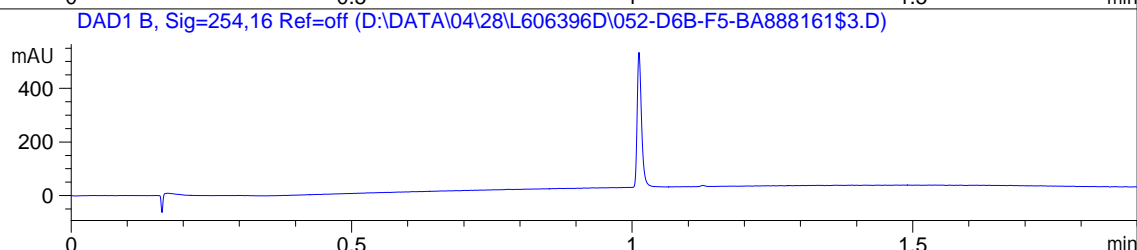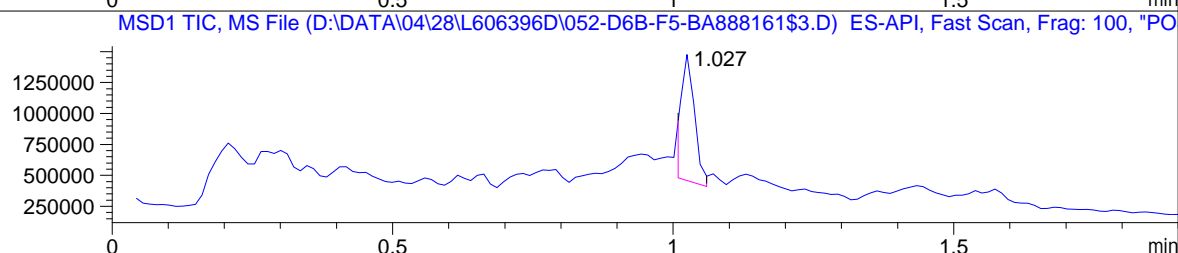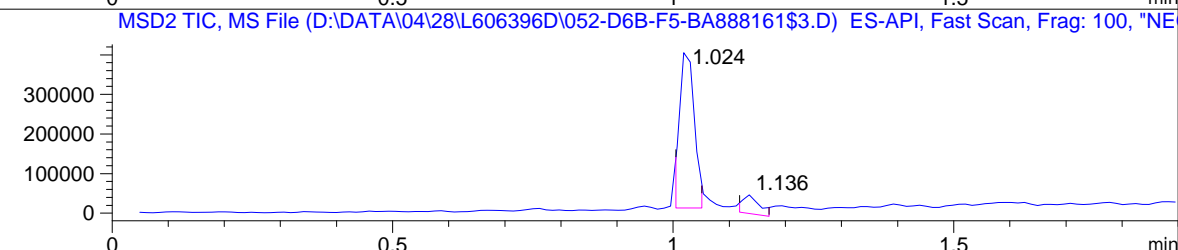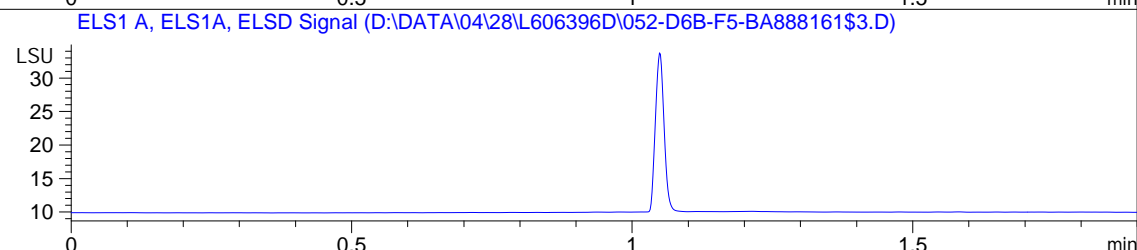

RT 1.027

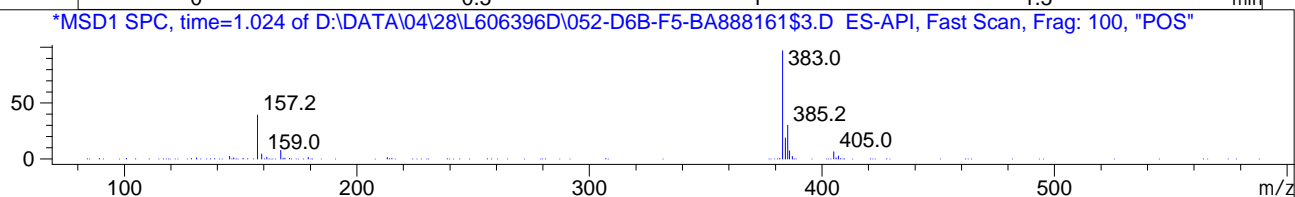

RT 1.024

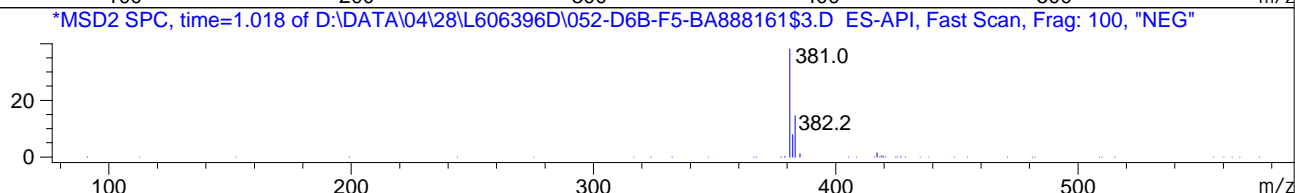

RT 1.136

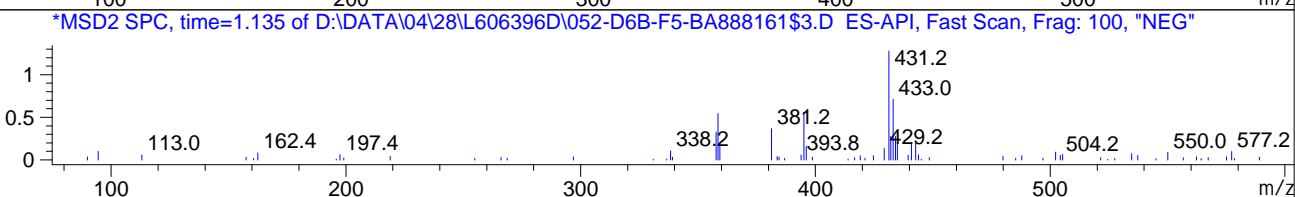

Supplement: Supplementary file 6 — Supplementary Data 3 [file 41467_2024_52061_MOESM6_ESM.zip › LC-MS-spectra/KLHDC2/Z8381047088.PDF]

MaxPeak: 100.00%  
Ret\_Time: 0.895 min

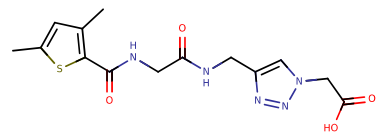

|                   |             |               |
|-------------------|-------------|---------------|
| <b>Mol Wt</b>     |             | <b>351.38</b> |
| <b>Exact Mass</b> |             | <b>351.1</b>  |
| <b>#</b>          | <b>Time</b> | <b>Area%</b>  |
| -----             |             |               |
| 1                 | 0.895       | 100.00        |

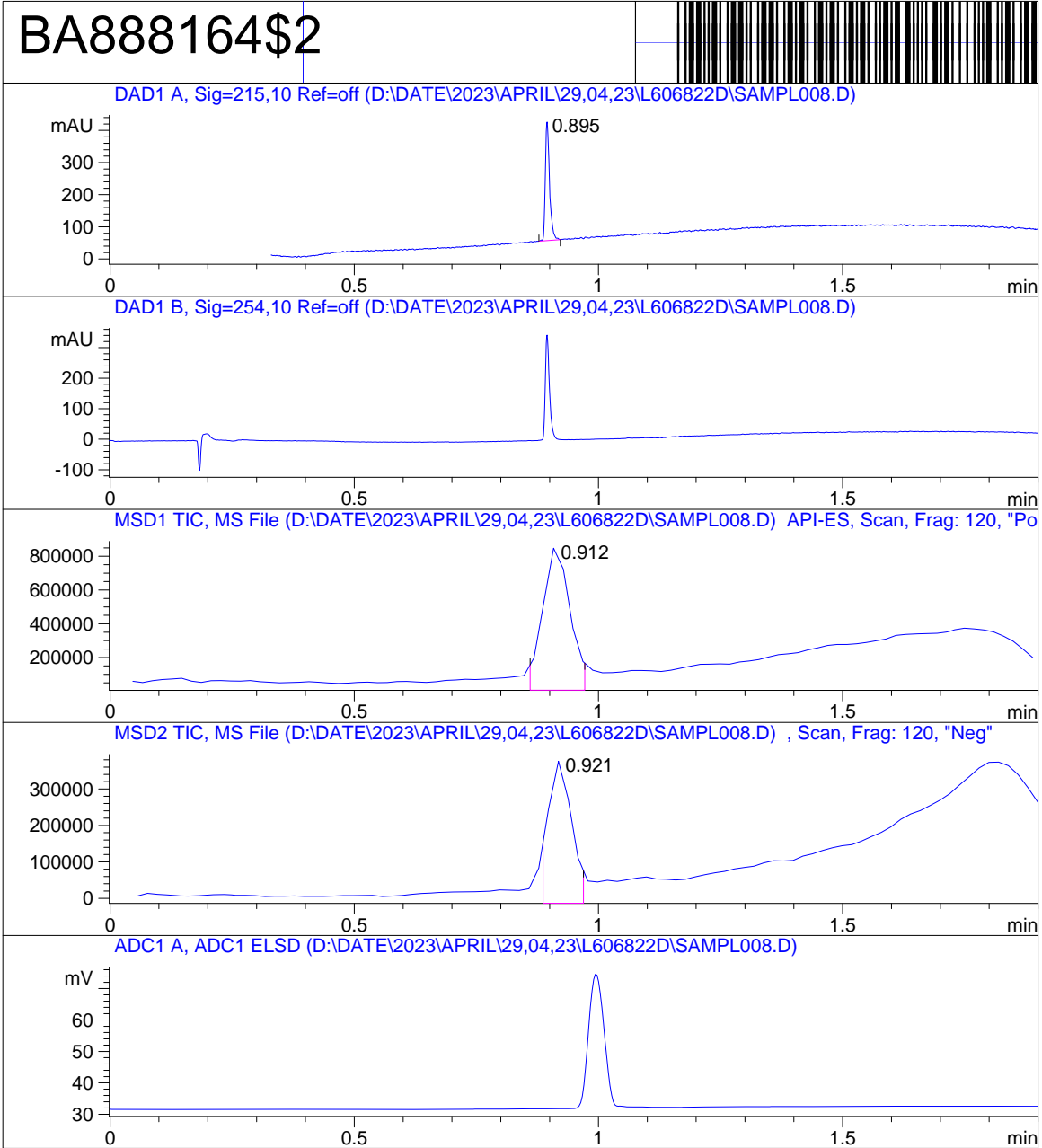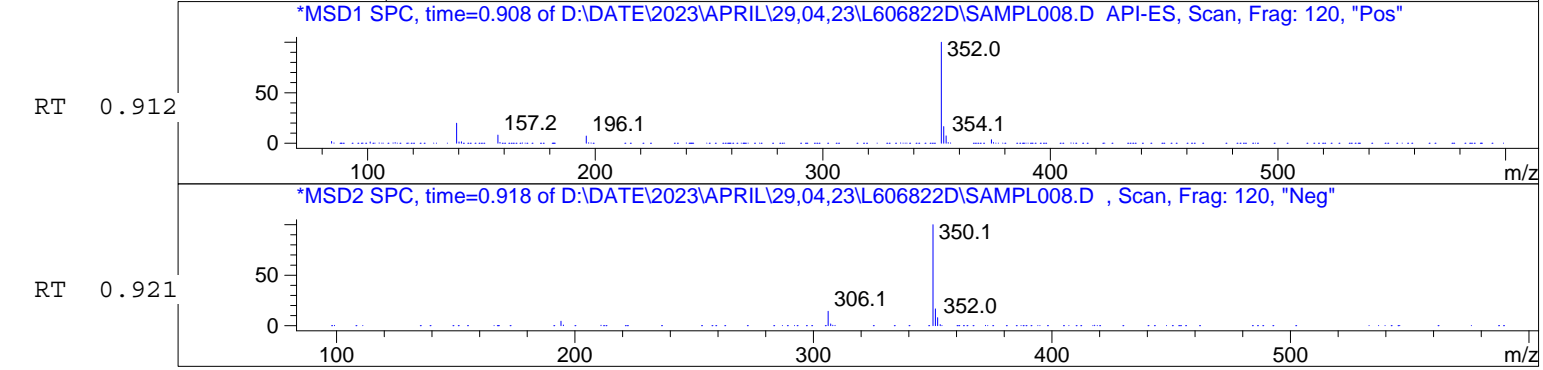

Supplement: Supplementary file 6 — Supplementary Data 3 [file 41467_2024_52061_MOESM6_ESM.zip › LC-MS-spectra/KLHDC2/Z8381047063.PDF]

BA888157\$2

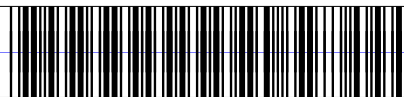

MaxPeak: 100.00%  
Ret\_Time: 1.120 min

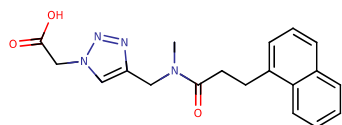

Mol Wt 352.39  
Exact Mass 352.17

| # | Time  | Area%  |
|---|-------|--------|
| 1 | 1.120 | 100.00 |

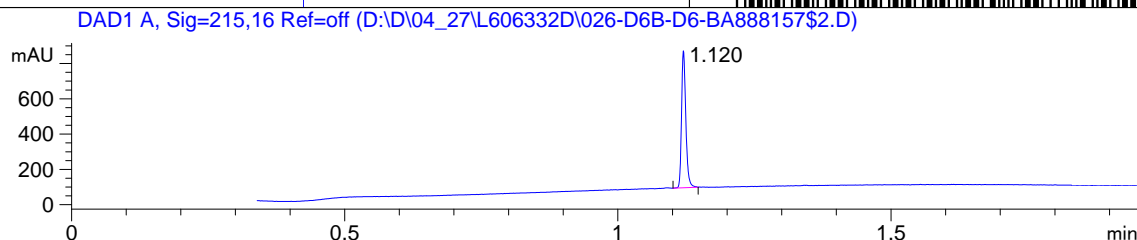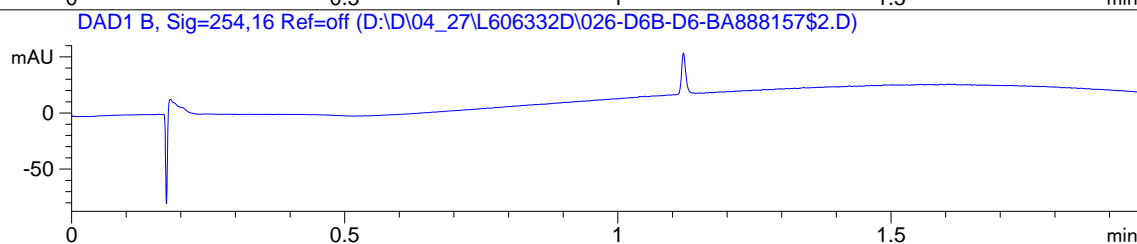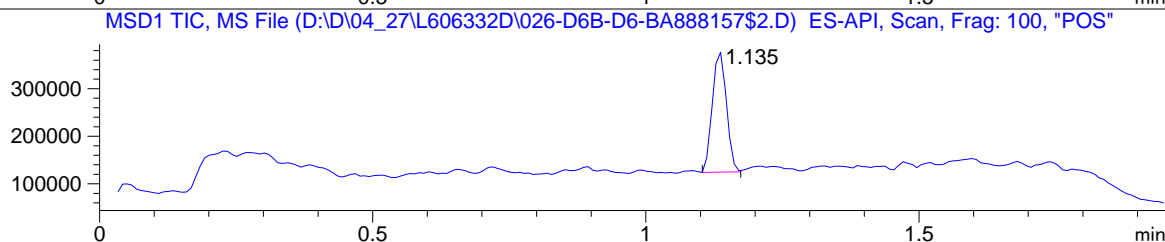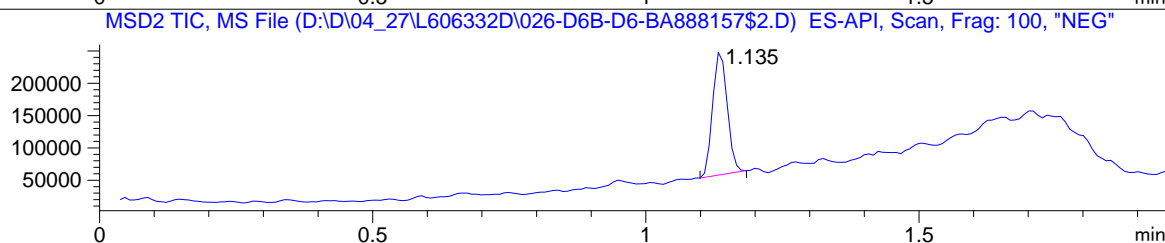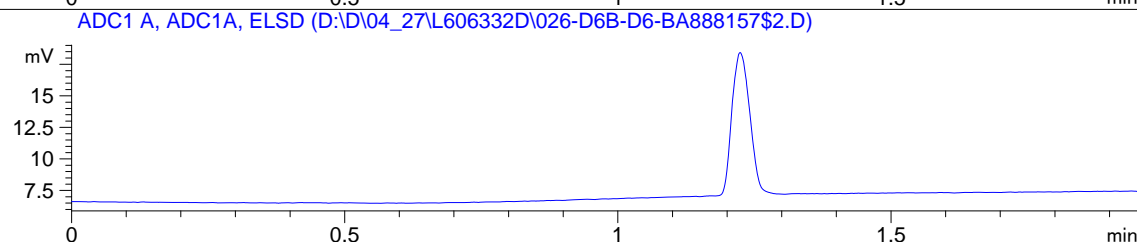

RT 1.135

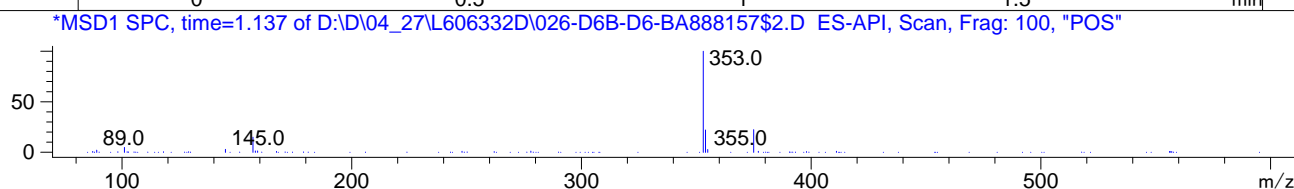

RT 1.135

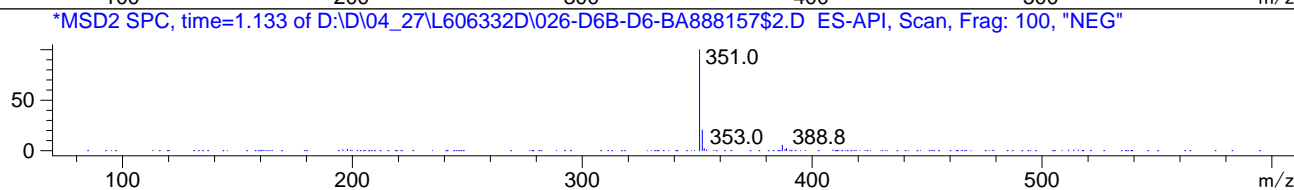

Supplement: Supplementary file 6 — Supplementary Data 3 [file 41467_2024_52061_MOESM6_ESM.zip › LC-MS-spectra/KLHDC2/Z8381047076.PDF]

BA888175\$2

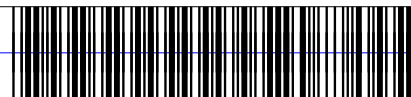

MaxPeak: 100.00%  
Ret\_Time: 1.427 min

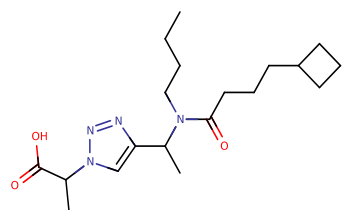

Mol Wt 364.48  
Exact Mass 364.29

| # | Time  | Area%  |
|---|-------|--------|
| 1 | 1.427 | 100.00 |

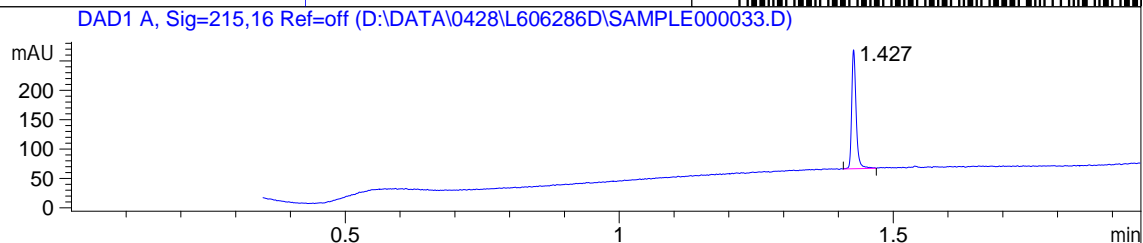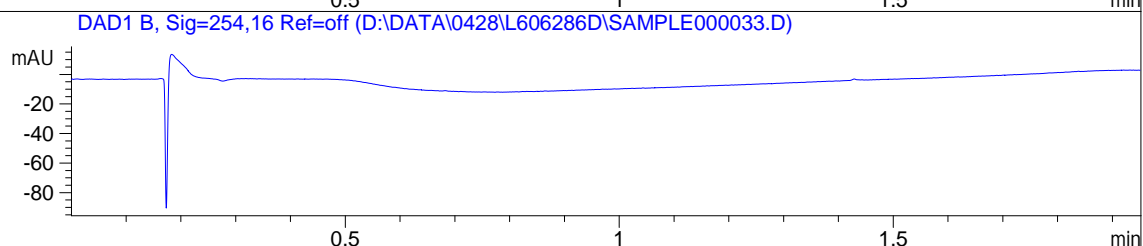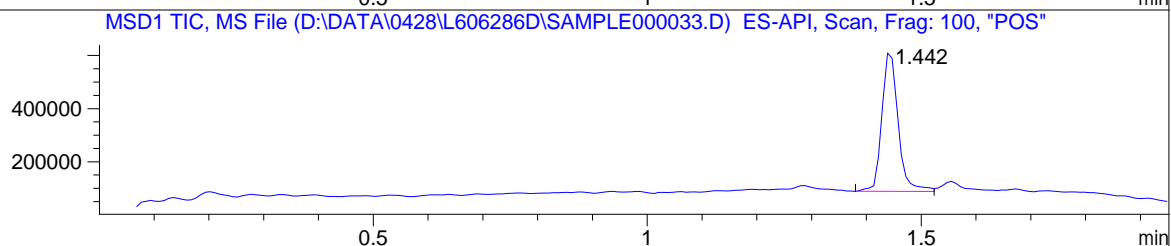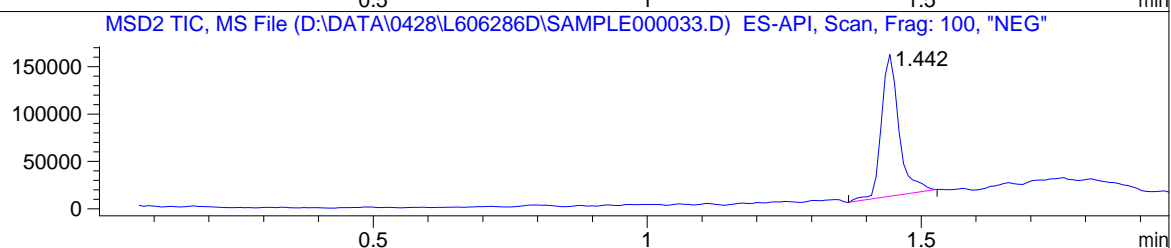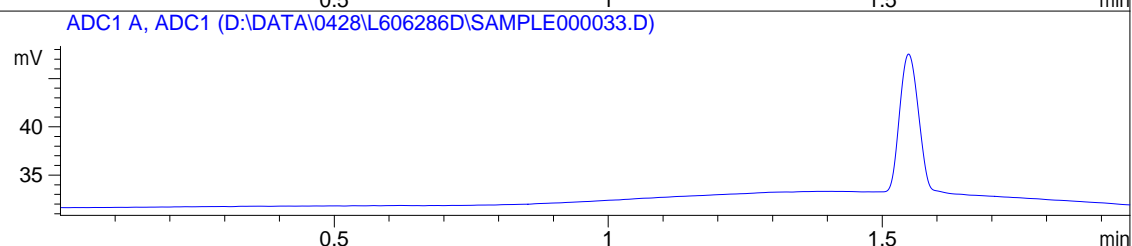

RT 1.442

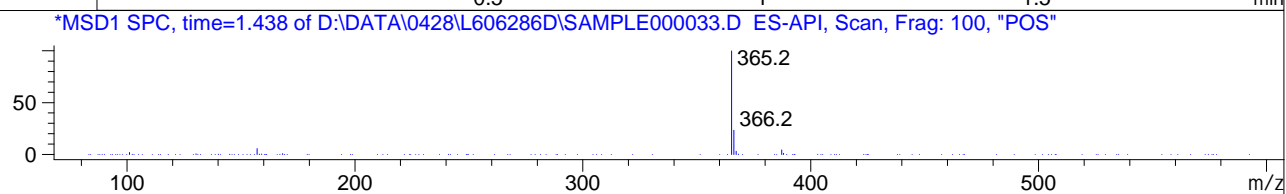

RT 1.442

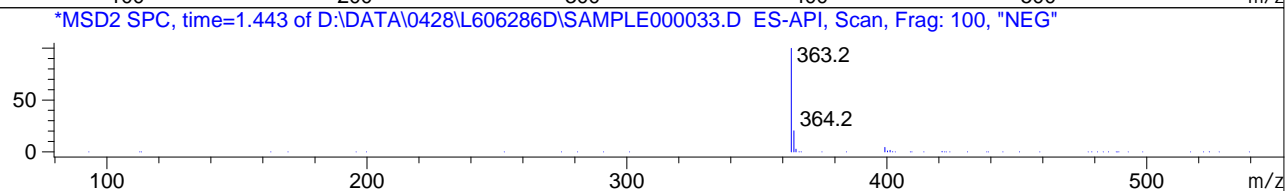

Supplement: Supplementary file 6 — Supplementary Data 3 [file 41467_2024_52061_MOESM6_ESM.zip › LC-MS-spectra/KLHDC2/Z8381047258.PDF]

MaxPeak: 100.00%  
Ret\_Time: 1.269 min

BA888167\$4

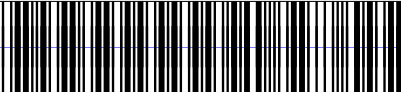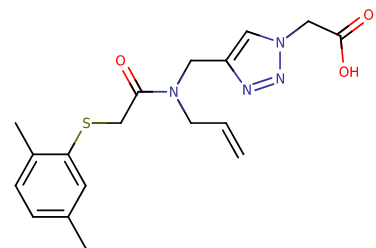

Mol Wt 374.46  
Exact Mass 374.16

| # | Time  | Area%  |
|---|-------|--------|
| 1 | 1.269 | 100.00 |

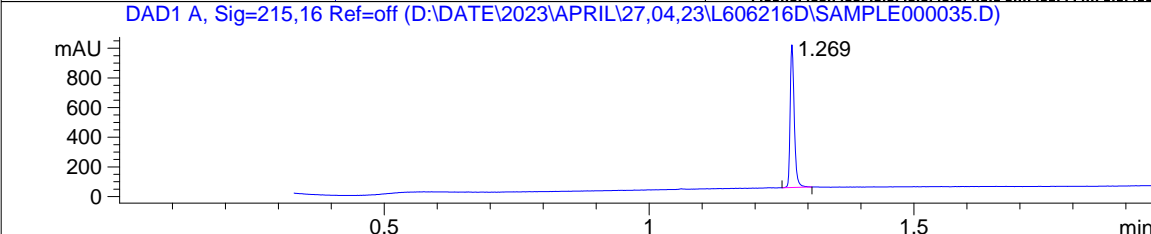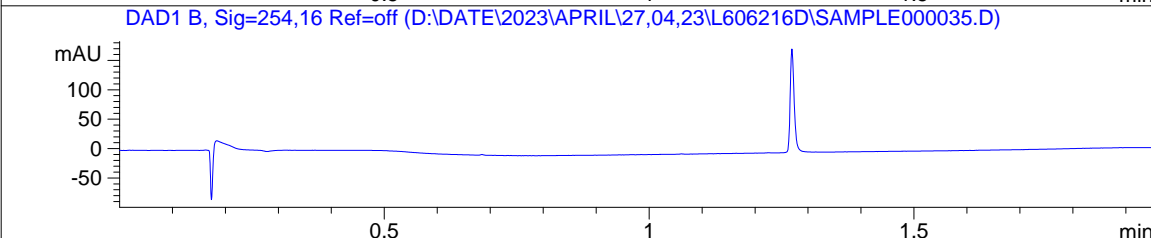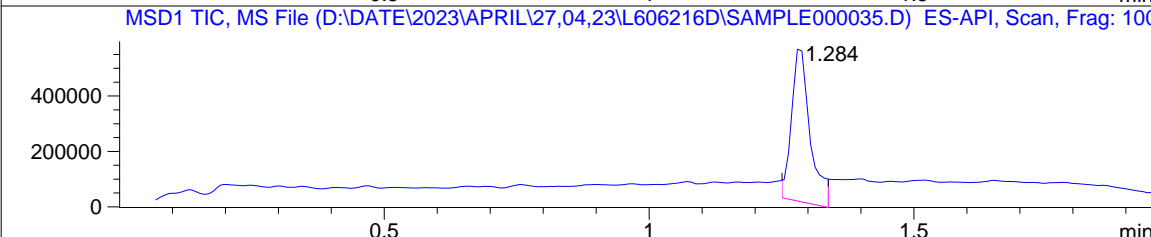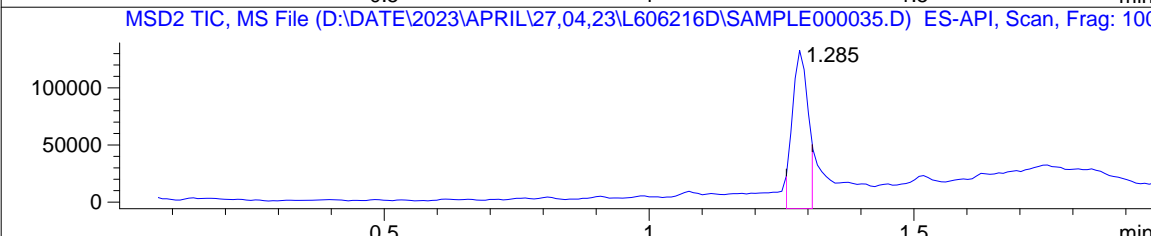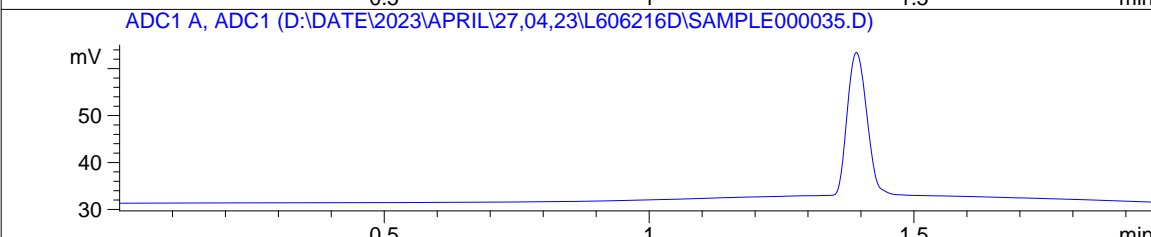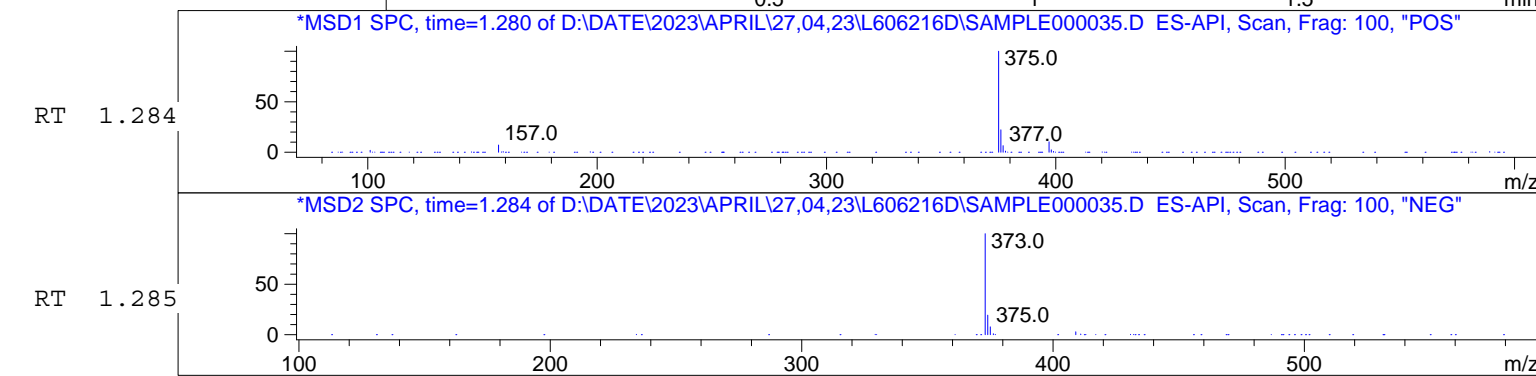

Supplement: Supplementary file 6 — Supplementary Data 3 [file 41467_2024_52061_MOESM6_ESM.zip › LC-MS-spectra/KLHDC2/Z8381047072.PDF]

MaxPeak: 100.00%  
Ret\_Time: 0.975 min

BA888194\$3

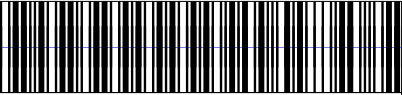

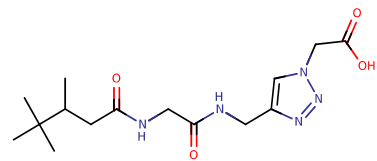

Mol Wt 339.39  
Exact Mass 339.21

| # | Time  | Area%  |
|---|-------|--------|
| 1 | 0.975 | 100.00 |

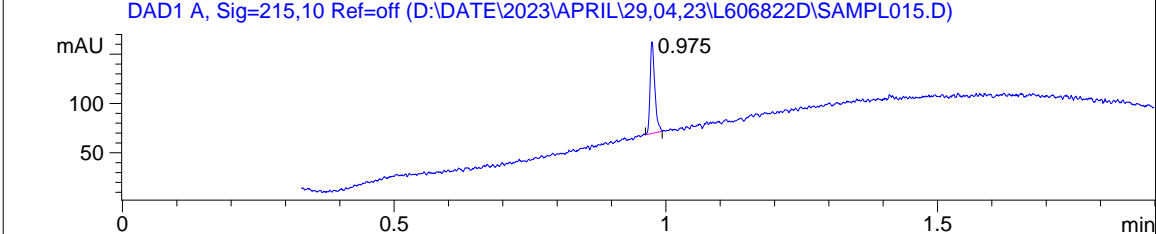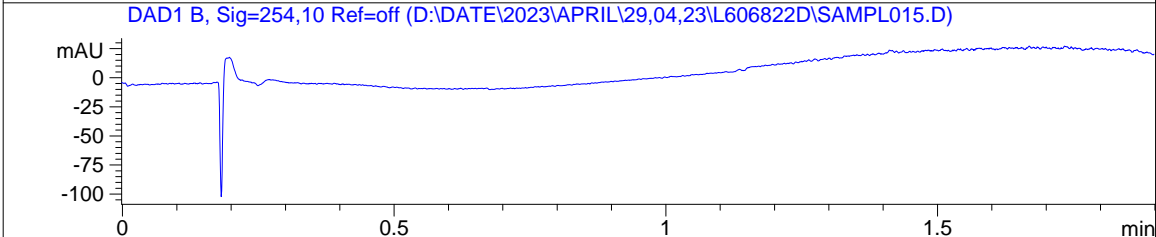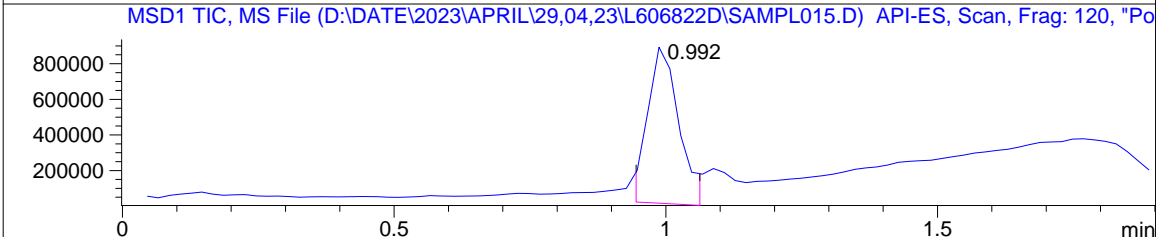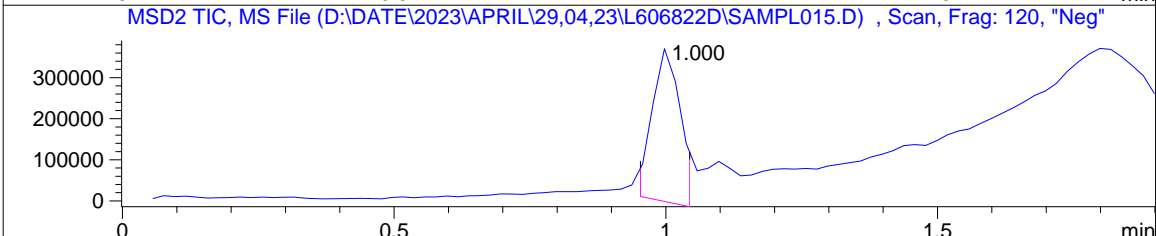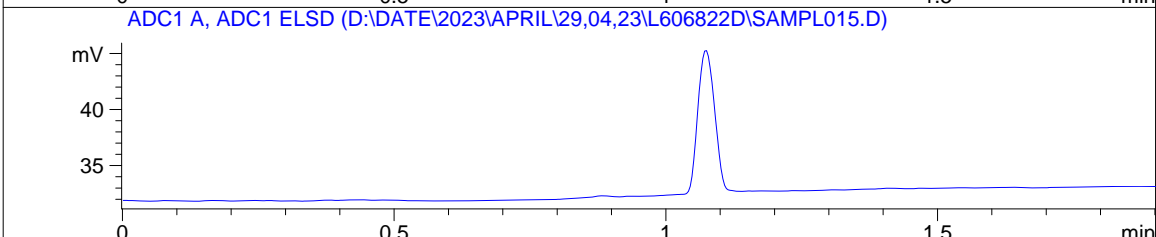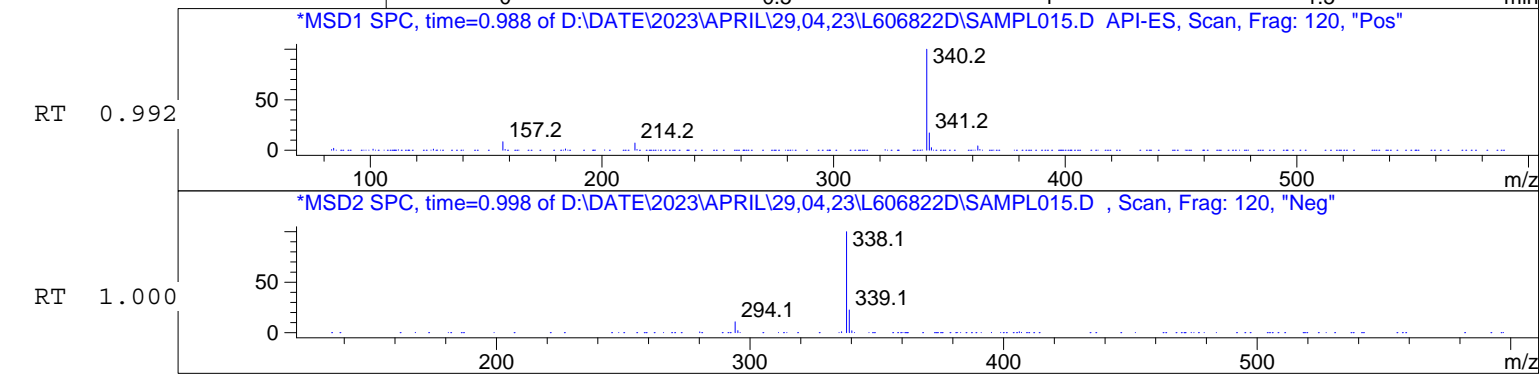

Supplement: Supplementary file 6 — Supplementary Data 3 [file 41467_2024_52061_MOESM6_ESM.zip › LC-MS-spectra/KLHDC2/Z8381047265.PDF]

BA888165\$4

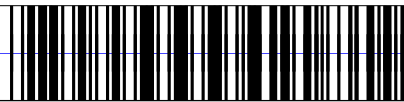

MaxPeak: 100.00%  
Ret\_Time: 0.899 min

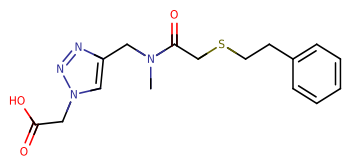

Mol Wt 348.42  
Exact Mass 348.14

| # | Time  | Area%  |
|---|-------|--------|
| 1 | 0.899 | 100.00 |

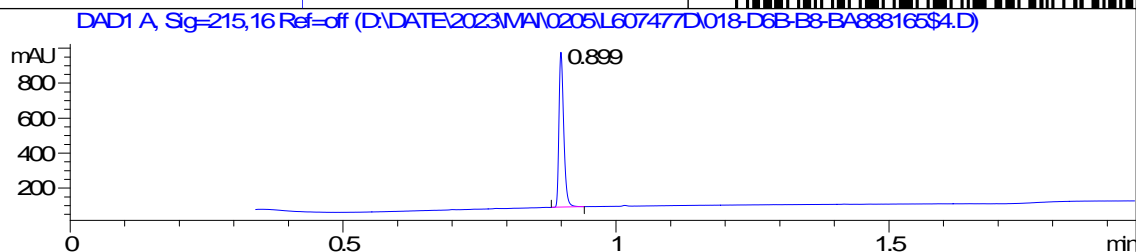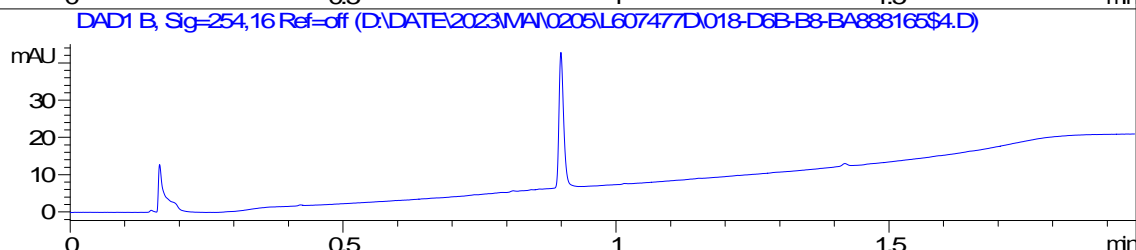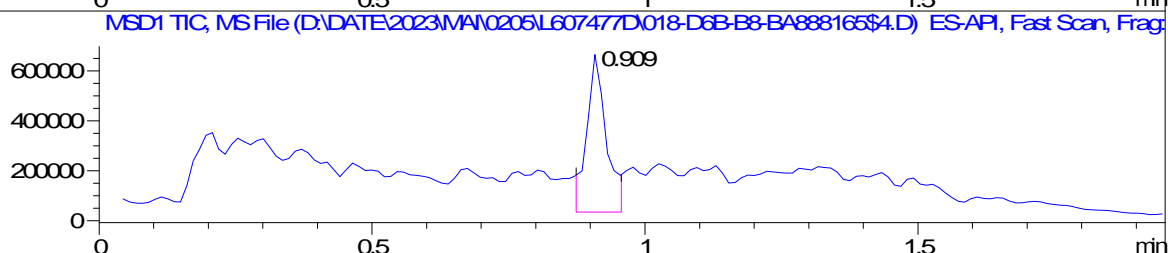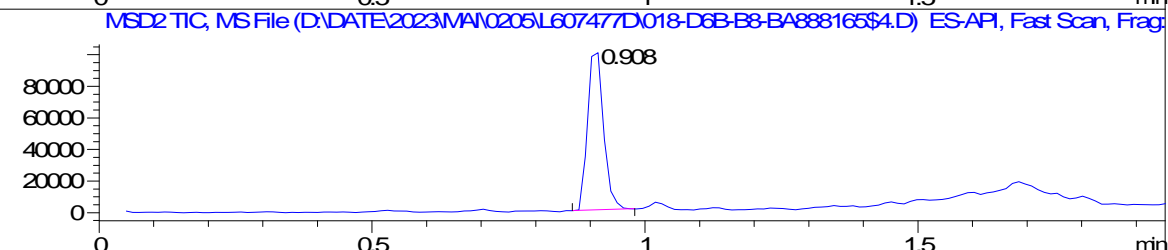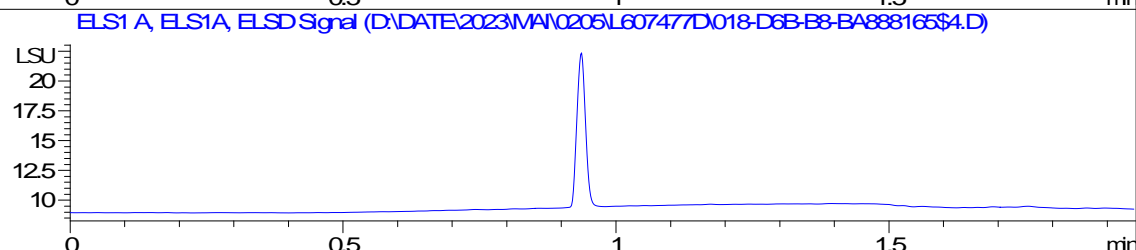

RT 0.909

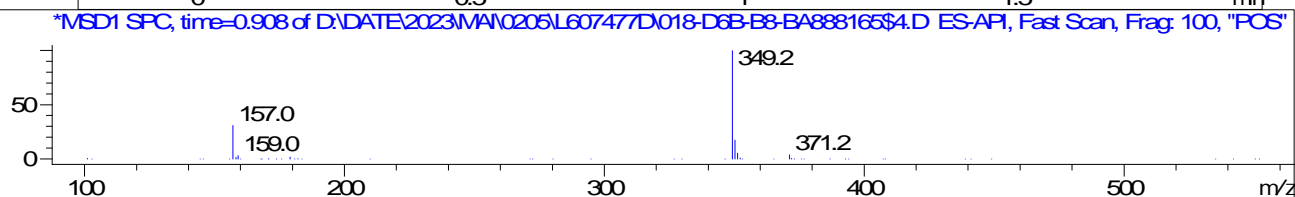

RT 0.908

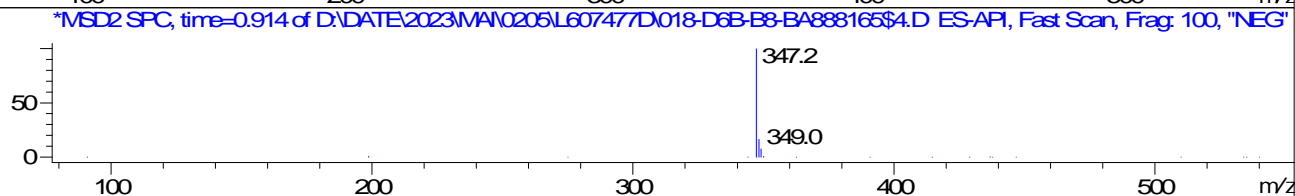

Supplement: Supplementary file 6 — Supplementary Data 3 [file 41467_2024_52061_MOESM6_ESM.zip › LC-MS-spectra/KLHDC2/Z8381047065.PDF]

BA888178\$1

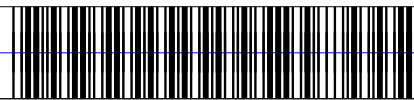

MaxPeak: 97.24%  
Ret\_Time: 0.611 min

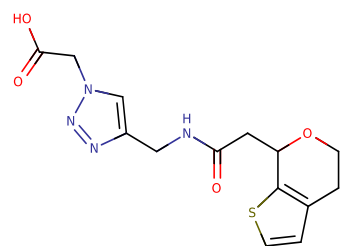

Mol Wt 336.37  
Exact Mass 336.09

| # | Time  | Area% |
|---|-------|-------|
| 1 | 0.611 | 97.24 |
| 2 | 0.715 | 2.76  |

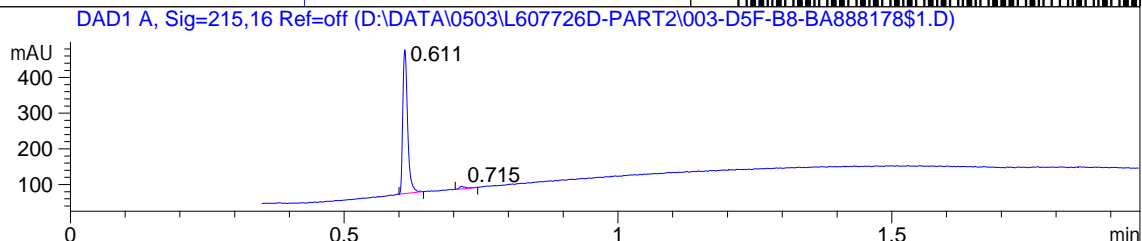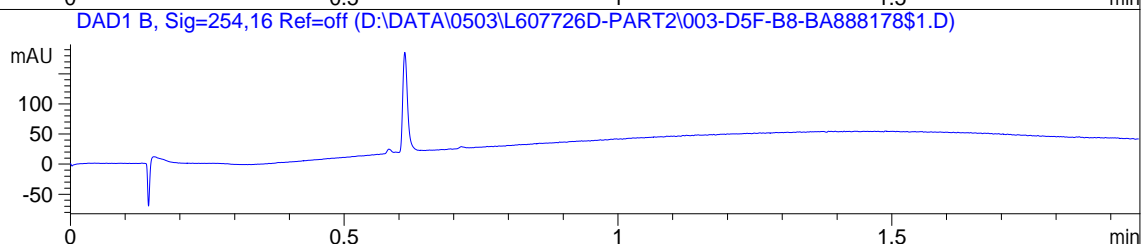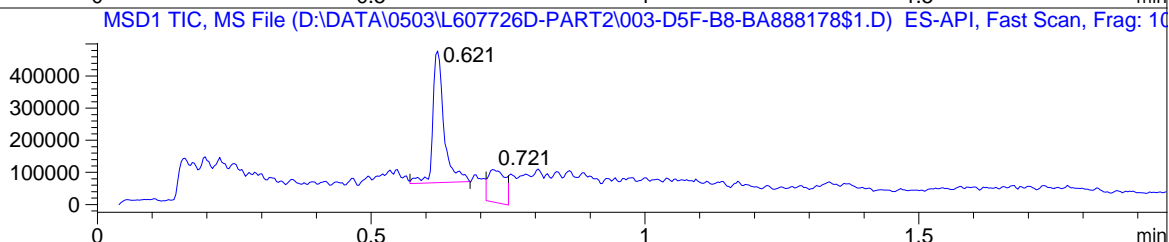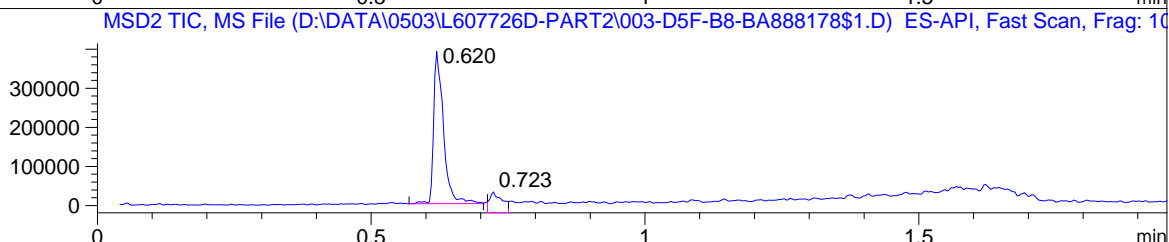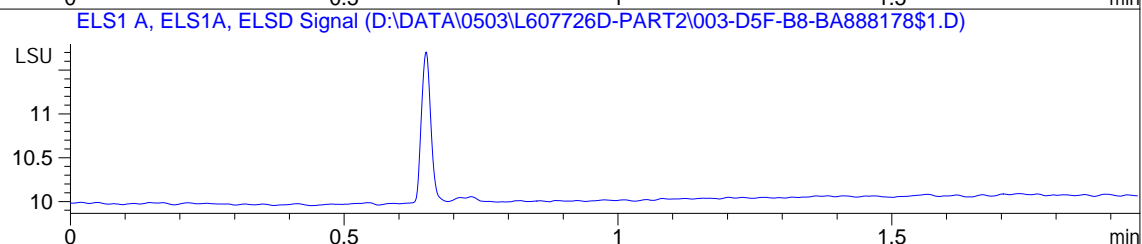

RT 0.621

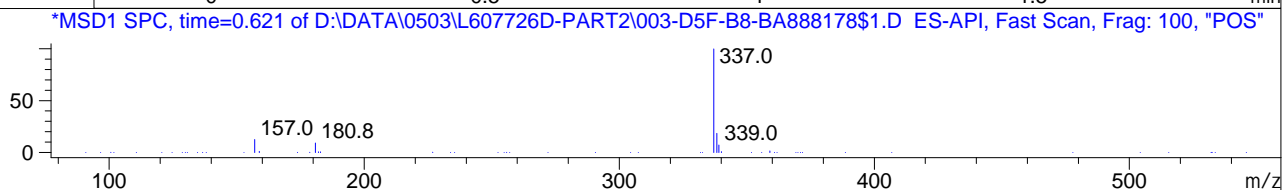

RT 0.721

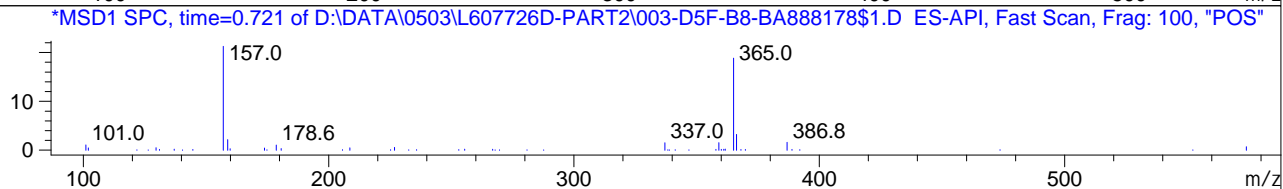

RT 0.620

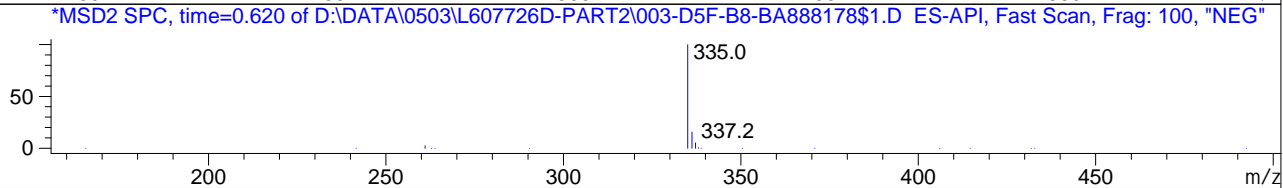

RT 0.723

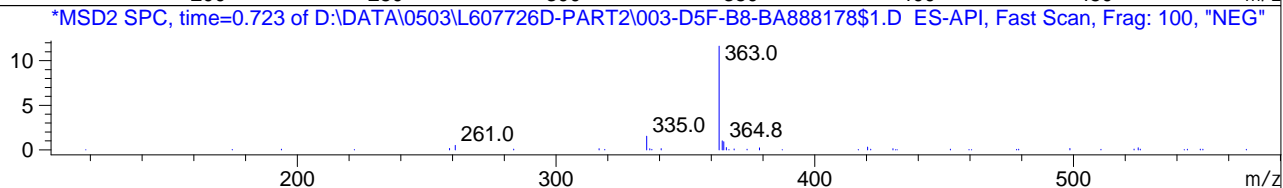

Supplement: Supplementary file 6 — Supplementary Data 3 [file 41467_2024_52061_MOESM6_ESM.zip › LC-MS-spectra/KLHDC2/Z1991864395.PDF]

BA005637\$2

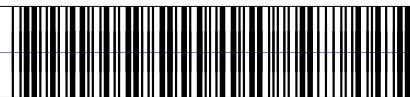

MaxPeak: 51.42%  
Ret\_Time: 2.625 min

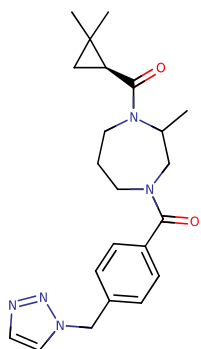

Mol Wt 395.5  
Exact Mass 395.27

| # | Time  | Area% |
|---|-------|-------|
| 1 | 2.585 | 48.58 |
| 2 | 2.625 | 51.42 |

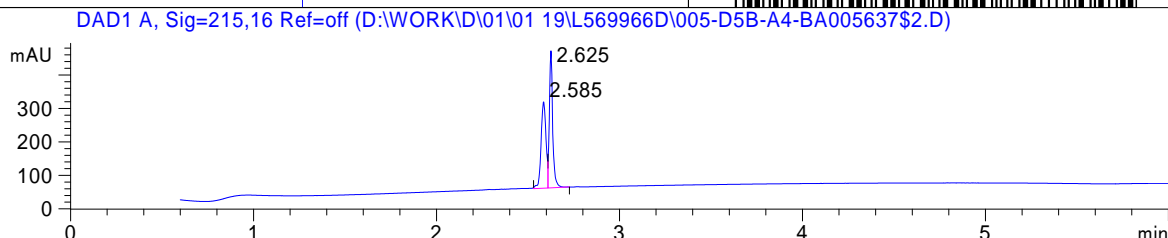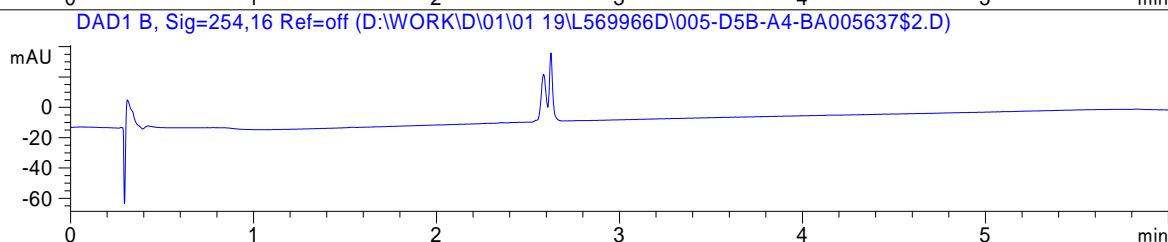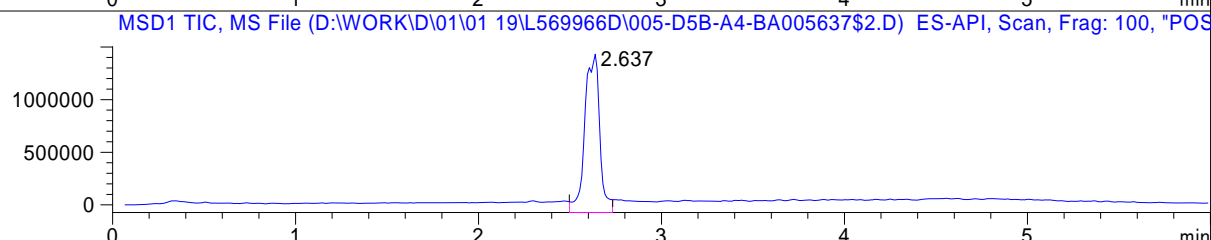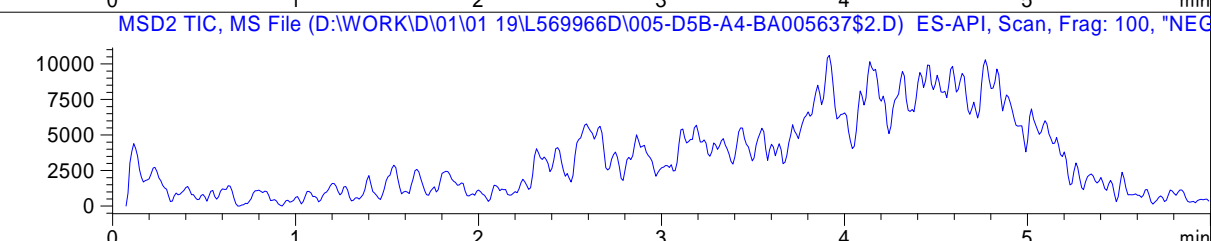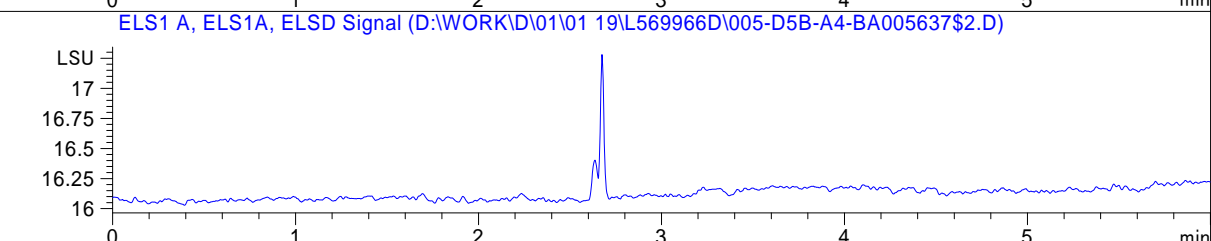

RT 2.637

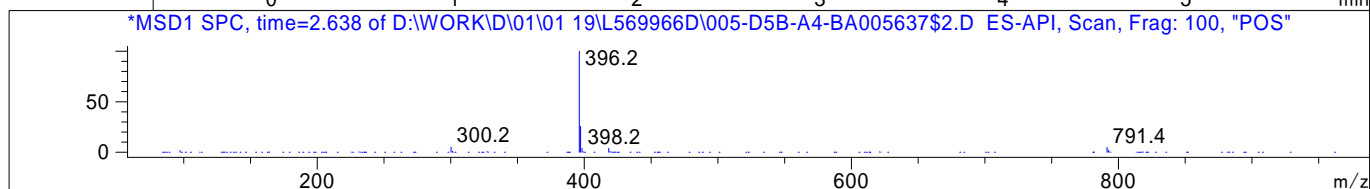

Supplement: Supplementary file 6 — Supplementary Data 3 [file 41467_2024_52061_MOESM6_ESM.zip › LC-MS-spectra/KLHDC2/Z7881785987.PDF]

MaxPeak: 100.00%  
Ret\_Time: 0.956 min

BA005655\$1

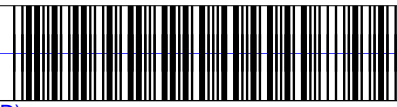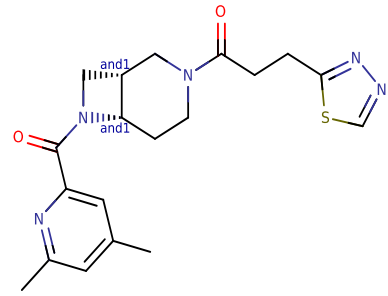

Mol Wt 385.48

Exact Mass 385.18

| # | Time  | Area%  |
|---|-------|--------|
| 1 | 0.956 | 100.00 |

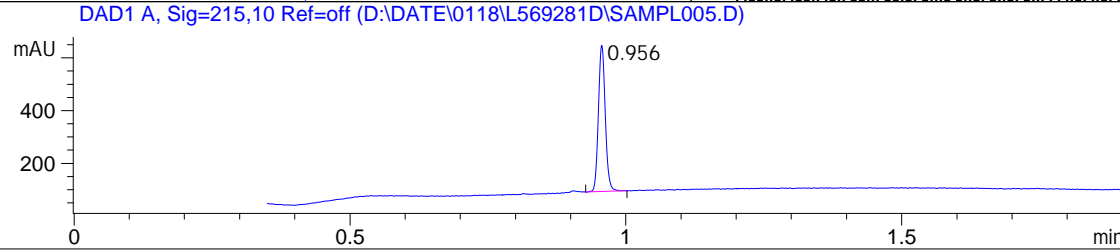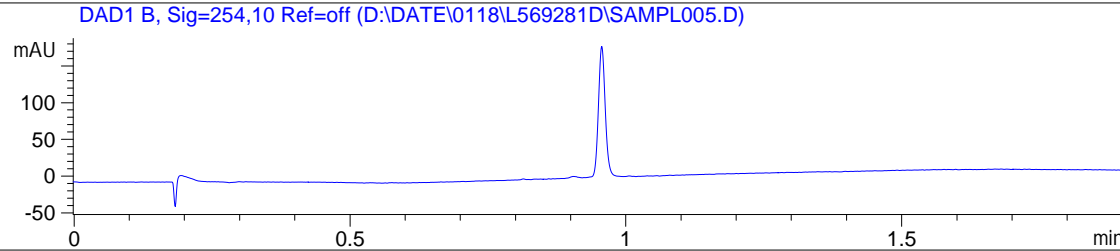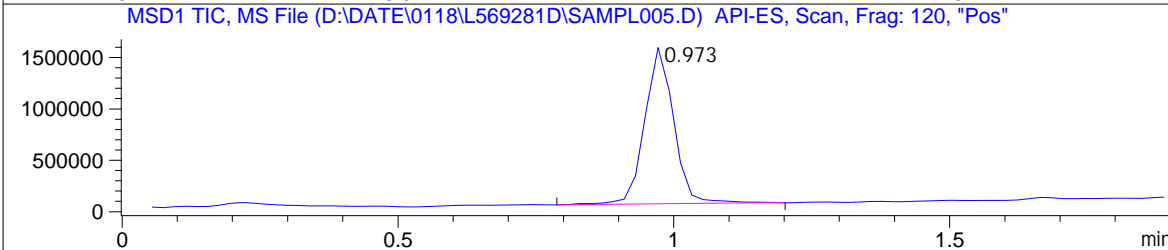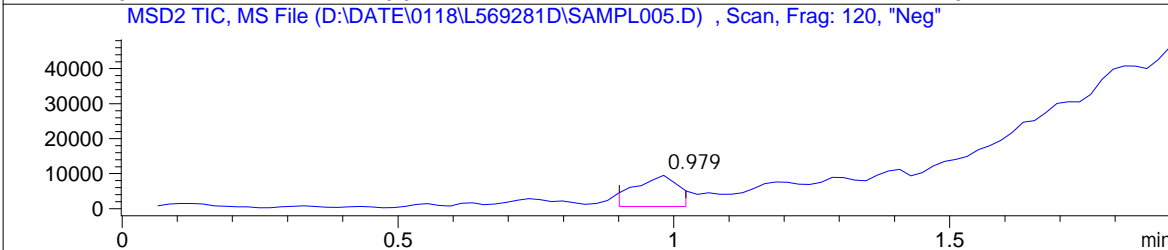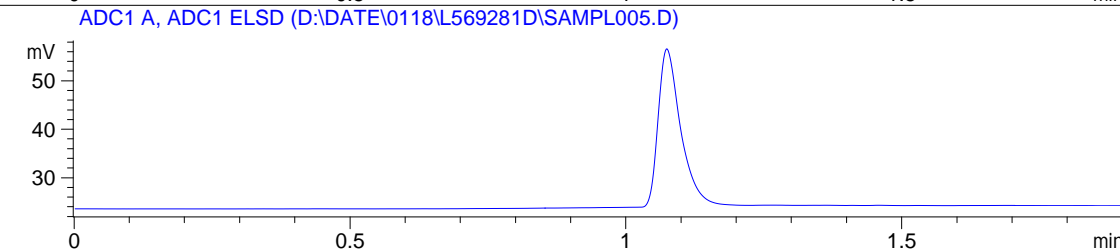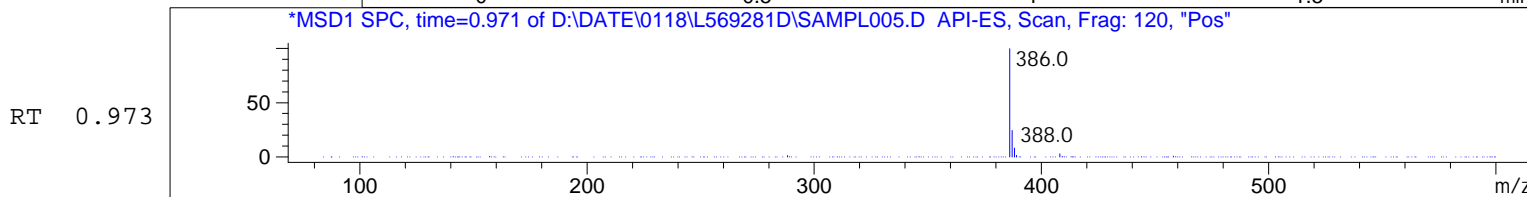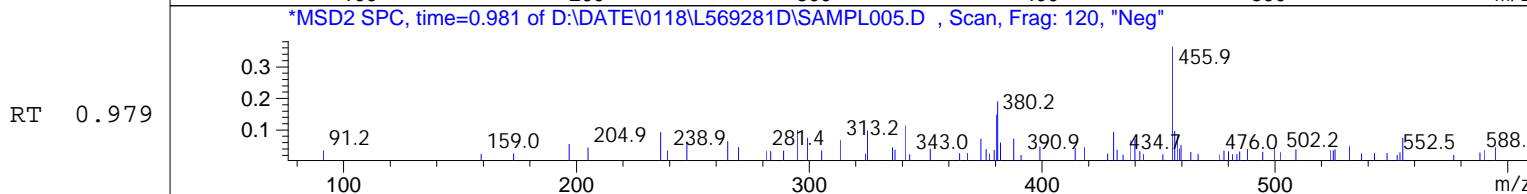

Supplement: Supplementary file 6 — Supplementary Data 3 [file 41467_2024_52061_MOESM6_ESM.zip › LC-MS-spectra/KLHDC2/Z7881785930.PDF]

Ret\_Time: 0.957 min

BA005621\$2

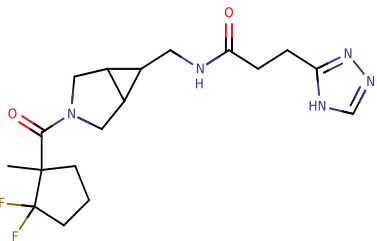

Mol Wt 381.42

**Exact Mass**      **381.23**

| # | Time  | Area% |
|---|-------|-------|
| 1 | 0.943 | 5.58  |
| 2 | 0.957 | 94.42 |

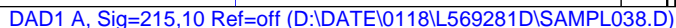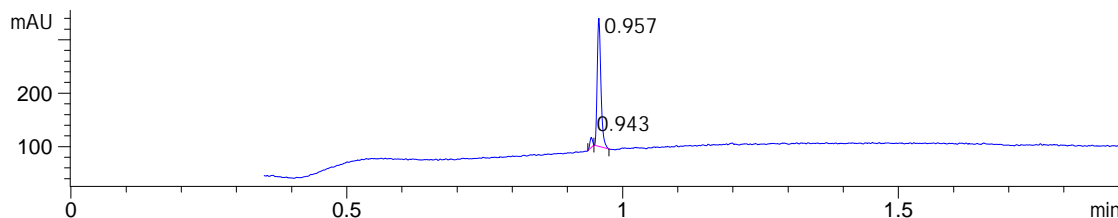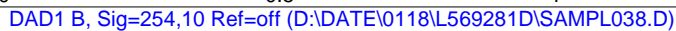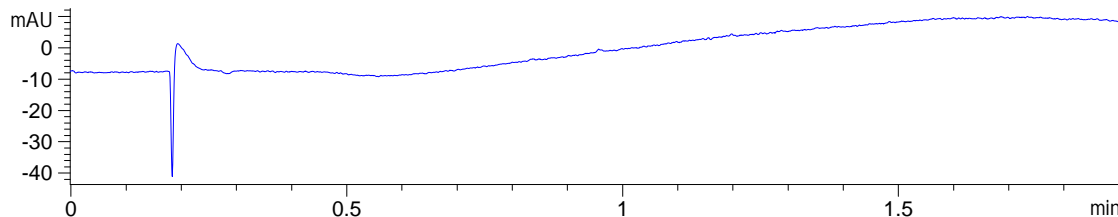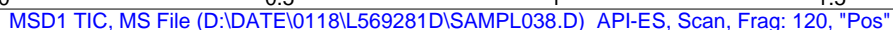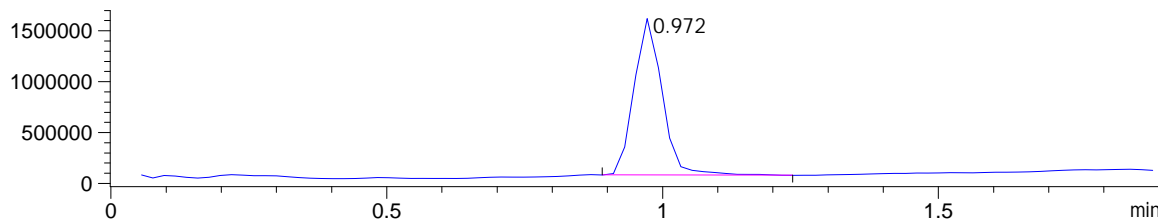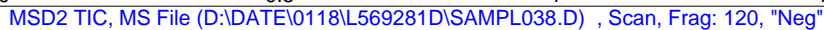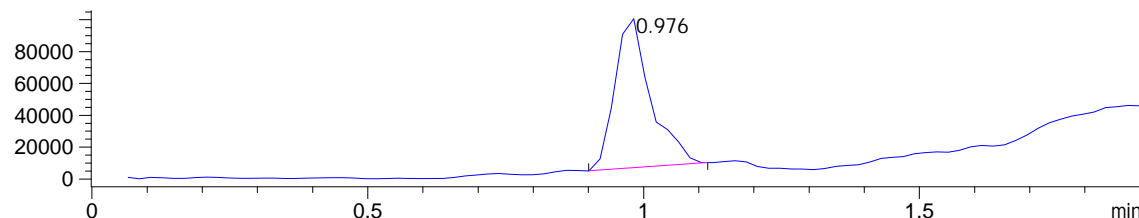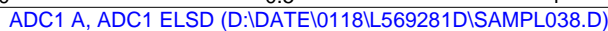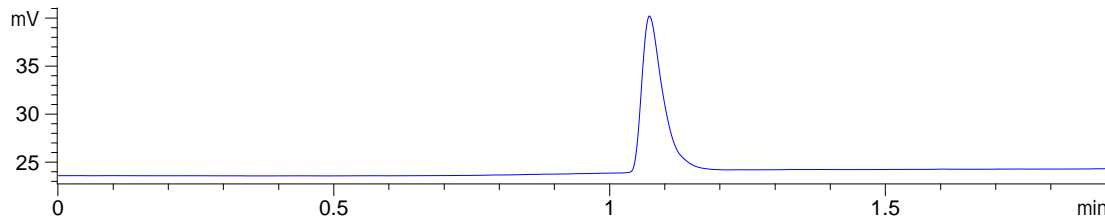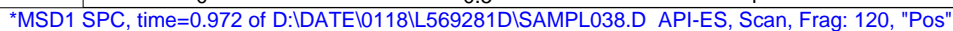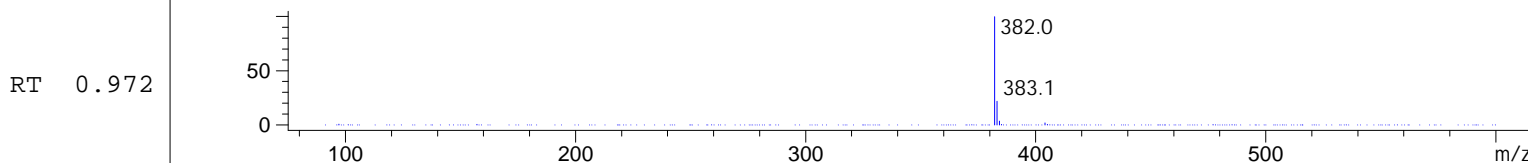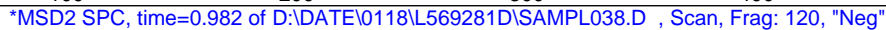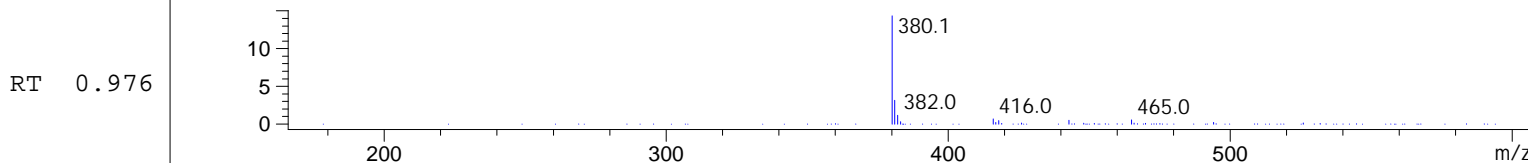

Supplement: Supplementary file 6 — Supplementary Data 3 [file 41467_2024_52061_MOESM6_ESM.zip › LC-MS-spectra/KLHDC2/Z7881785886.PDF]

MaxPeak: 100.00%  
Ret\_Time: 1.495 min

BA888192\$4

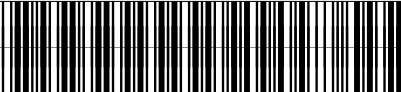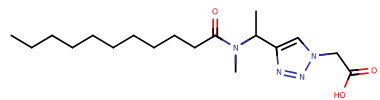

Mol Wt 352.47  
Exact Mass 352.29

| # | Time  | Area%  |
|---|-------|--------|
| 1 | 1.495 | 100.00 |

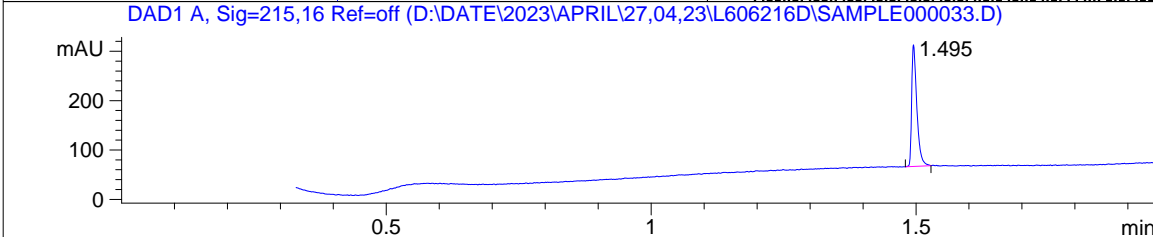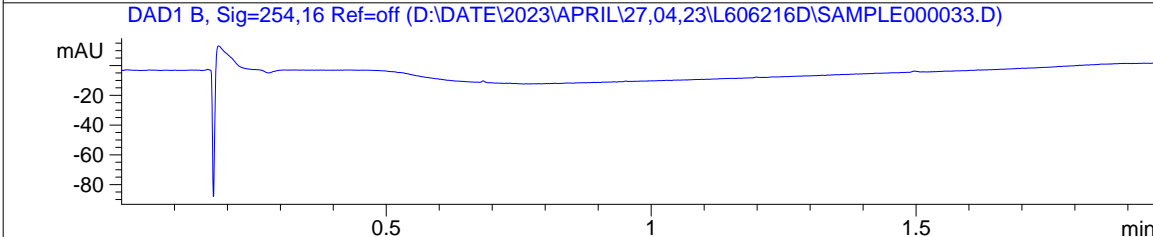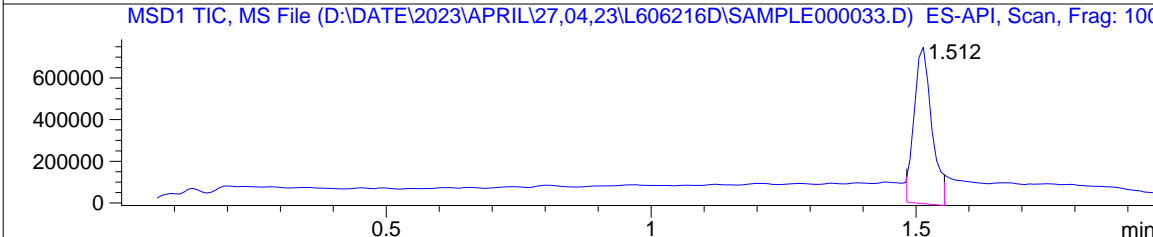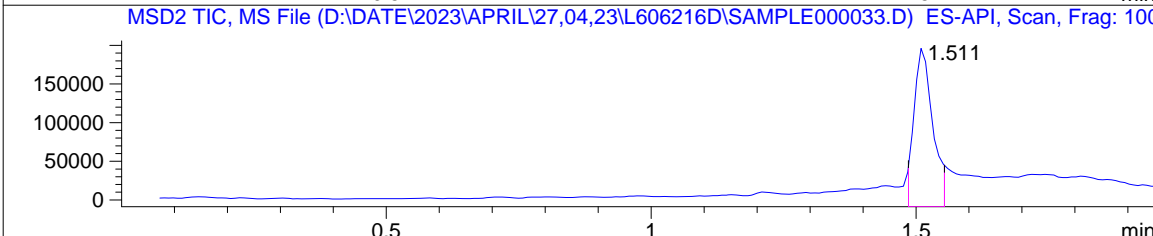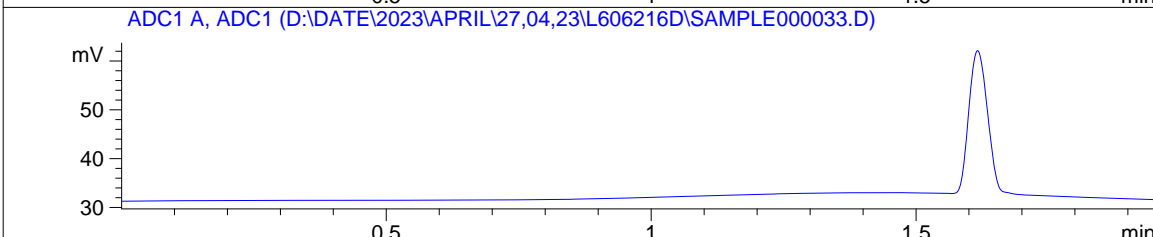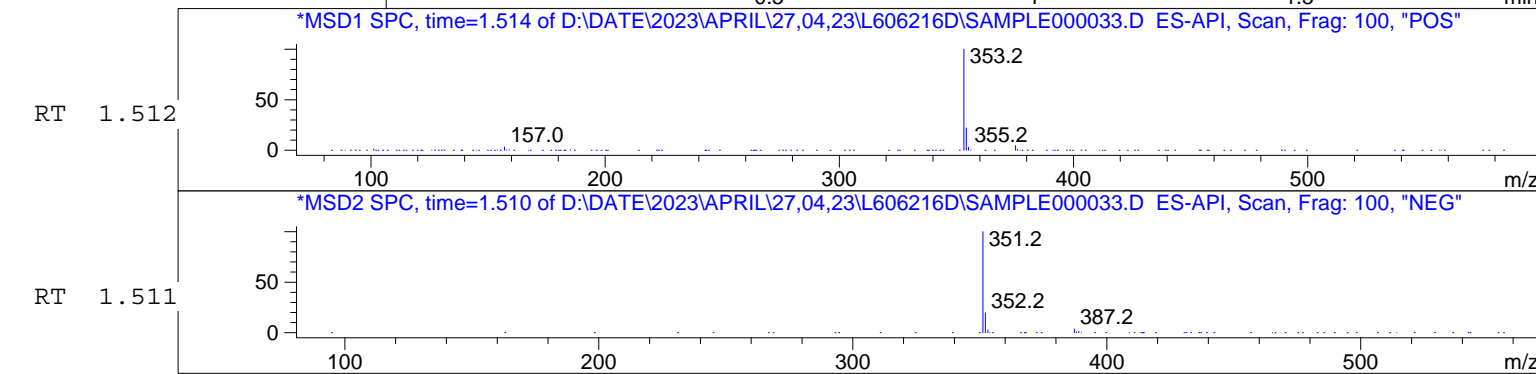

Supplement: Supplementary file 6 — Supplementary Data 3 [file 41467_2024_52061_MOESM6_ESM.zip › LC-MS-spectra/KLHDC2/Z8381047292.PDF]

BC030625\$4

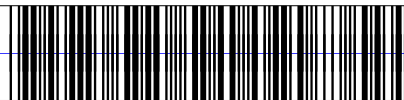

MaxPeak: 95.09%  
Ret\_Time: 1.009 min

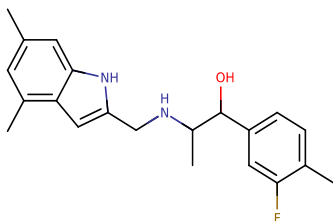

Mol Wt 340.43  
Exact Mass 340.24

| # | Time  | Area% |
|---|-------|-------|
| 1 | 0.605 | 0.46  |
| 2 | 0.873 | 0.68  |
| 3 | 0.883 | 1.23  |
| 4 | 0.936 | 1.10  |
| 5 | 1.009 | 95.09 |
| 6 | 1.130 | 0.82  |
| 7 | 1.448 | 0.61  |

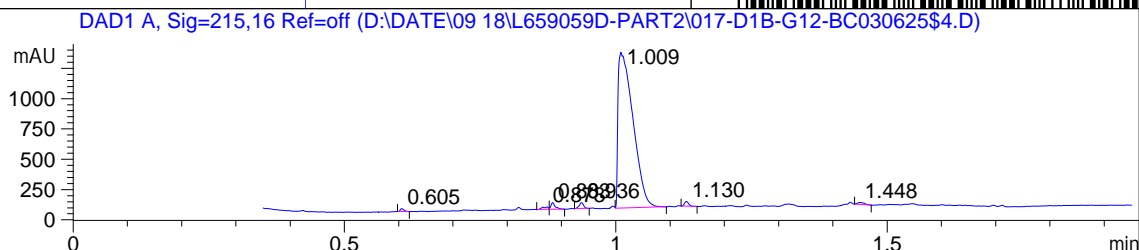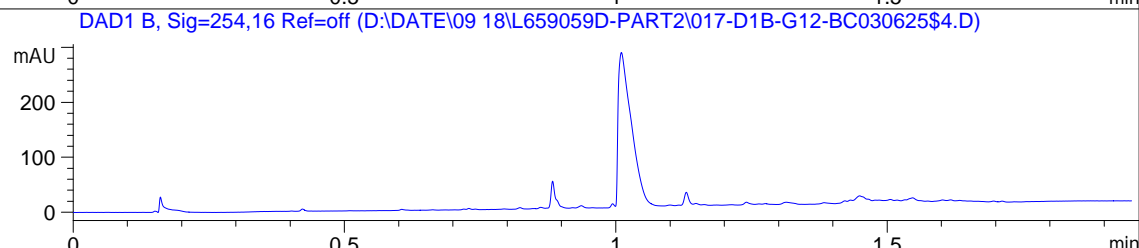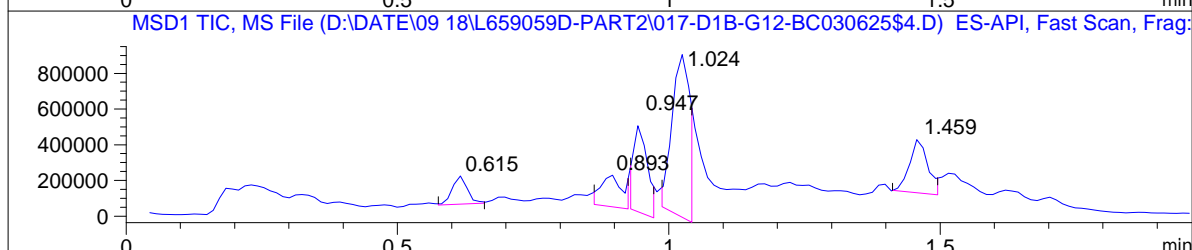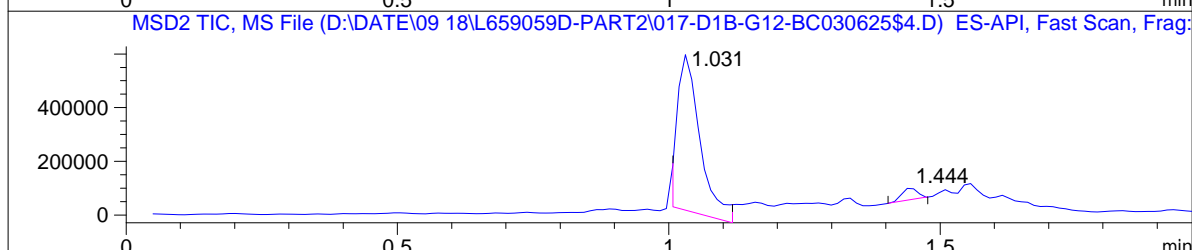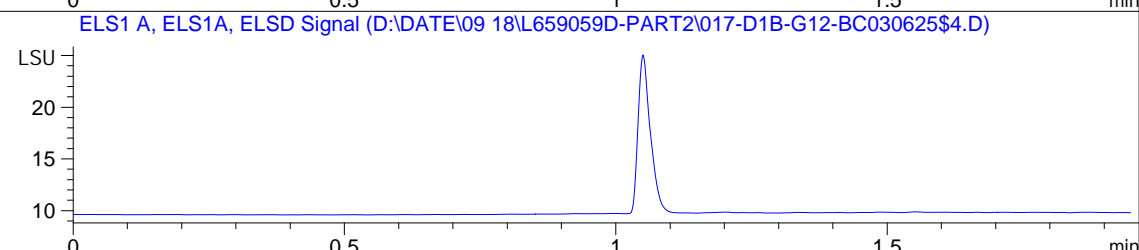

RT 0.615

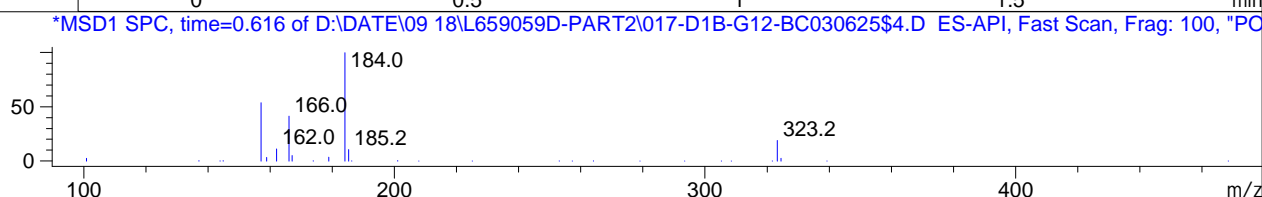

RT 0.893

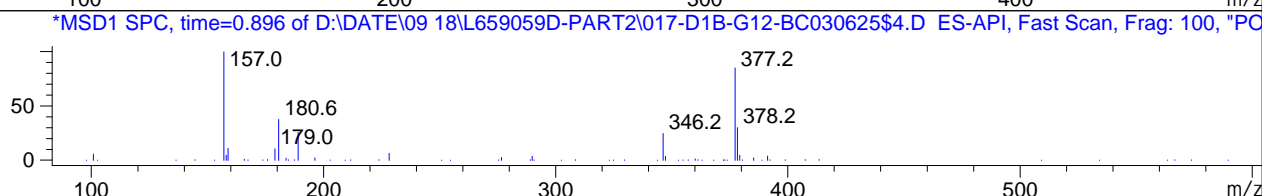

RT 0.947

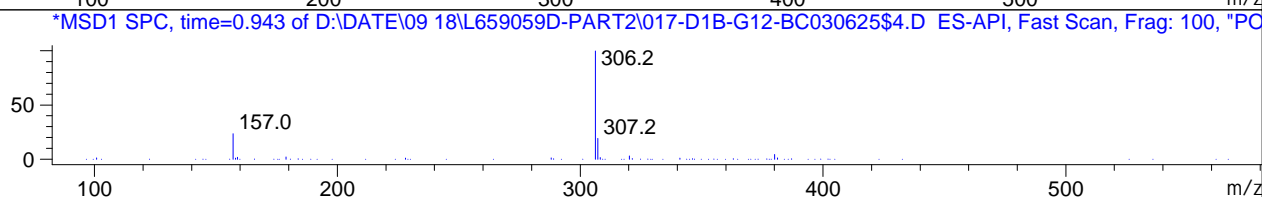

RT 1.024

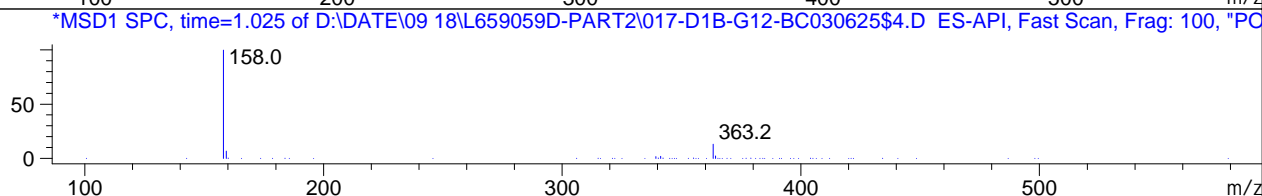

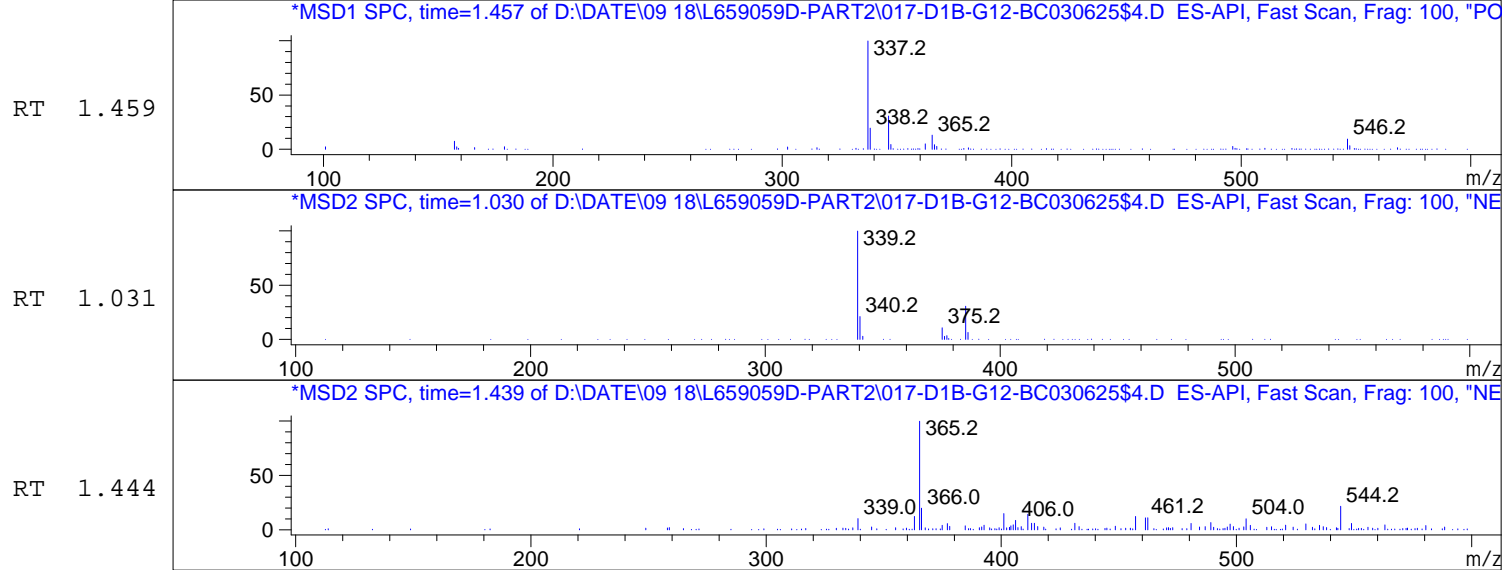

Supplement: Supplementary file 6 — Supplementary Data 3 [file 41467_2024_52061_MOESM6_ESM.zip › LC-MS-spectra/Nav1.7/Z8718594710.PDF]

BC030628\$3

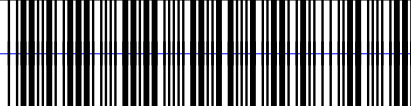

MaxPeak: 100.00%  
Ret\_Time: 1.094 min

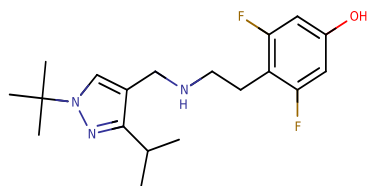

Mol Wt 351.43  
Exact Mass 351.26

| # | Time  | Area%  |
|---|-------|--------|
| 1 | 1.094 | 100.00 |

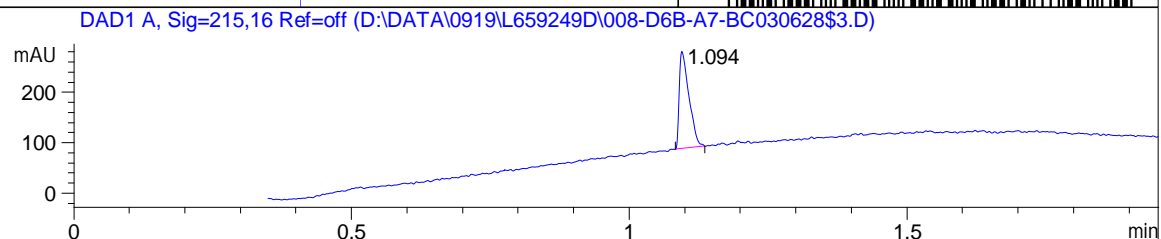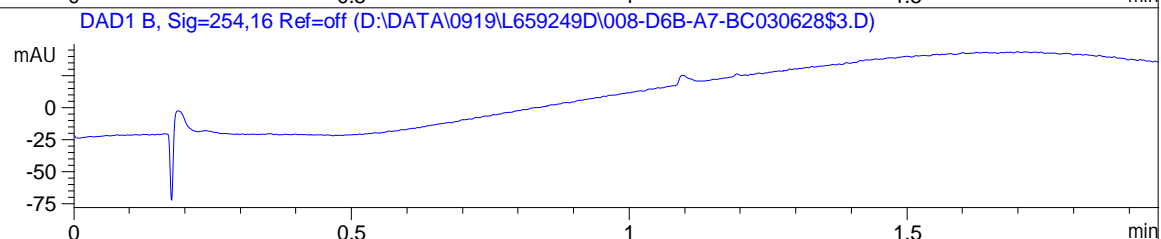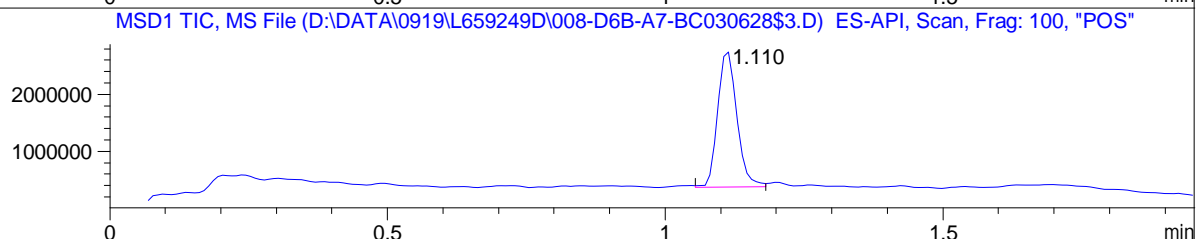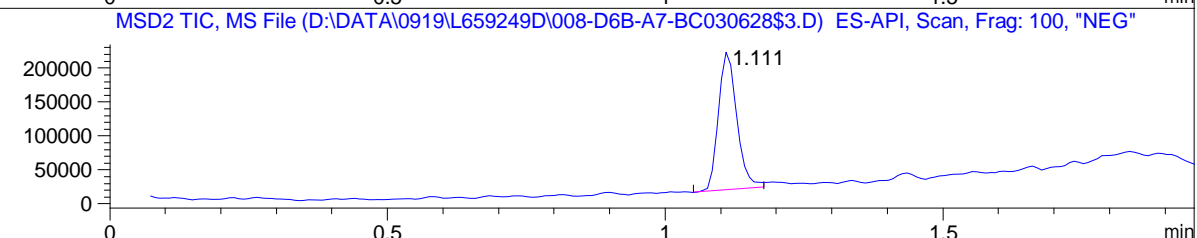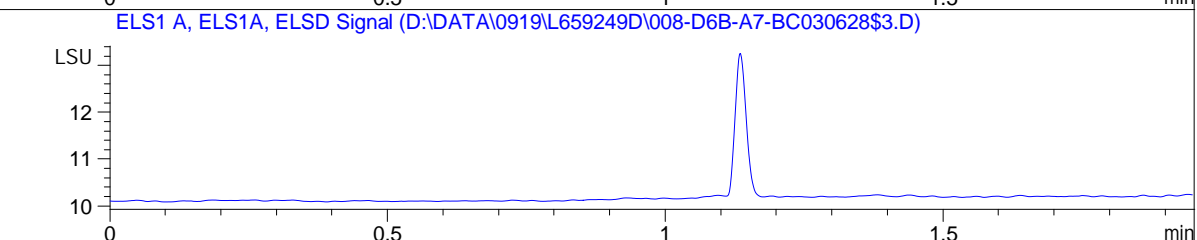

RT 1.110

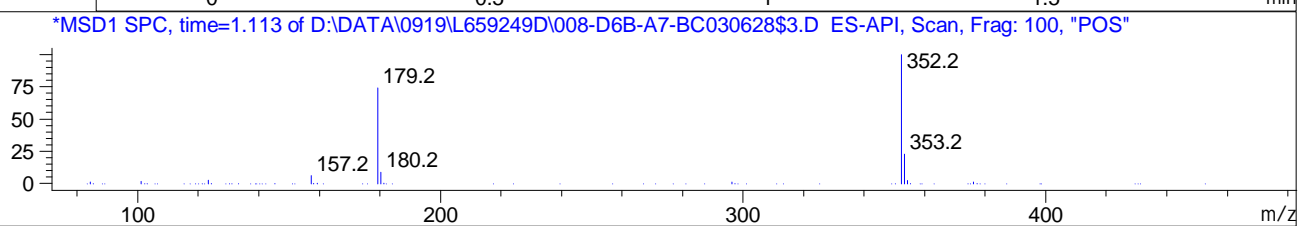

RT 1.111

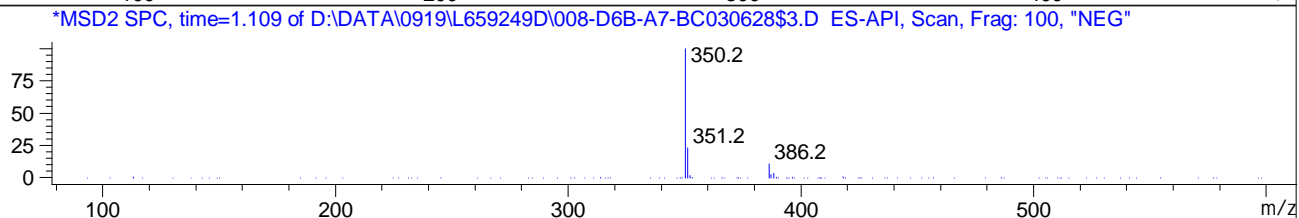

Supplement: Supplementary file 6 — Supplementary Data 3 [file 41467_2024_52061_MOESM6_ESM.zip › LC-MS-spectra/Nav1.7/Z8718594704.PDF]
